# Supplementary material for: Structure of Complement C3(H2O) Revealed By Quantitative Cross-Linking/Mass Spectrometry And Modeling
Source: Mol Cell Proteomics. 2016 Jun 1;15(8):2730–43. doi: 10.1074/mcp.M115.056473 (PMC4974347; doi:10.1074/mcp.M115.056473)
Supplement: Supplemental Data [file supp_15_8_2730__index.html]

Structure of complement C3(H2O) revealed by quantitative cross-linking/mass spectrometry and modelling — Structure of Complement C3(H2O) Revealed By Quantitative Cross-Linking/Mass Spectrometry And Modeling — QCLMS and Modeling Reveals Structure of C3(H2O) — Supplemental Data 

# Structure of Complement C3(H2O) Revealed By Quantitative Cross-Linking/Mass Spectrometry And Modeling

## Supplemental Data

- Supplemental Table S1 (.xlsx, 36 KB) - Identification of quantified cross-linked peptides
- Supplemental Table S2 (.xlsx, 21 KB) - Quantified cross-links
- Supplemental Table S3 (.xlsx, 16 KB) - Quantified cross-links used for integrative modeling
- Supplemental File S1 (.pdf, 4.9 MB) - Supplemental Figure S1, S2 and S3. Annotated best matched MS2 spectra of identified and quantified cross-linked peptides.
