# Supplementary material for: Structure of Complement C3(H2O) Revealed By Quantitative Cross-Linking/Mass Spectrometry And Modeling
Source: Mol Cell Proteomics. 2016 Jun 1;15(8):2730–43. doi: 10.1074/mcp.M115.056473 (PMC4974347; doi:10.1074/mcp.M115.056473)
Supplement: Supplemental Data [file 10.1074_M115.056473_mcp.M115.056473-4.pdf]

Supplemental Information

Structure of complement $C3(H_2O)$ revealed by quantitative cross-linking/mass spectrometry and modelling

Zhuo A. Chen, Riccardo Pellarin, Lutz Fischer, Andrej Sali, Michael Nilges,
Paul N. Barlow*, Juri Rappsilber*

Page 2-3	Supplemental Figure S1. Domain arrangements of C3 and C3b in the crystal structures.
Page 4-5	Supplemental Figure S2. Assessment of cross-link site assignment.
Page 6-7	Supplemental Figure S3. Clustering of $C3(H_2O)$ solutions.
Page 8-187	Annotated best-matched MS2 spectrum for identified cross-linked peptides

Supplemental Figure 1

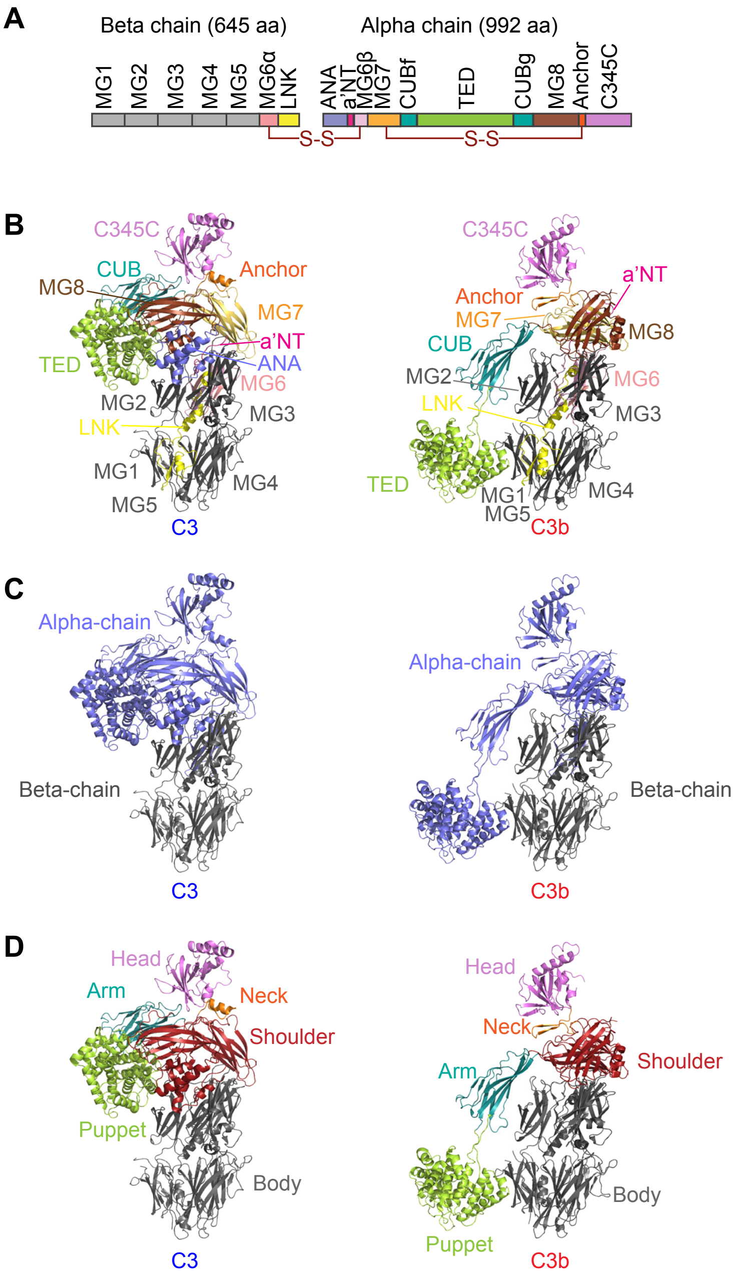

Supplemental Figure 1: Domain arrangements of C3 and C3b in the crystal structures

(**A**) Summary of the multiple domains of C3 (24). (**B**) Domains of C3 and C3b are coloured and labelled in the crystal structures of C3 (PDB|2A73) and C3b (PDB|2I07) (the same PDB entries are used for C and D). (**C**) The α-chain and the β-chain of C3/C3b are coloured and labelled in the crystal structures of C3 and C3b (**D**) The crystal structures of C3 and C3b are coloured and labelled according to the metaphor of a puppeteer (1).

1. Janssen, B. J., Christodoulidou, A., McCarthy, A., Lambris, J. D., and Gros, P. (2006) Structure of C3b reveals conformational changes that underlie complement activity. Nature 444, 213-216

Supplemental Figure S2

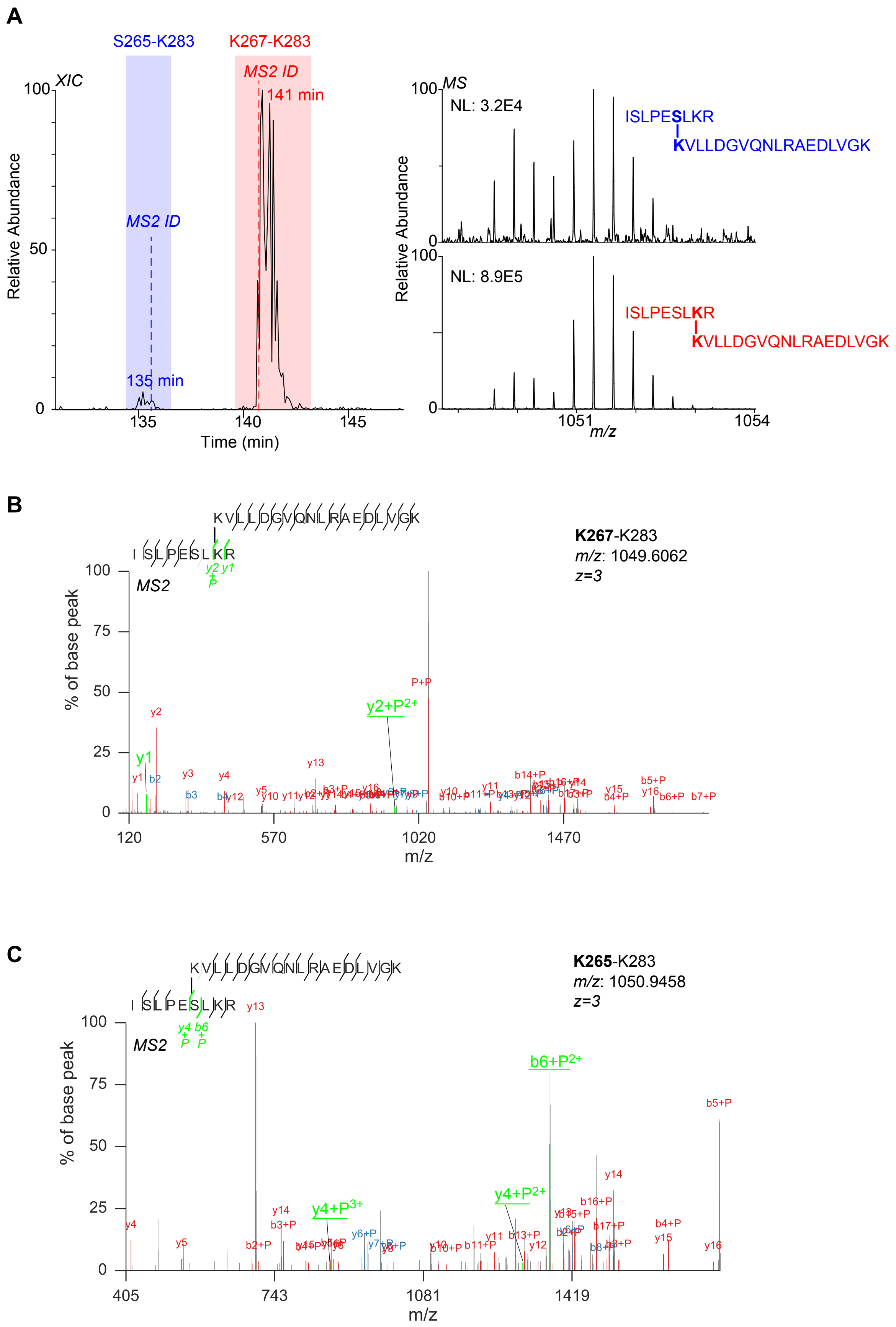

Supplemental Fig S2. Assessment of cross-link site assignment

Assessment of site assignment of cross-linked peptides is demonstrated using an example. (A)The peptides ISLPESLKR and KVLLDGVQNLRAEDLVGK were found cross-linked in two ways, once linking 267K and 283K and once linking 265S and 283K. The 267K – 283K linked peptide eluted with a retention time of 141 minute from our HPLC system and was assigned this linkage based on back-bone fragments y1 and y2+P ions that flank 267K (annotated spectrum shown in(B)). 265S – 283K is placed in close sequence proximity to 267K – 283K. Given the much higher reactivity of NHS-esters to K when compared to S, special caution needed to be employed when calling 265S – 283K. 265S is supported over 267K in the matching fragmentation spectrum by the observation of b6+P and y4+P ions that flank 265S (annotated spectrum shown in (C)). In addition, the 265S – 283K peptide eluted at 135 minute in a base line separated peak from our HPLC system. Notably, the intensity of this peptide is much lower than that carrying the 267K – 283K link as one might expect considering the reactivity difference of S and K.

Supplemental Figure S3

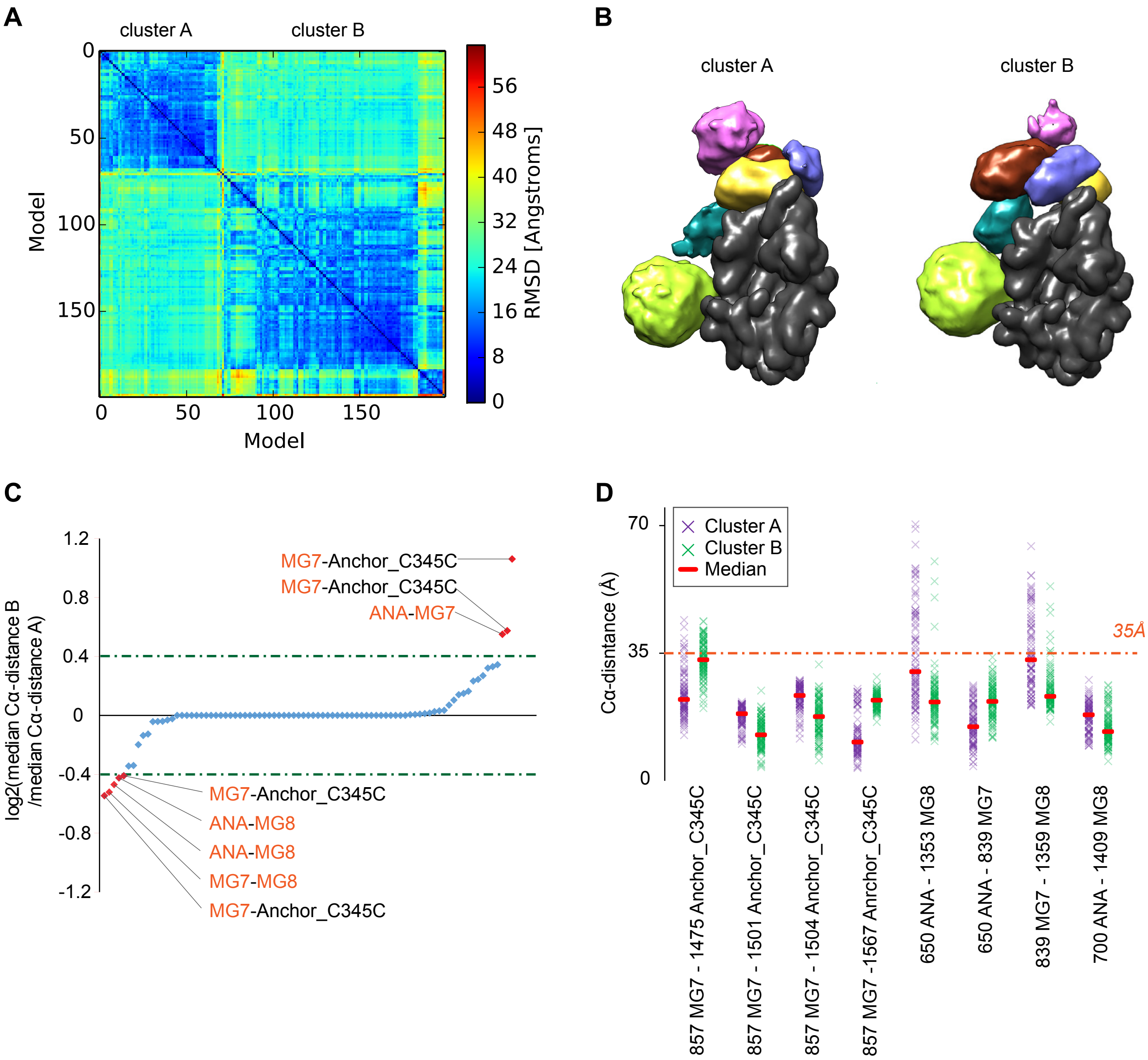

Supplementary Figure 3. Clustering of C3(H_2O) solutions.

The 200 best scoring models (solutions) were partitioned into clusters, using the k-means clustering method. The two blue blocks of the pair-wise r.m.s.-distance matrix calculated between the 200 models **(A**) suggested the presence of at least two main clusters of similar structures. The resulting clusters (**B**) displayed a different orientation of MG8 (brown density) and ANA (violet density), with respect to the other domains. A comparison of the distances between cross-linked residues, in cluster A *versus* cluster B, is shown in (**C**). For each cross-link (displayed along the *x*-axis) the ratio (for cluster A *versus* cluster B) of median Cα-Cα distances is shown in the *y*-axis (log scale). Distances that differ most between the two clusters (red) all involve domains at the “shoulder” region of the molecule (ANA, MG7 and MG8). (**D**) For those cross-linked pairs of residues for which mean Cα-Cα distances differed between clusters A and B (highlighted in red in (**C**)), the Cα-Cα distance for every solution (shown as crosses, purple for cluster A and green for cluster B) are plotted. The median of the distribution is represented by a red line. Cluster B show better satisfaction on residue proximities defined by cross-links.

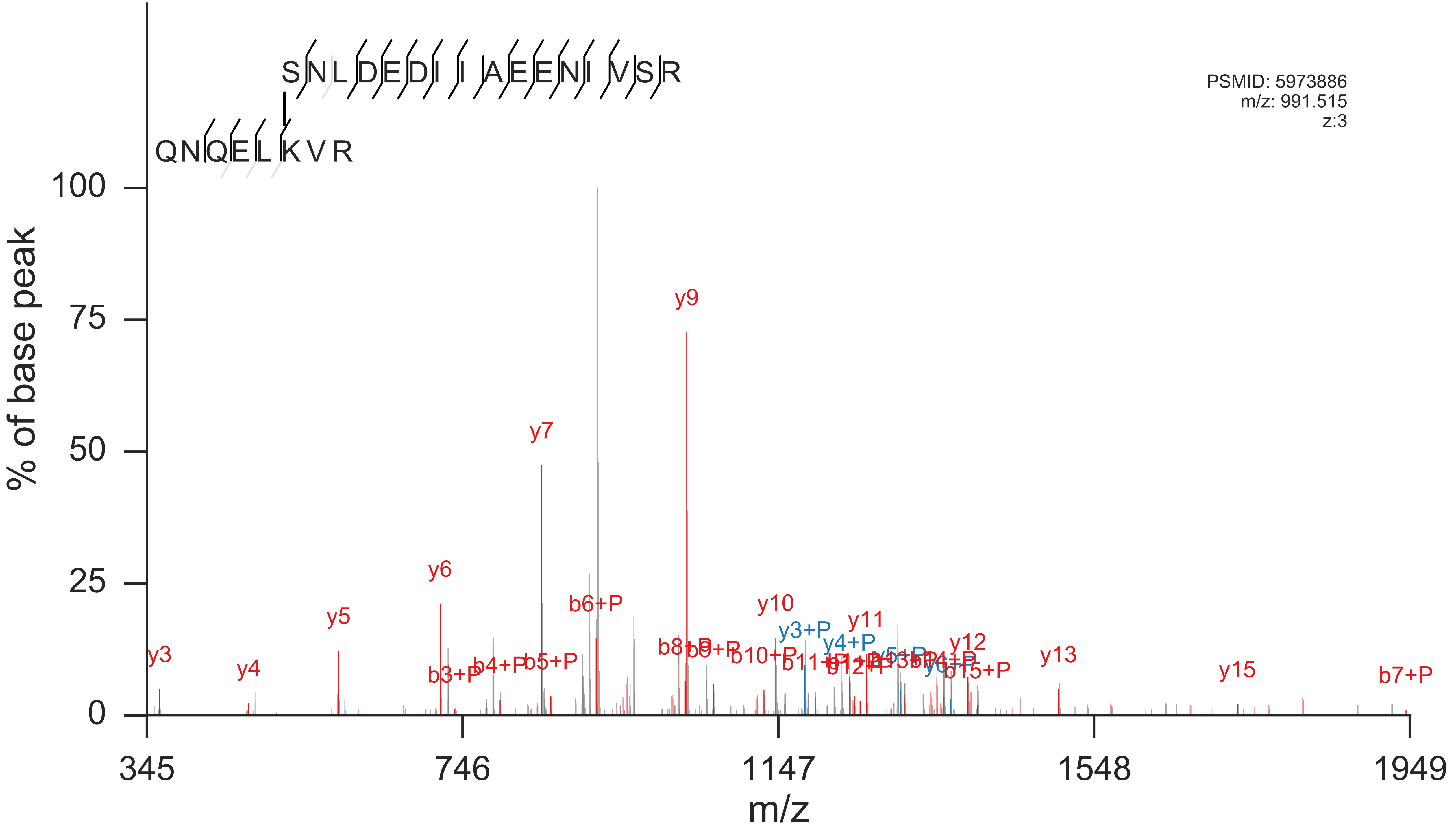

SNLDEDIIAEENIVSR
QNQELKVR
PSMID: 5973886
m/z: 991.515
z:3
% of base peak
m/z
100
75
50
25
0
345
746
1147
1548
1949
y3
y4
y5
y6
y7
y9
y10
y11
y12
y13
y15
b3+P
b4+P
b5+P
b6+P
b7+P
b10+P
b12+P
b15+P
y3+P
y4+P

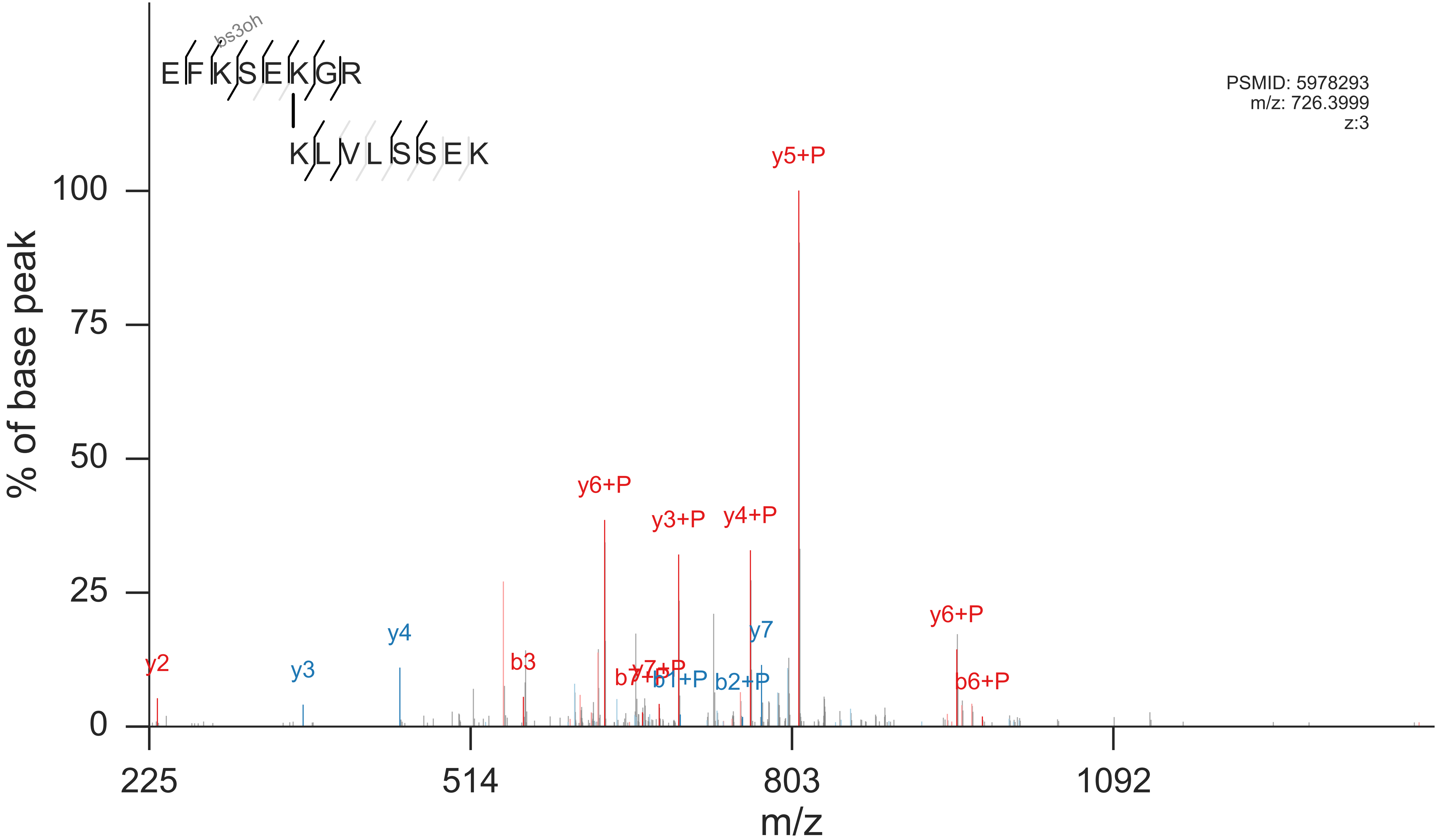
EFKSEKGR
KLVLSSEK
bs3oh
PSMID: 5978293
m/z: 726.3999
z:3
% of base peak
m/z
100
75
50
25
0
225
514
803
1092
y5+P
y6+P
y3+P
y4+P
y7
y6+P
y2
y3
y4
b3
b7+P
y7+P
b1+P
b2+P
b6+P

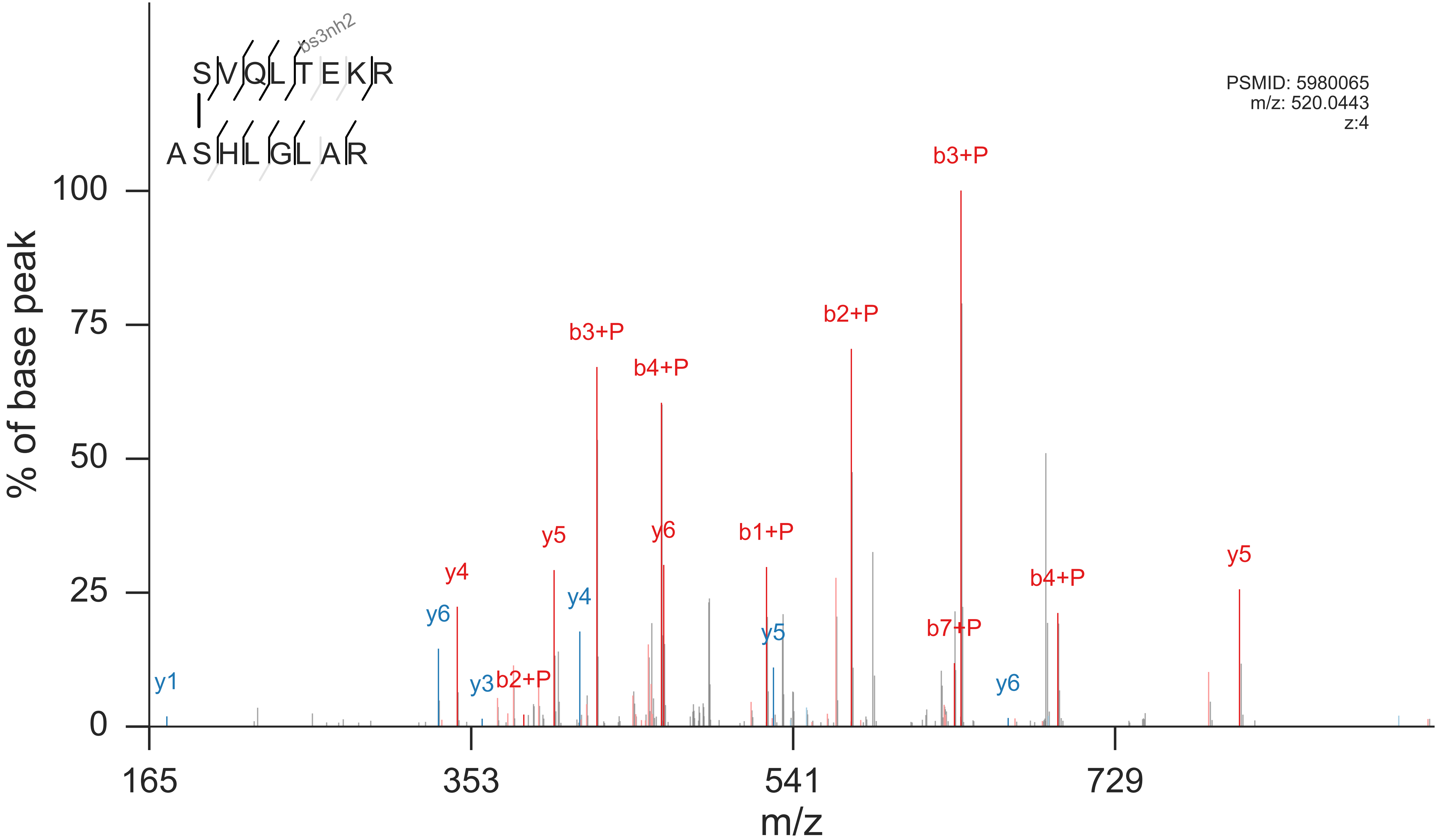

bs3nh2
SVQLTEKR
ASHLGLAR
PSMID: 5980065
m/z: 520.0443
z:4
% of base peak
m/z
100
75
50
25
0
165
353
541
729
y1
y6
y4
y3
b2+P
y5
b3+P
y4
b4+P
y6
b1+P
y5
b2+P
b3+P
b7+P
y6
b4+P
y5

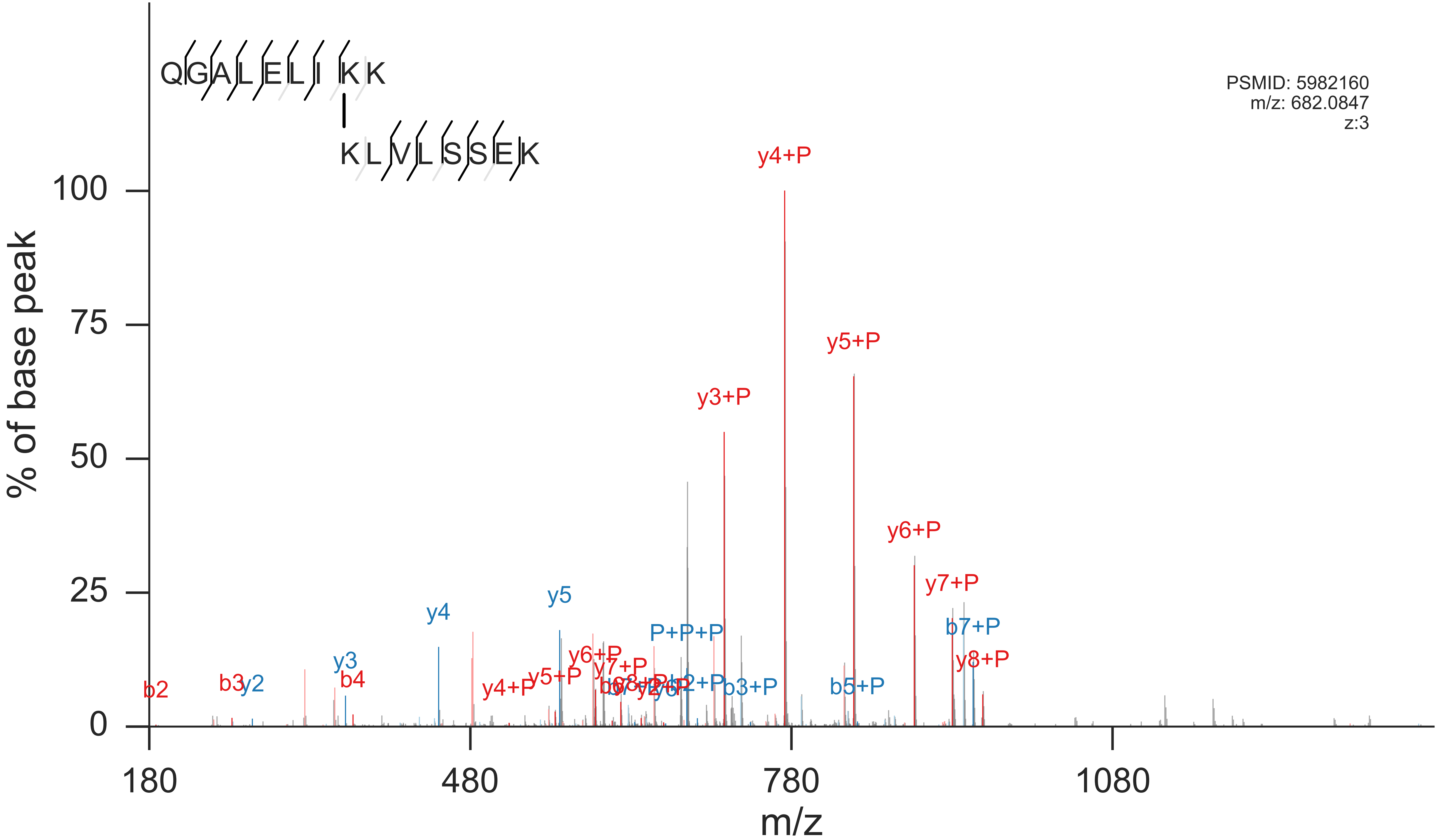
QGALELIKK
KLVLSSEK
PSMID: 5982160
m/z: 682.0847
z:3
% of base peak
m/z
y4+P
y5+P
y3+P
y6+P
y7+P
b7+P
y8+P
P+P+P
y5
y4
y3
b4
b3
y2
b2
b3+P
b5+P
100
75
50
25
0
180
480
780
1080

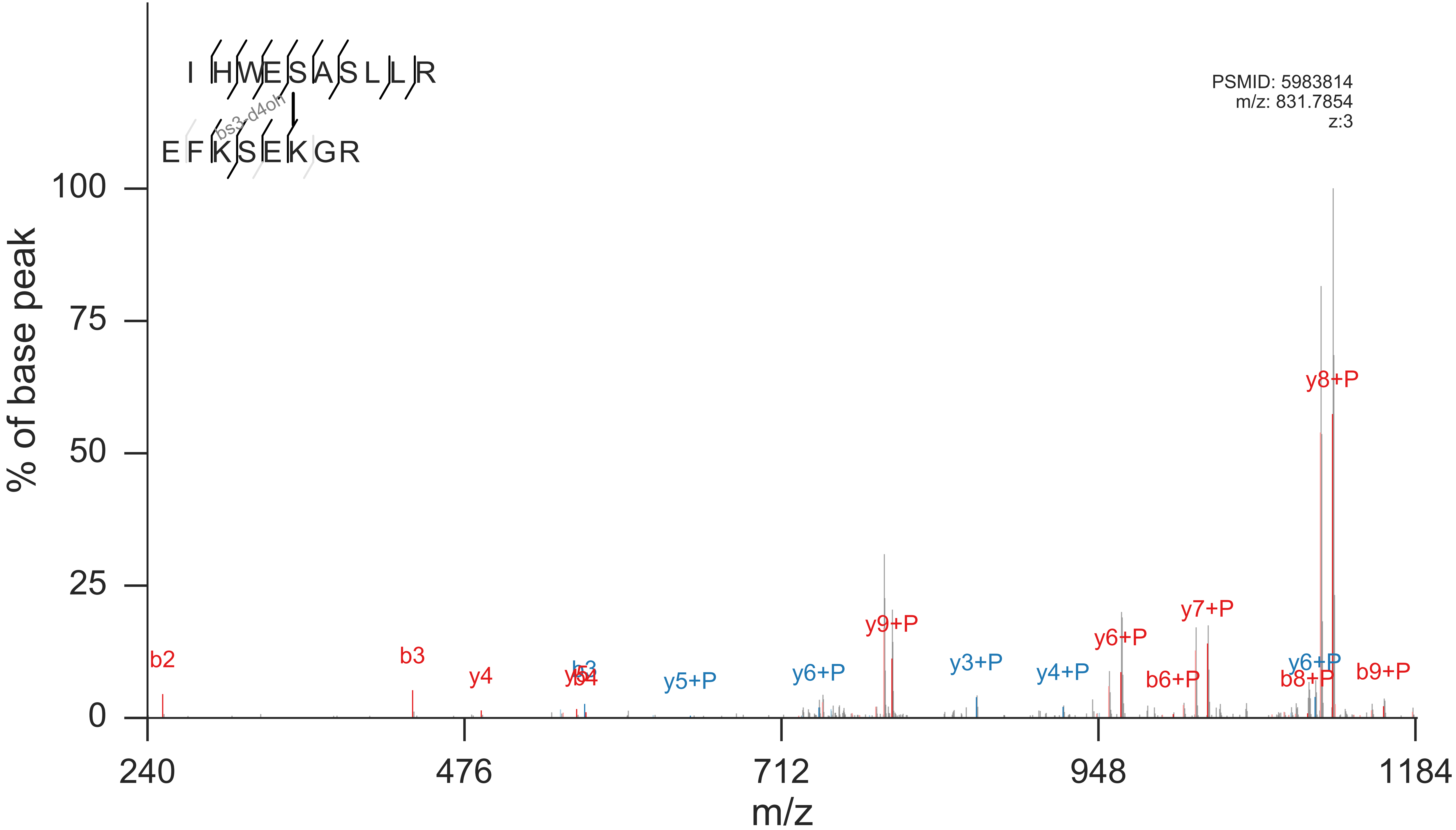

I HWESASLLR
bs3-d4oh
EFKSEKGR
PSMID: 5983814
m/z: 831.7854
z:3
% of base peak
100
75
50
25
0
b2
b3
y4
y5
b4
b3
y5+P
y6+P
y9+P
y3+P
y4+P
y6+P
b6+P
y7+P
y6+P
b8+P
y8+P
b9+P
240
476
712
948
1184
m/z

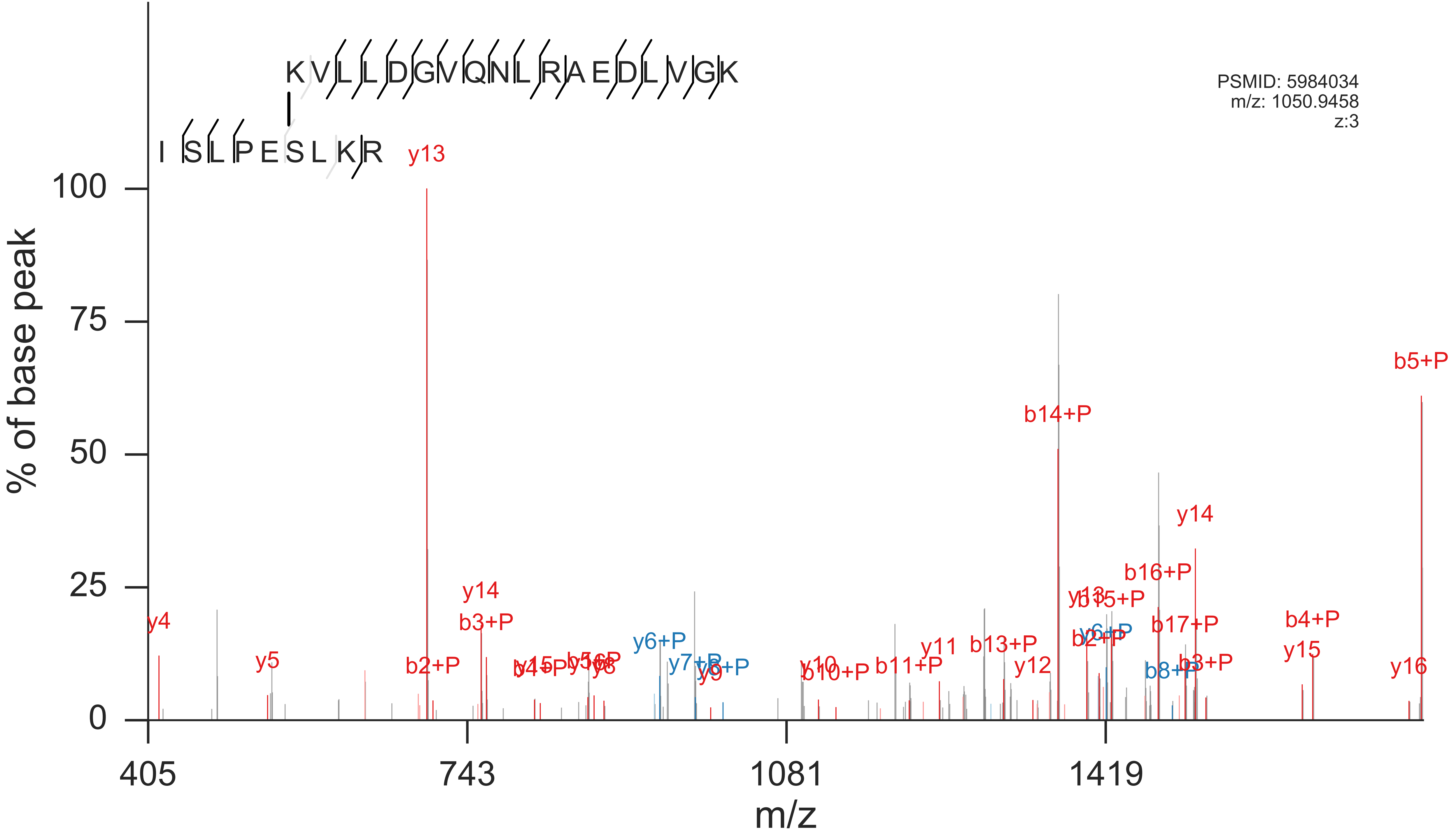

KVLLDGVQNLRAEDLVGK
ISLPESLKR
PSMID: 5984034
m/z: 1050.9458
z:3
% of base peak
100
75
50
25
0
405
743
1081
1419
m/z
y13
b5+P
b14+P
y14
b16+P
b17+P
b4+P
y15
y16
b3+P
y4
y5
b2+P
y6+P
y11
b13+P
y12
b11+P
b8+P

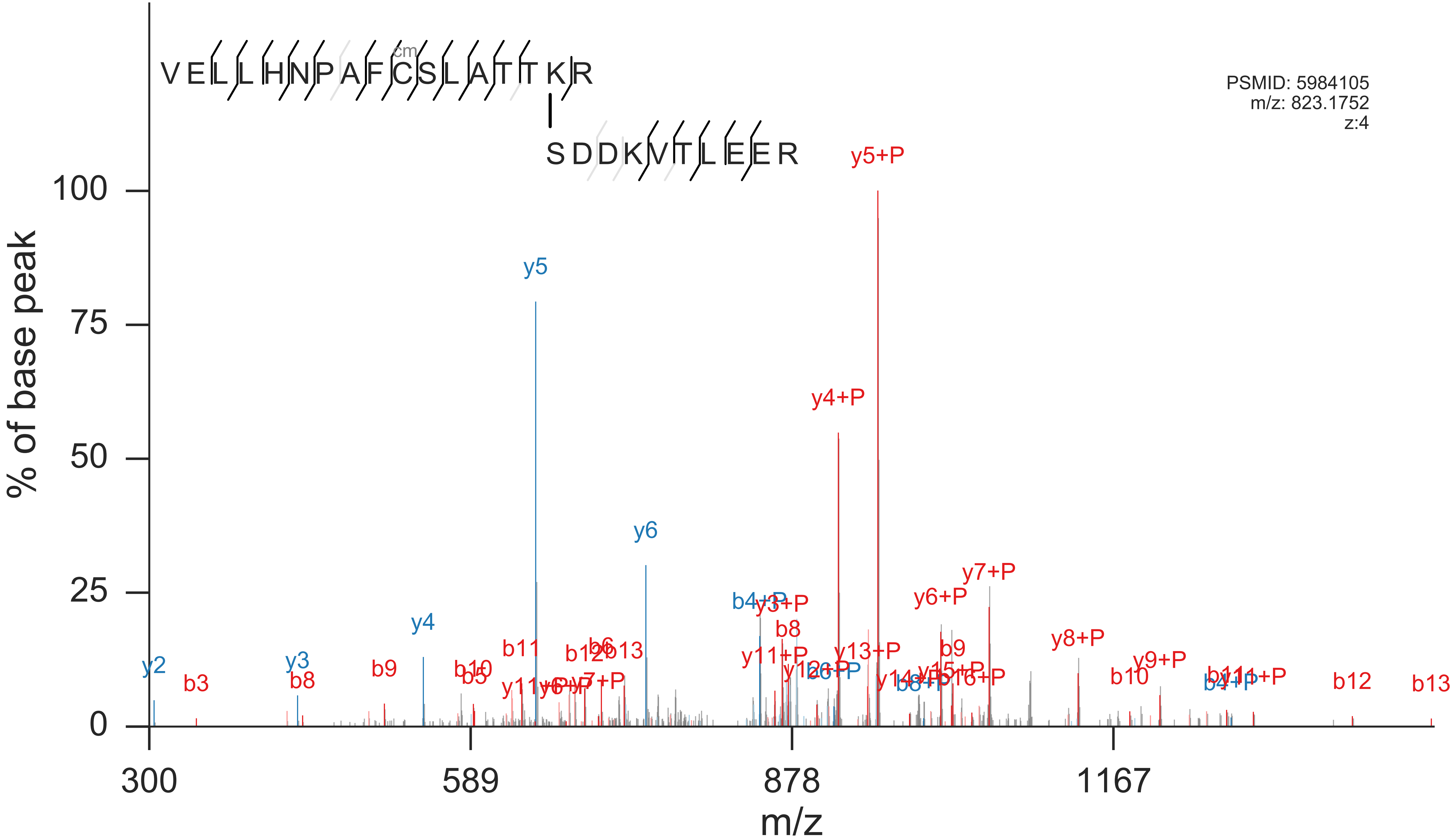

VELLHNPAFCSLATTKR
cm
SDDKVTLEER
PSMID: 5984105
m/z: 823.1752
z:4
% of base peak
m/z
100
75
50
25
0
300
589
878
1167
y5+P
y5
y4+P
y6
y7+P
y6+P
b4+P
y3+P
b8
y4
y8+P
y9+P
b10
b12
b13
y2
b3
y3
b8
b9
b5
b11
b9

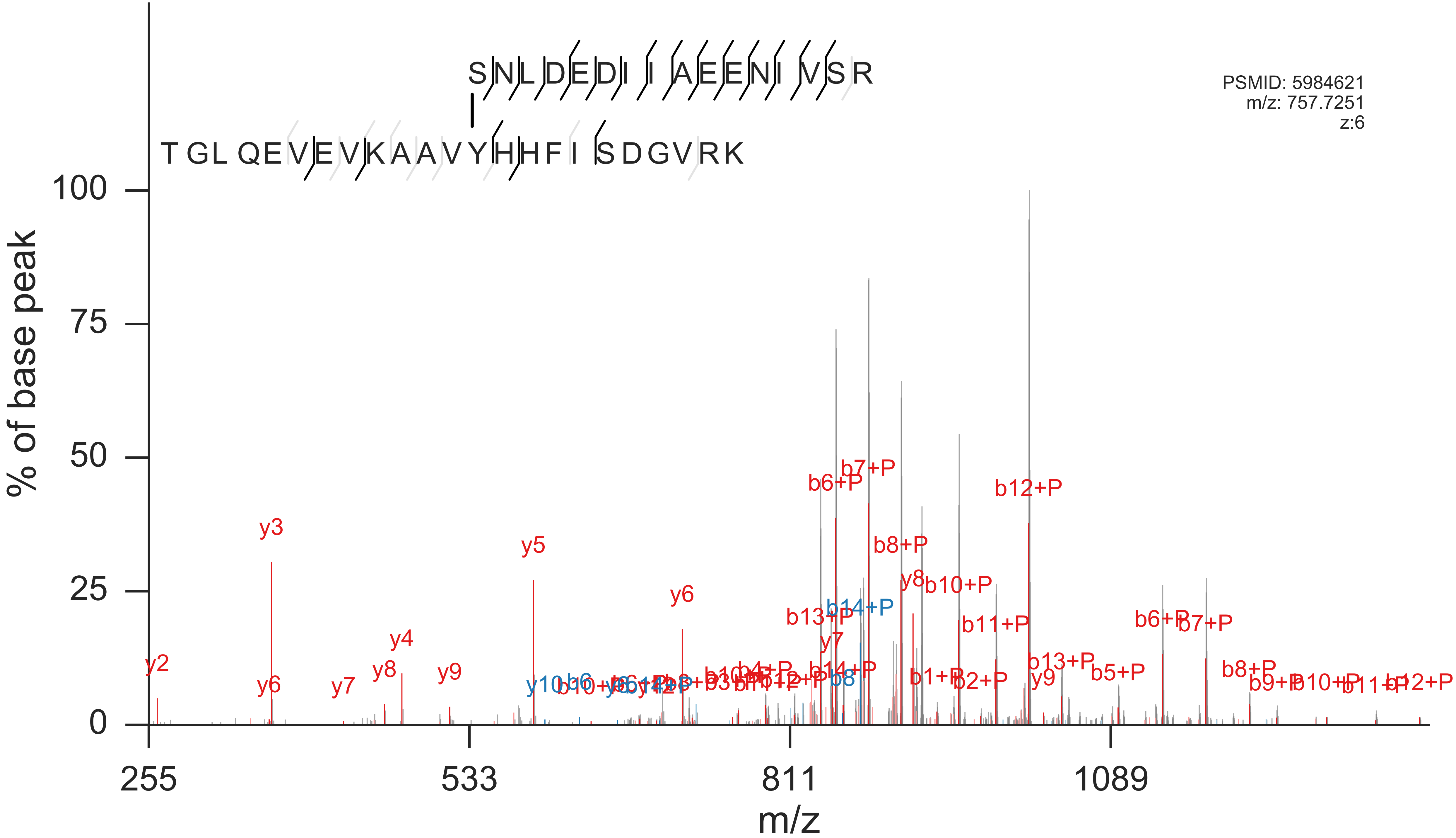
PSMID: 5984621
m/z: 757.7251
z:6
SNLDEDIIAEENIVSR
TGLQEVEVKAAVYHHFISDGVRK
% of base peak
m/z
0
25
50
75
100
255
533
811
1089
y2
y3
y5
y6
y4
y7
y8
y9
b6+P
b7+P
b8+P
b10+P
b11+P
b12+P
b13+P
b14+P
b1+P
b2+P
b5+P
b9+P
y10

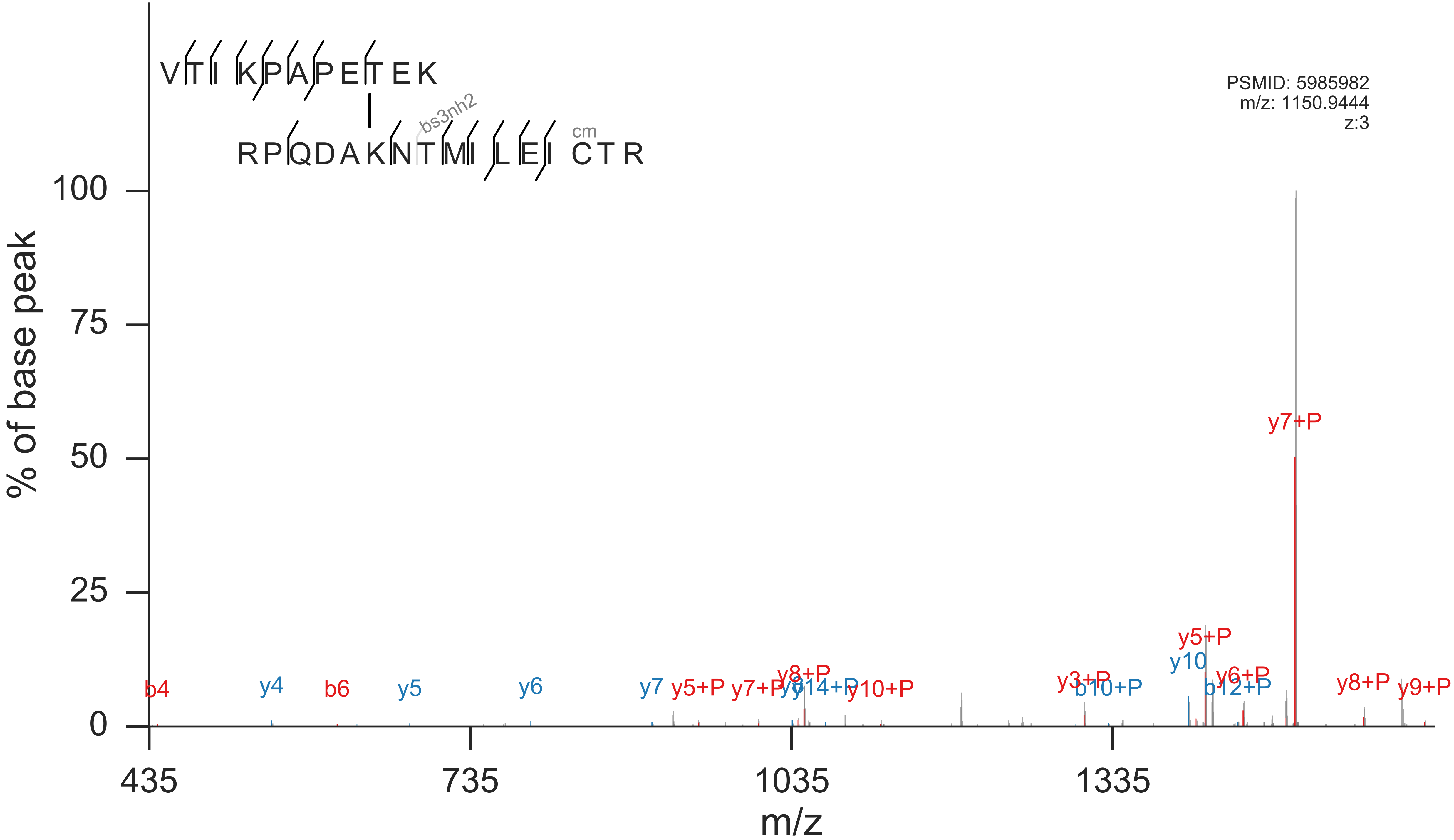

VTIKPAPETEK
RPQDAKNTMILEICTR
bs3nh2
cm
PSMID: 5985982
m/z: 1150.9444
z:3
% of base peak
100
75
50
25
0
435
735
1035
1335
m/z
y7+P
y5+P
y10
y6+P
b12+P
y3+P
b10+P
y8+P
y9+P
b4
y4
b6
y5
y6
y7
y5+P
y7+P
y10+P

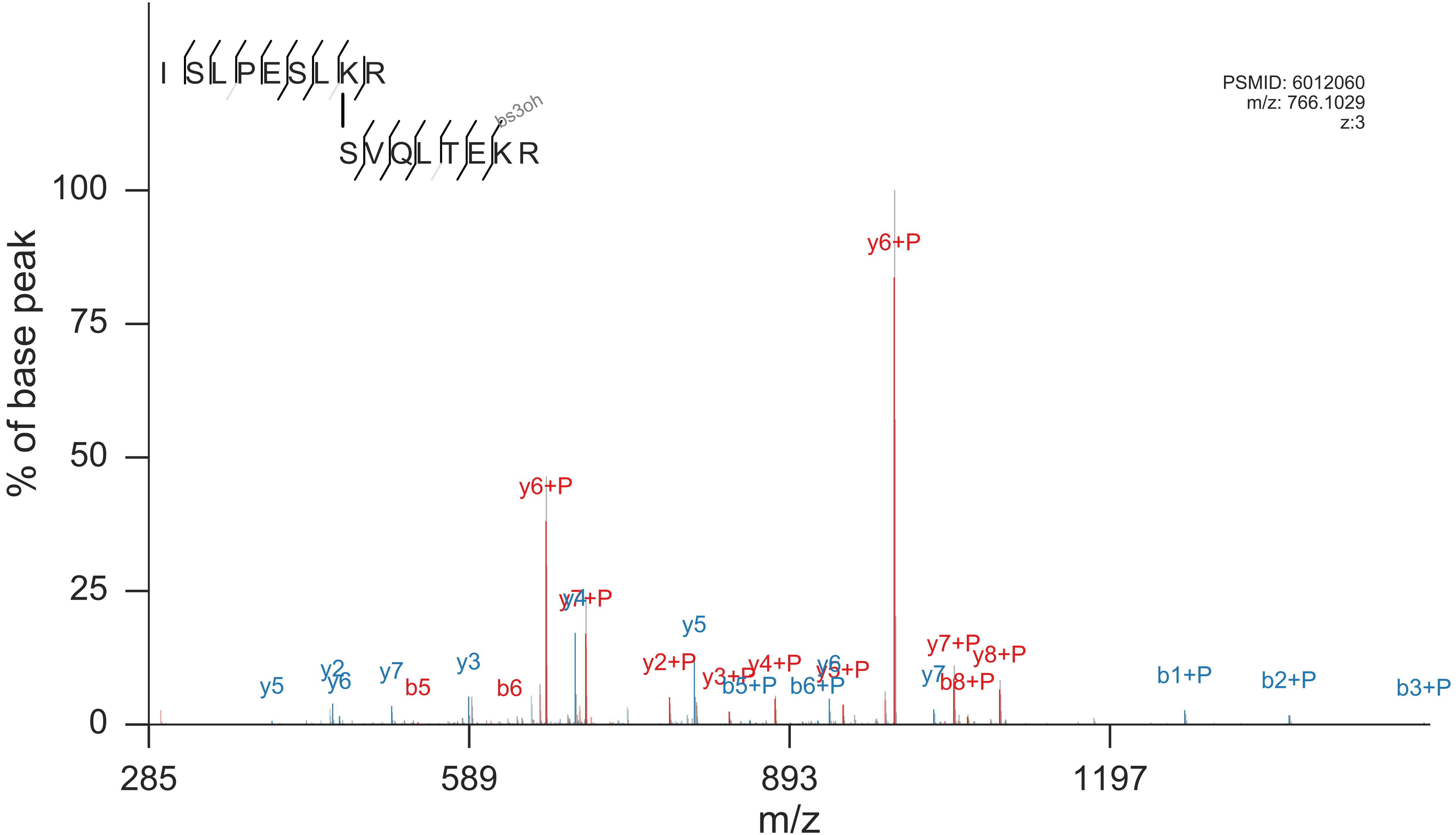

I SLPESLKR
SVQLTEKR
bs3oh
PSMID: 6012060
m/z: 766.1029
z:3
% of base peak
m/z
100
75
50
25
0
285
589
893
1197
y6+P
y6+P
y7
y4+P
y5
y2+P
y3+P
y4+P
y6
y5+P
b5+P
b6+P
y7+P
y8+P
y7
b8+P
b1+P
b2+P
b3+P
y5
y2
y6
y7
b5
y3
b6

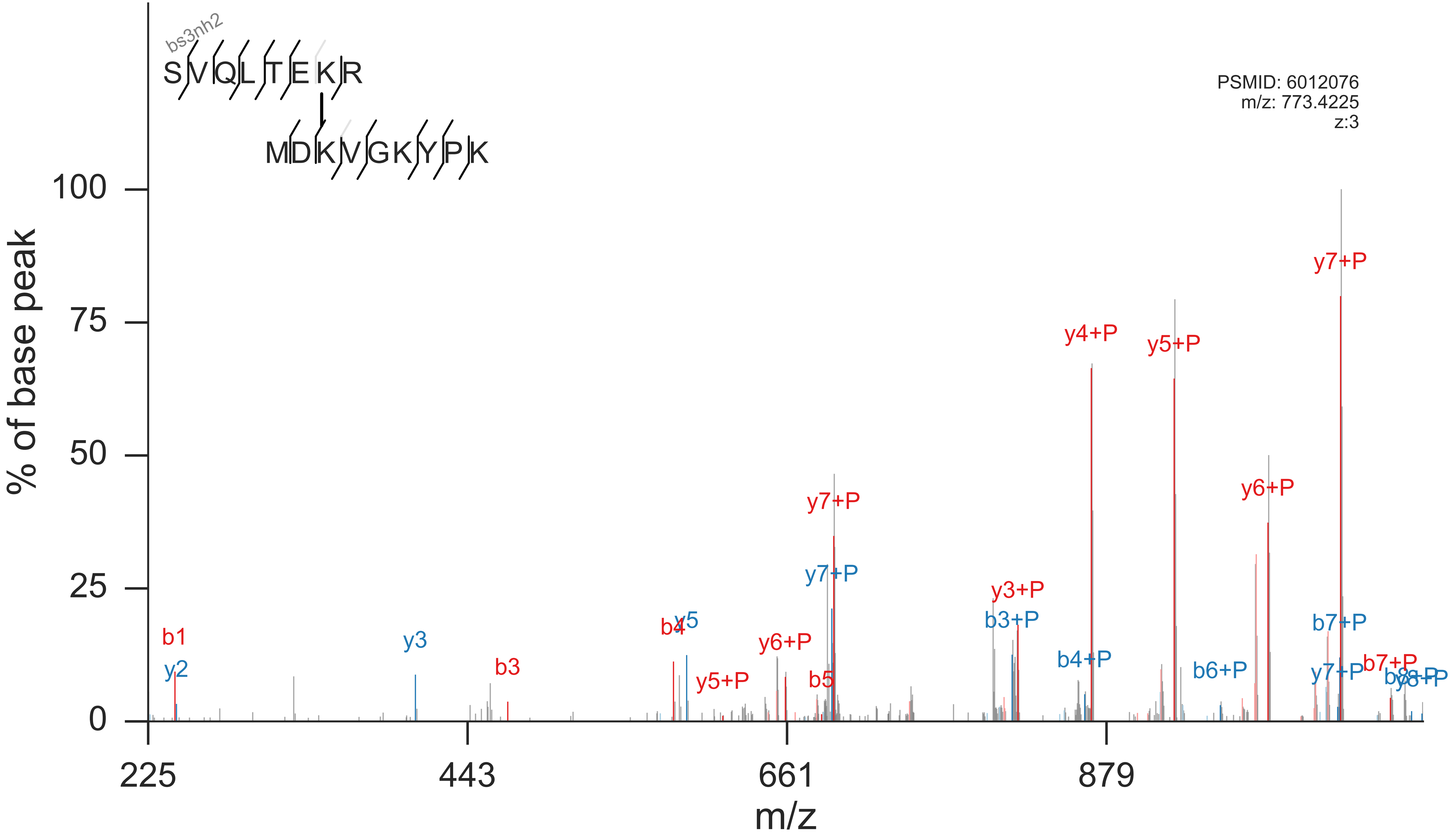

bs3nh2
SVQLTEKR
MDKVGKYPK
PSMID: 6012076
m/z: 773.4225
z:3
% of base peak
100
75
50
25
0
225
443
661
879
m/z
y7+P
y4+P
y5+P
y6+P
y7+P
y7+P
y3+P
b3+P
b7+P
b1
y2
y3
b3
b4
y5
y5+P
y6+P
b5
b4+P
b6+P
y7+P
b7+P

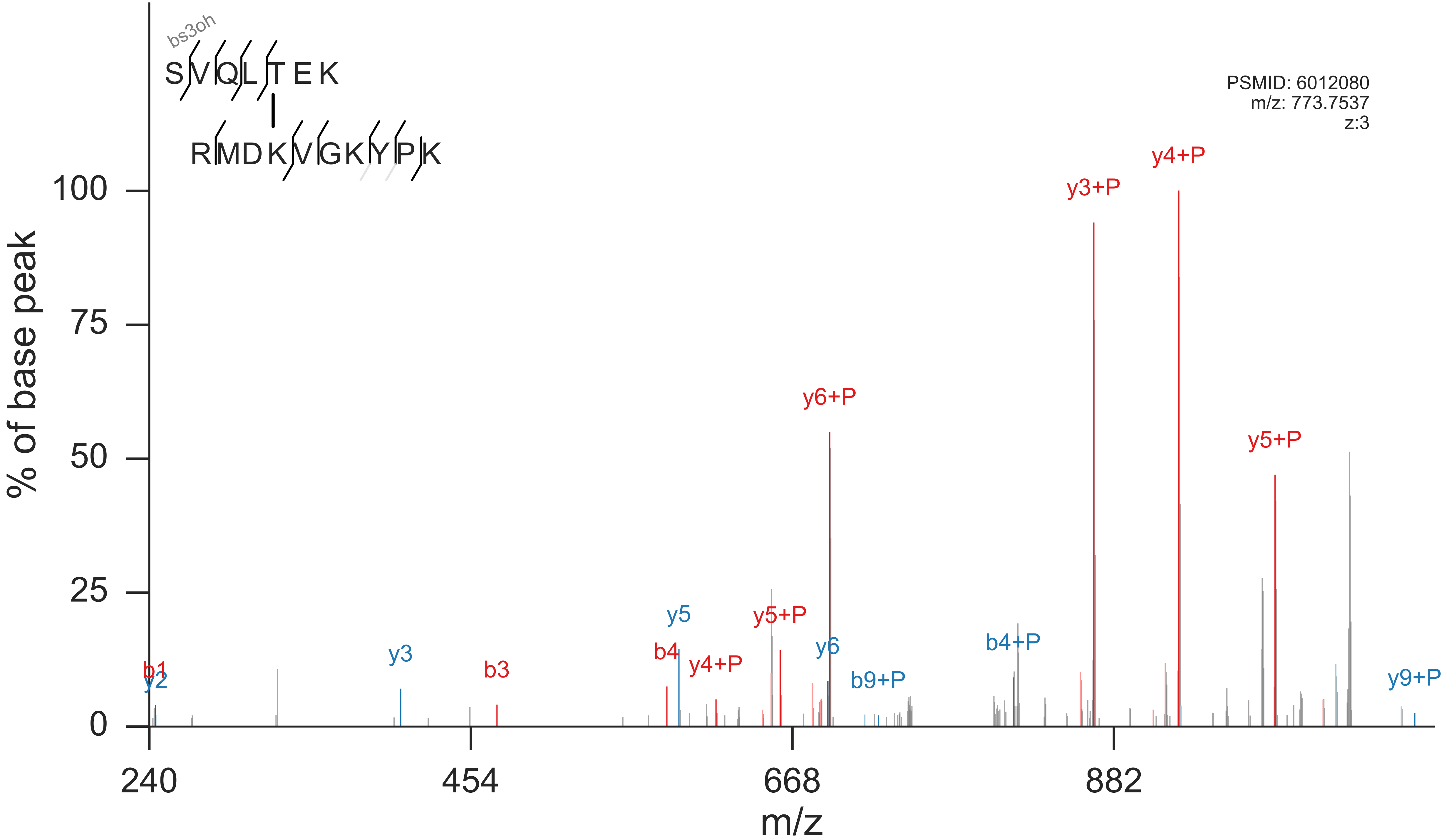
bs3oh
SVQLTEK
RMDKVGKYPK
PSMID: 6012080
m/z: 773.7537
z:3
% of base peak
100
75
50
25
0
240
454
668
882
m/z
b1
y2
y3
b3
b4
y5
y4+P
y5+P
y6
y6+P
b9+P
b4+P
y3+P
y4+P
y5+P
y9+P

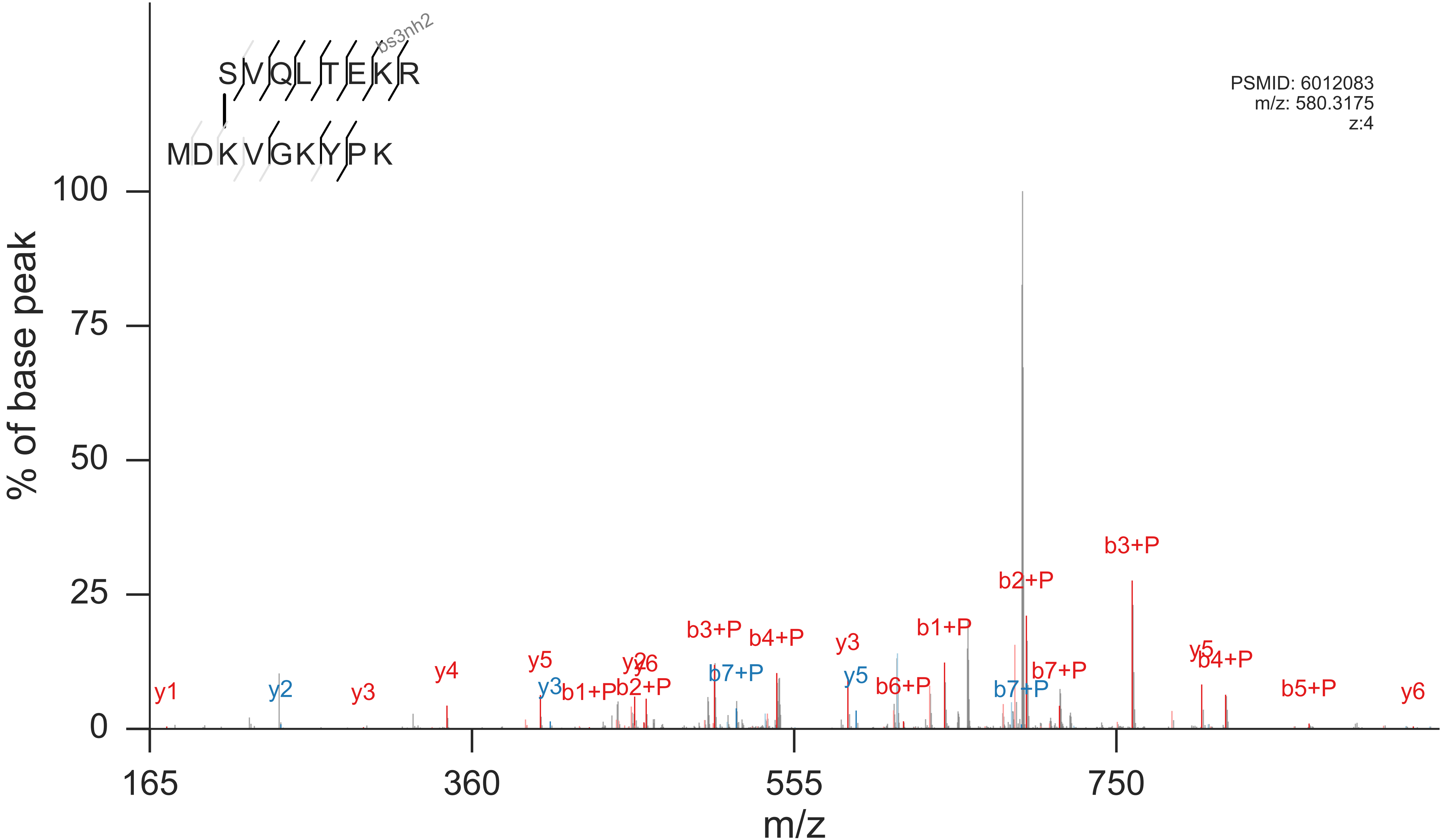

SVQLTEKR
MDKVGKYPK
bs3nh2
PSMID: 6012083
m/z: 580.3175
z:4
% of base peak
m/z
0
25
50
75
100
165
360
555
750
y1
y2
y3
y4
y5
y3
b1+P
y2
y6
b2+P
b3+P
b7+P
b4+P
y3
y5
b6+P
b1+P
b2+P
b7+P
b7+P
b3+P
y5
b4+P
b5+P
y6

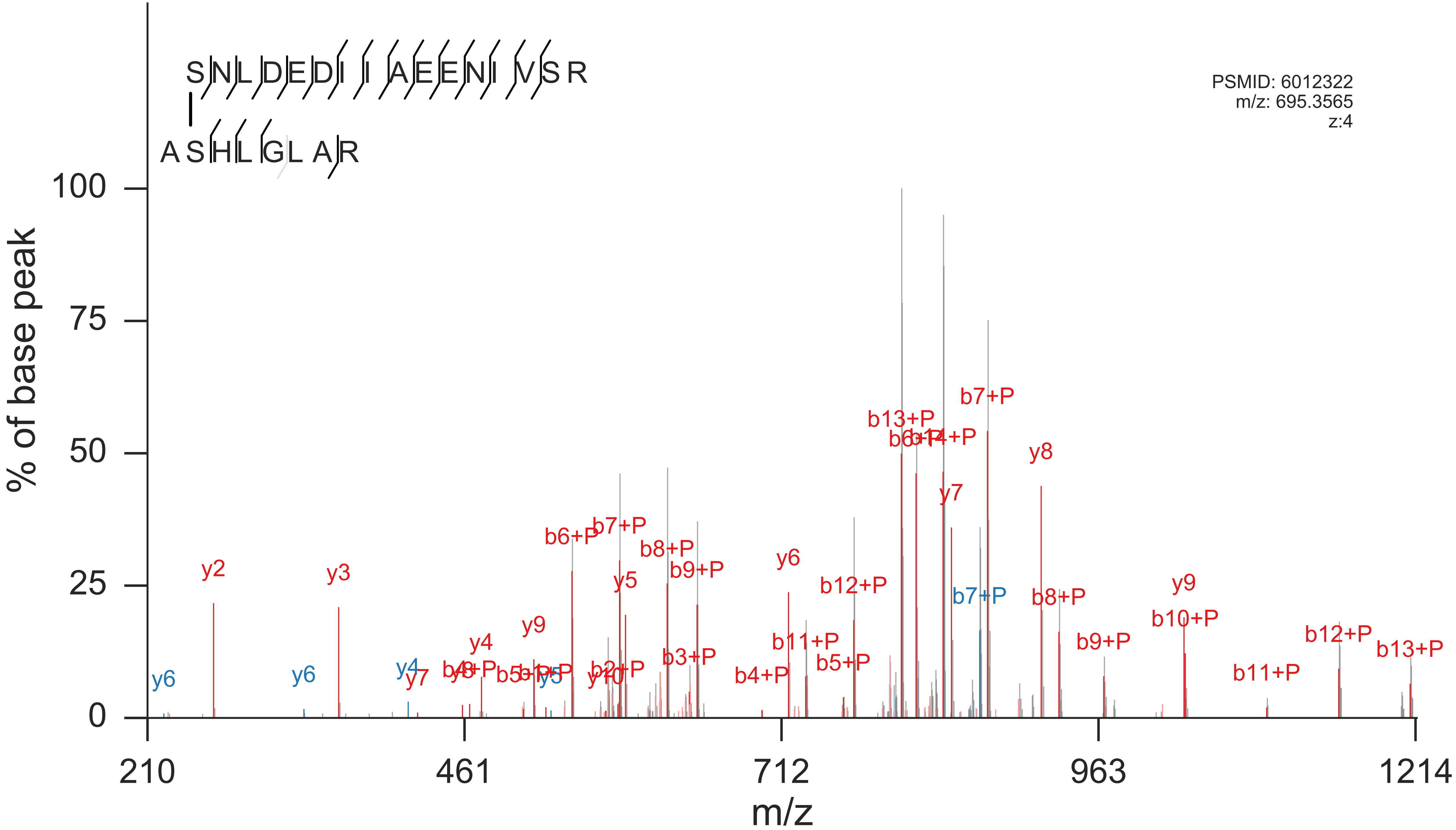

SNLDEDIIAEENIVSR
ASHLGLAR
PSMID: 6012322
m/z: 695.3565
z:4
% of base peak
m/z

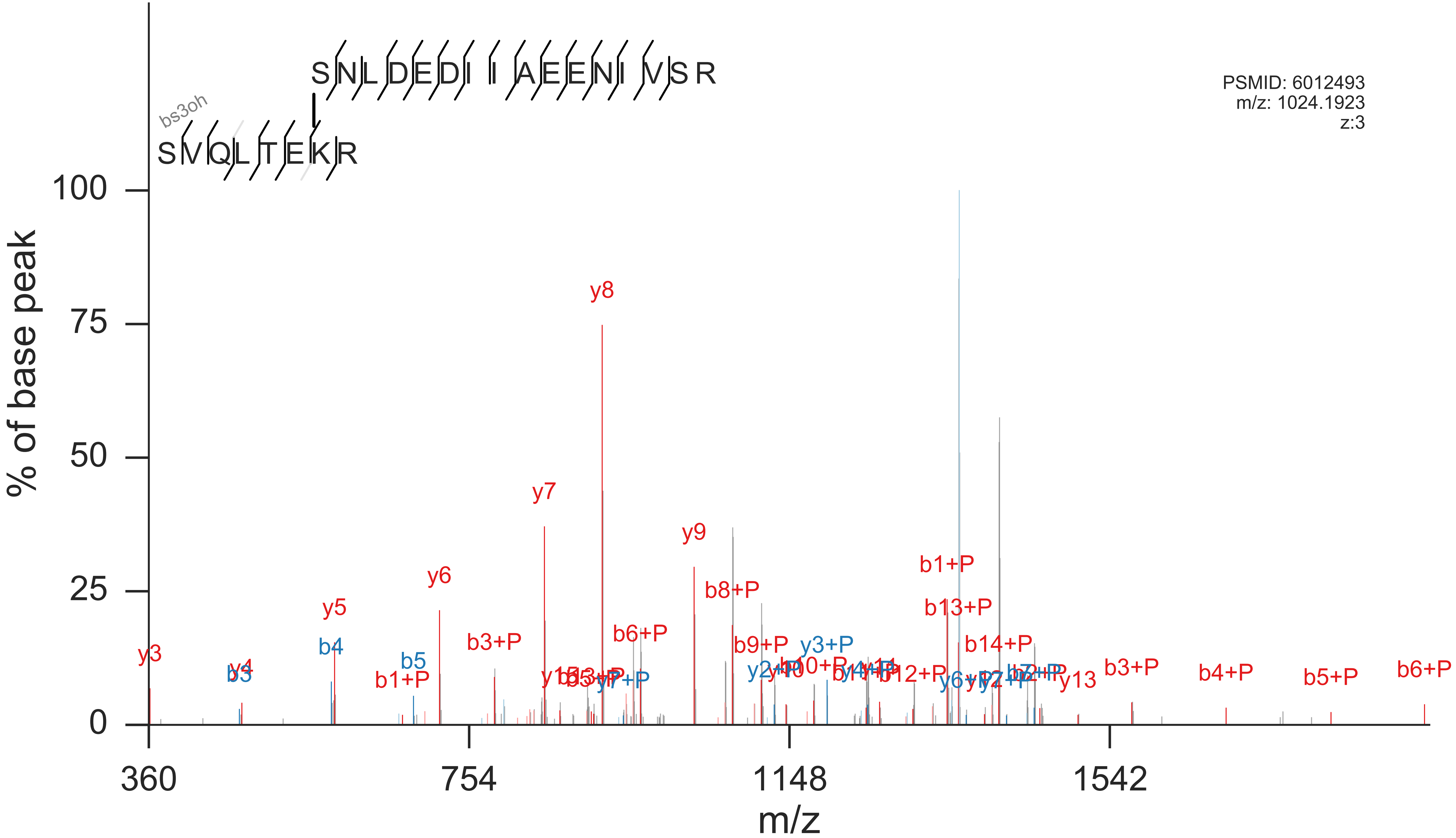

SNLDEDIIAEENIVSR
SVQLTEKR
bs3oh
PSMID: 6012493
m/z: 1024.1923
z:3
% of base peak
m/z
y3
y4
b3
y5
b4
b5
b1+P
y6
b3+P
y7
y8
b6+P
y9
b8+P
b9+P
y3+P
b1+P
b13+P
b14+P
y13
b3+P
b4+P
b5+P
b6+P
100
75
50
25
0
360
754
1148
1542

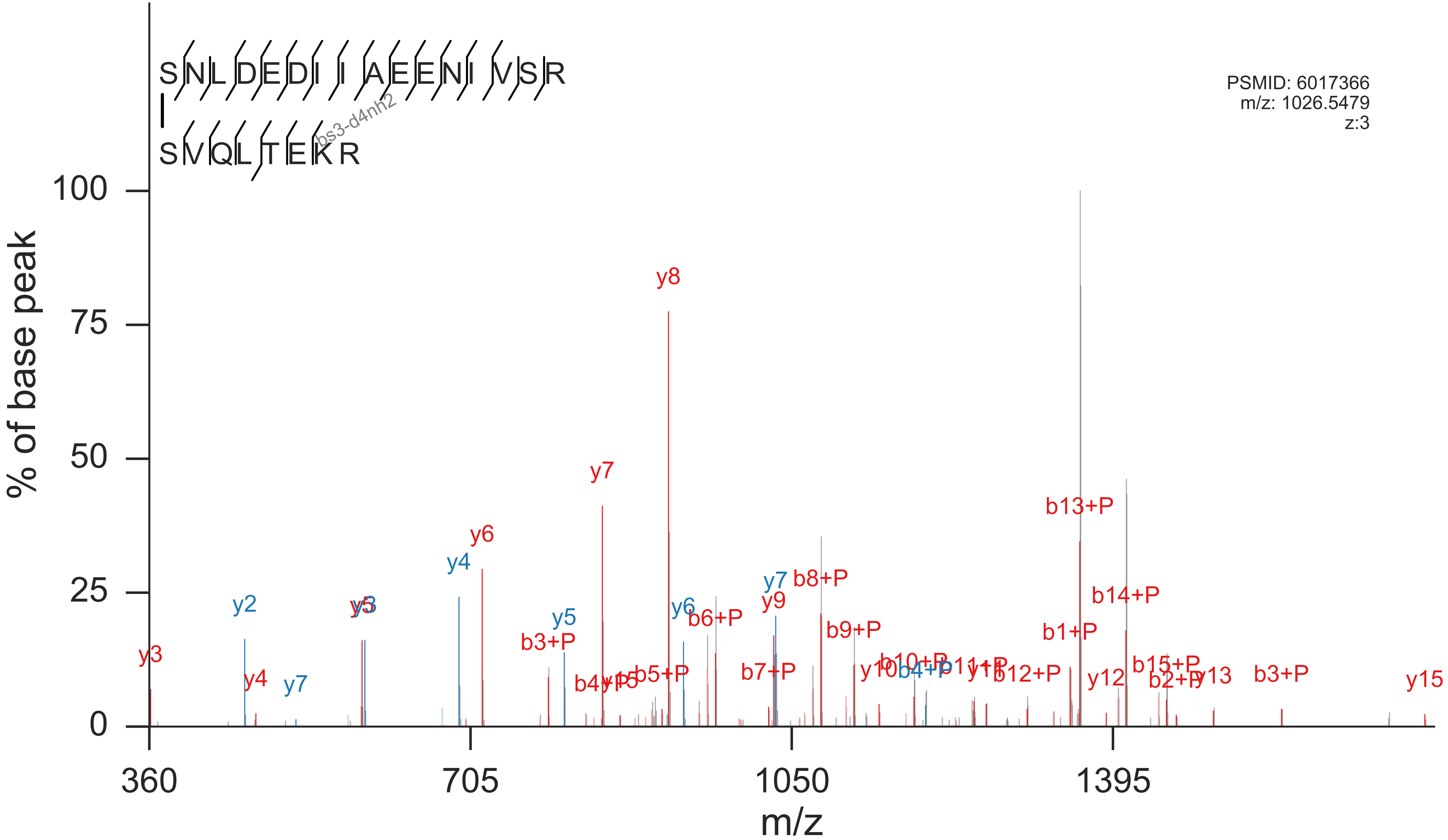
SNLDEDIIAEENIVSR
SVQLTEKR
bs3-d4nh2
PSMID: 6017366
m/z: 1026.5479
z:3
% of base peak
m/z
y8
y7
y6
y4
y2
y3
y5
y6
y7
y9
b3+P
b6+P
b8+P
b9+P
b13+P
b14+P
b1+P
b7+P
b5+P
b12+P
y12
b15+P
y13
b3+P
y15

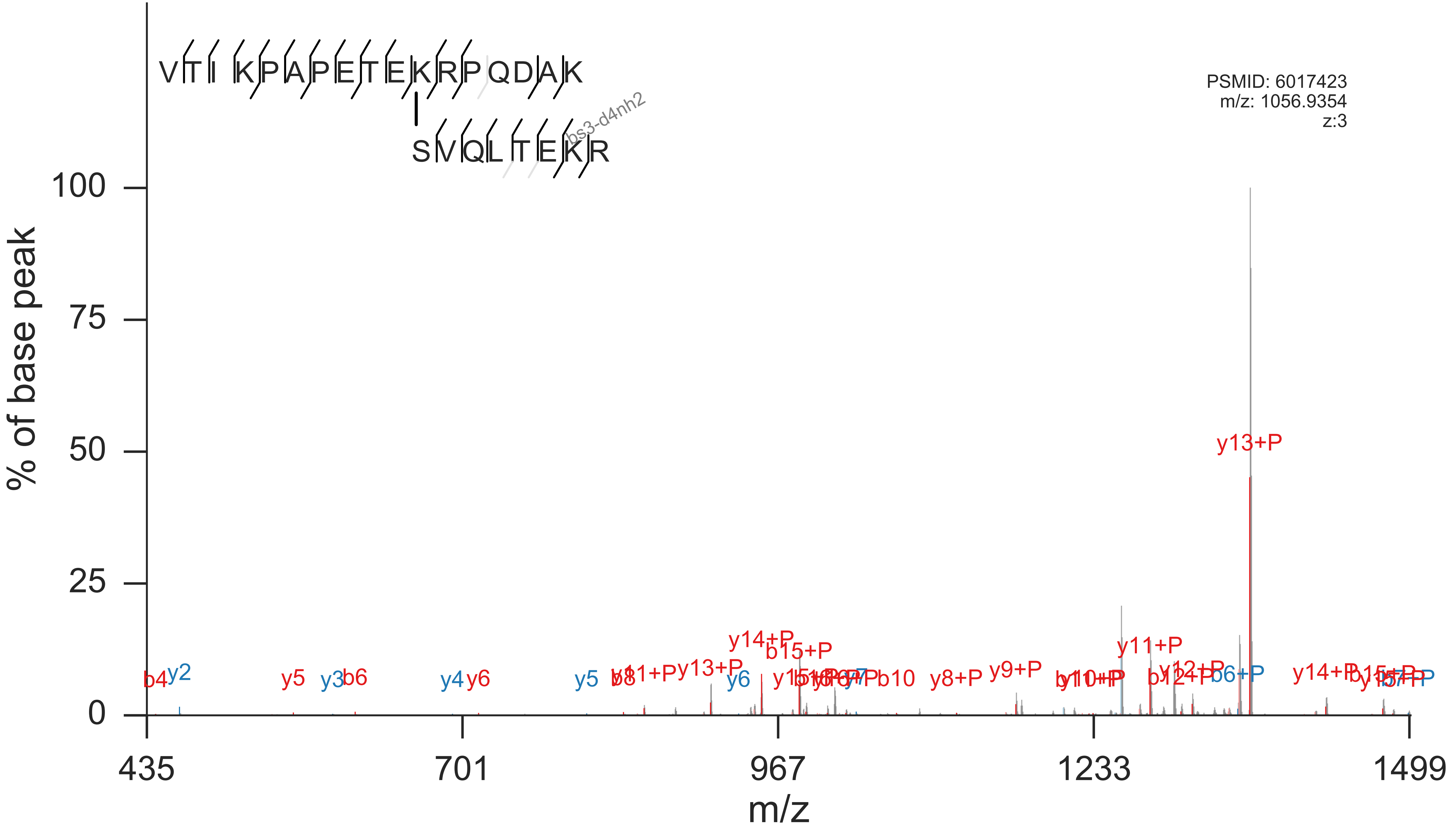

VTIKPAPETEKRPQDAK
SVQLTEKR
bs3-d4nh2
PSMID: 6017423
m/z: 1056.9354
z:3
% of base peak
m/z
y13+P
y11+P
y14+P
b15+P
y9+P
y8+P
b10
b4
y2
y5
y3
b6
y4
y6
y5
y6

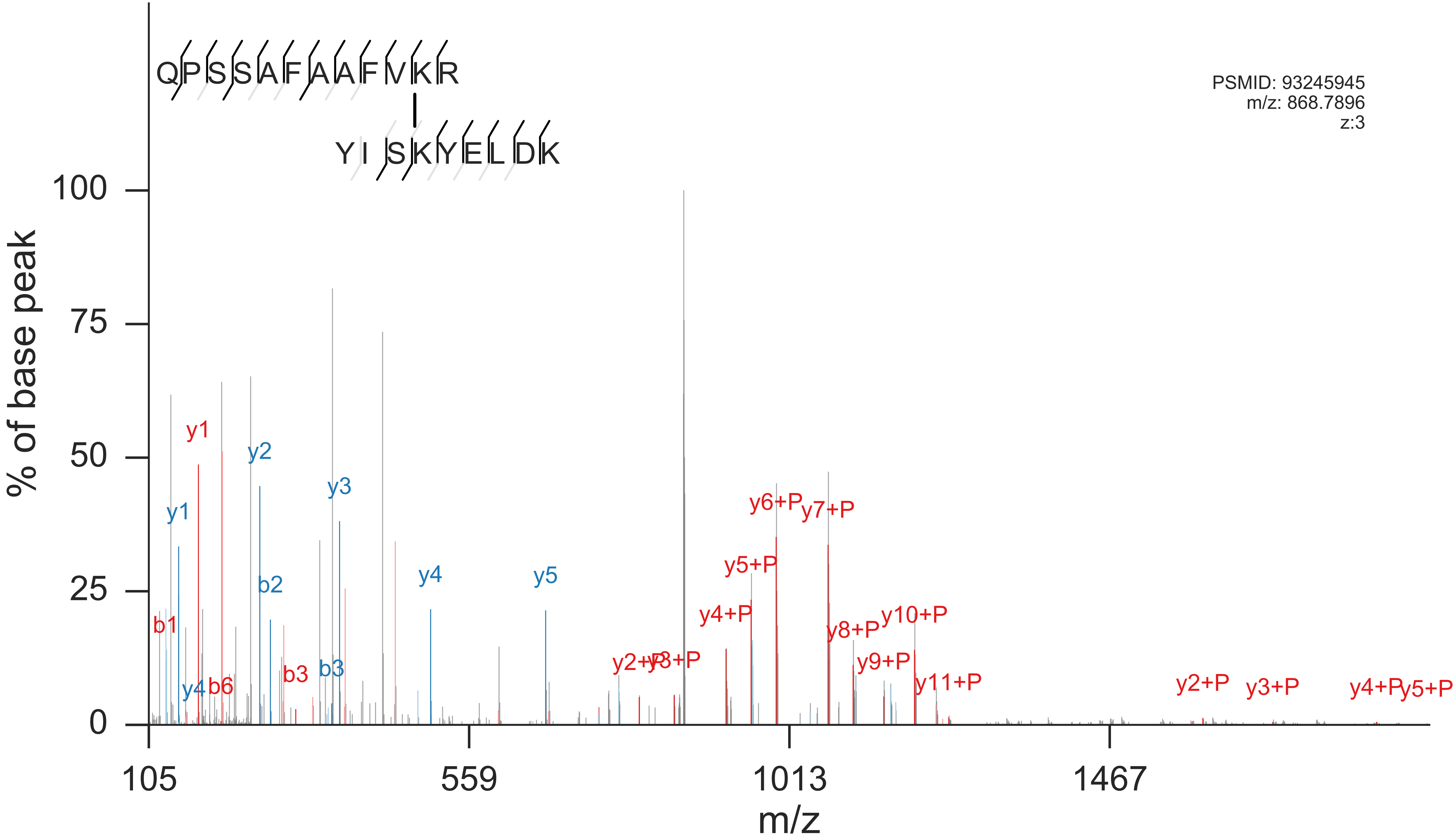

QPSSAFAAFVKR
YISKYELDK
PSMID: 93245945
m/z: 868.7896
z:3
% of base peak
100
75
50
25
0
105
559
1013
1467
m/z
y1
y2
y3
y1
y4
y5
b2
b1
y4
b6
b3
b3
y6+P
y7+P
y5+P
y4+P
y10+P
y8+P
y9+P
y2+P
y3+P
y11+P
y2+P
y3+P
y4+P
y5+P

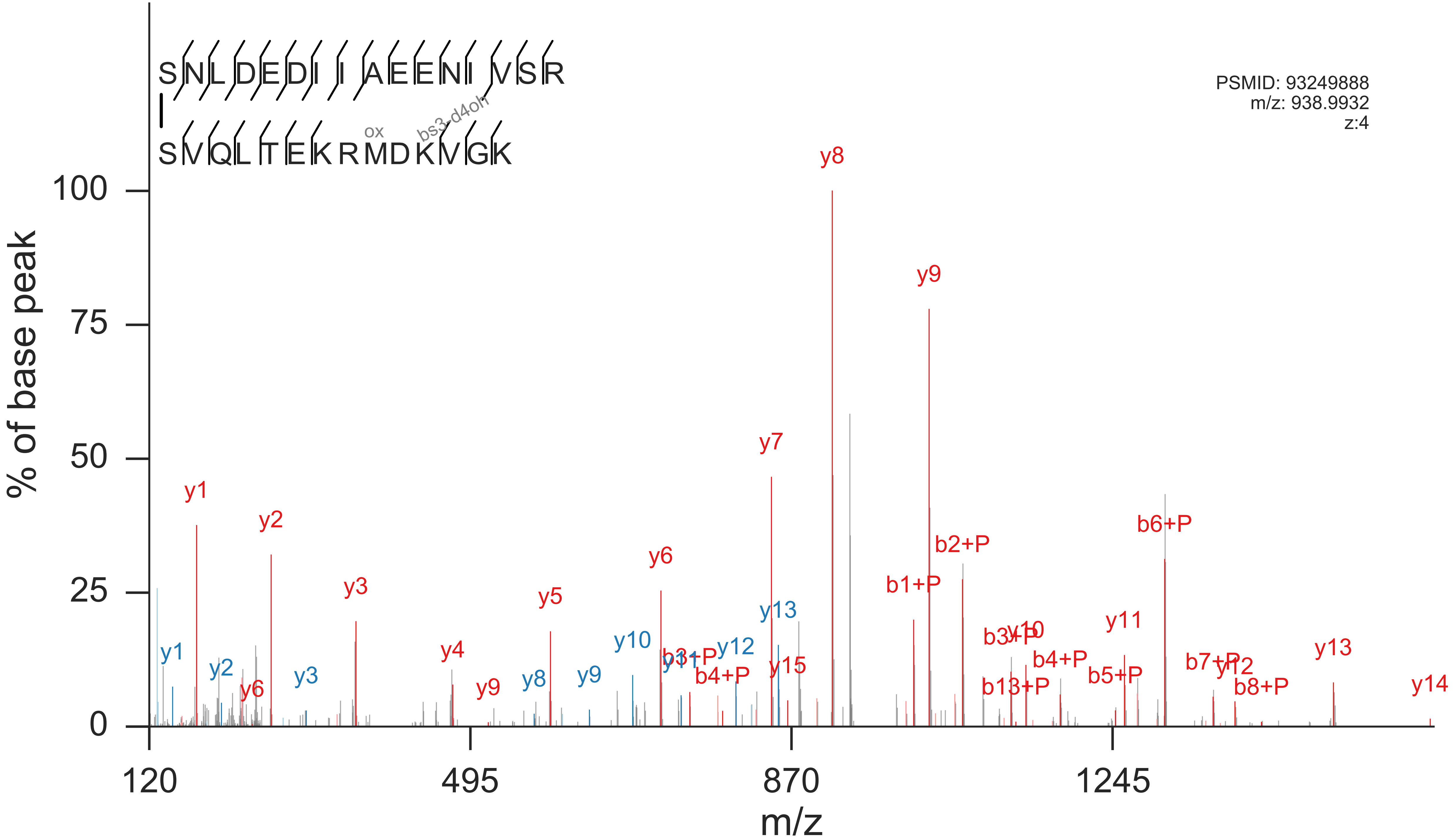

SNLDEDIIAEENIVSR
SVQLTEKRMDKVGK
ox
bs3-d4oh
PSMID: 93249888
m/z: 938.9932
z:4
% of base peak
m/z

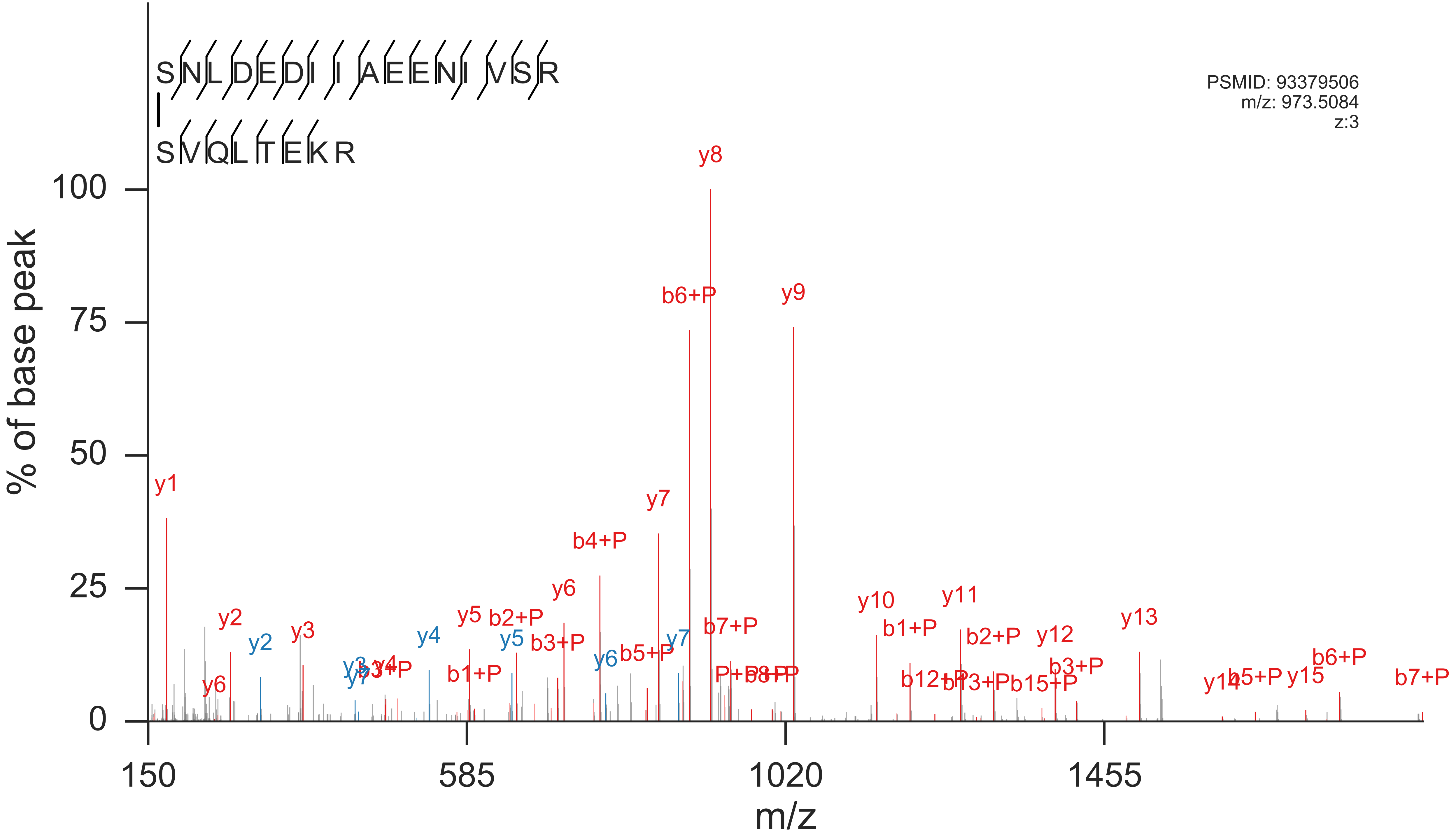

SNLDEDIIAEENIVSR
SVQLTEKR
PSMID: 93379506
m/z: 973.5084
z:3
% of base peak
100
75
50
25
0
150
585
1020
1455
m/z
y1
y2
y3
y6
y2
y4
y5
y5
b2+P
b3+P
b1+P
y6
b4+P
y6
y7
b5+P
y7
b6+P
y8
y9
b7+P
y10
b1+P
y11
b2+P
y12
b3+P
y13
b6+P
y15
b7+P
b15+P

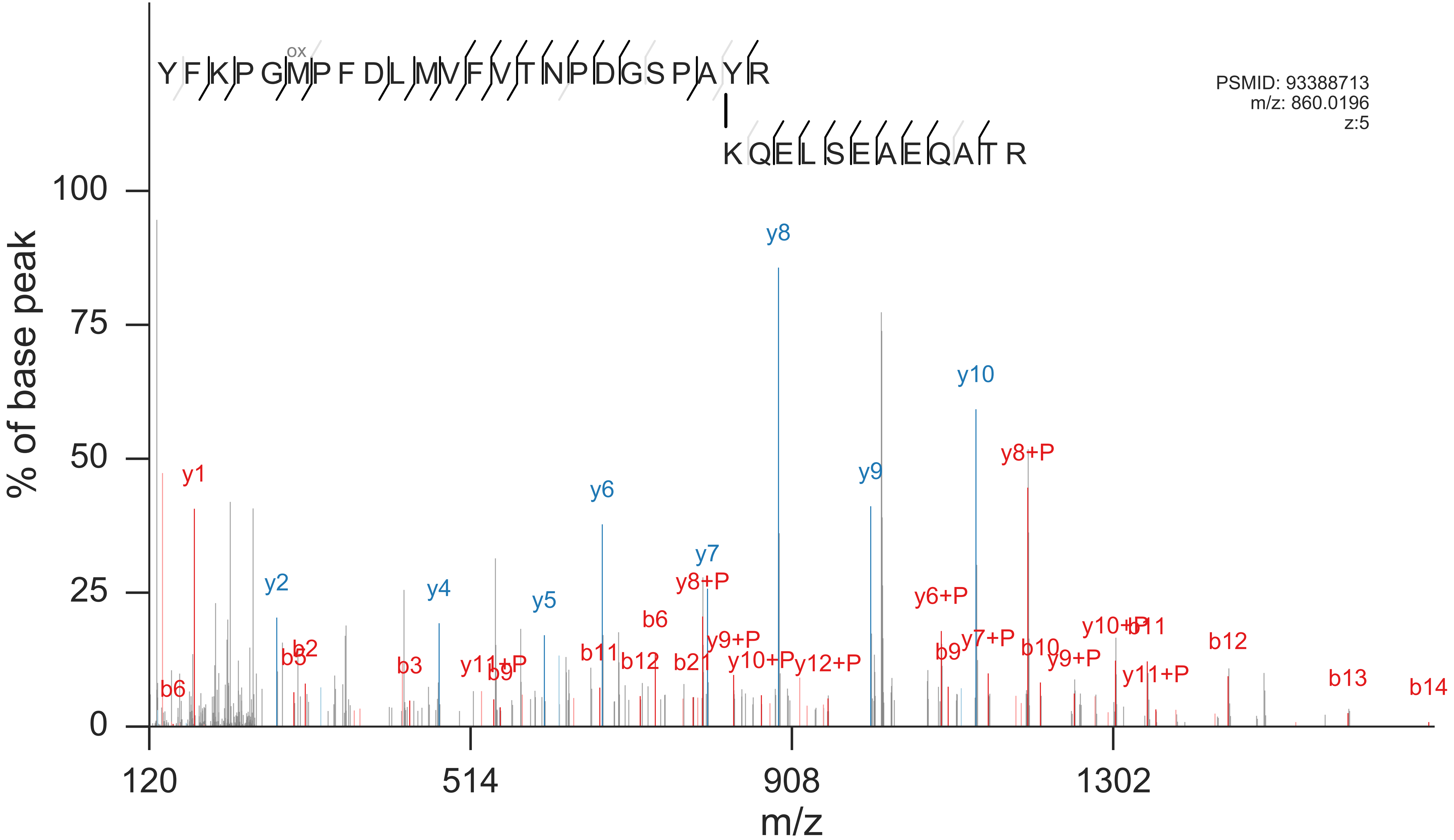

YFKPGMPFDLMVFVTNPDGSPAYR
KQELSEAEQATR
ox
PSMID: 93388713
m/z: 860.0196
z:5
% of base peak
m/z
100
75
50
25
0
120
514
908
1302
y1
y2
y4
y5
y6
y7
y8
y9
y10
y8+P
y6+P
y7+P
y9+P
y10+P
y11+P
y12+P
b2
b3
b5
b6
b9
b10
b11
b12
b13
b14
b21

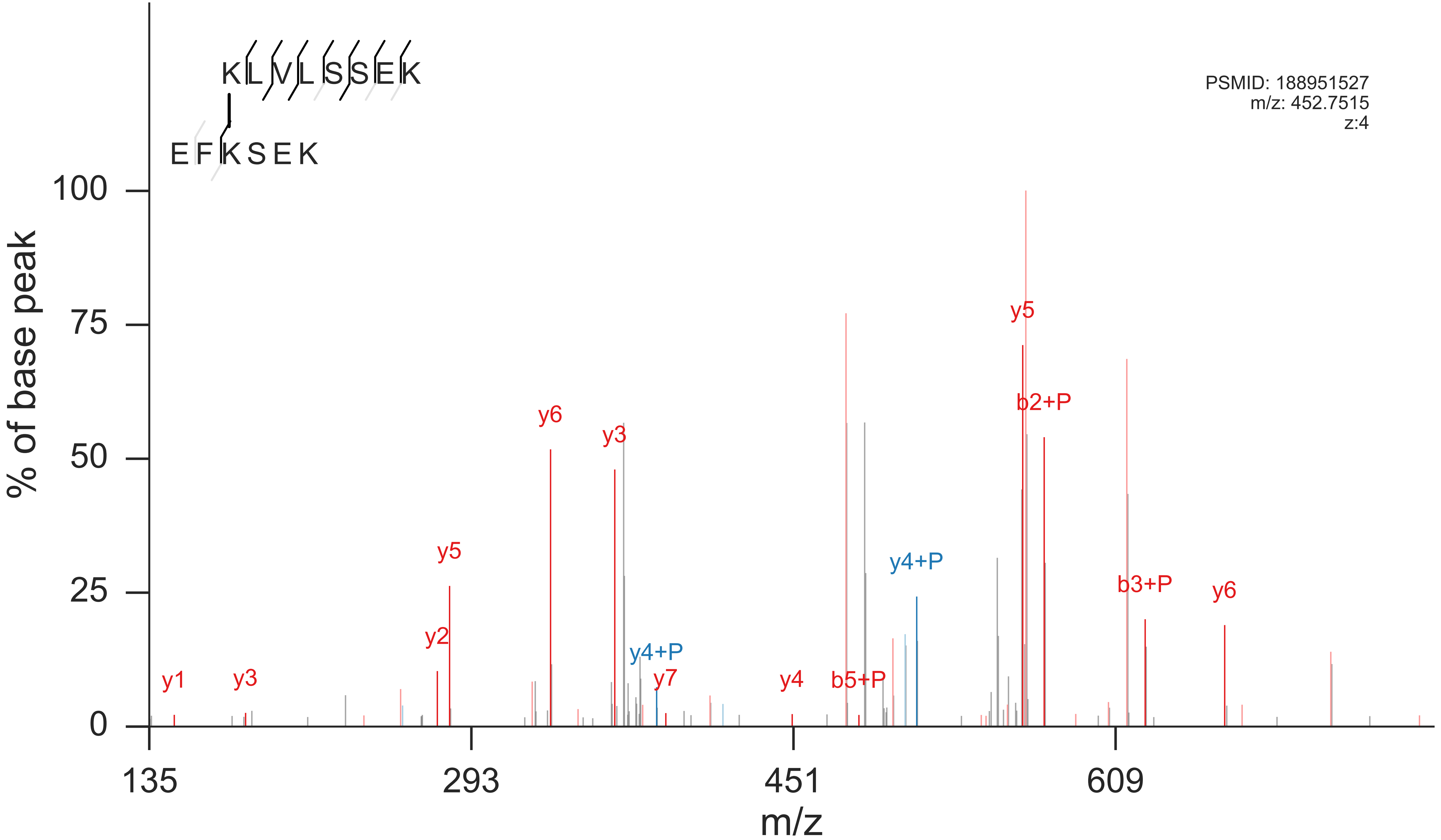

KLVLSSEK
EFKSEK
PSMID: 188951527
m/z: 452.7515
z:4
% of base peak
m/z
y1
y3
y2
y5
y6
y3
y4+P
y7
y4
b5+P
y4+P
y5
b2+P
b3+P
y6

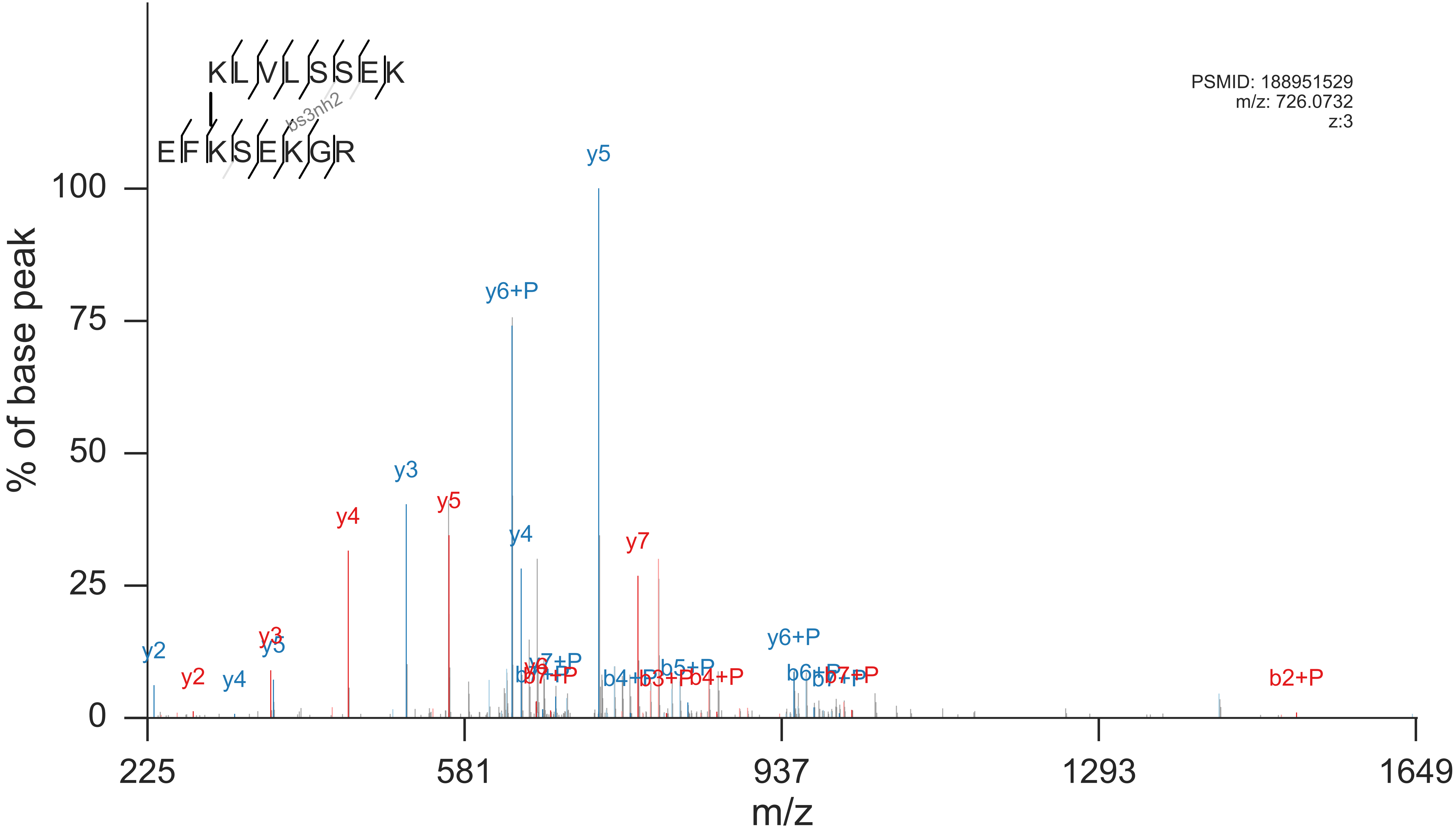

KLVLSSEK
EFKSEKGR
bs3nh2
PSMID: 188951529
m/z: 726.0732
z:3
% of base peak
m/z
0
25
50
75
100
225
581
937
1293
1649

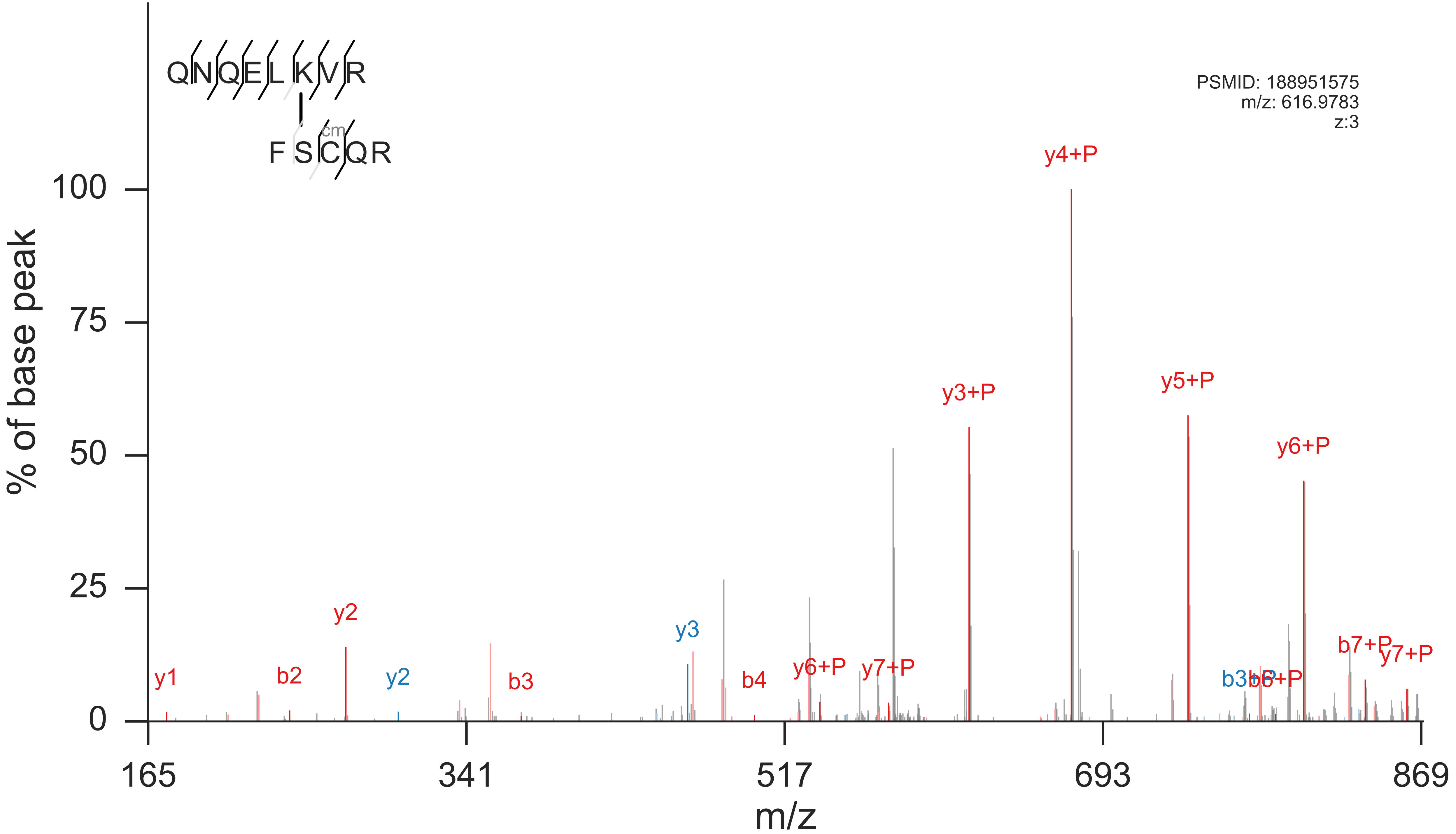
QNQELKVR
FSCQR
cm
PSMID: 188951575
m/z: 616.9783
z:3
% of base peak
m/z
y1
b2
y2
y2
b3
y3
b4
y6+P
y7+P
y3+P
y4+P
y5+P
y6+P
b3+P
b6+P
b7+P
y7+P
100
75
50
25
0
165
341
517
693
869

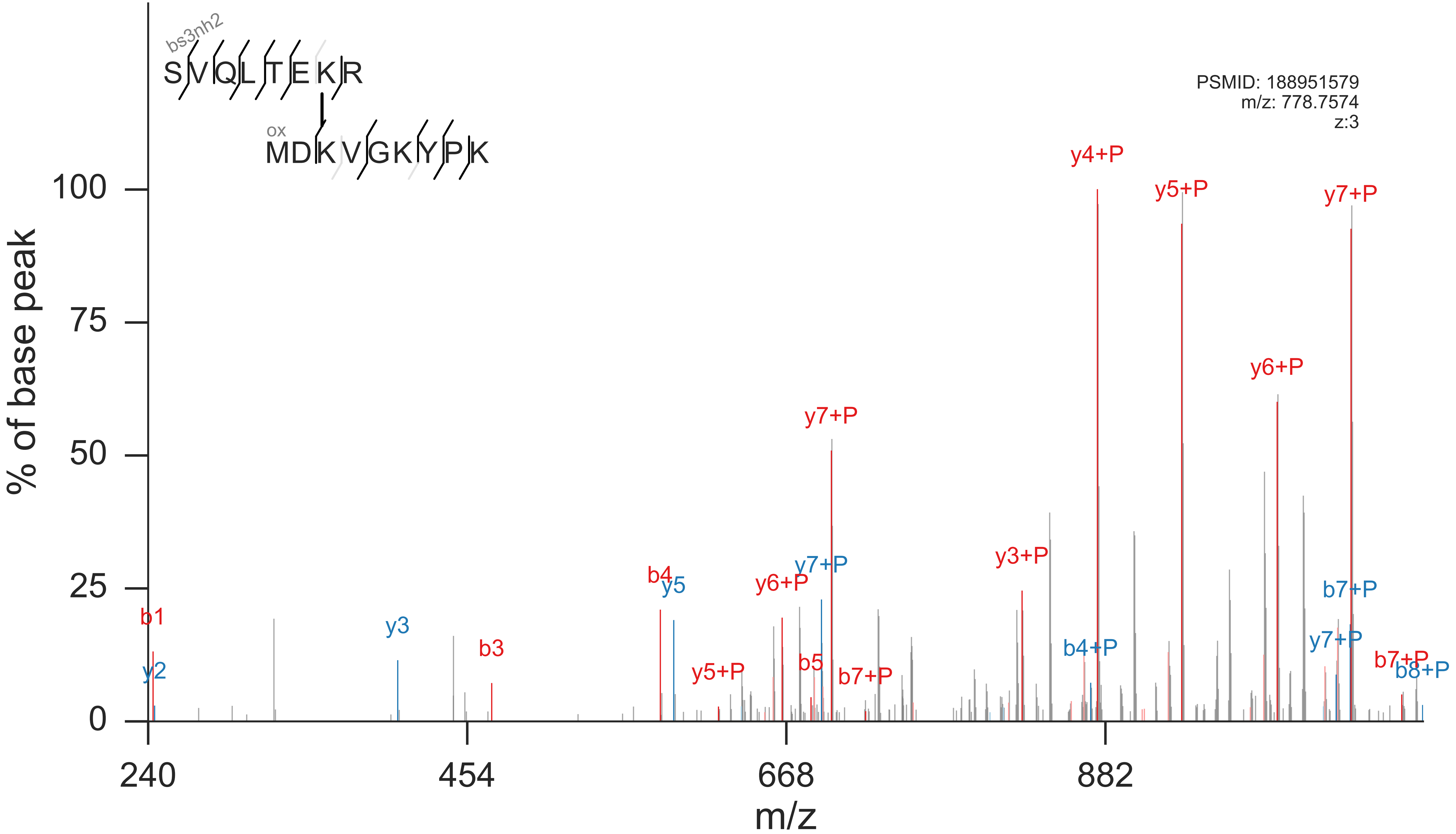
bs3nh2
SVQLTEKR
ox
MDKVGKYPK
PSMID: 188951579
m/z: 778.7574
z:3
% of base peak
m/z
y4+P
y5+P
y7+P
y6+P
y7+P
y3+P
y7+P
b4
y5
y6+P
b7+P
y7+P
b4+P
b7+P
b8+P
b1
y3
b3
y2
y5+P
b5
b7+P

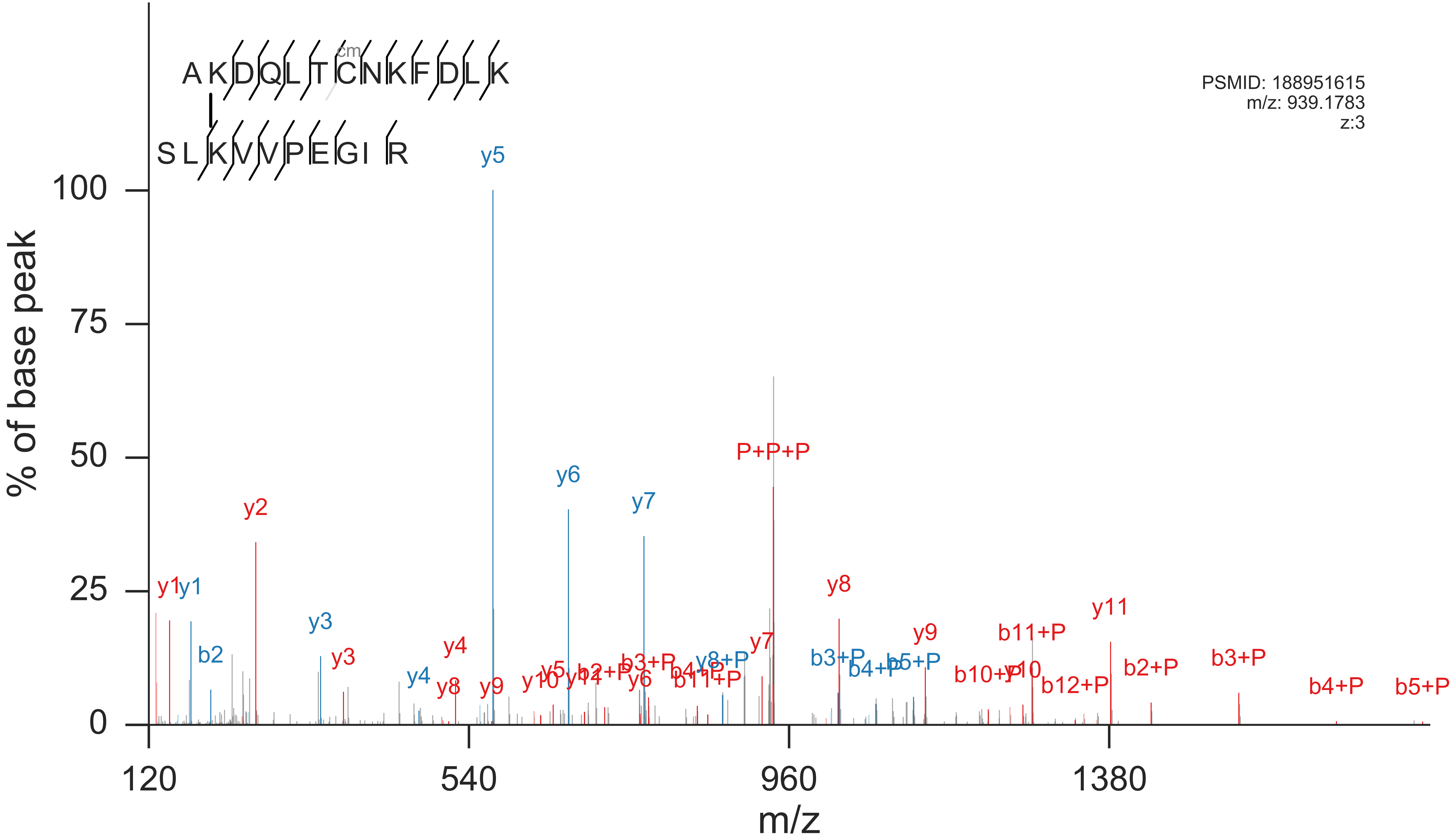

PSMID: 188951615
m/z: 939.1783
z:3
A K D Q L T C N K F D L K
cm
S L K V V P E G I R
% of base peak
m/z
100
75
50
25
0
120
540
960
1380
y5
y6
y7
P+P+P
y2
y1
y1
y3
b2
y3
y4
y4
y8
y9
y10
y5
y11
b2+P
b3+P
y6
b4+P
b11+P
y8+P
y7
b3+P
b4+P
b5+P
y8
y9
b10+P
y10
b11+P
y11
b12+P
b2+P
b3+P
b4+P
b5+P

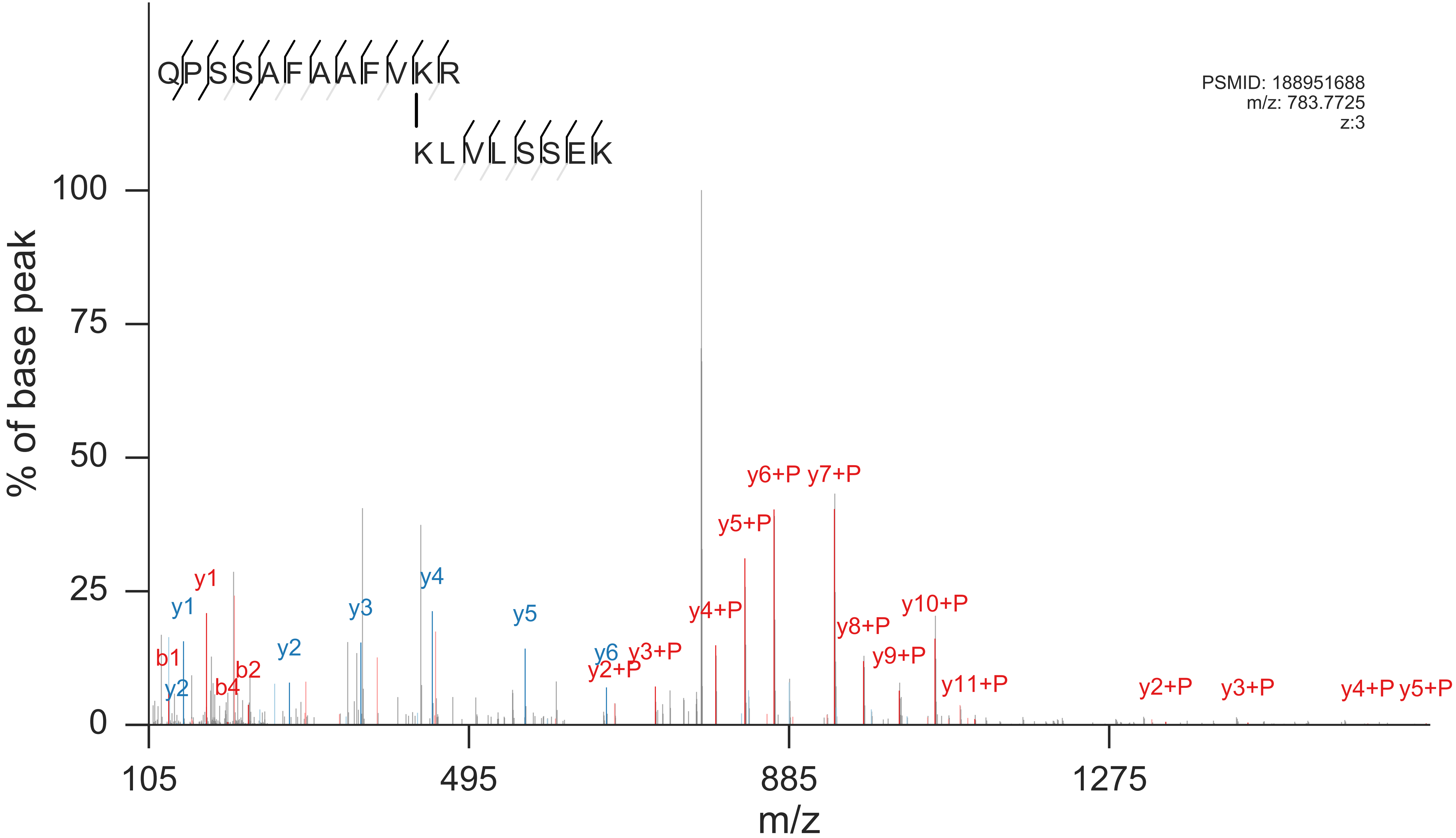

QPSSAFAAFVKR
KLVLSSEK
PSMID: 188951688
m/z: 783.7725
z:3
% of base peak
100
75
50
25
0
105
495
885
1275
m/z
b1
y2
y1
y1
b4
b2
y2
y3
y4
y5
y6
y2+P
y3+P
y4+P
y5+P
y6+P
y7+P
y8+P
y9+P
y10+P
y11+P
y2+P
y3+P
y4+P
y5+P

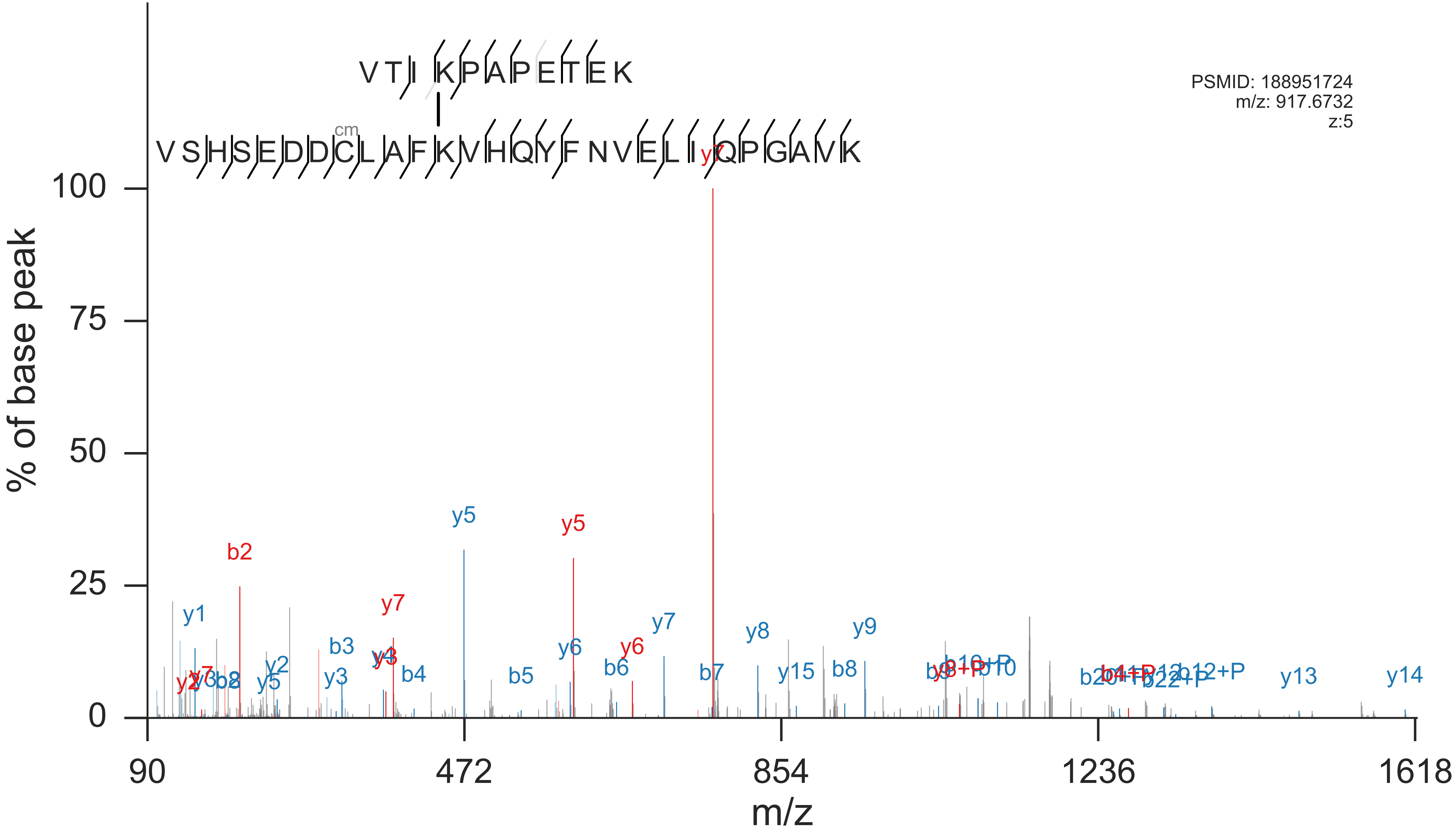

VTIKPAPETEK
VSHSEDDCLAFKVHQYFNVELIQPGAVK
cm
PSMID: 188951724
m/z: 917.6732
z:5
% of base peak
m/z
100
75
50
25
0
90
472
854
1236
1618

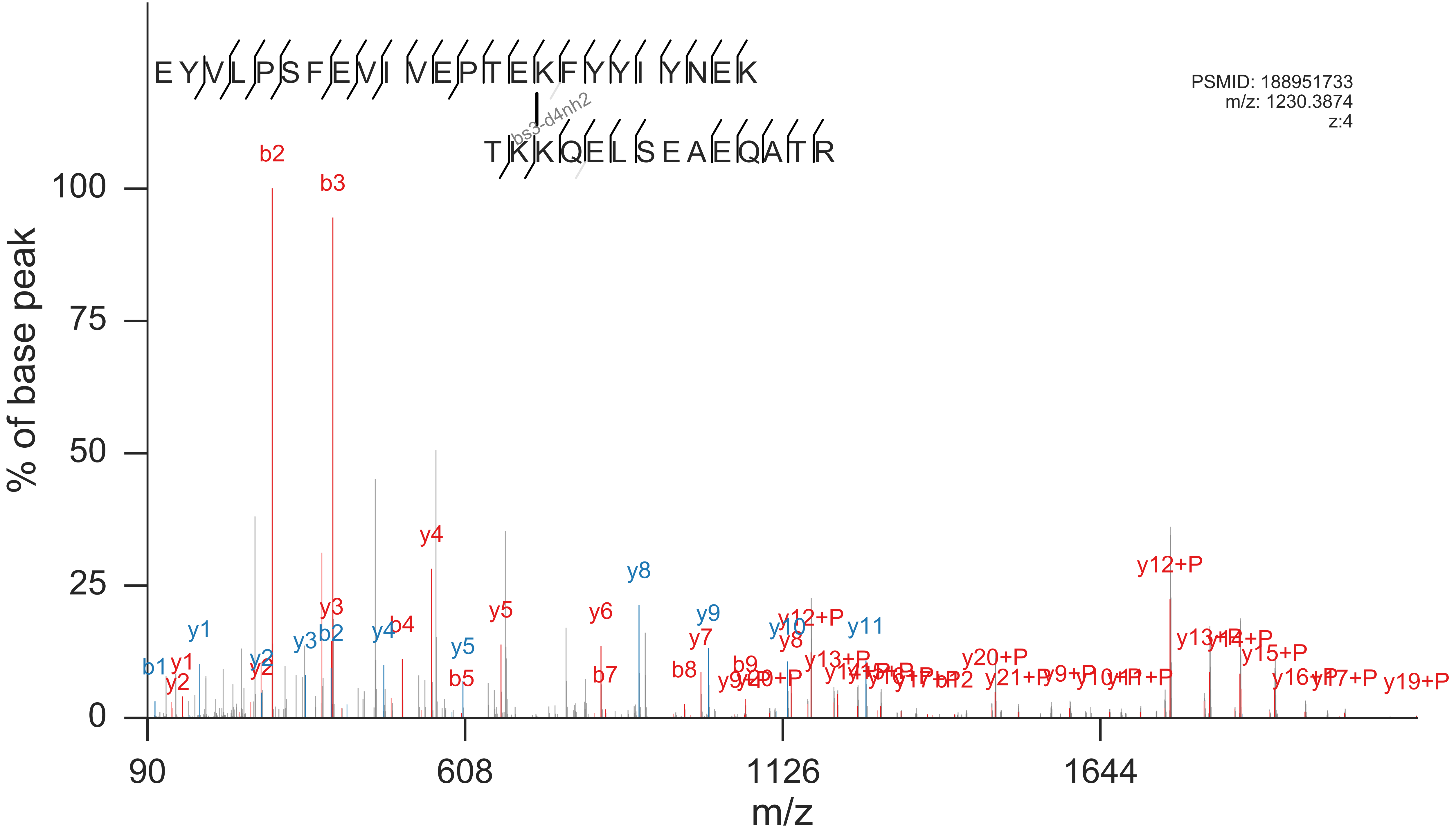

EYVLPSFEVIVEPTEKFYYIYNEK
bs3-d4nh2
TKKQELSEAEQATR
PSMID: 188951733
m/z: 1230.3874
z:4
% of base peak
m/z

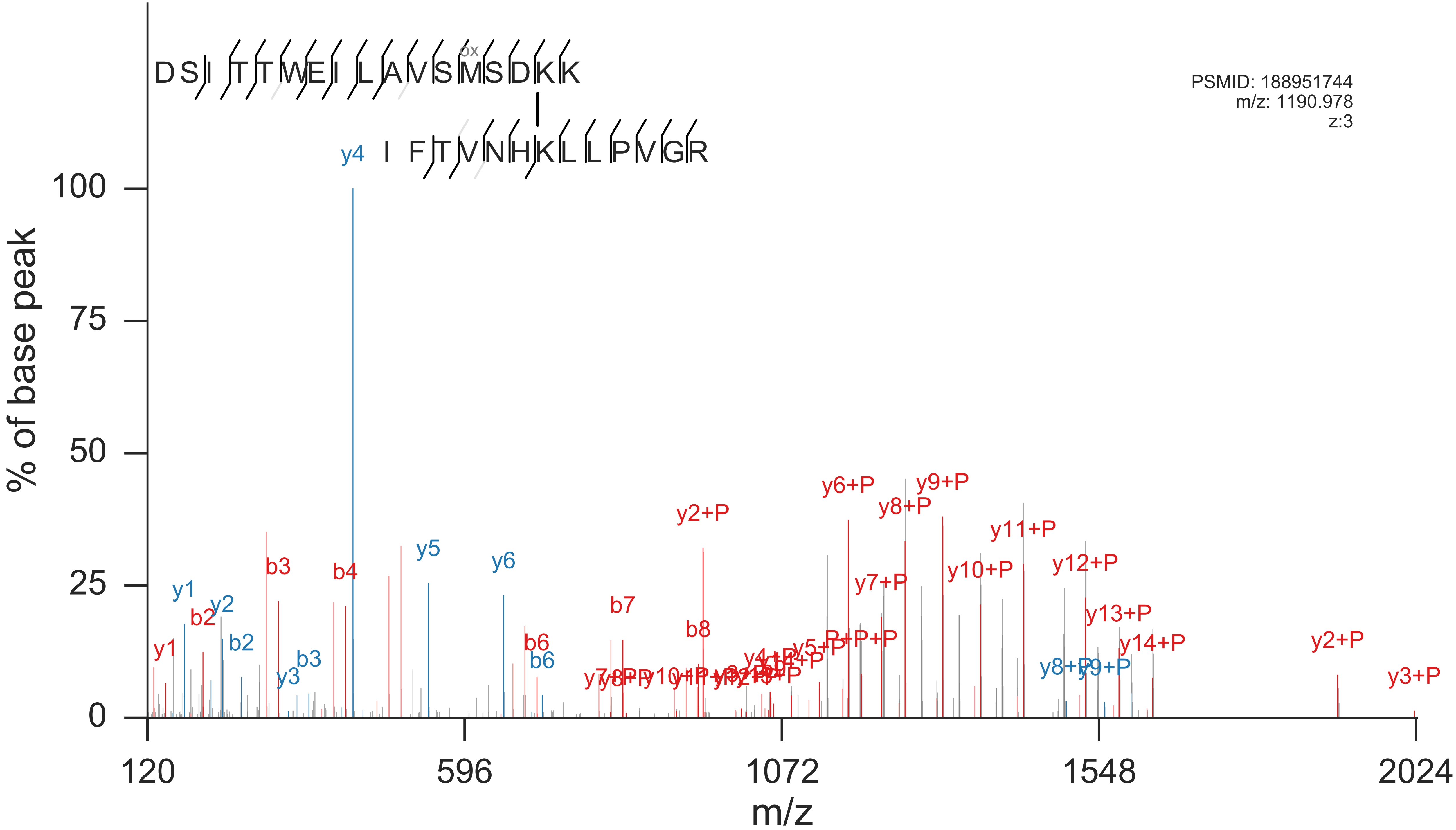
DSITTWEILAVSMSDKK
ox
IFTVNHKLLPVGR
PSMID: 188951744
m/z: 1190.978
z:3
% of base peak
m/z
100
75
50
25
0
120
596
1072
1548
2024
y1
y2
y3
y4
y5
y6
b2
b3
b6
b1
b2
b3
b4
b6
b7
b8
y2+P
y6+P
y7+P
y8+P
y9+P
y10+P
y11+P
y12+P
y13+P
y14+P
y8+P
y9+P
y2+P
y3+P

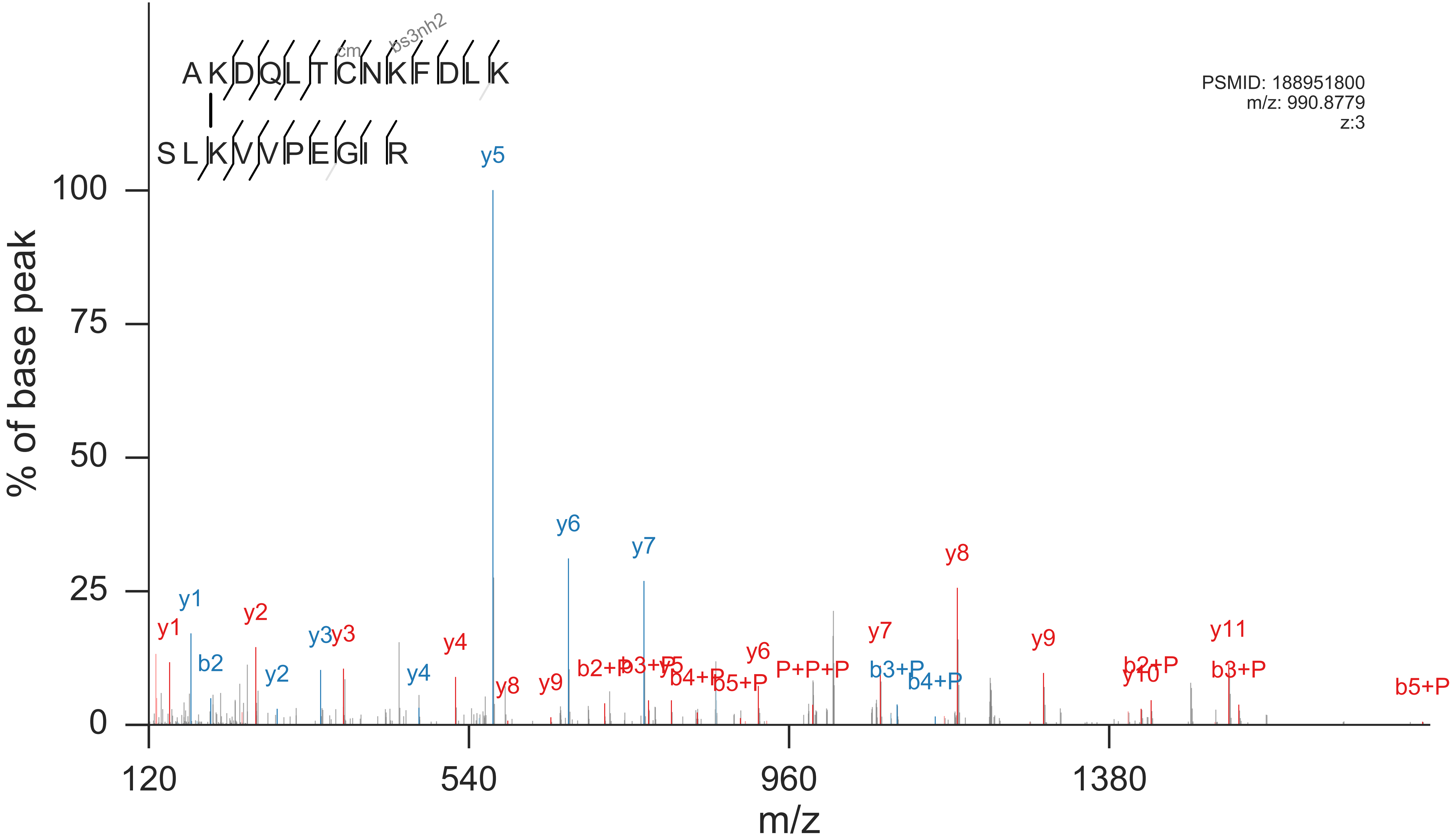
A K D Q L T C N K F D L K
cm
bs3nh2
S L K V V P E G I R
PSMID: 188951800
m/z: 990.8779
z:3
% of base peak
m/z
0
25
50
75
100
120
540
960
1380
y5
y6
y7
y8
y1
y1
y2
b2
y2
y3
y3
y4
y4
y8
y9
y6
y7
y9
y11
b3+P
b5+P
P+P+P
b3+P
b4+P
b5+P

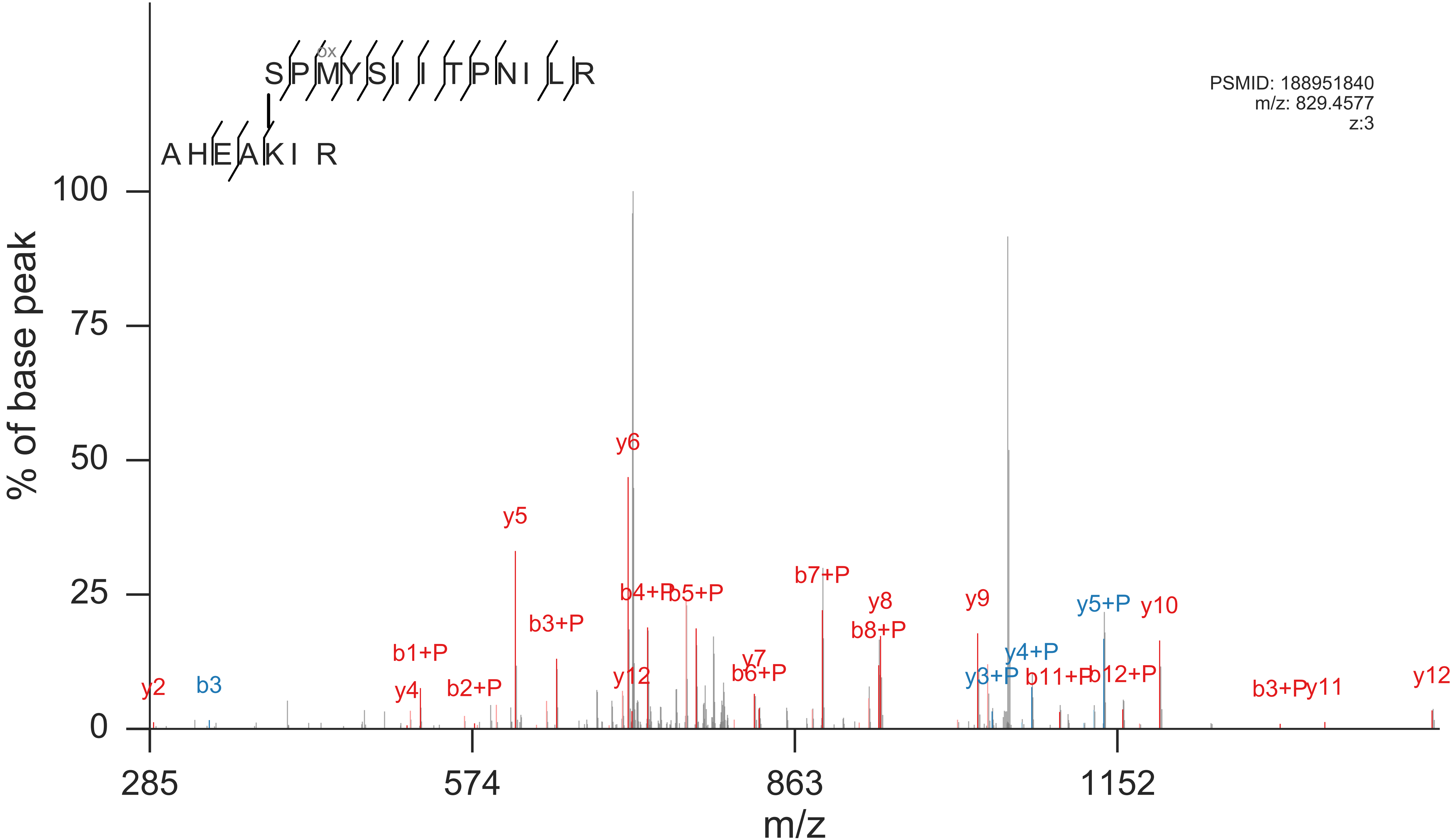
SPMYSIITPNILR
ox
AHEAKIR
PSMID: 188951840
m/z: 829.4577
z:3
% of base peak
m/z
y2
b3
y4
b1+P
b2+P
y5
b3+P
y6
y12
b4+P
b5+P
y7
b6+P
b7+P
y8
b8+P
y9
y3+P
y4+P
b11+P
y5+P
b12+P
y10
b3+P
y11
y12

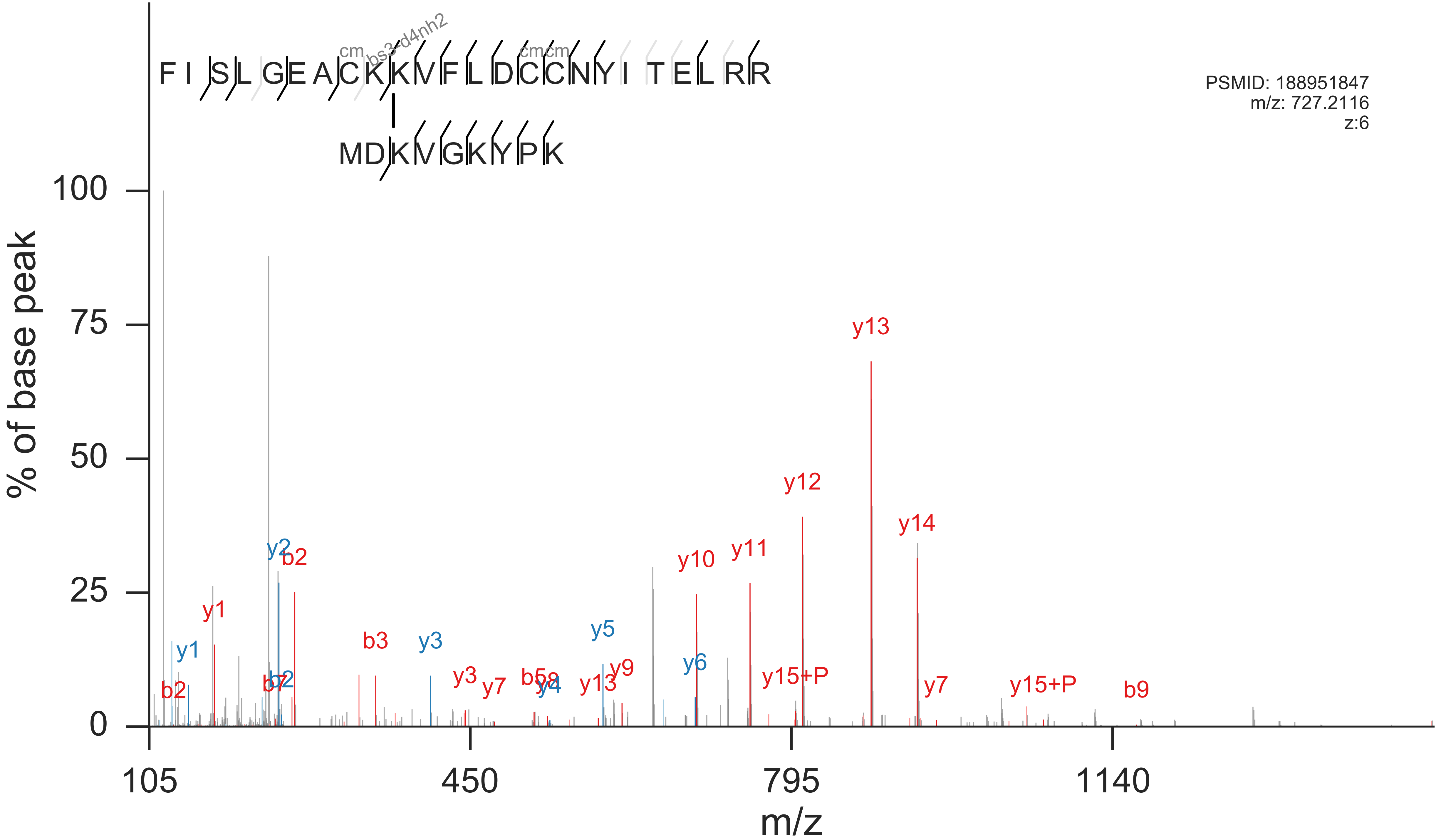
FISLGEACKKVFLDCCNYITELRR
MDKVGKYPK
cm
bs3-d4nh2
cm cm
PSMID: 188951847
m/z: 727.2116
z:6
% of base peak
m/z
100
75
50
25
0
105
450
795
1140
y13
y12
y14
y10
y11
y2
b2
y1
y1
b3
y3
y5
y6
y3
y7
b5
y4
y13
y9
y15+P
y7
y15+P
b9
b2
b7
b2

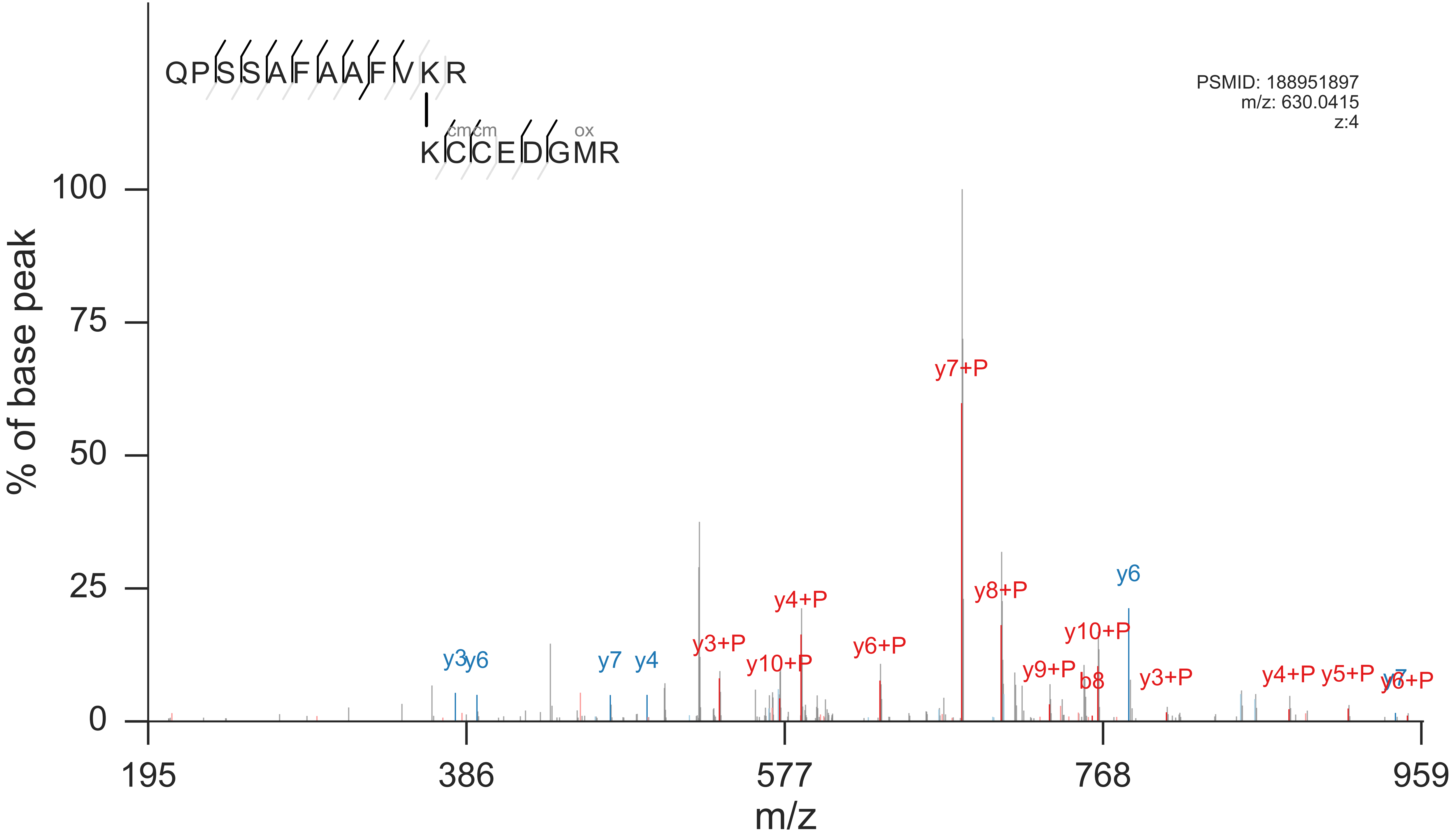
QPSSAFAAFVKR
KCCEDGMR
cm cm
ox
PSMID: 188951897
m/z: 630.0415
z:4
% of base peak
100
75
50
25
0
195
386
577
768
959
m/z
y7+P
y4+P
y8+P
y6
y10+P
y3+P
y6+P
y3
y6
y7
y4
y10+P
y9+P
b8
y3+P
y4+P
y5+P
y7
y6+P

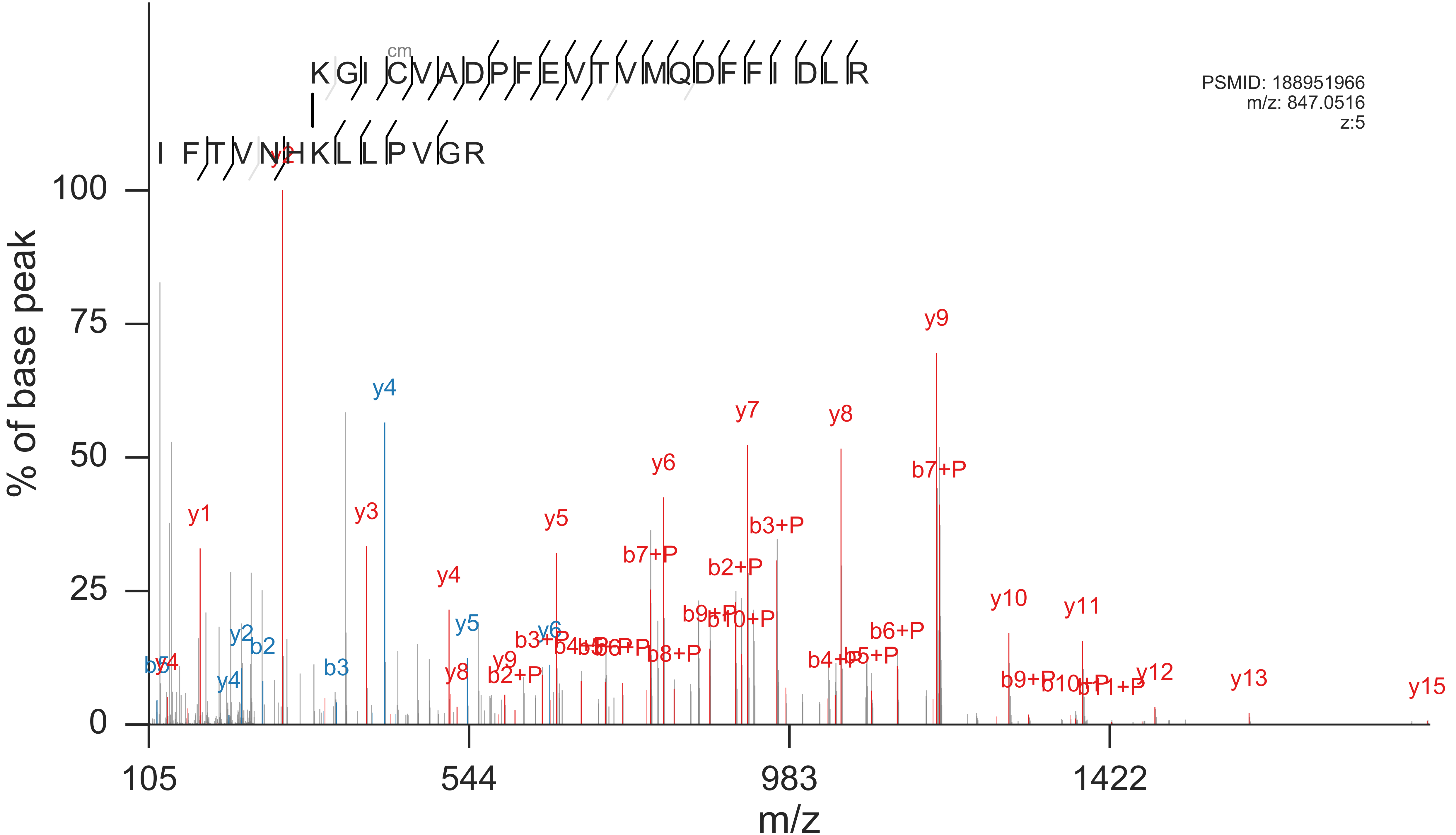

PSMID: 188951966
m/z: 847.0516
z:5
KGICVADPFEVTVMQDFFIDLR
cm
IFTVNHKLLPVGR
% of base peak
m/z

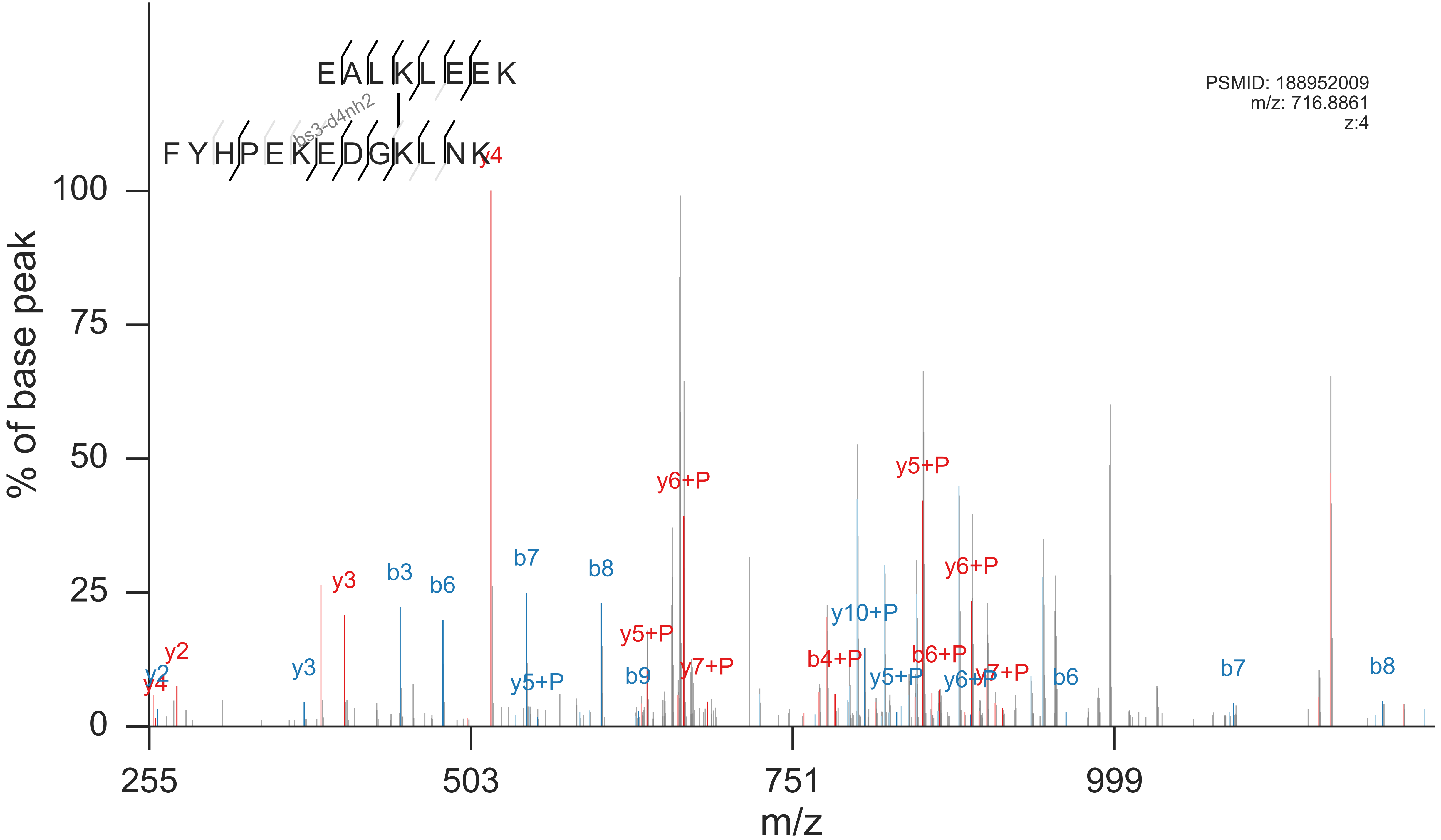
PSMID: 188952009
m/z: 716.8861
z:4
EALKLEEK
FYHPEKEDGKLNK
bs3-d4nh2
% of base peak
m/z
100
75
50
25
0
255
503
751
999
y4
y2
y2
y4
y3
y3
b3
b6
b7
b8
y5+P
y5+P
b9
y6+P
y7+P
b4+P
y10+P
y5+P
y5+P
b6+P
y6+P
y6+P
y7+P
b6
b7
b8

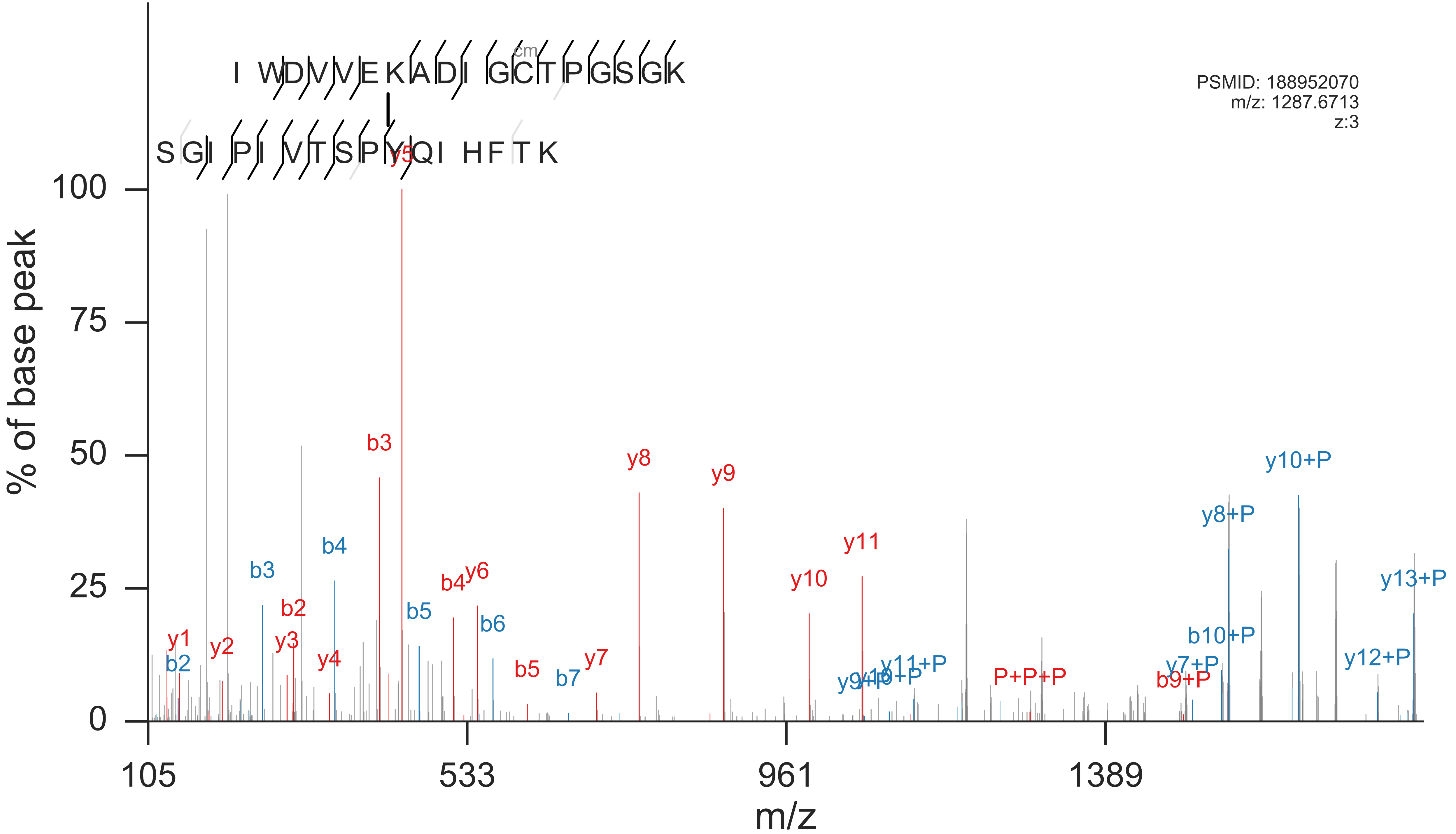
PSMID: 188952070
m/z: 1287.6713
z:3
I W D V V E K A D I G C T P G S G K
cm
S G I P I V T S P Y Q I H F T K
% of base peak
m/z
100
75
50
25
0
105
533
961
1389
y1
y2
y3
y4
y5
y6
y7
y8
y9
y10
y11
b2
b3
b4
b5
y7+P
y8+P
y10+P
y11+P
y12+P
y13+P
b10+P
b9+P
P+P+P
b6
b7

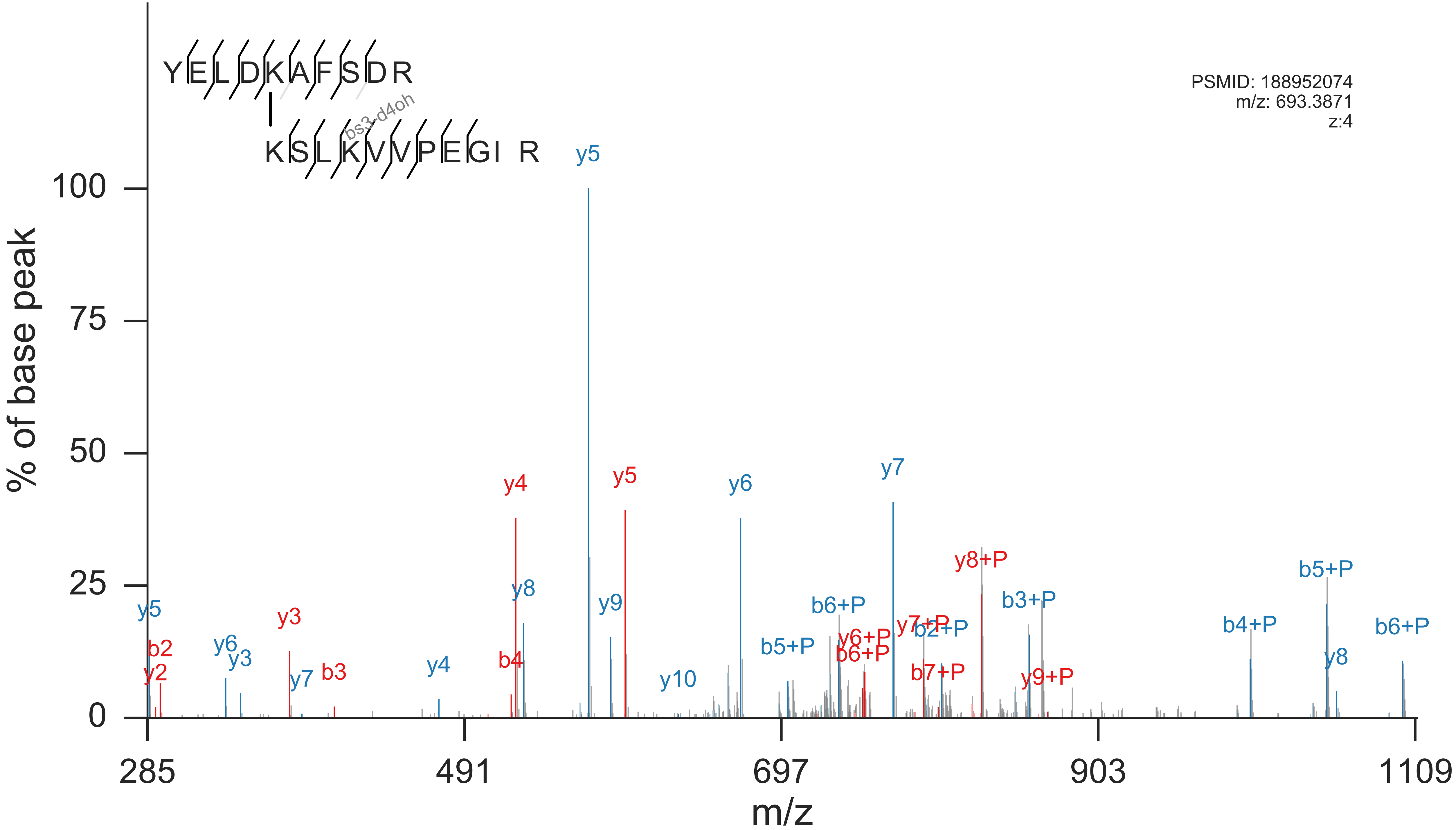

YELDKAFSDR
KSLKVVPEGIR
bs3-d4oh
PSMID: 188952074
m/z: 693.3871
z:4
% of base peak
m/z
100
75
50
25
0
285
491
697
903
1109
y5
y4
y5
y6
y7
y8+P
b5+P
y8
y9
b6+P
b3+P
b4+P
b6+P
y5
y3
b2
y6
y3
y2
y7
b3
y4
b4
y10
b5+P
y6+P
b6+P
y7+P
b2+P
b7+P
y9+P
y8

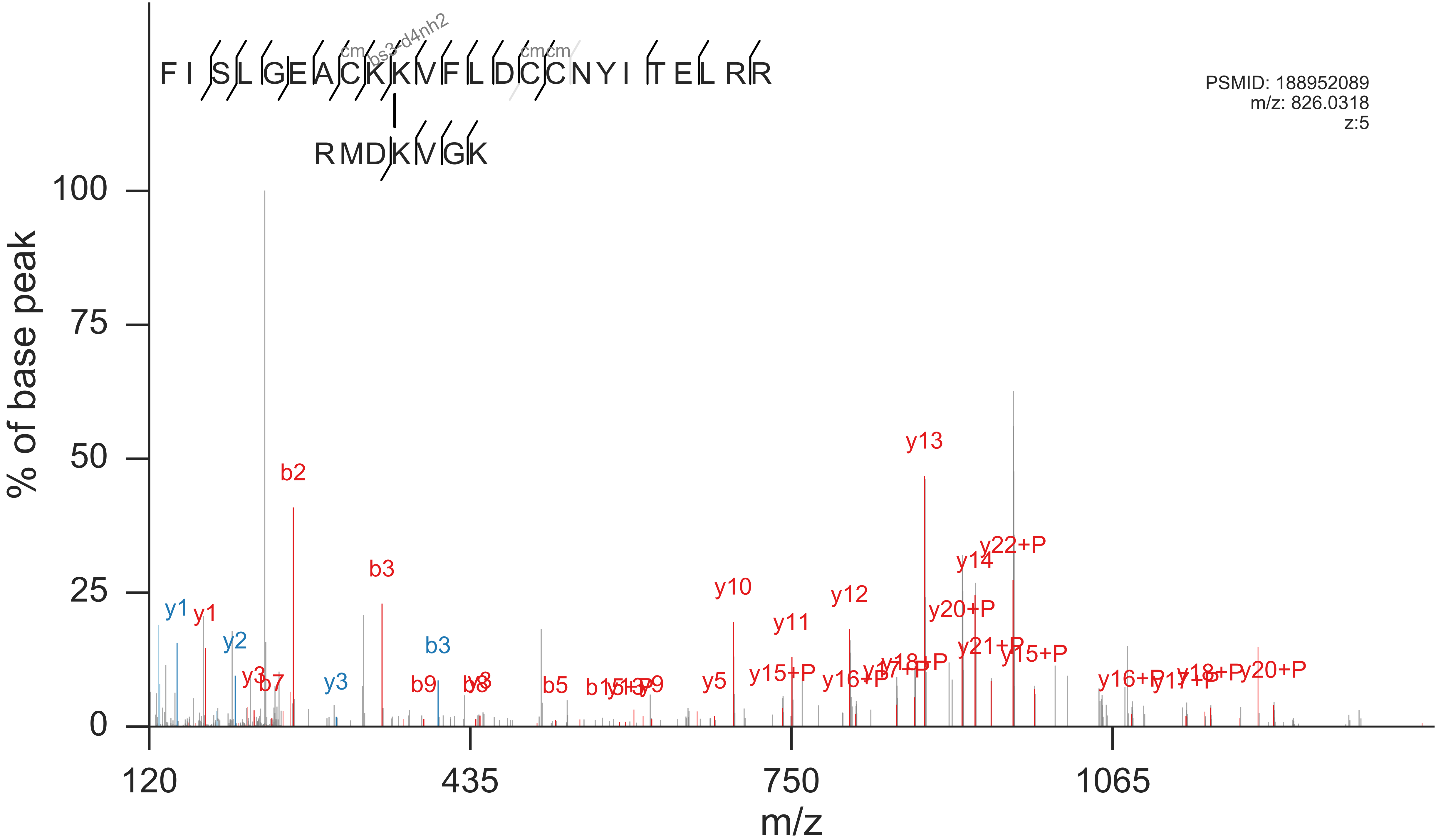
FISLGEACKKVFLDCCNYITELRR
RMDKVGK
PSMID: 188952089
m/z: 826.0318
z:5
% of base peak
m/z
0
25
50
75
100
120
435
750
1065
b2
b3
y13
y14
y22+P
y10
y11
y12
y20+P
y21+P
y15+P
b5
y5
b9
y3
y1
y2
b3

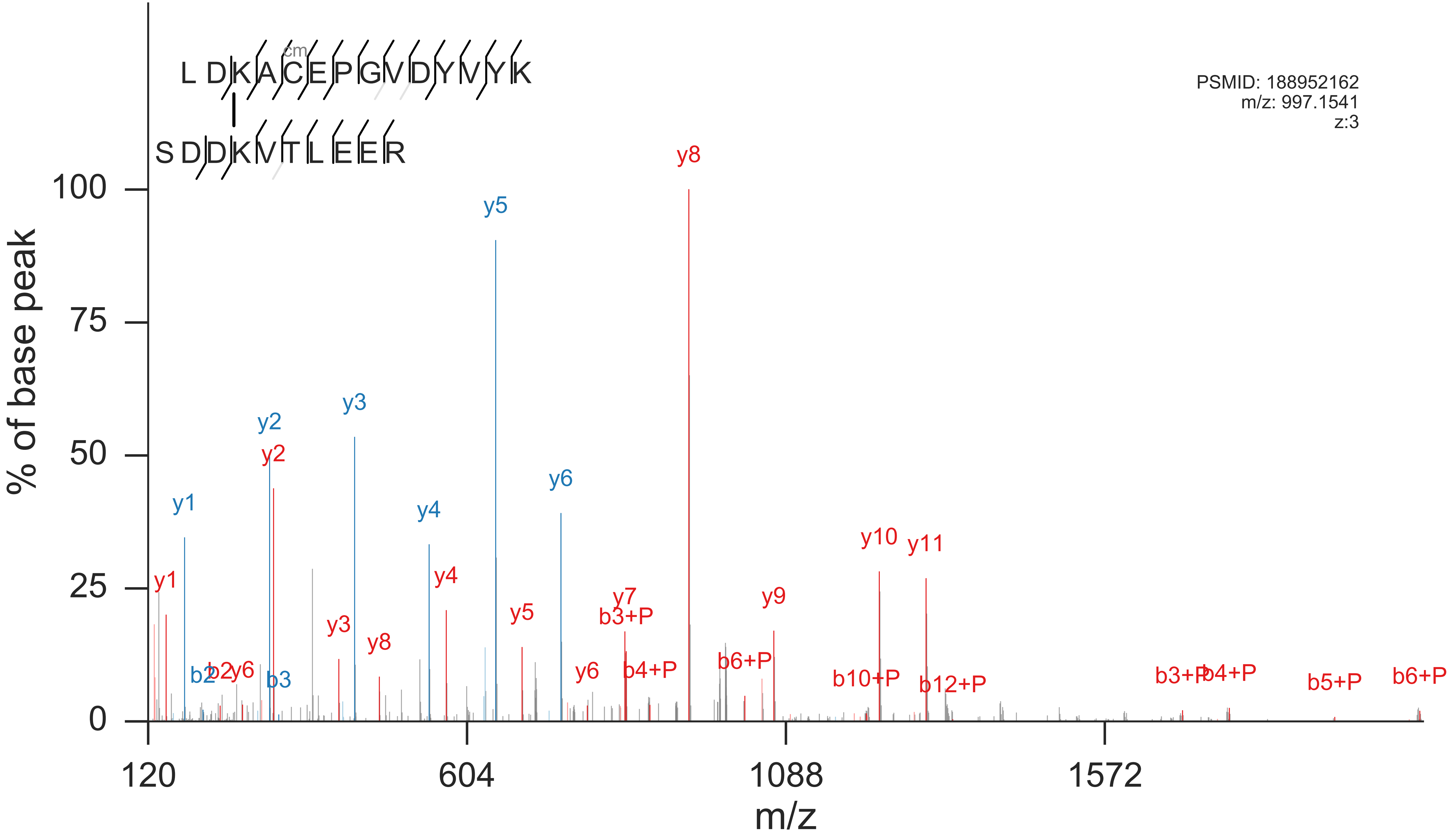

L D K A C E P G V D Y V Y K
cm
S D D K V T L E E R
PSMID: 188952162
m/z: 997.1541
z:3
% of base peak
m/z
0
25
50
75
100
120
604
1088
1572
y1
y2
y3
y4
y5
y6
y8
y10
y11
y9
y7
b2
b3
b3+P
b4+P
b6+P
b10+P
b12+P
b5+P

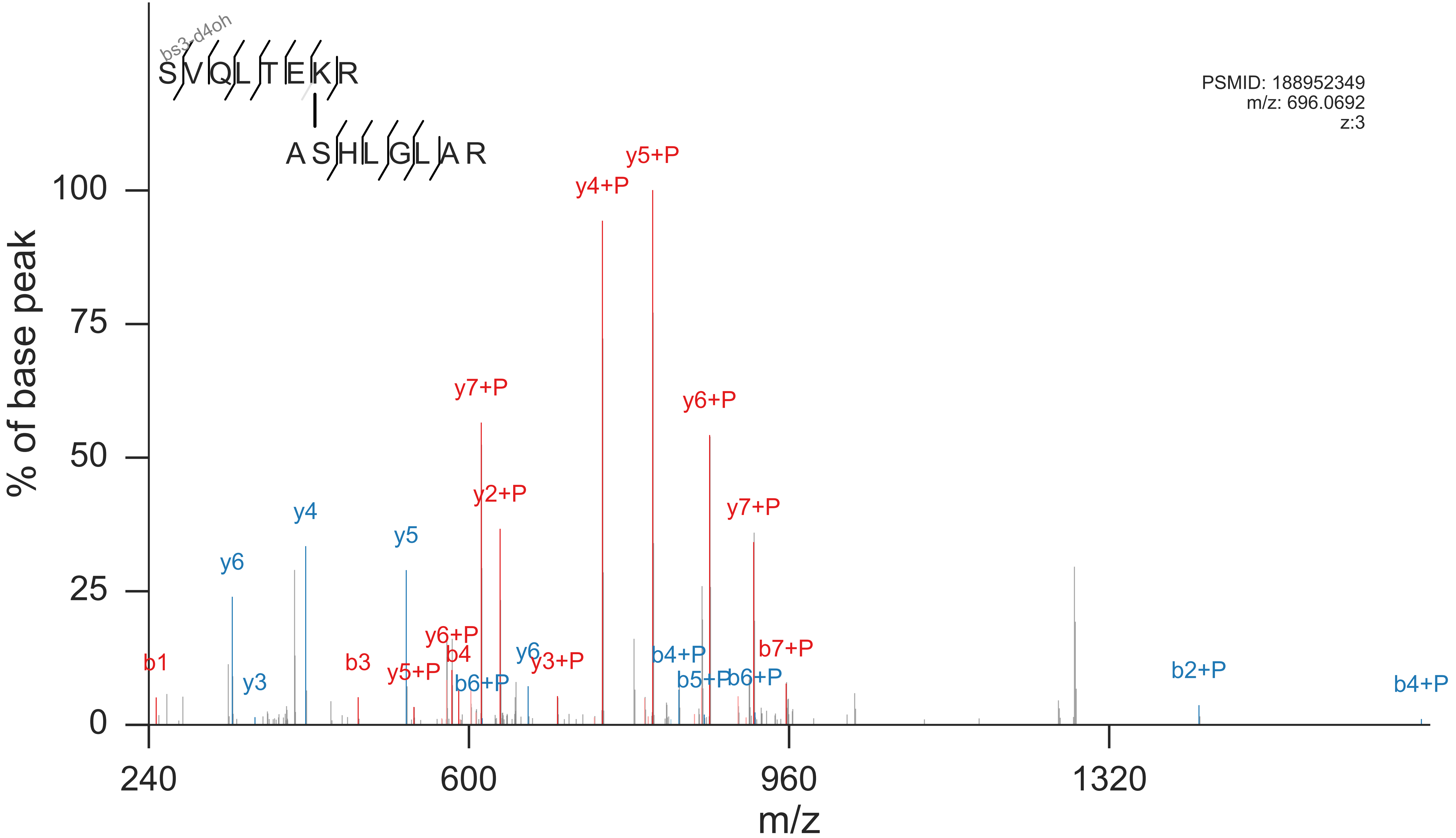

bs3-d4oh
SVQLTEKR
ASHLGLAR
PSMID: 188952349
m/z: 696.0692
z:3
% of base peak
m/z
100
75
50
25
0
240
600
960
1320
y5+P
y4+P
y7+P
y6+P
y2+P
y7+P
y4
y5
y6
y6+P
b4
b1
b3
y3
y5+P
b6+P
y6
y3+P
b4+P
b5+P
b6+P
b7+P
b2+P
b4+P

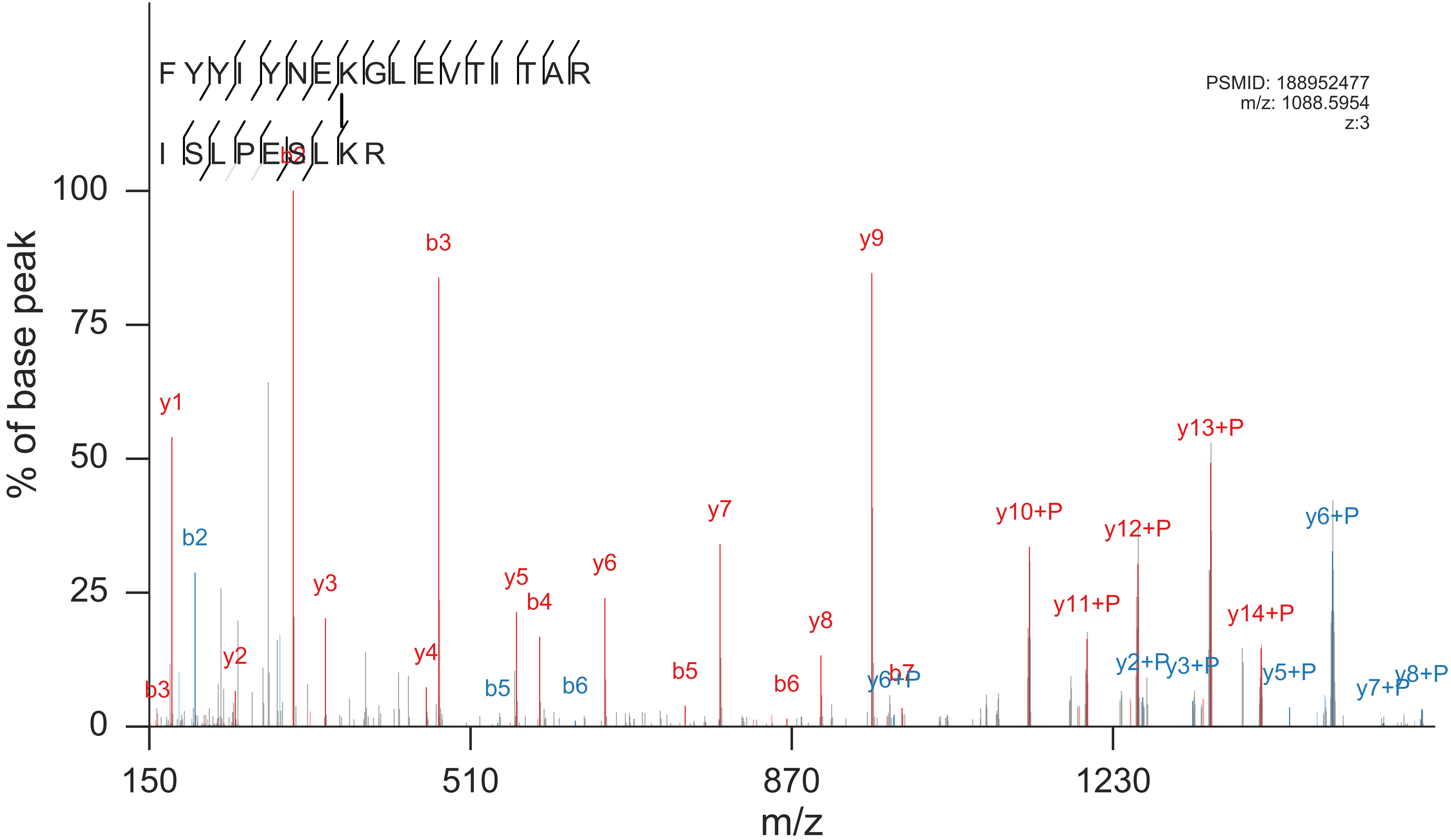

F Y Y I Y N E K G L E V T I T A R
I S L P E S L K R
PSMID: 188952477
m/z: 1088.5954
z:3
% of base peak
m/z
100
75
50
25
0
150
510
870
1230
y1
b2
b3
y2
b3
y3
y4
y5
b4
b5
y6
b6
y7
b5
b6
y8
y9
y6+P
b7
y10+P
y11+P
y12+P
y2+P
y3+P
y13+P
y14+P
y5+P
y6+P
y7+P
y8+P

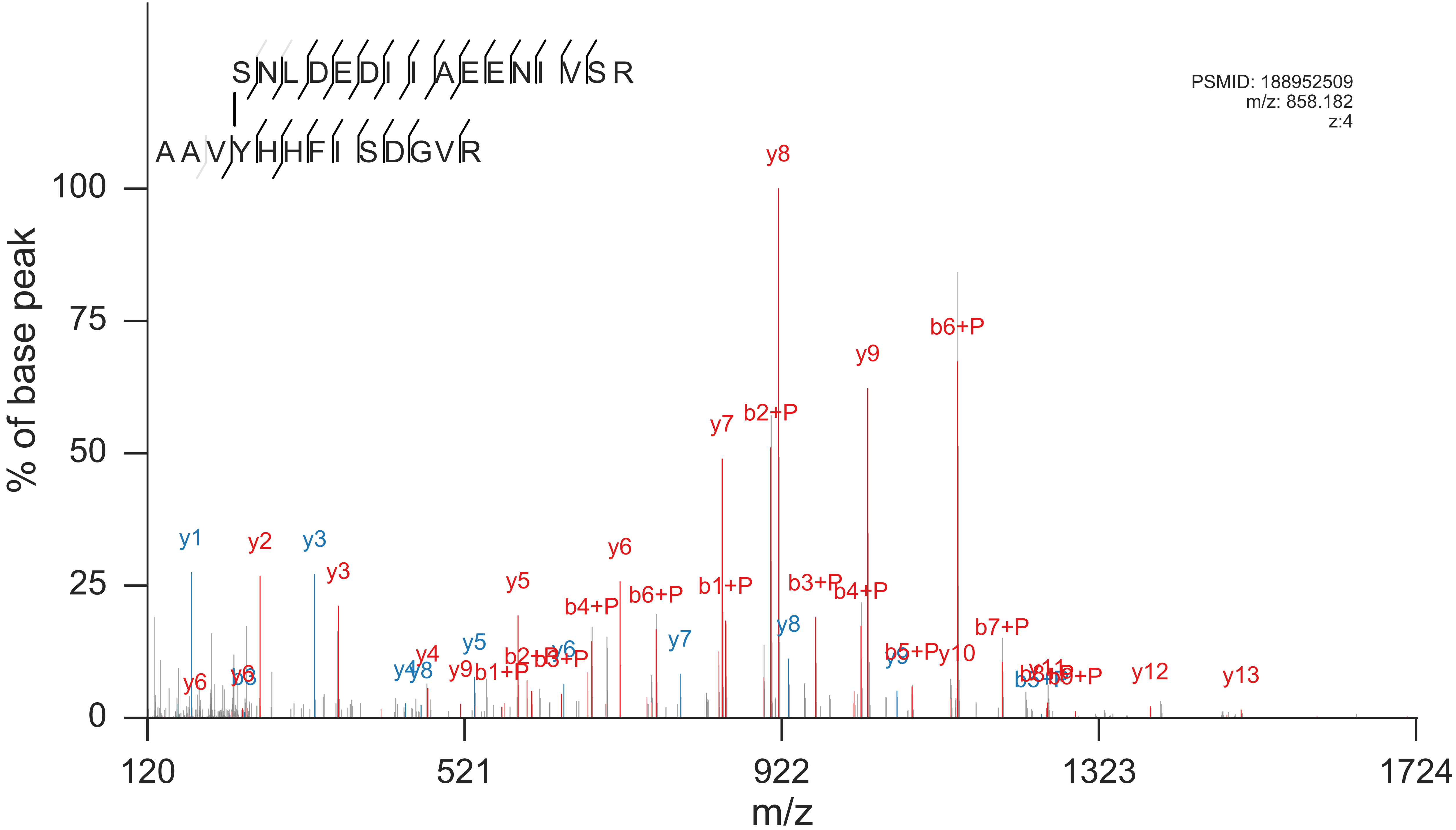

SNLDEDIIAEENIVSR
AAVYHHFISDGVR
PSMID: 188952509
m/z: 858.182
z:4
% of base peak
m/z
y8
b6+P
y9
y7
b2+P
y1
y2
y3
y6
b1+P
b3+P
b4+P
y5
b7+P
y12
y13
y10

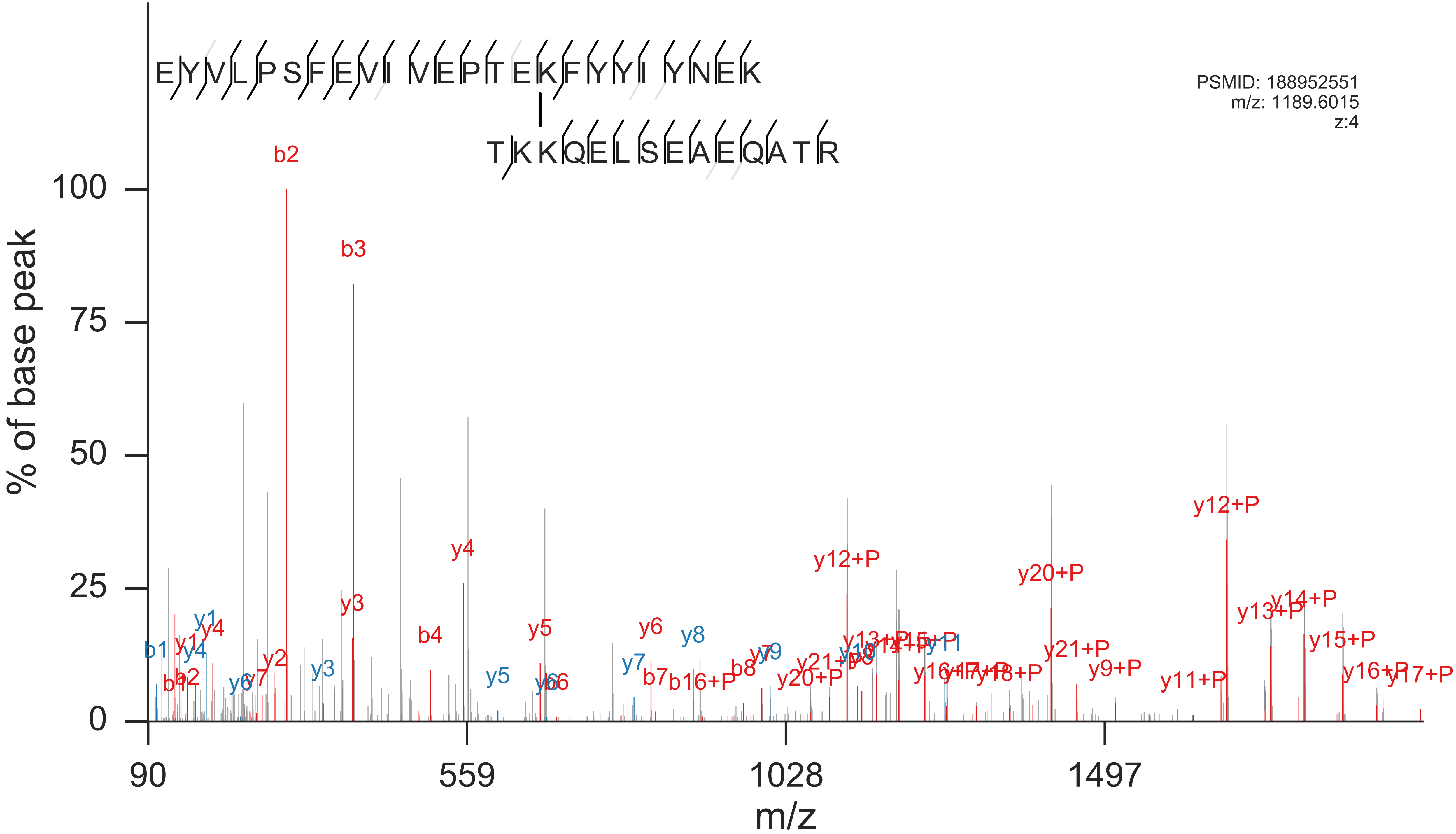

PSMID: 188952551
m/z: 1189.6015
z:4
% of base peak
m/z

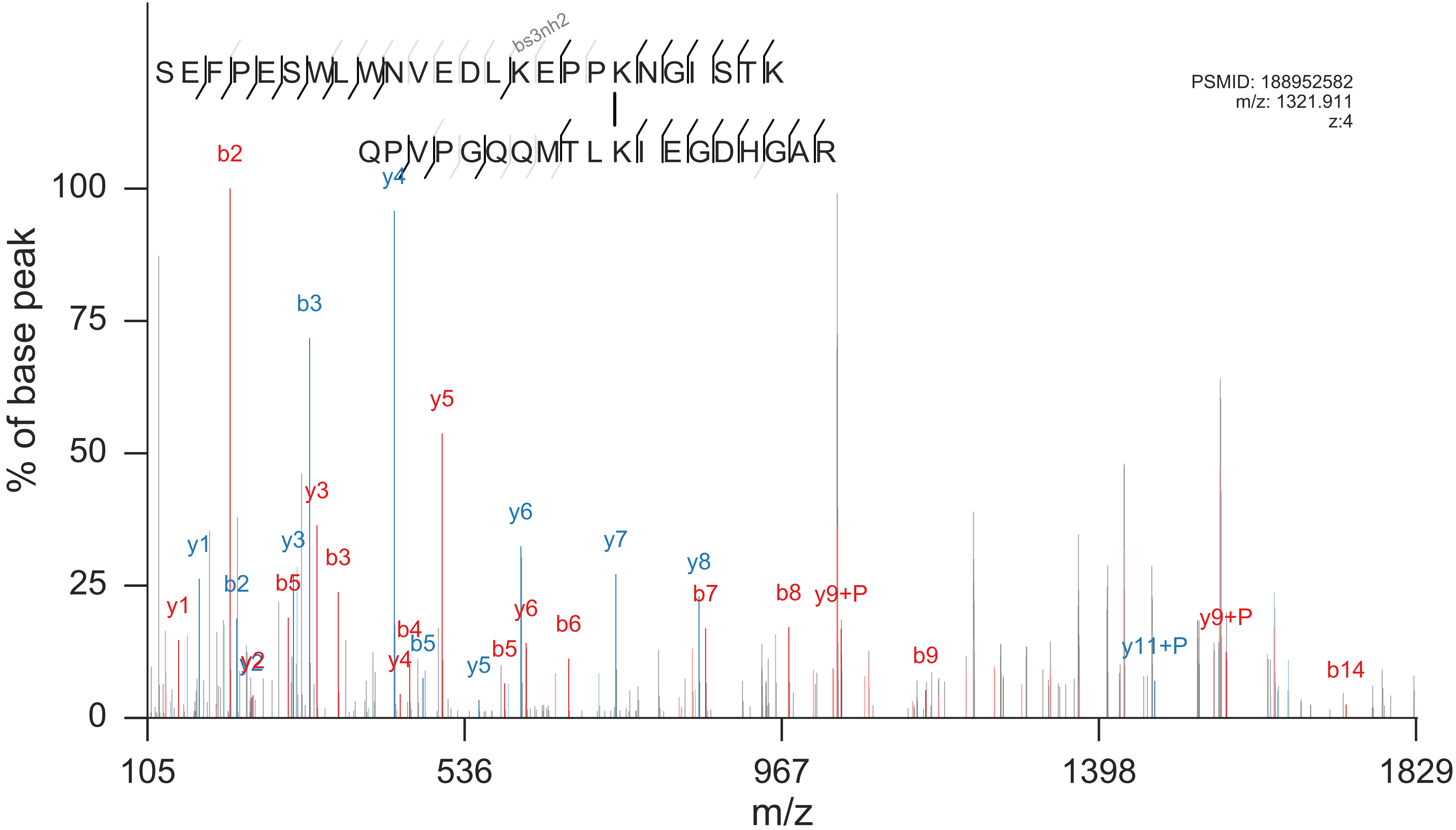
PSMID: 188952582
m/z: 1321.911
z:4
SEFPESWLWNVEDLKEPPKNGISTK
QPVPGQQMTLKIEGDHGAR
bs3nh2
% of base peak
m/z
0
25
50
75
100
105
536
967
1398
1829
b2
y4
b3
y5
y3
y6
y7
y1
y3
b3
y8
b2
b5
y1
b7
b8
y9+P
y9+P
b4
b5
y6
b6
y2
y4
y5
b5
b9
y11+P
b14

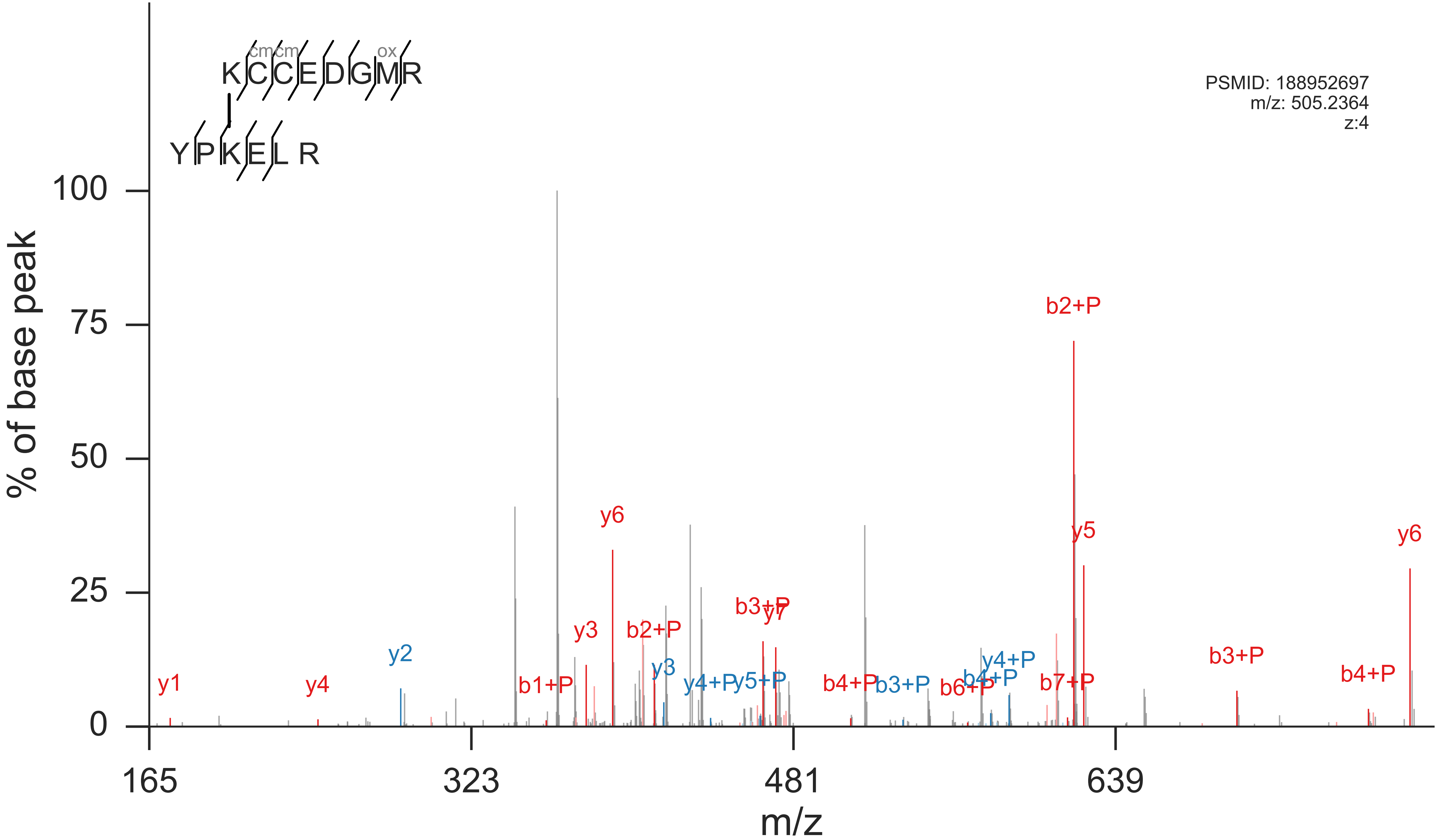

KCCEDGMR
cm cm ox
YPKELR
PSMID: 188952697
m/z: 505.2364
z:4
% of base peak
m/z

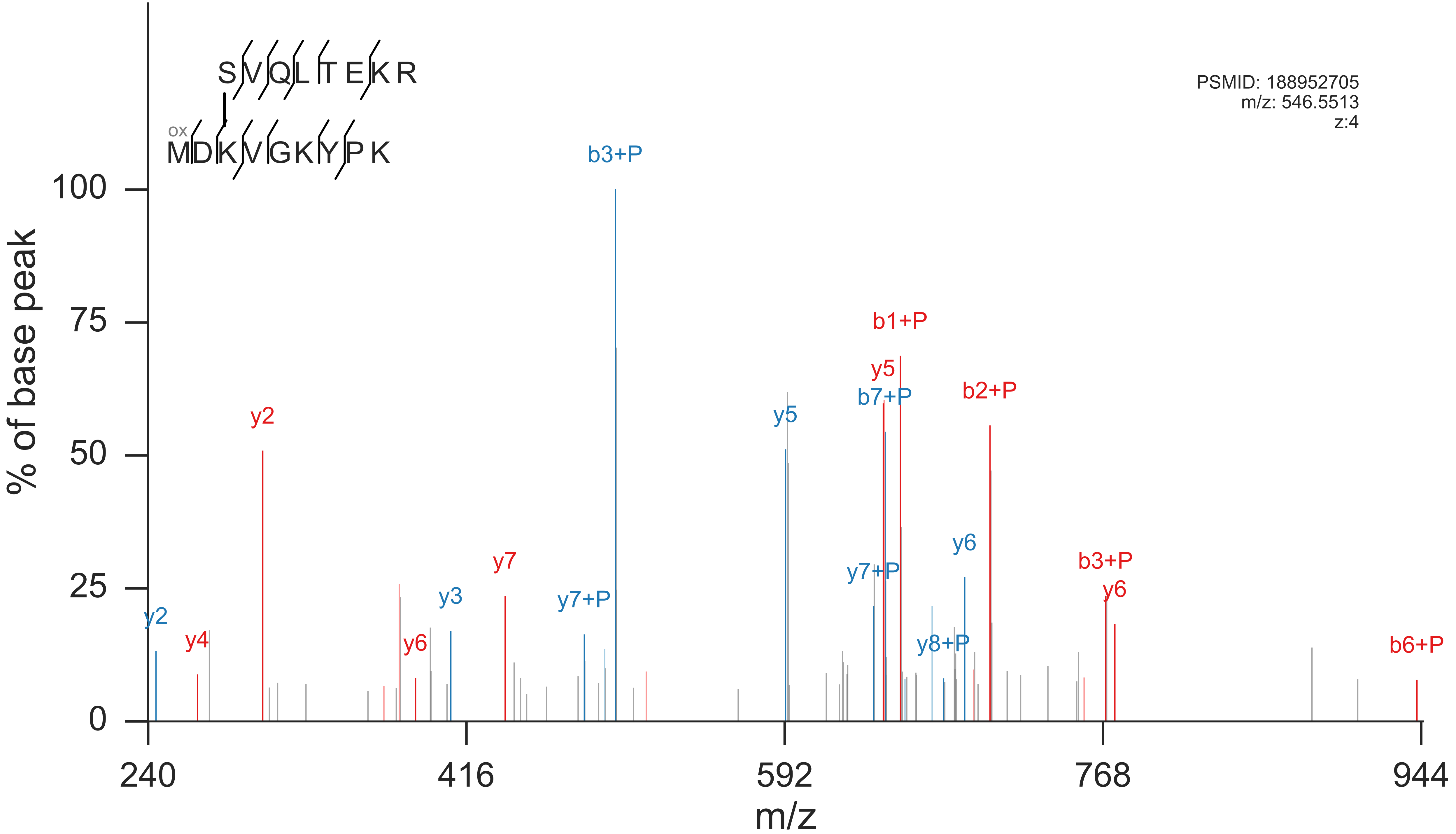

SVQLTEKR
oxMDKVGKYPK
PSMID: 188952705
m/z: 546.5513
z:4
% of base peak
m/z
b3+P
b1+P
y5
b7+P
b2+P
y5
y2
y2
y4
y6
y3
y7
y7+P
y7+P
y6
y8+P
b3+P
y6
b6+P
100
75
50
25
0
240
416
592
768
944

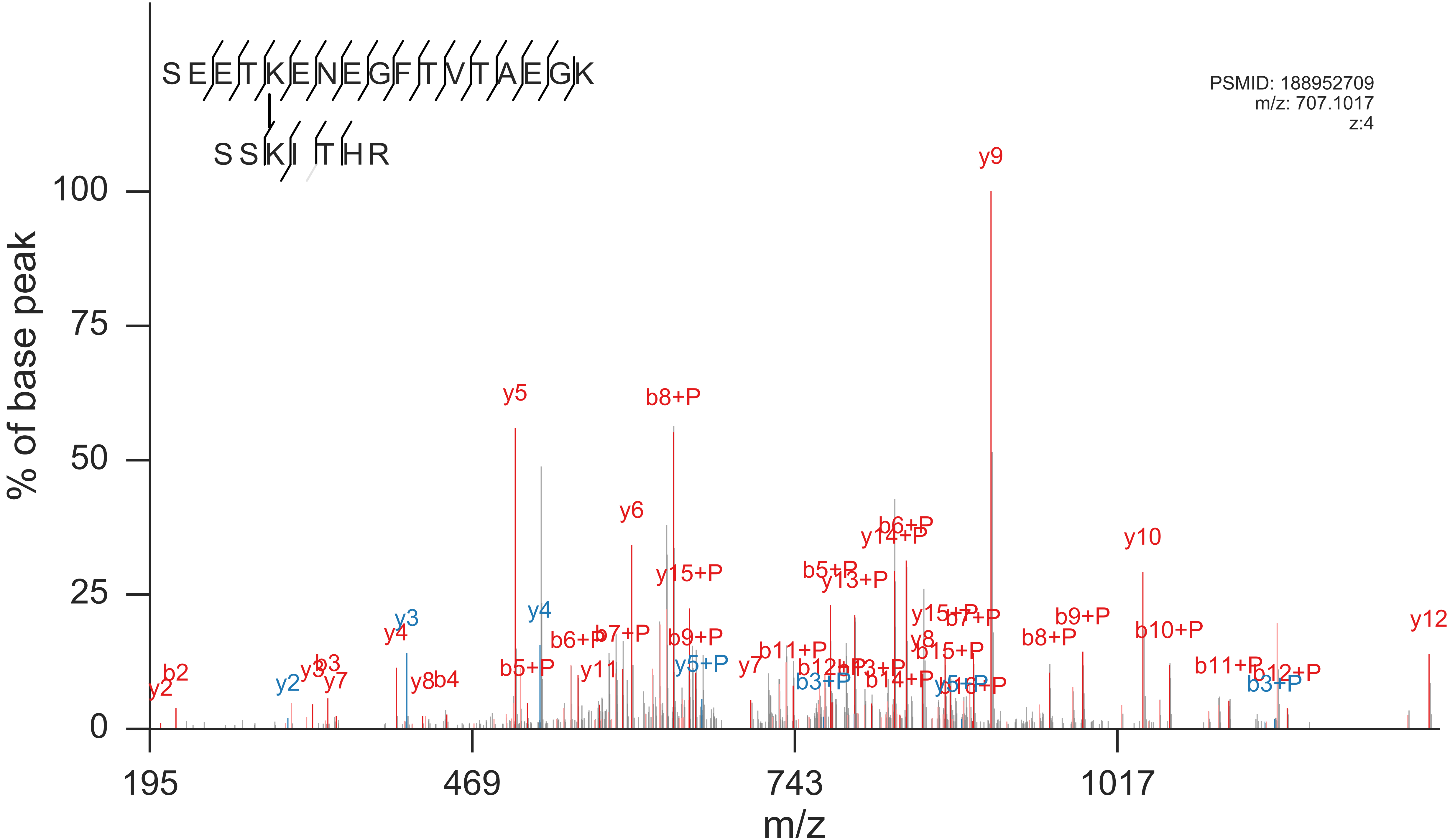
SEETKENEGFTVTAEGK
SSKITHR
PSMID: 188952709
m/z: 707.1017
z:4
% of base peak
m/z
0
25
50
75
100
195
469
743
1017
y9
y5
b8+P
y6
y10
y12
y4
y3
b2
y2
b3
y7
b4
b5+P
b6+P
b7+P
b9+P
y11
y15+P
y5+P
b11+P
b12+P
b3+P
y13+P
b5+P
y14+P
b6+P
y8
b15+P
b14+P
b8+P
b9+P
b10+P
b11+P
b12+P

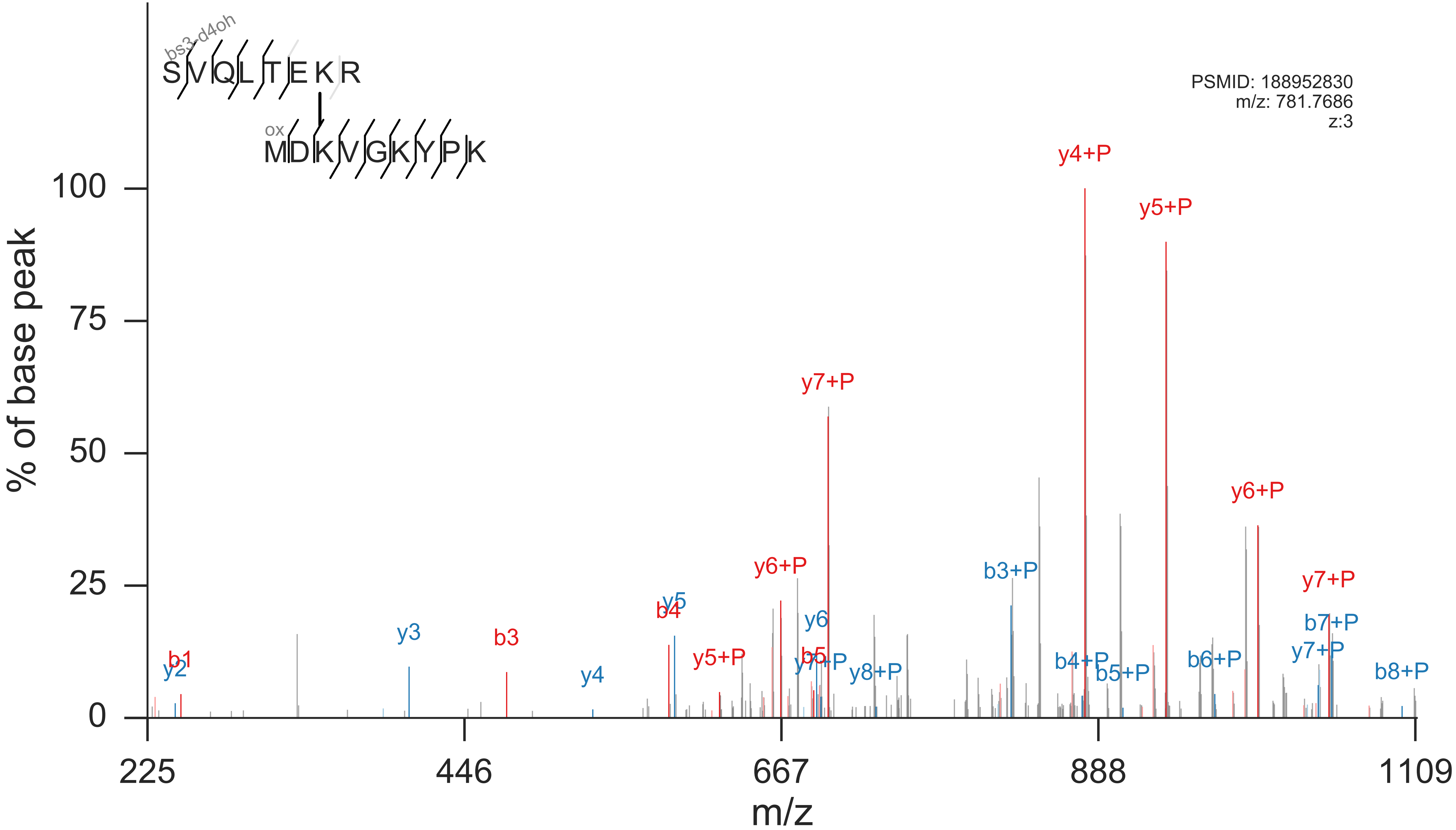

bs3-d4oh
SVQLTEKR
ox
MDKVGKYPK
PSMID: 188952830
m/z: 781.7686
z:3
% of base peak
m/z

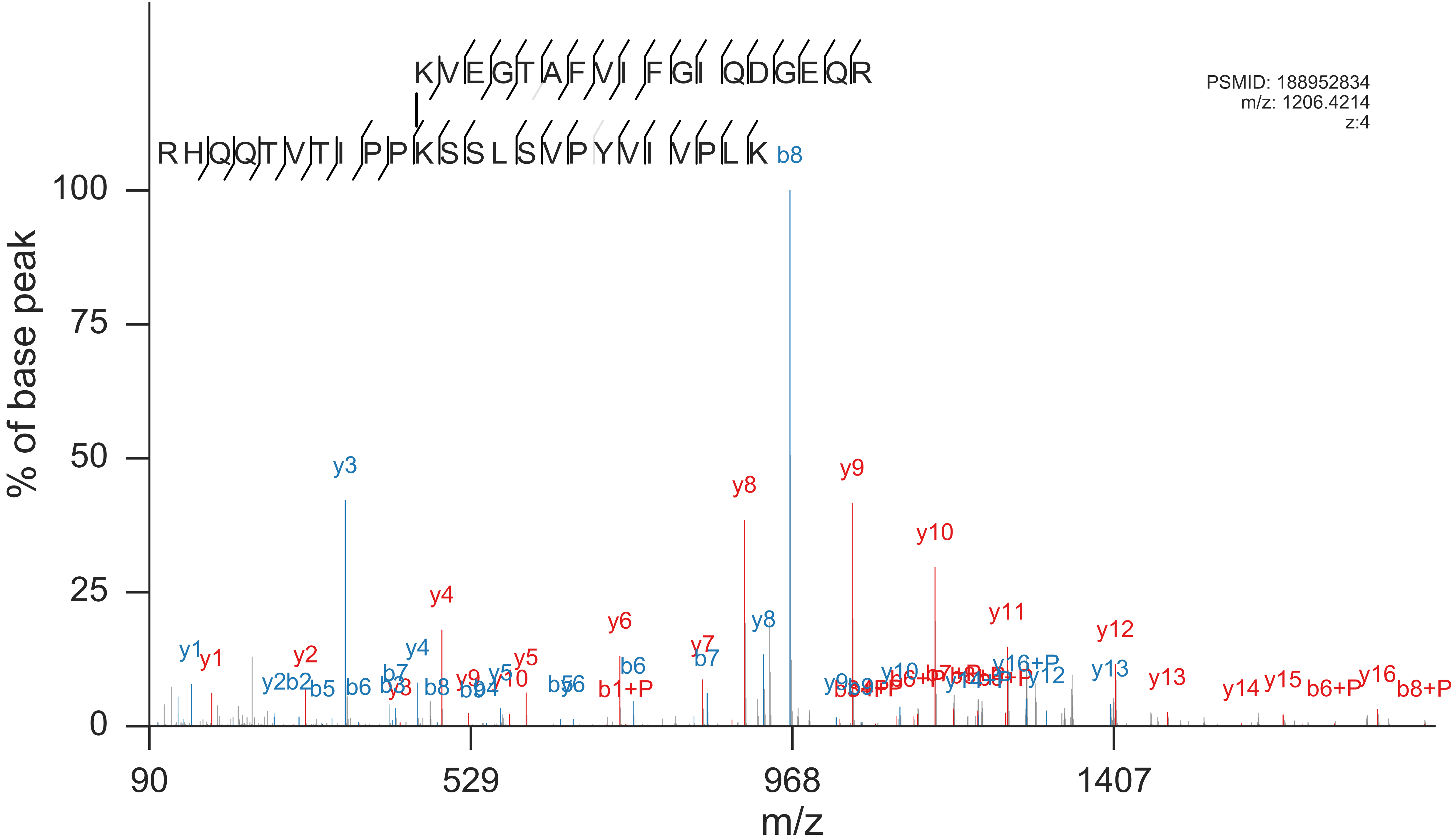
KVEGTAFVIFGIQDGEQR
RHQQTVTIPPKSSLSVPYVIVPLK
PSMID: 188952834
m/z: 1206.4214
z:4
% of base peak
100
75
50
25
0
90
529
968
1407
m/z
b8
y3
y8
y9
y10
y4
y6
y11
y12
y13
y14
y15
y16
b6+P
b8+P
b1+P
y5
y2
y1
b5
b6
b7
b8
y7

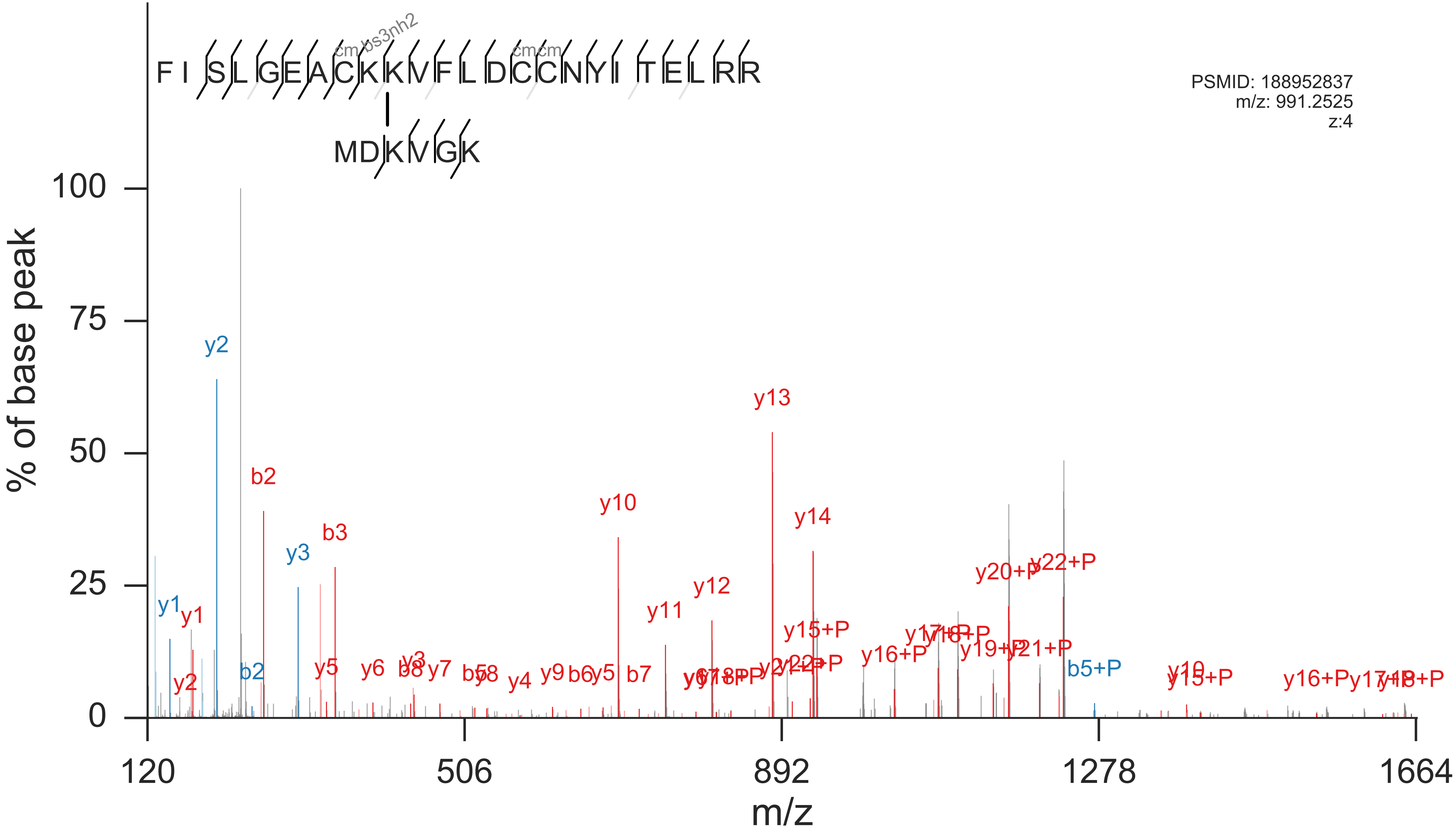

F I S L G E A C K K V F L D C C N Y I T E L R R
cm bs3nh2
cm cm
MD K V G K
PSMID: 188952837
m/z: 991.2525
z:4
% of base peak
m/z

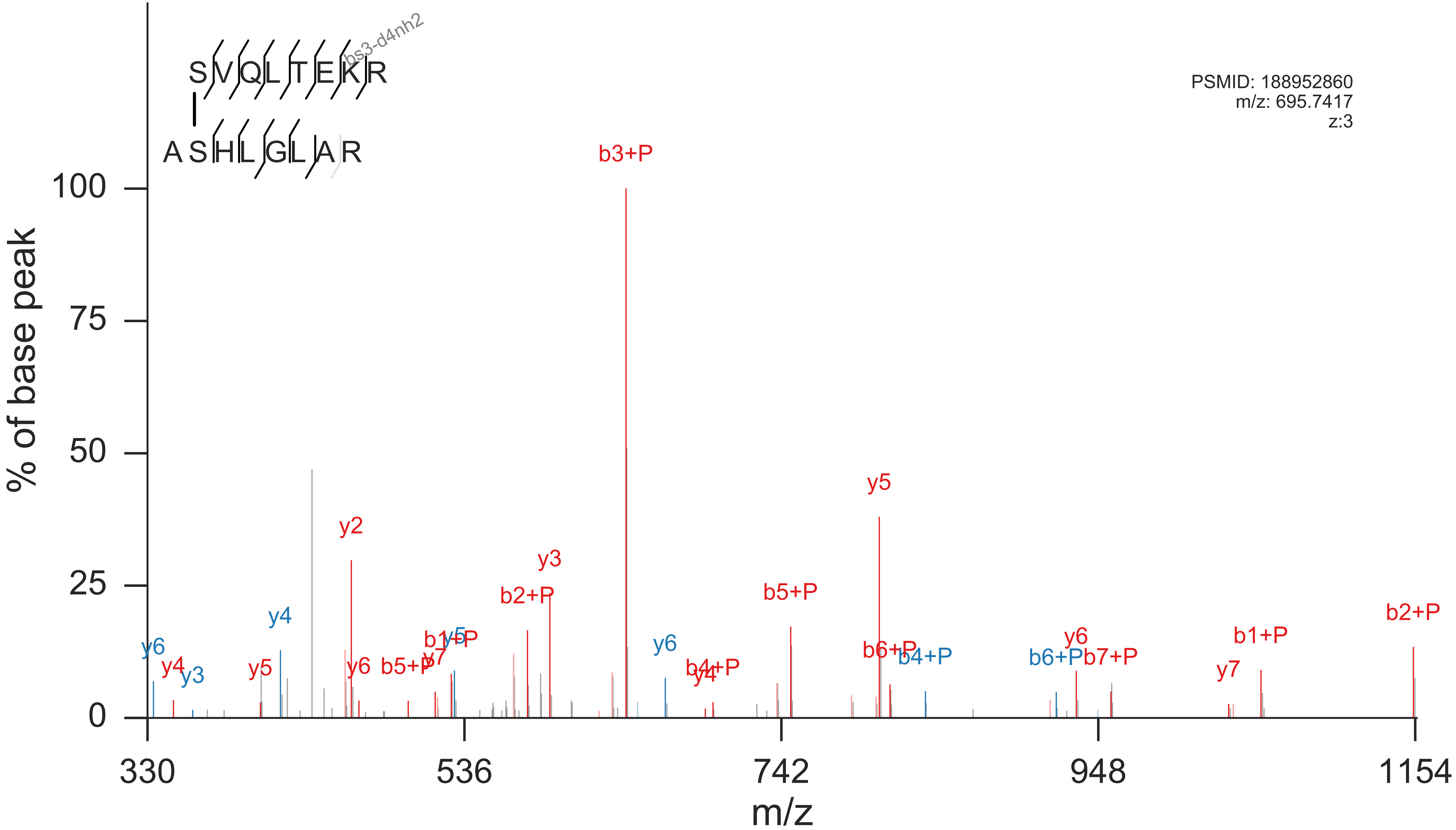

SVQLTEKR
ASHLGLAR
bs3-d4nh2
PSMID: 188952860
m/z: 695.7417
z:3
% of base peak
m/z
b3+P
y5
y2
y3
b2+P
b5+P
y4
y6
y3
y5
b1+P
y7
b5+P
b6+P
b4+P
b6+P
b7+P
y7
b1+P
b2+P

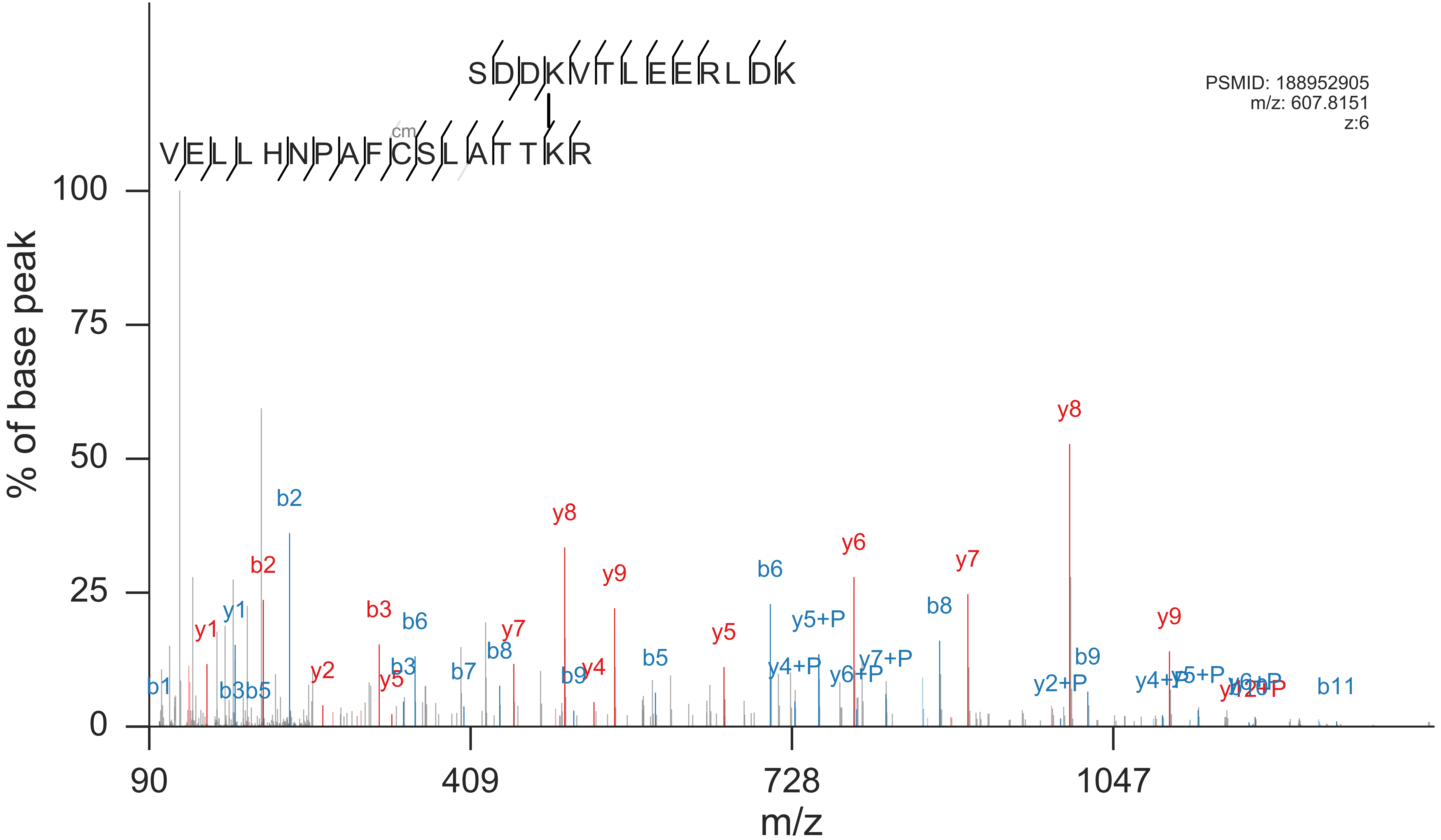
SDDKVTLEERLDK
VELLHNPAFCSLATTKR
cm
PSMID: 188952905
m/z: 607.8151
z:6
% of base peak
m/z
100
75
50
25
0
90
409
728
1047

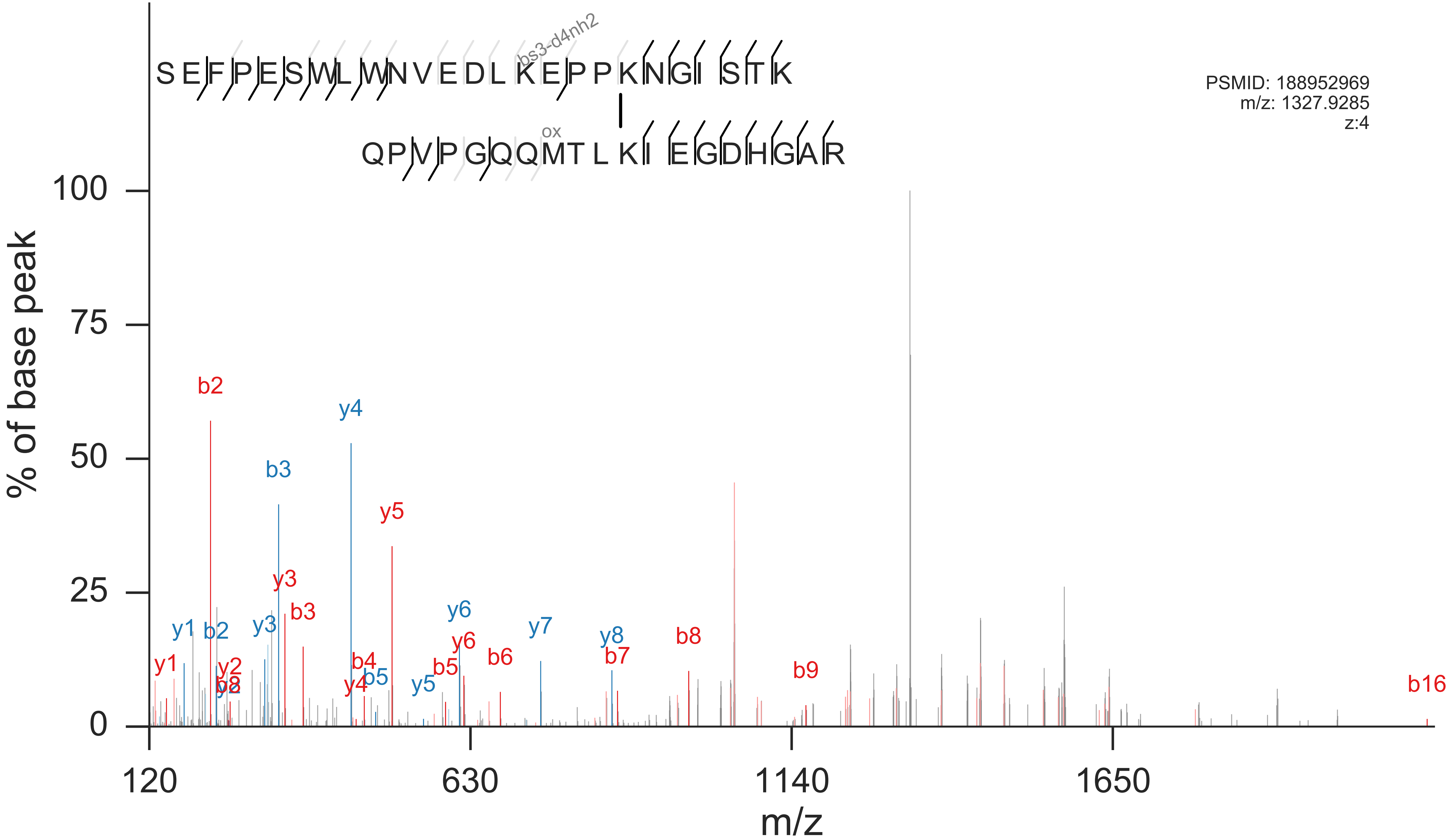

SEFPESWLWNVEDLKEPPKNGISTK
bs3-d4nh2
QPVPGQQMTLKIEGDHGAR
ox
PSMID: 188952969
m/z: 1327.9285
z:4
% of base peak
m/z
100
75
50
25
0
120
630
1140
1650
b2
y4
b3
y5
y3
b3
y1
b2
y3
y6
y6
y7
y8
b7
b8
b9
b16
y1
y2
b4
y4
b5
b5
y5
b6

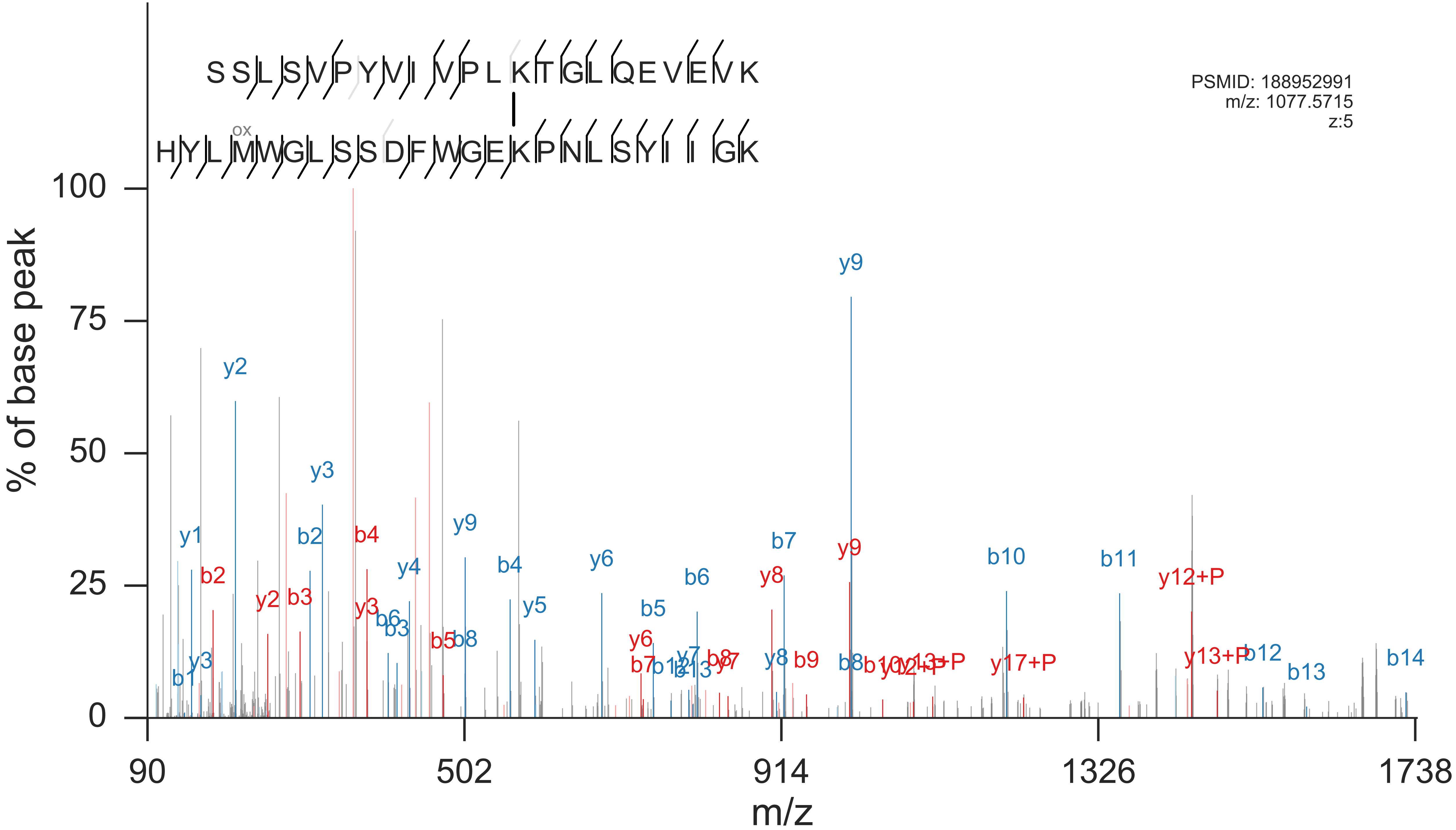

S S L S V P Y V I V P L K T G L Q E V E V K
H Y L M W G L S S D F W G E K P N L S Y I I G K
ox
PSMID: 188952991
m/z: 1077.5715
z:5
% of base peak
m/z
0
25
50
75
100
90
502
914
1326
1738

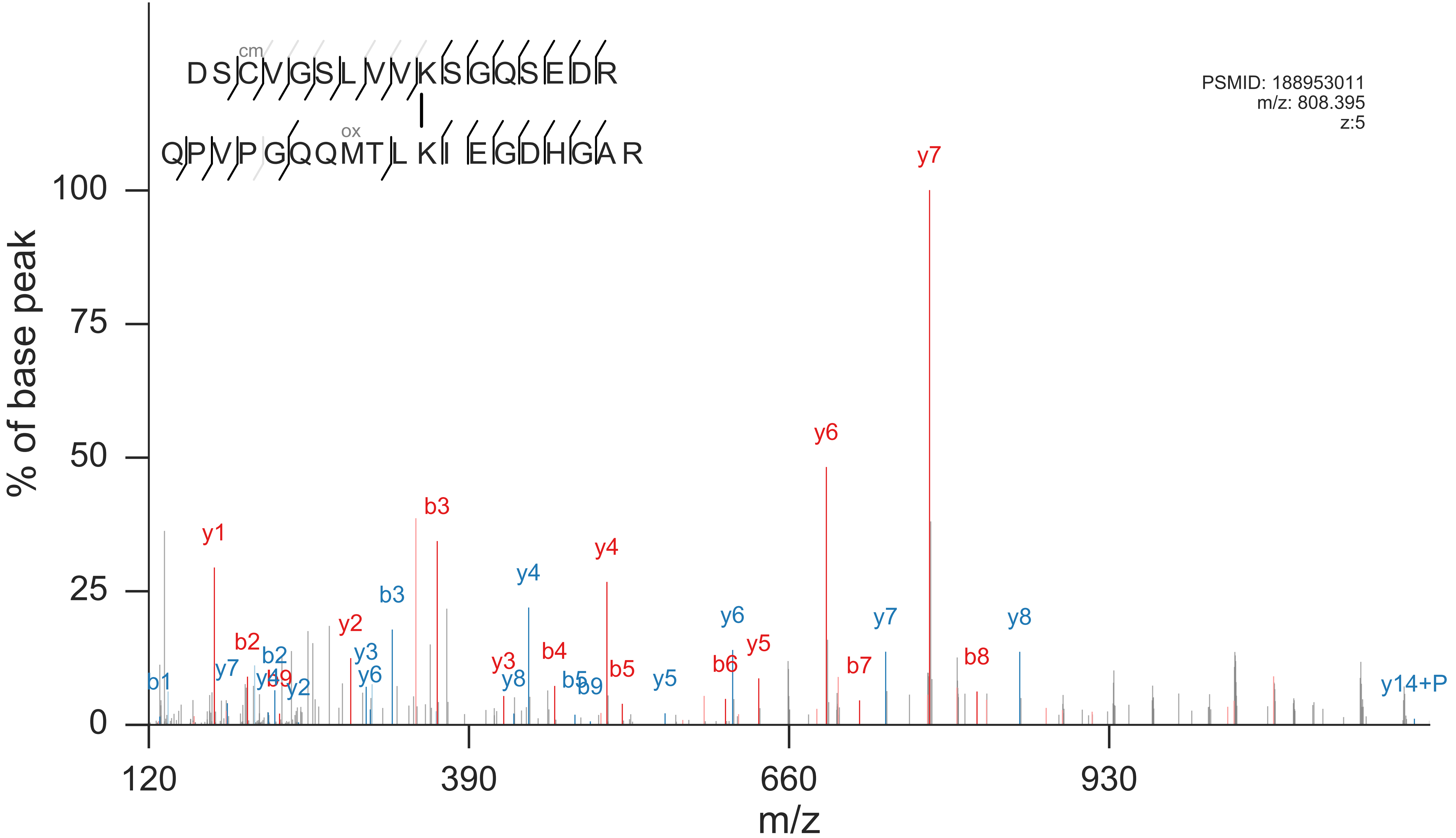

D S C V G S L V V K S G Q S E D R
cm
Q P V P G Q Q M T L K I E G D H G A R
ox
PSMID: 188953011
m/z: 808.395
z:5
% of base peak
m/z

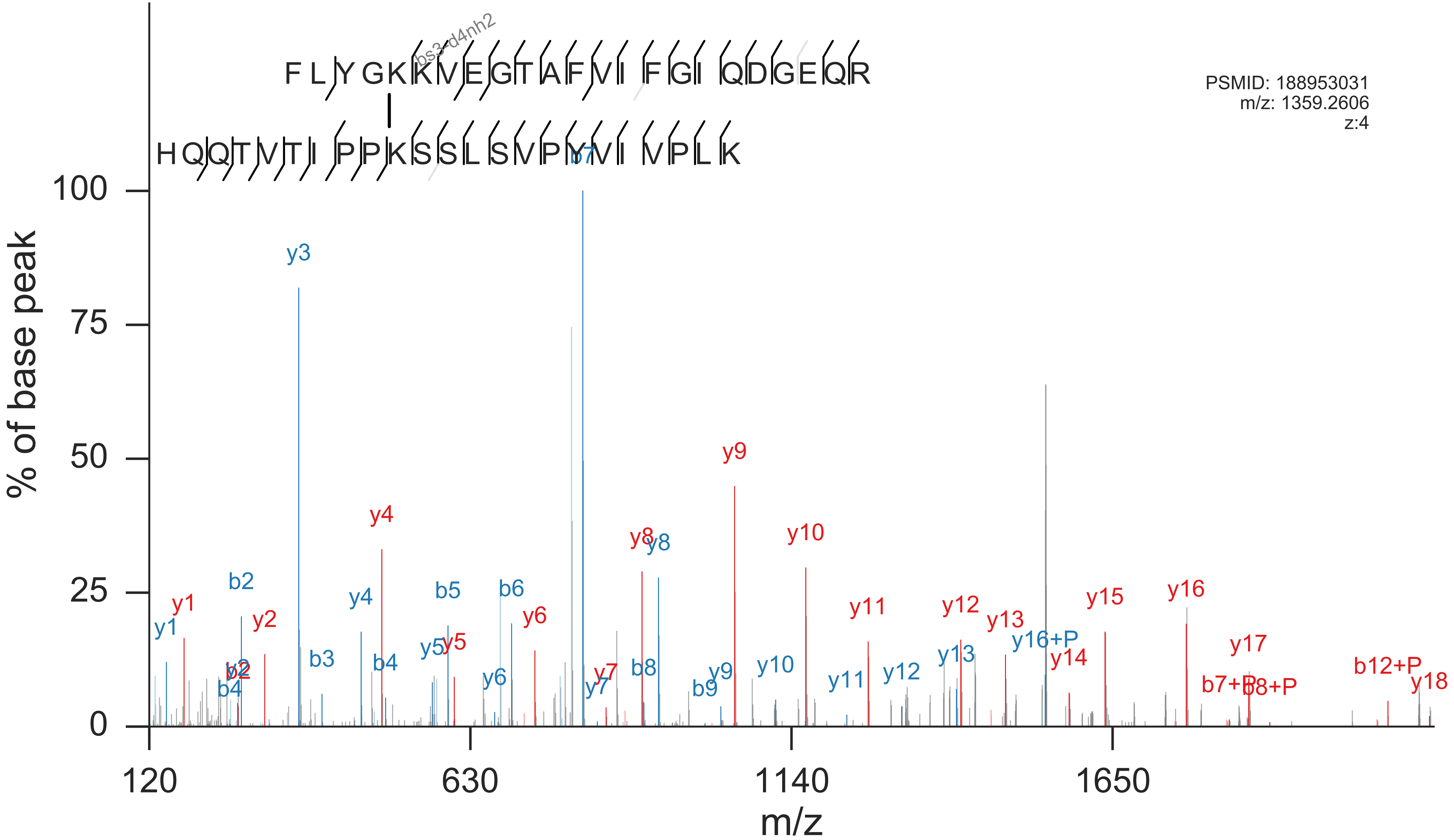

PSMID: 188953031
m/z: 1359.2606
z:4
FLYGKKVEGTAFVIFGIQDGEQR
HQQTVTIPPKSSLSVPYVIVPLK
bs3-d4nh2
% of base peak
m/z

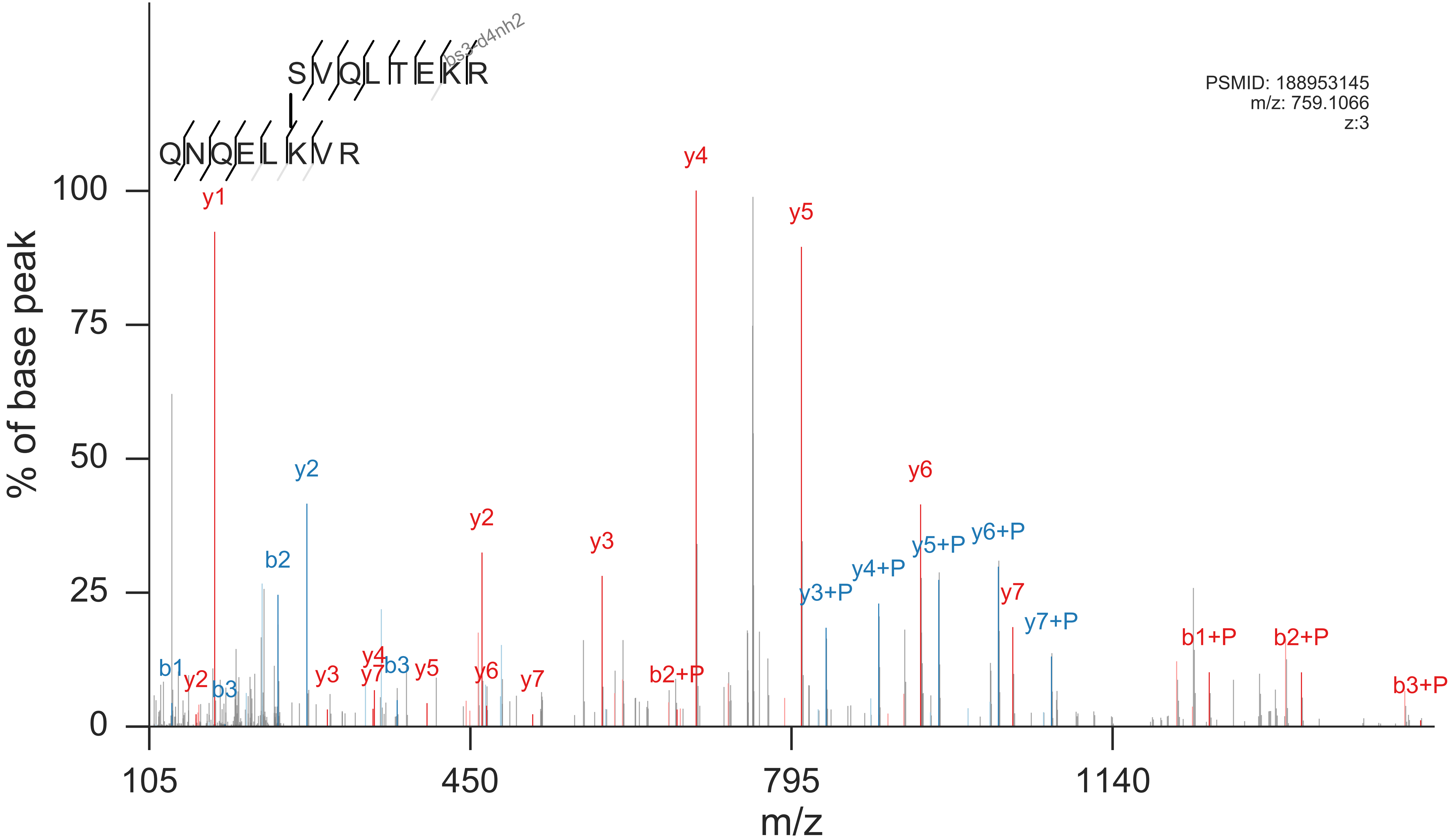

SVQLTEKR
bs3-d4nh2
QNQELKVR
PSMID: 188953145
m/z: 759.1066
z:3
% of base peak
m/z
100
75
50
25
0
105
450
795
1140
y1
y4
y5
y6
y2
b2
y2
y3
y7
y3+P
y4+P
y5+P
y6+P
y7+P
b1+P
b2+P
b3+P
b1
y2
b3
y3
y4
y7
b3
y5
y6
y7
b2+P

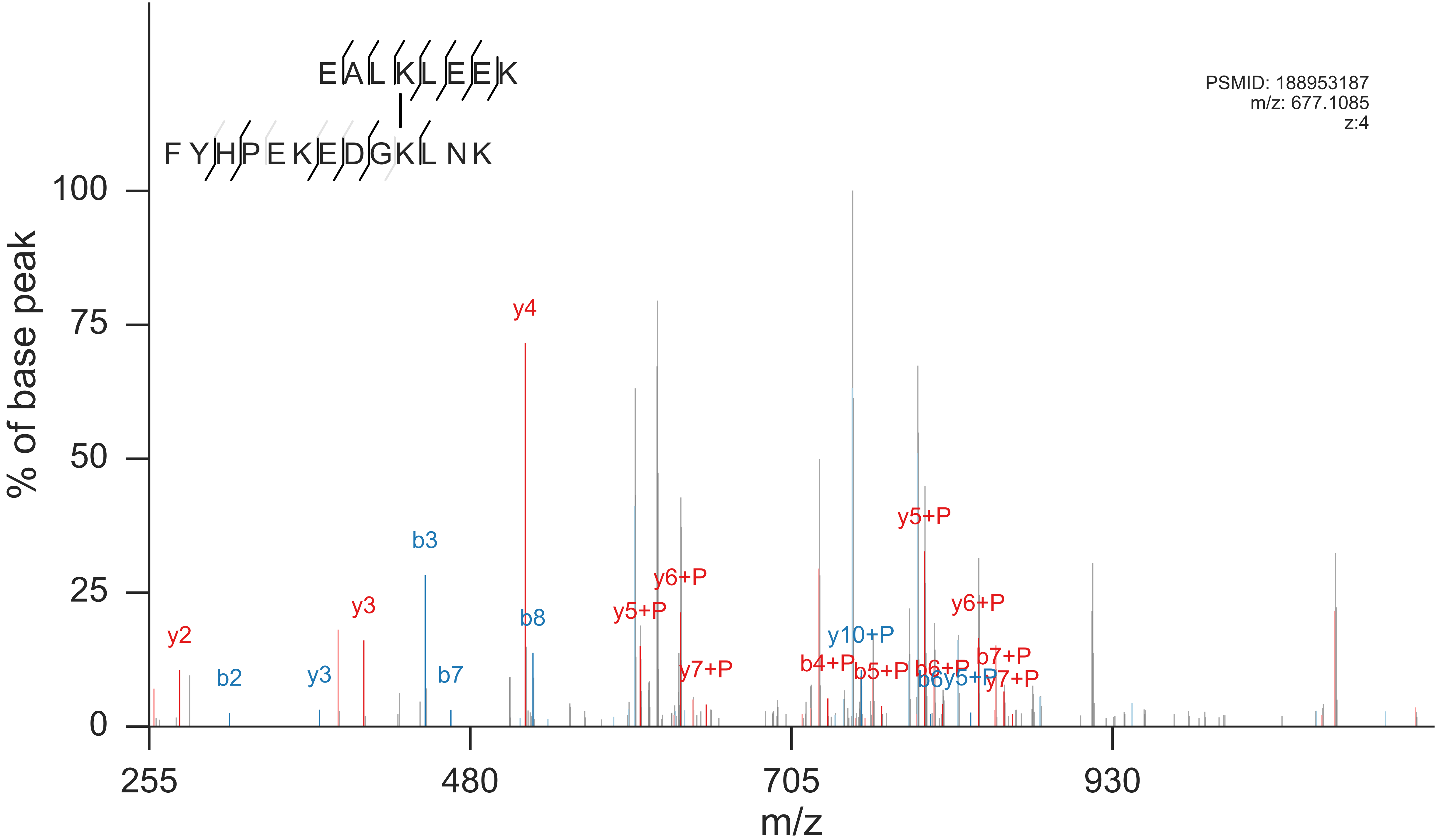

EALKLEEK
FYHPEKEDGKLNK
PSMID: 188953187
m/z: 677.1085
z:4
% of base peak
100
75
50
25
0
255
480
705
930
m/z
y2
b2
y3
y3
b3
b7
y4
b8
y5+P
y6+P
y7+P
y10+P
b4+P
b5+P
y5+P
b6+P
b6
y5+P
y6+P
b7+P
y7+P

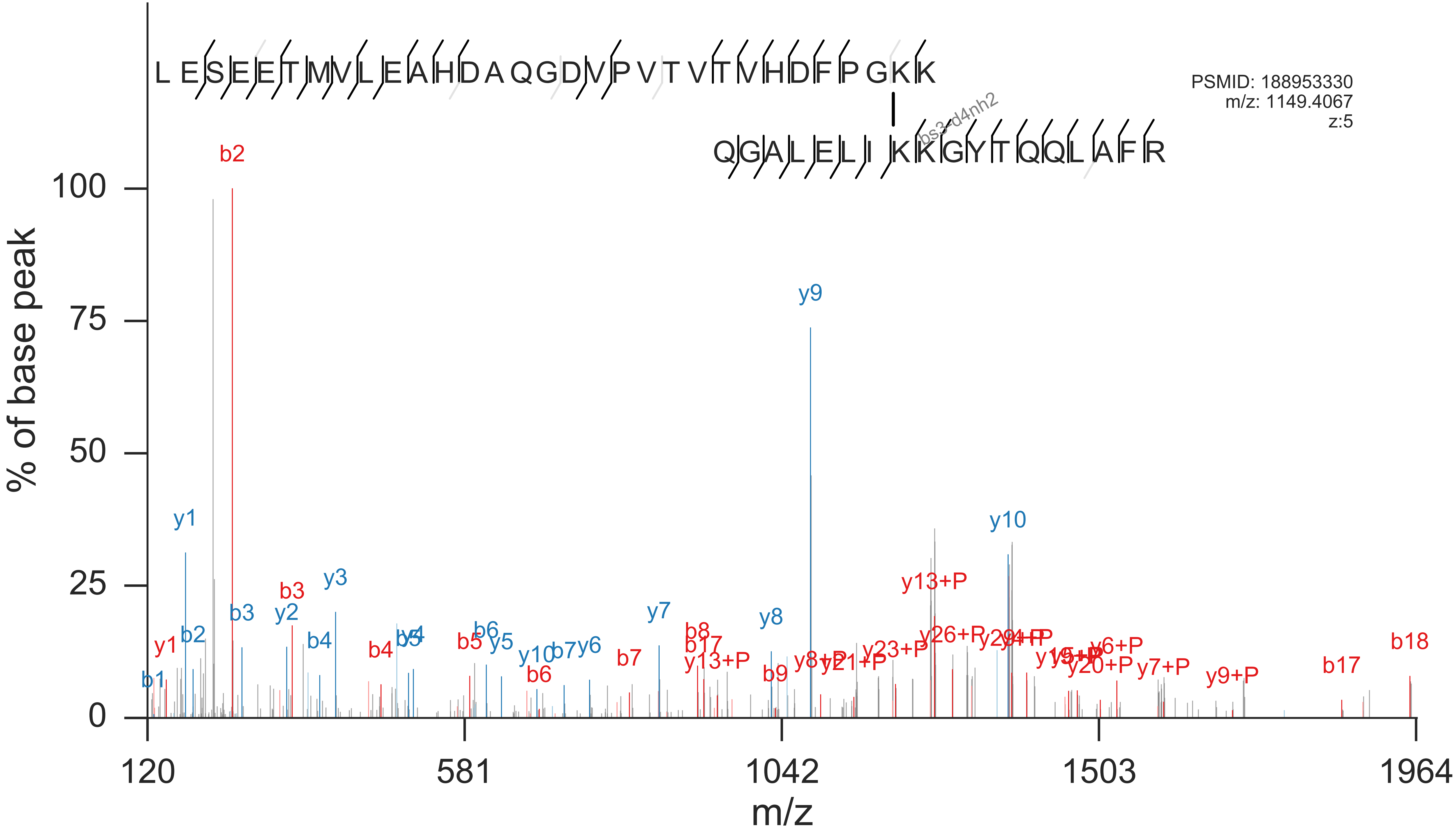

PSMID: 188953330
m/z: 1149.4067
z:5
LESEETMVLEAHDAQGDVPVTVTVHDFPGKK
QGALELIKKGYTQQLAFR
bs3-d4nh2
% of base peak
m/z

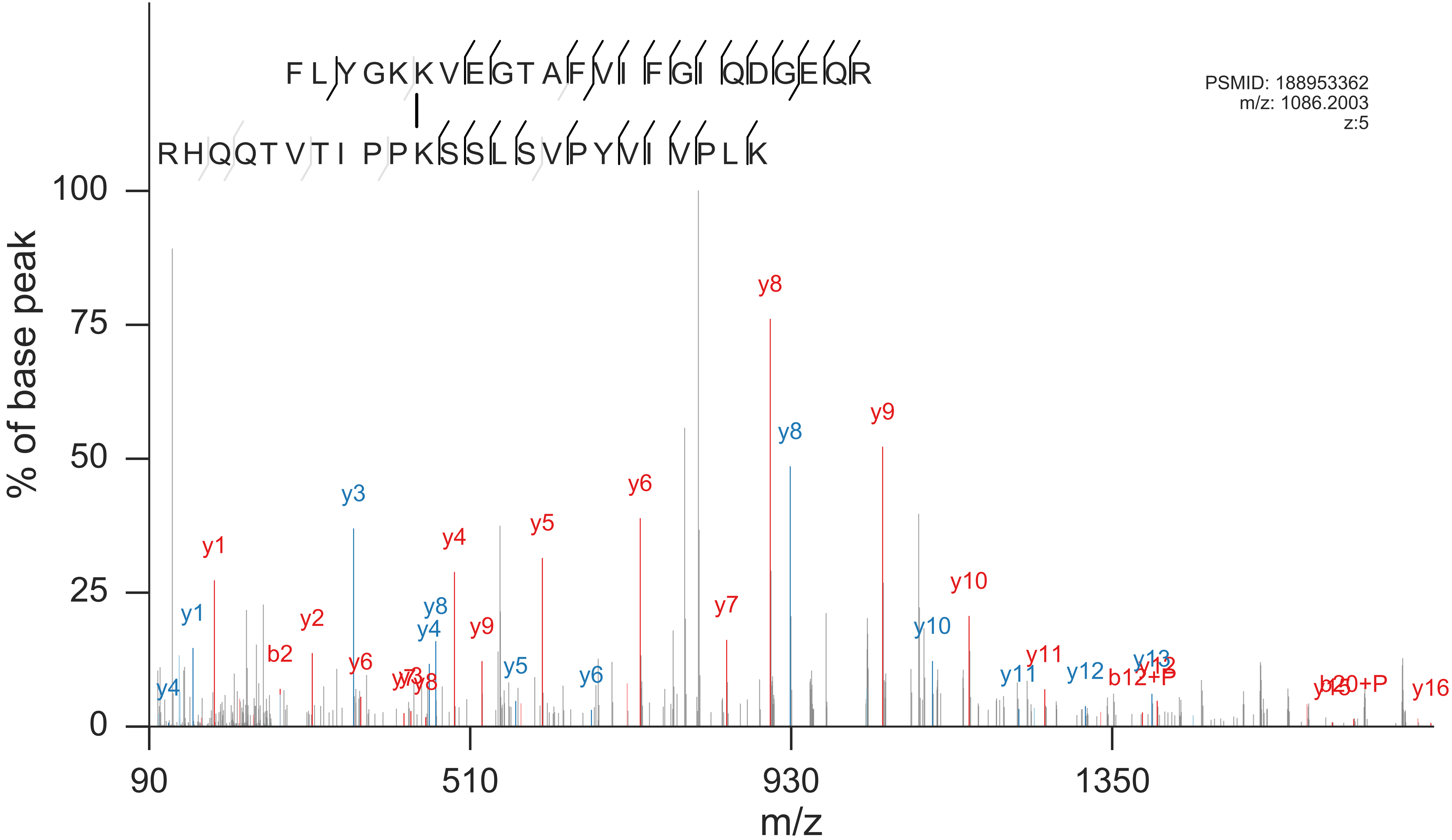
FLYGKKVEGTAFVIFGIQDGEQR
RHQQTVTIPPKSSLSVPYVIVPLK
PSMID: 188953362
m/z: 1086.2003
z:5
% of base peak
m/z
100
75
50
25
0
90
510
930
1350

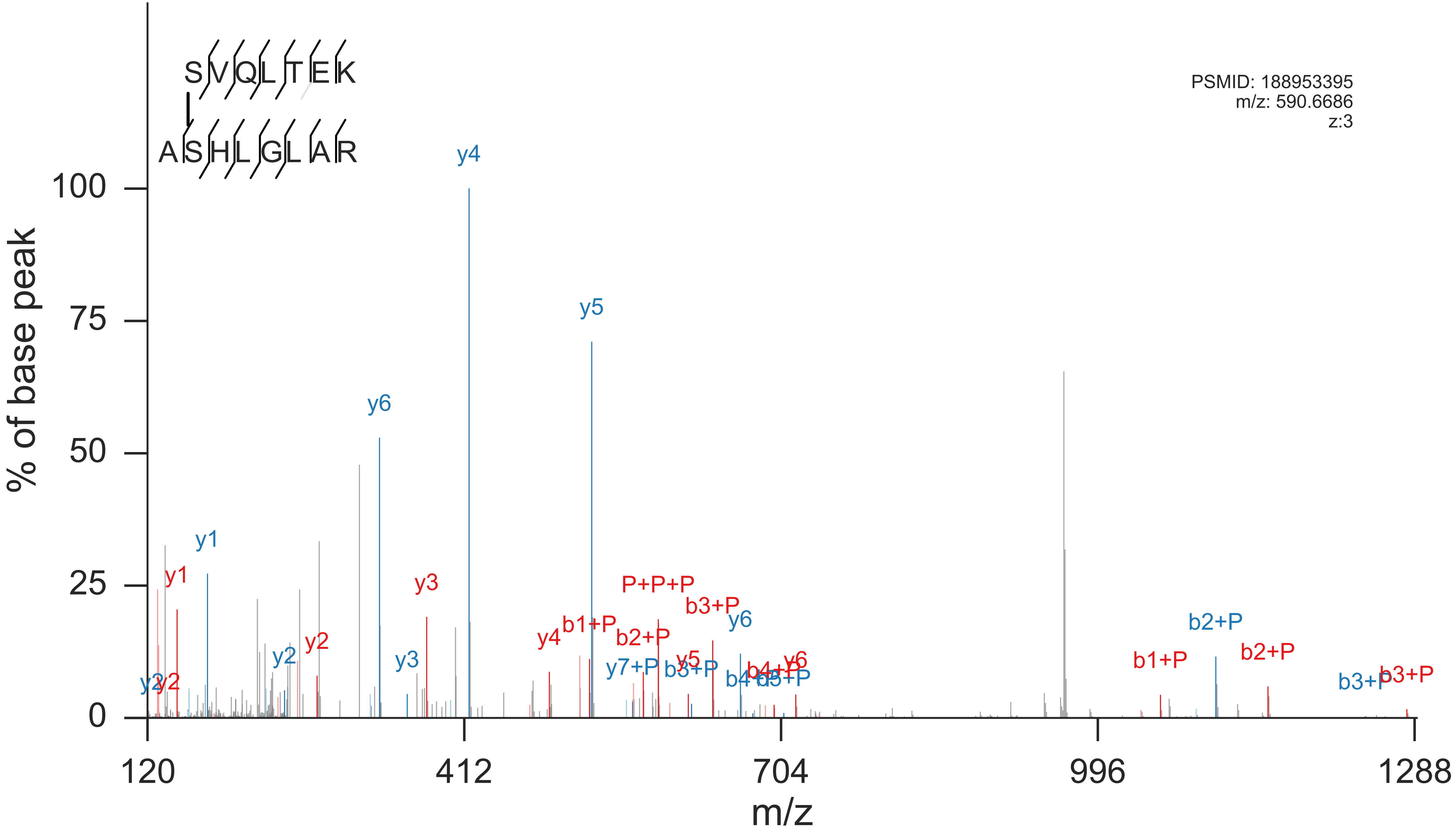

SVQLTEK
ASHLGLAR
PSMID: 188953395
m/z: 590.6686
z:3
% of base peak
m/z
y4
y5
y6
y1
y1
y3
y2
y3
y2
y2
y2
P+P+P
b3+P
y6
b1+P
b2+P
y4
y5
y7+P
b3+P
b4+P
b5+P
y6
b4+P
b2+P
b1+P
b2+P
b3+P
b3+P
0
25
50
75
100
120
412
704
996
1288

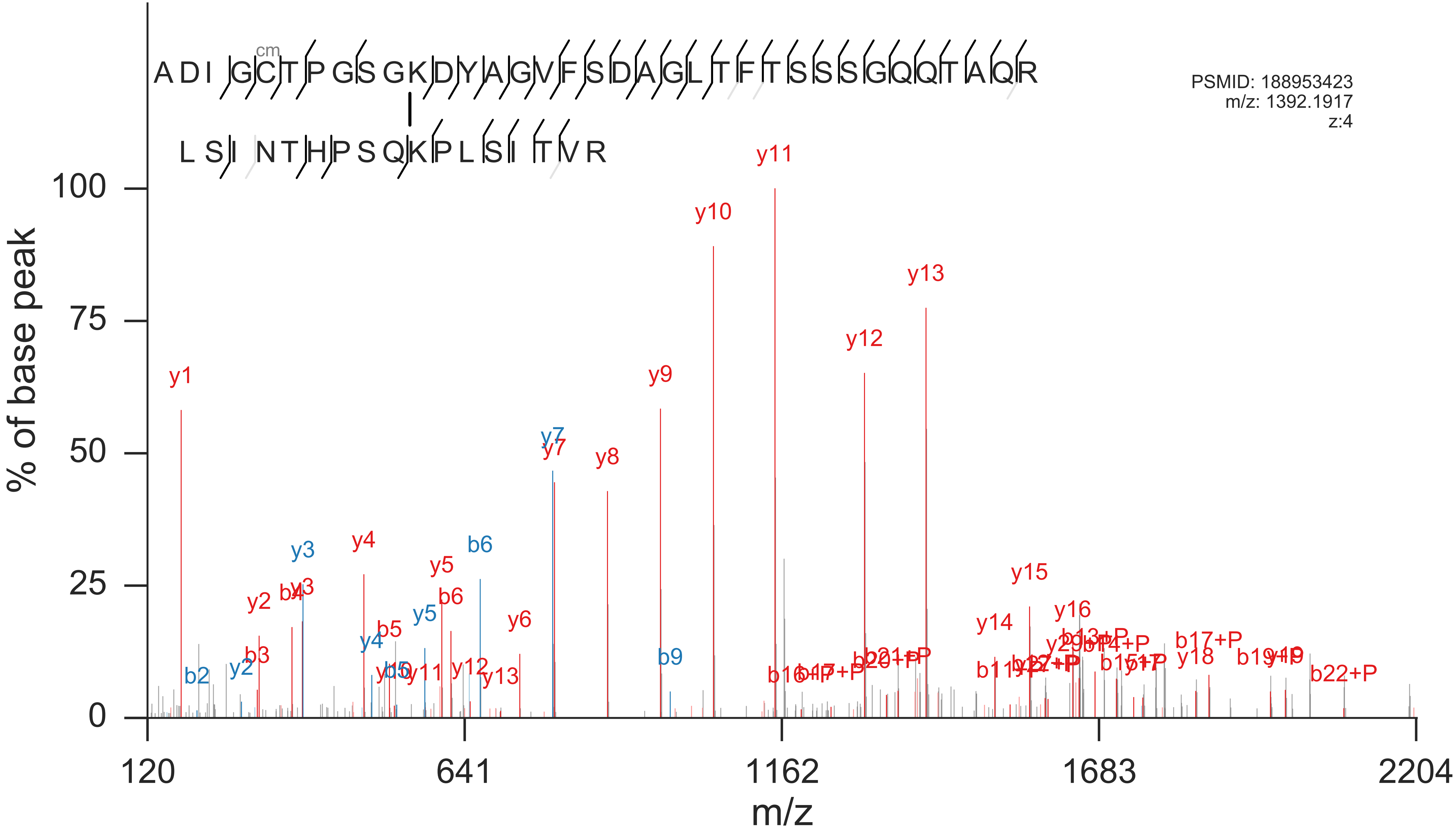

ADIGCTPGSGKDYAGVFSDAGLTFTSSSGQQTAQR
cm
LSINTHPSQKPLSITVR
PSMID: 188953423
m/z: 1392.1917
z:4
% of base peak
m/z
0
25
50
75
100
120
641
1162
1683
2204
y1
y2
y3
y4
y5
y6
y7
y8
y9
y10
y11
y12
y13
y14
y15
y16
y18
b2
b3
b4
b5
b6
b9
b17+P
b22+P

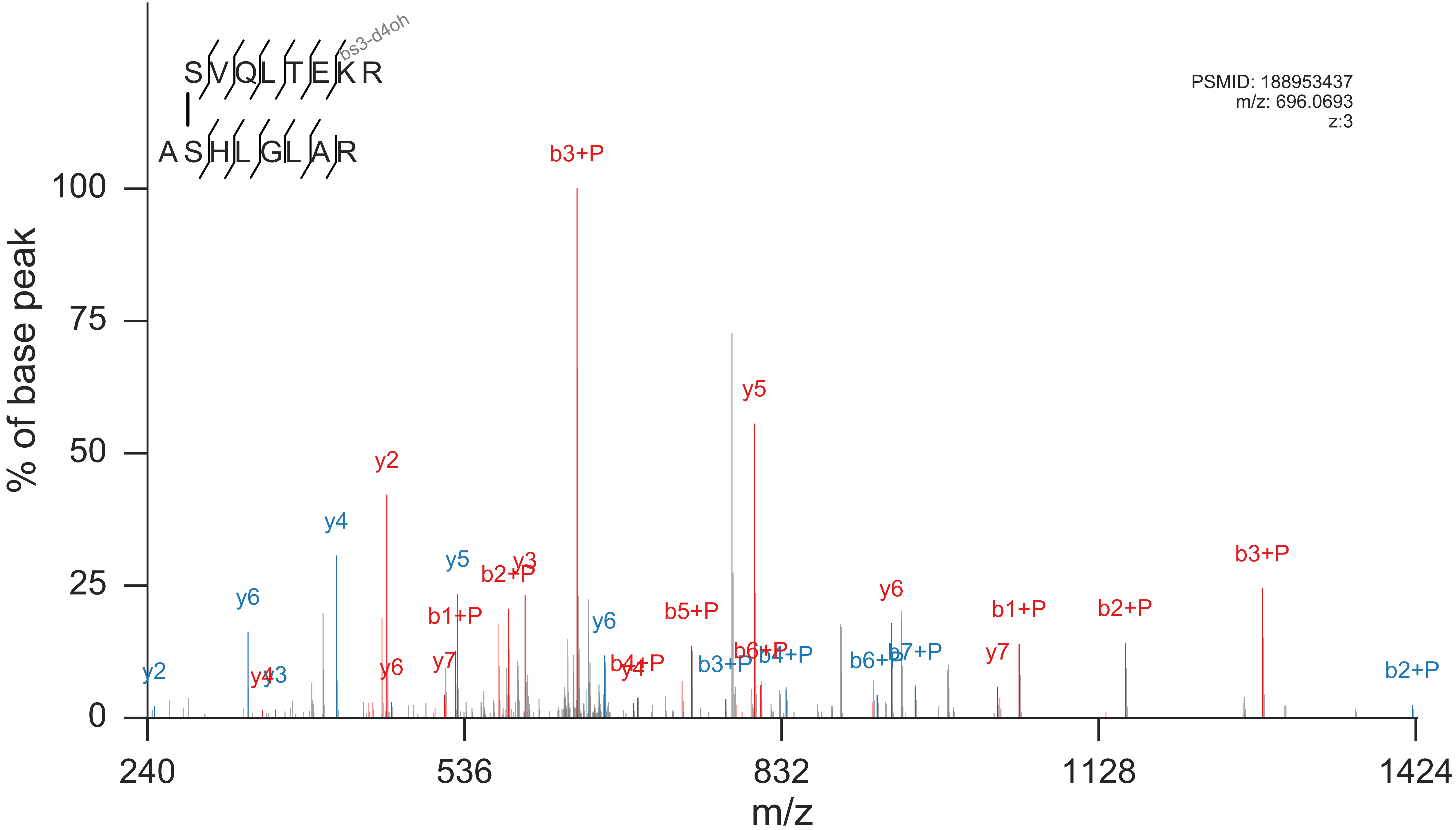

SVQLTEKR
bs3-d4oh
ASHLGLAR
PSMID: 188953437
m/z: 696.0693
z:3
% of base peak
m/z
0
25
50
75
100
240
536
832
1128
1424
b3+P
y5
y2
y4
y5
b2+P
y3
y6
b1+P
y6
b5+P
y6
b1+P
b2+P
b3+P
y2
y4
y3
y6
y7
b4+P
y4
b3+P
b6+P
b4+P
b6+P
b7+P
y7
b2+P

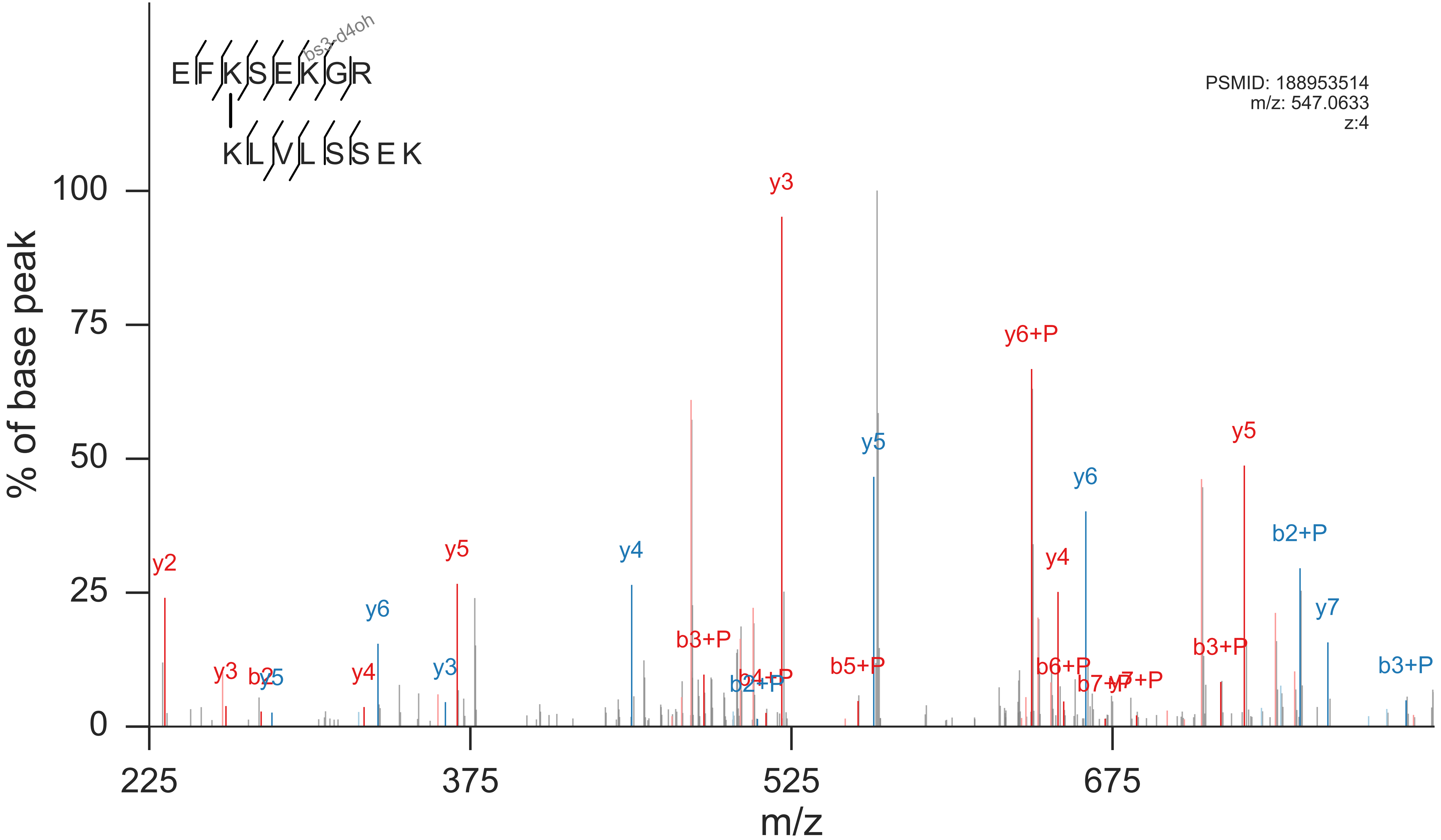

EFKSEKGR
bs3-d4oh
KLVLSSEK
PSMID: 188953514
m/z: 547.0633
z:4
% of base peak
m/z
y2
y3
b2
y5
y4
y6
y3
y5
y4
b3+P
b2+P
b4+P
y3
b5+P
y5
y6+P
b6+P
y4
y6
b7+P
y7+P
b3+P
y5
b2+P
y7
b3+P

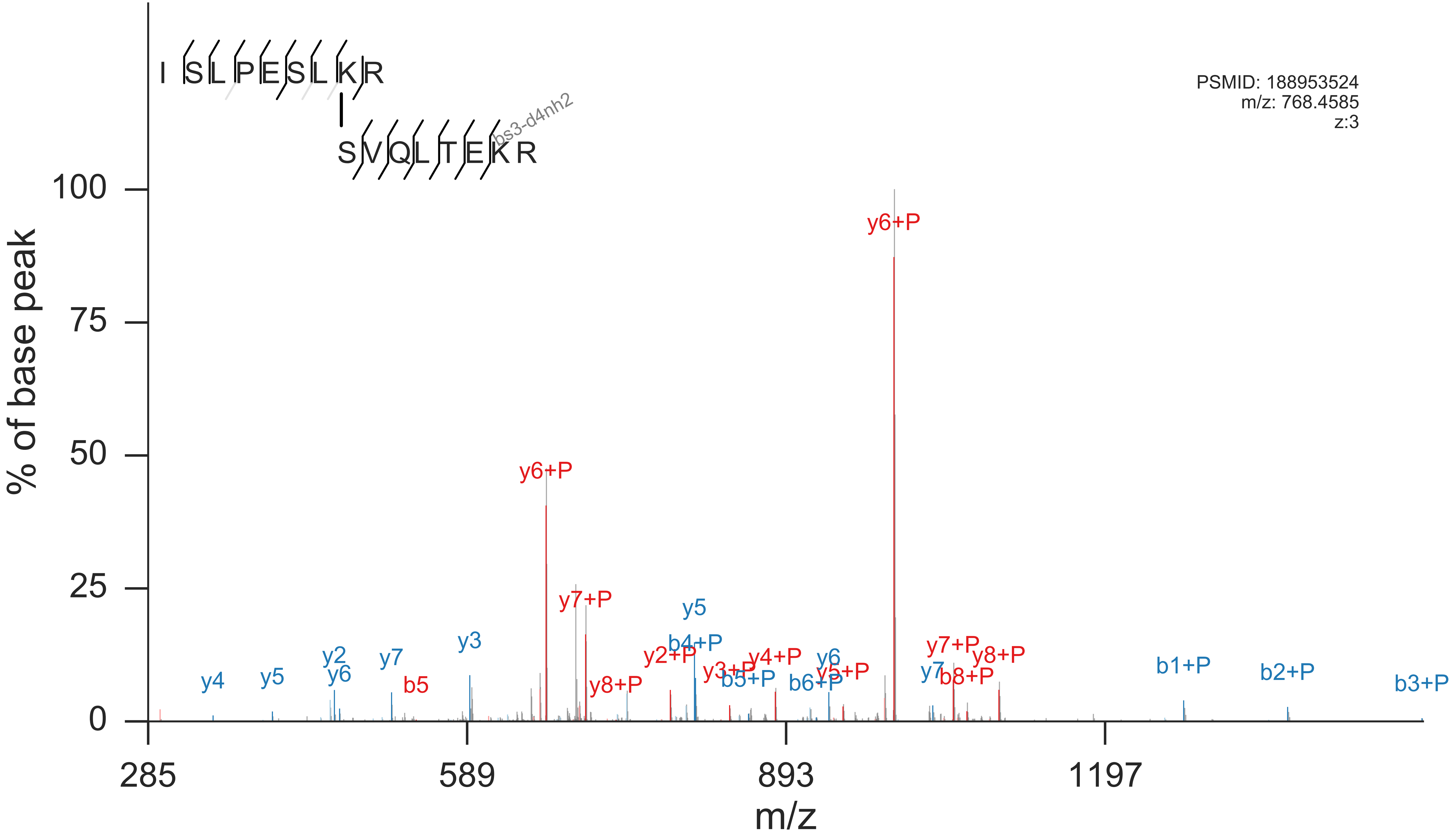

I SLPESLKR
SVQLTEKR
bs3-d4nh2
PSMID: 188953524
m/z: 768.4585
z:3
% of base peak
m/z
0
25
50
75
100
285
589
893
1197
y6+P
y6+P
y7+P
y5
b4+P
y2+P
y3+P
y4+P
y6
b5+P
b6+P
y5+P
y7+P
y8+P
y7
b8+P
y8+P
b1+P
b2+P
b3+P
y3
y2
y6
y7
y4
y5
b5

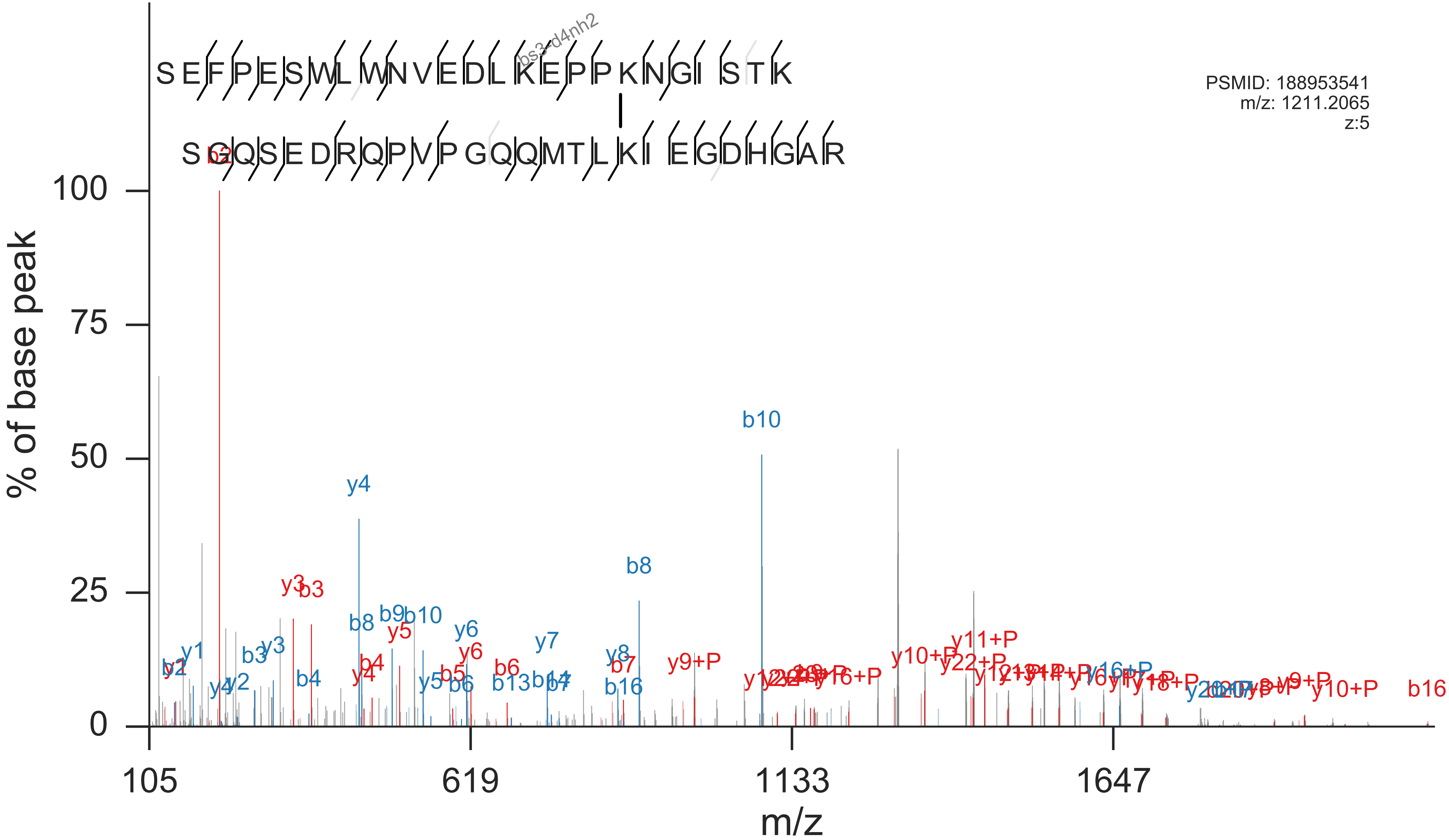

SEFPESWLWNVEDLKEPPKNGISTK
SGQSEDRQPVPGQQMTLKIEGDHGAR
bs3-d4nh2
PSMID: 188953541
m/z: 1211.2065
z:5
% of base peak
m/z

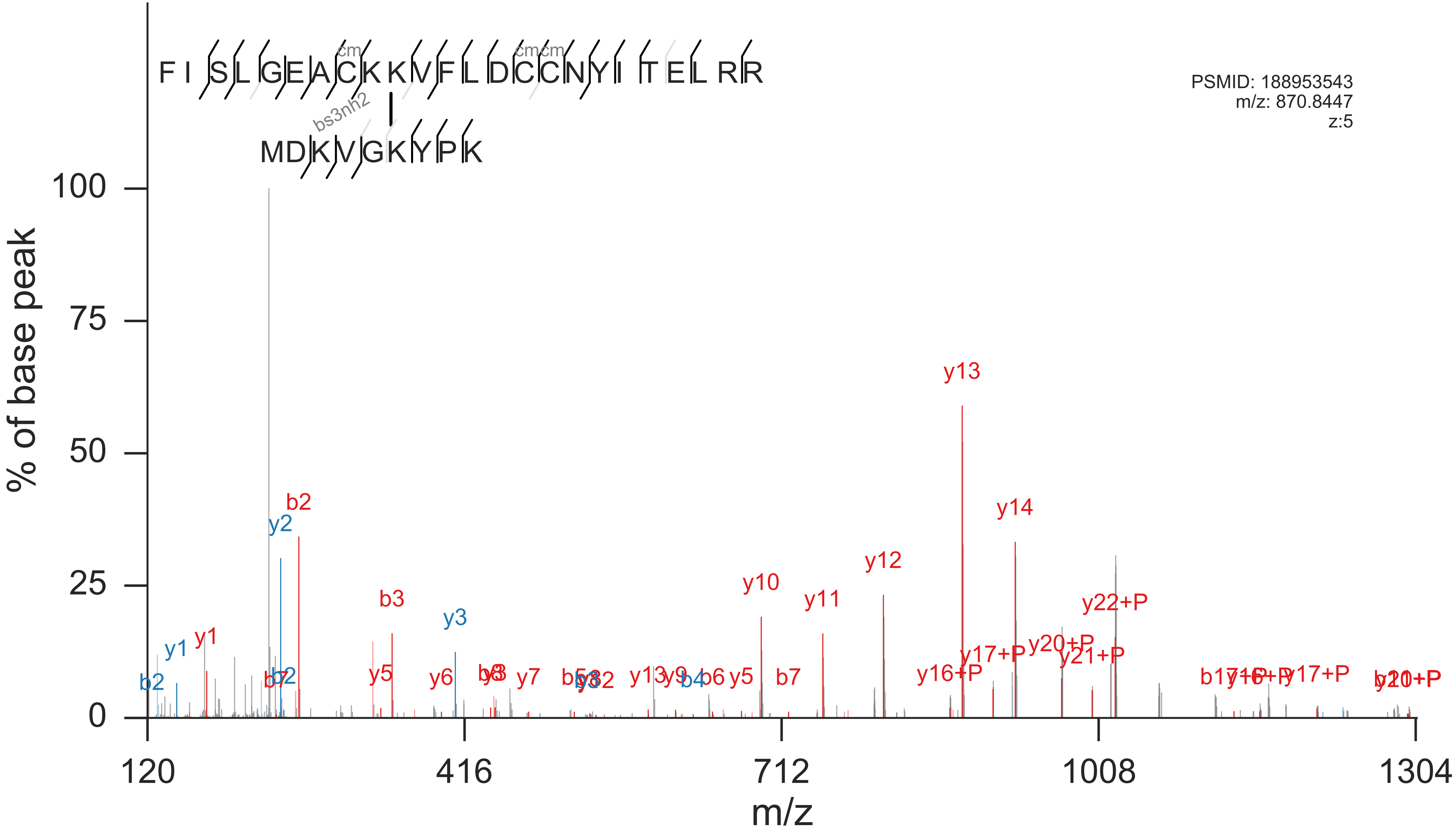
FISLGEACKKVFLDCCNYITELRR
MDKVGKYPK
cm
cmcm
bs3nh2
PSMID: 188953543
m/z: 870.8447
z:5
% of base peak
m/z
y13
y14
y12
y10
y11
b2
y2
b3
y3
y22+P
y20+P
y17+P
y21+P
y16+P
y1
y5
y6
y7
b7
y5
b6
y17+P
0
25
50
75
100
120
416
712
1008
1304

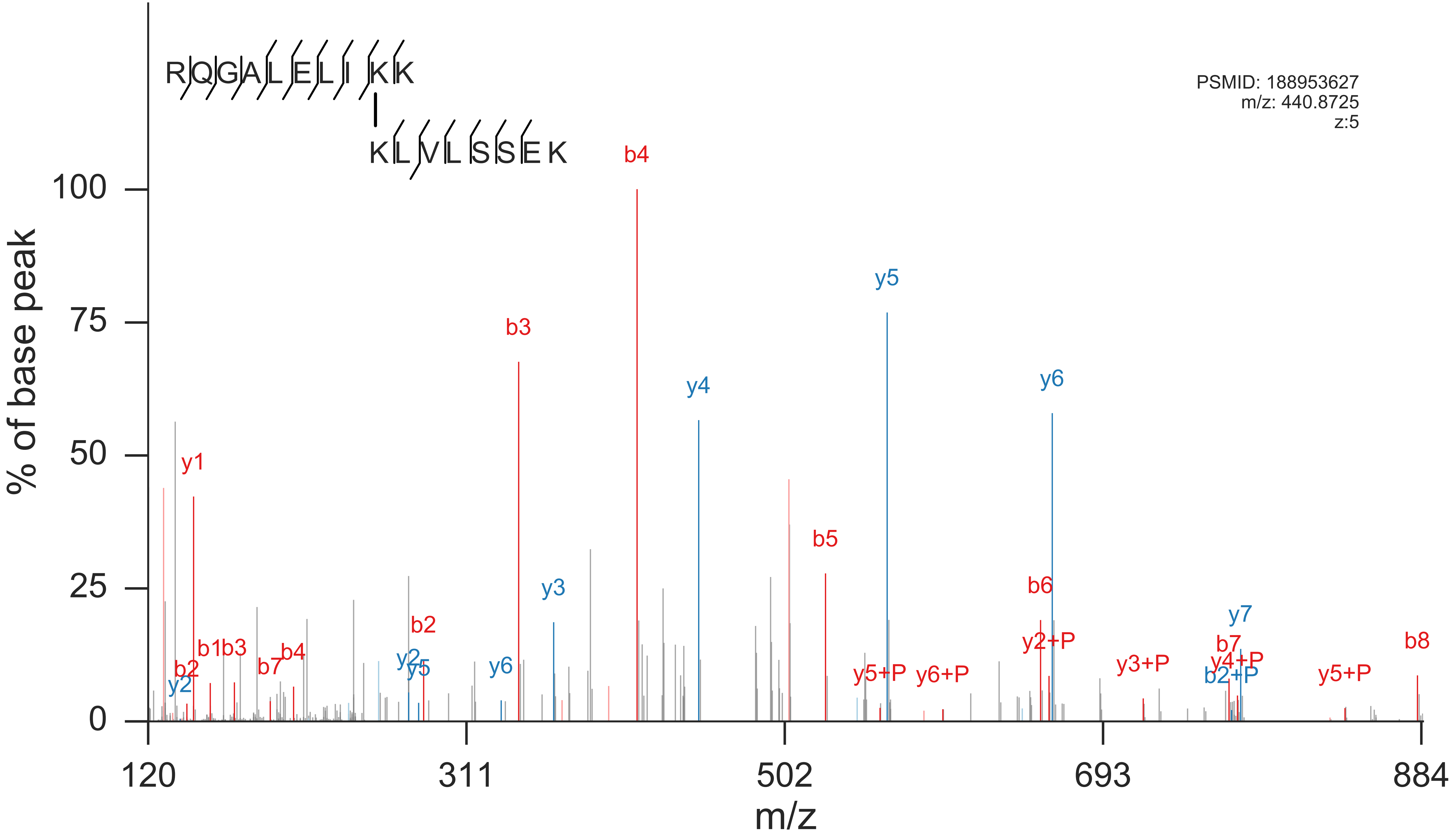
RQGALELIKK
KLVLSSEK
PSMID: 188953627
m/z: 440.8725
z:5
% of base peak
m/z
b4
b3
y5
y4
y6
y1
b5
y3
b6
b2
y2+P
y7
b7
b8
y4+P
b2+P
y3+P
y5+P
y6+P
y6
y2
y5
b1b3
b7
b4
b2
y2
y5+P

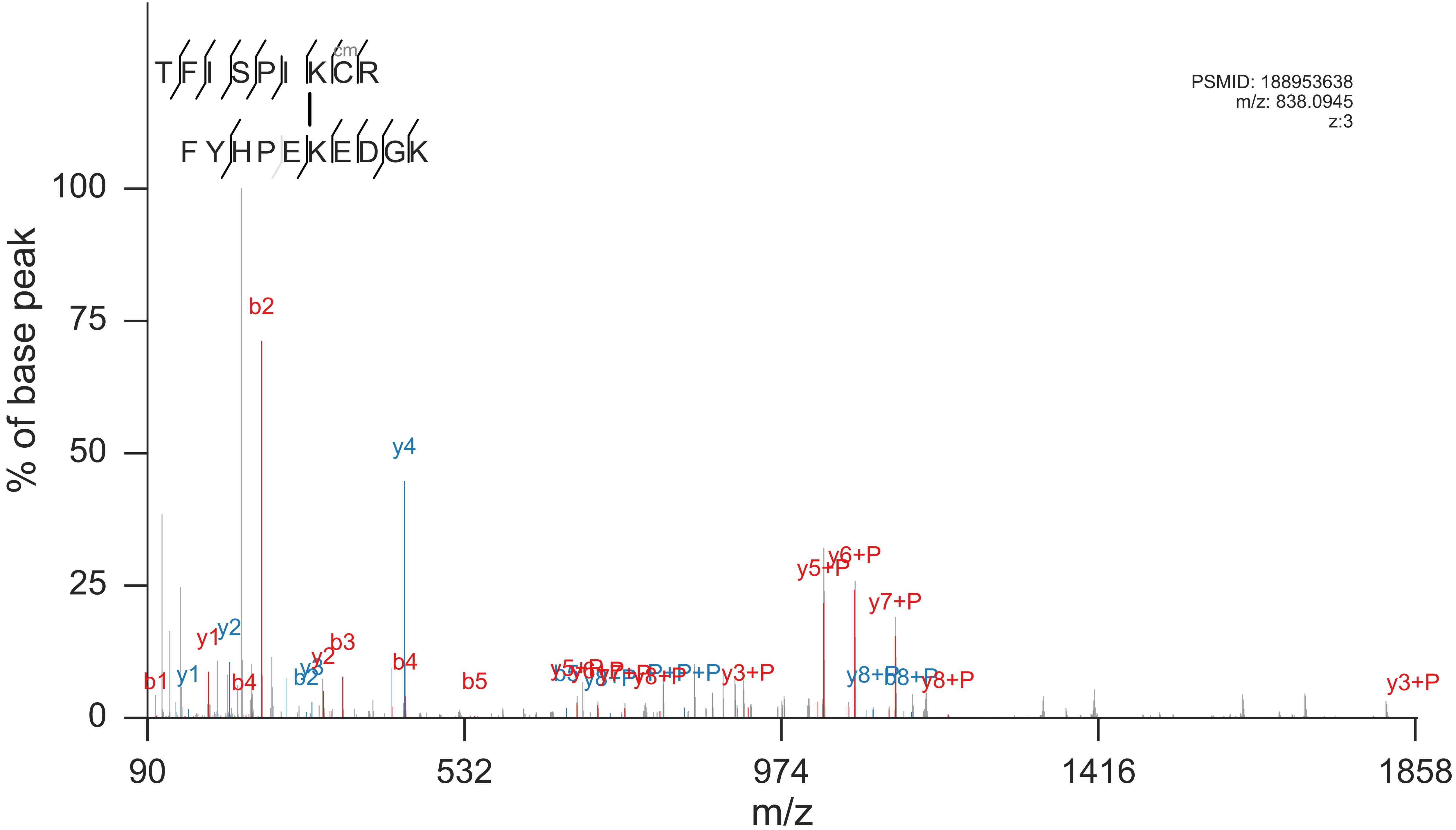

PSMID: 188953638
m/z: 838.0945
z:3
TFISPIKCR
cm
FYHPEKEDGK
% of base peak
100
75
50
25
0
m/z
90
532
974
1416
1858
b2
y4
y5+P
y6+P
y7+P
y1
y2
b3
b4
b1
b5
y3+P
y8+P

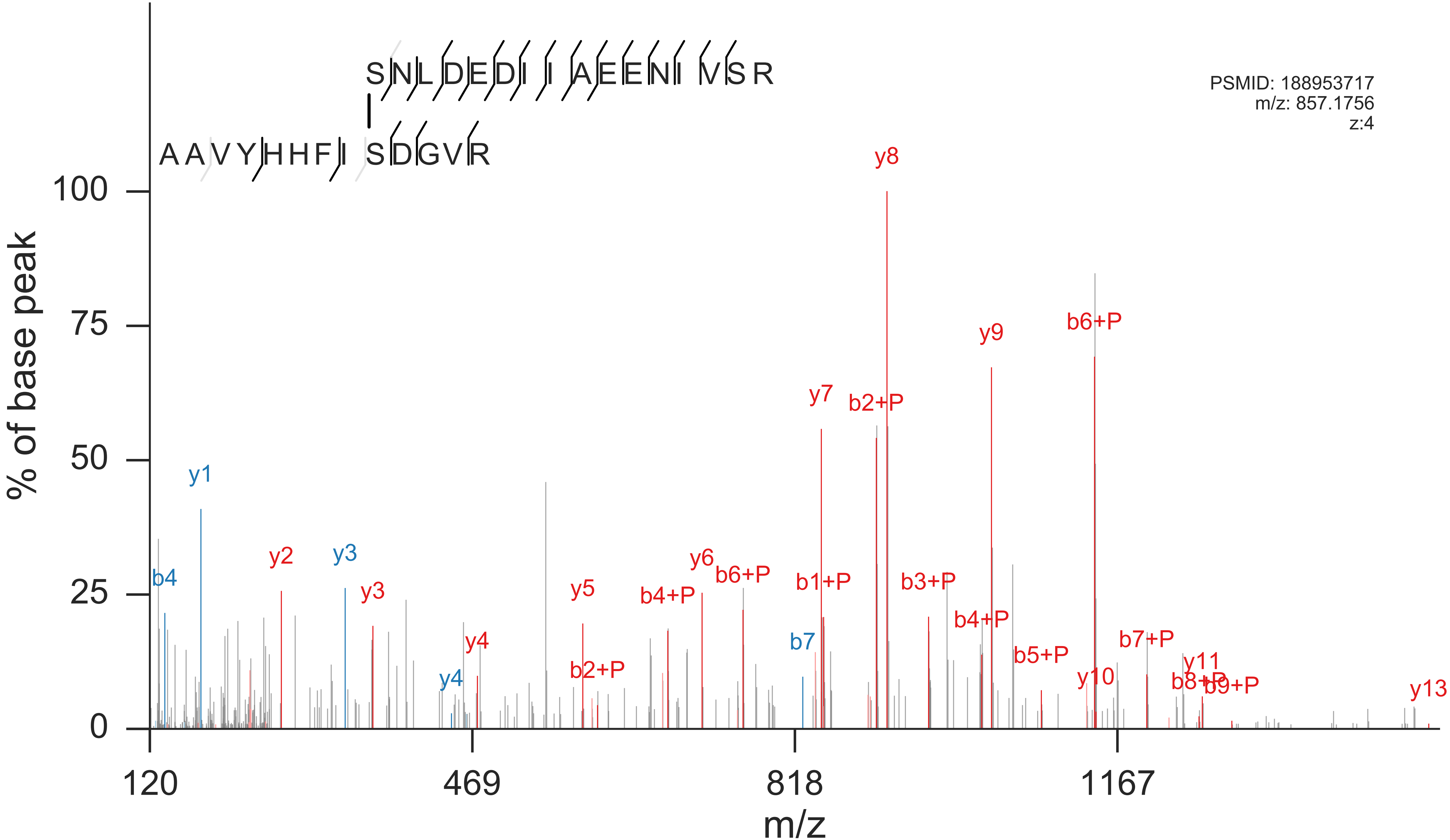

SNLDEDIIAEENIVSR
AAVYHHFISDGVR
PSMID: 188953717
m/z: 857.1756
z:4
% of base peak
m/z
y1
b4
y2
y3
y3
y4
y4
y5
b2+P
b4+P
y6
b6+P
b1+P
b7
y7
b2+P
y8
b3+P
b4+P
y9
b5+P
b6+P
y10
b7+P
y11
b8+P
b9+P
y13

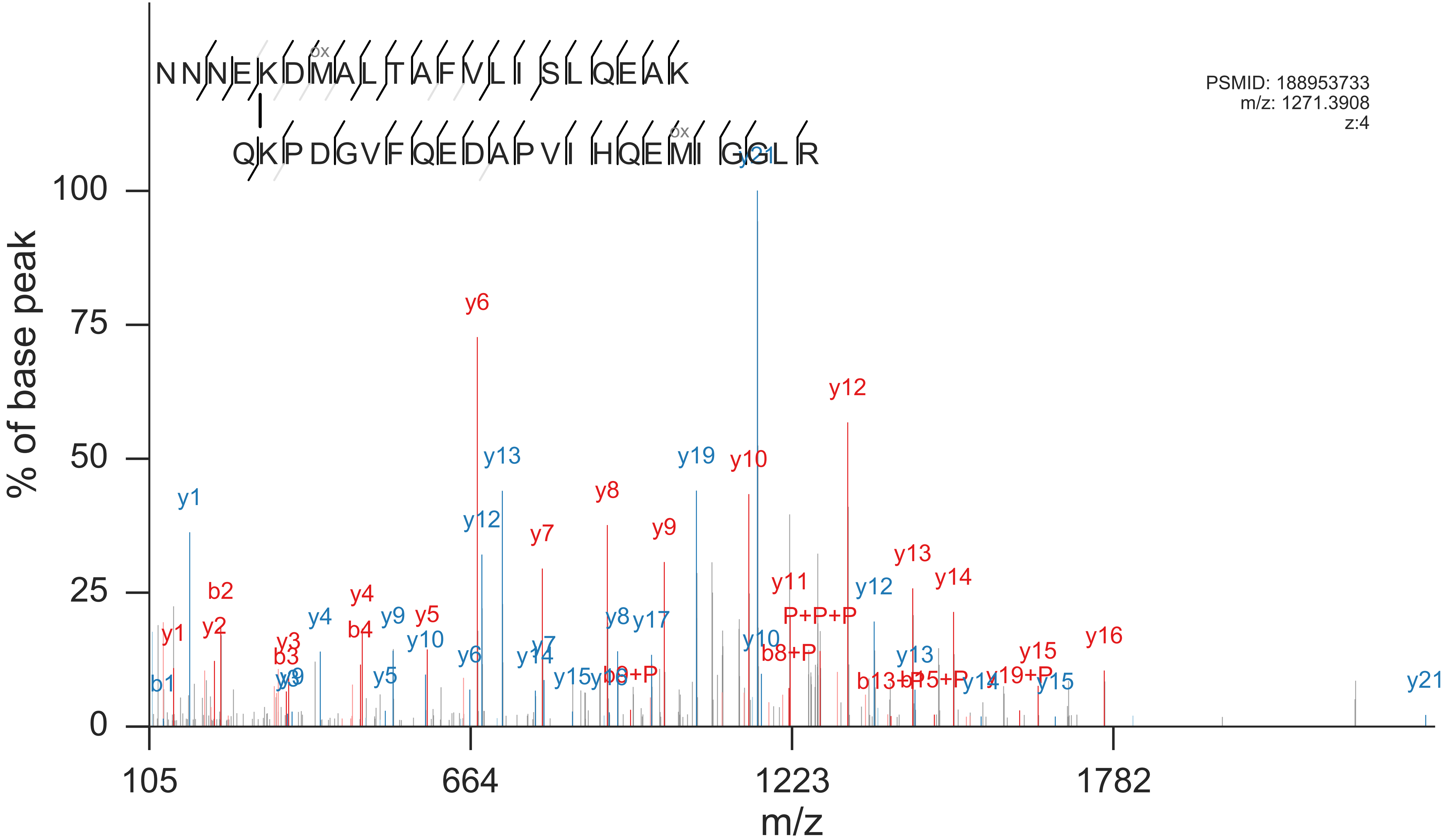

NNNEKDMALTAFVLISLQEAK
QKPDGVFQEDAPVIHQEMIGGLR
ox
PSMID: 188953733
m/z: 1271.3908
z:4
% of base peak
m/z
0
25
50
75
100
105
664
1223
1782
y6
y12
y13
y19
y10
y1
y8
y9
y7
y11
y13
y14
y21
y16
y15
P+P+P
b8+P
b2
y4
y5
b4

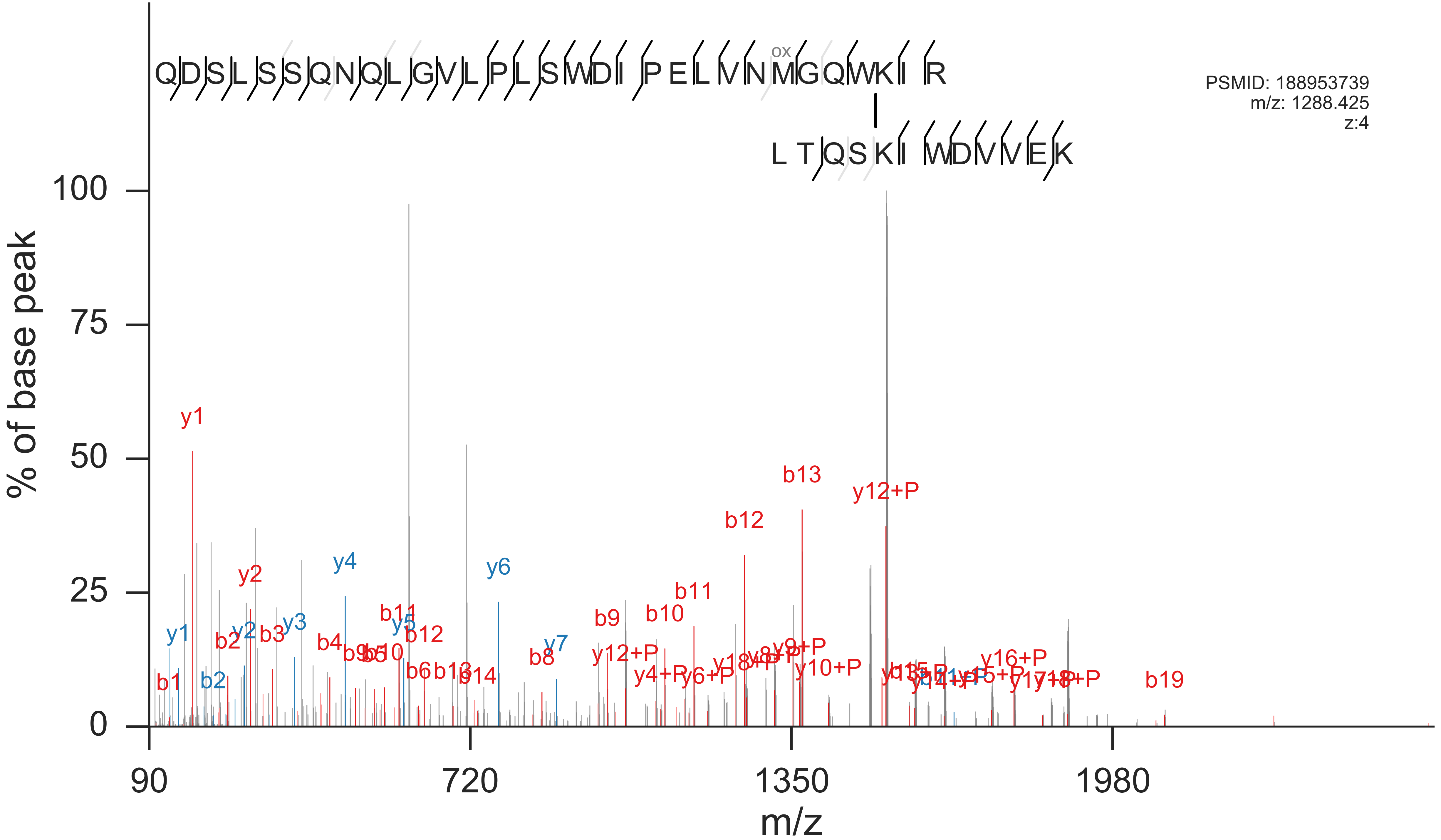
QDSLSSQNQLGVLPLSWDIPELVNMGQWKIR
ox
LTQSKIWDVVEK
PSMID: 188953739
m/z: 1288.425
z:4
% of base peak
m/z
100
75
50
25
0
90
720
1350
1980
y1
b13
y12+P
b12
y2
y4
y6
b11
b10
b9
y3
y1
b2
b1
b19

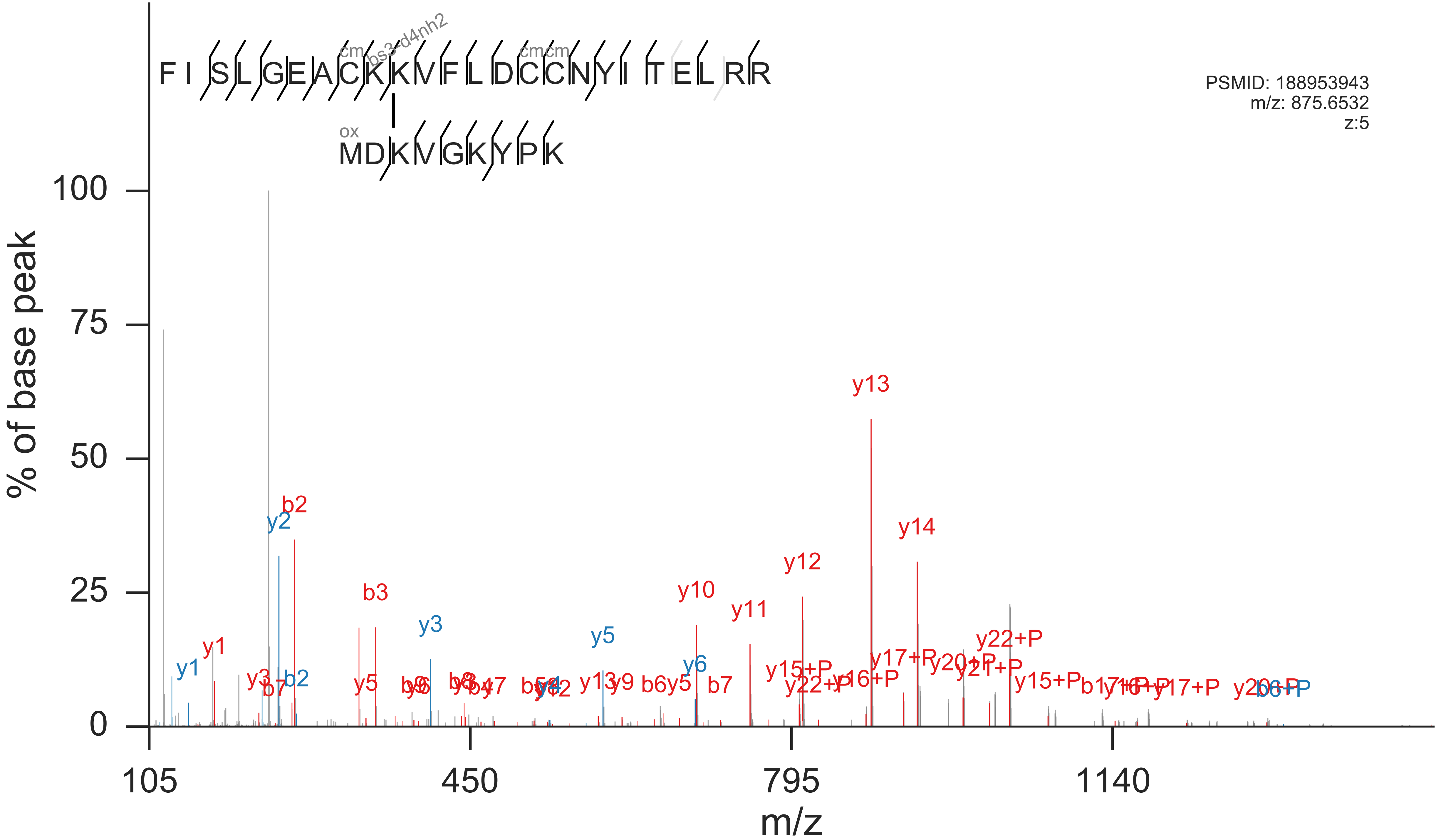

PSMID: 188953943
m/z: 875.6532
z:5
% of base peak
m/z
y13
y14
y12
y10
y11
b2
y2
b3
y3
y5
y6
y1
y22+P
y17+P
y15+P

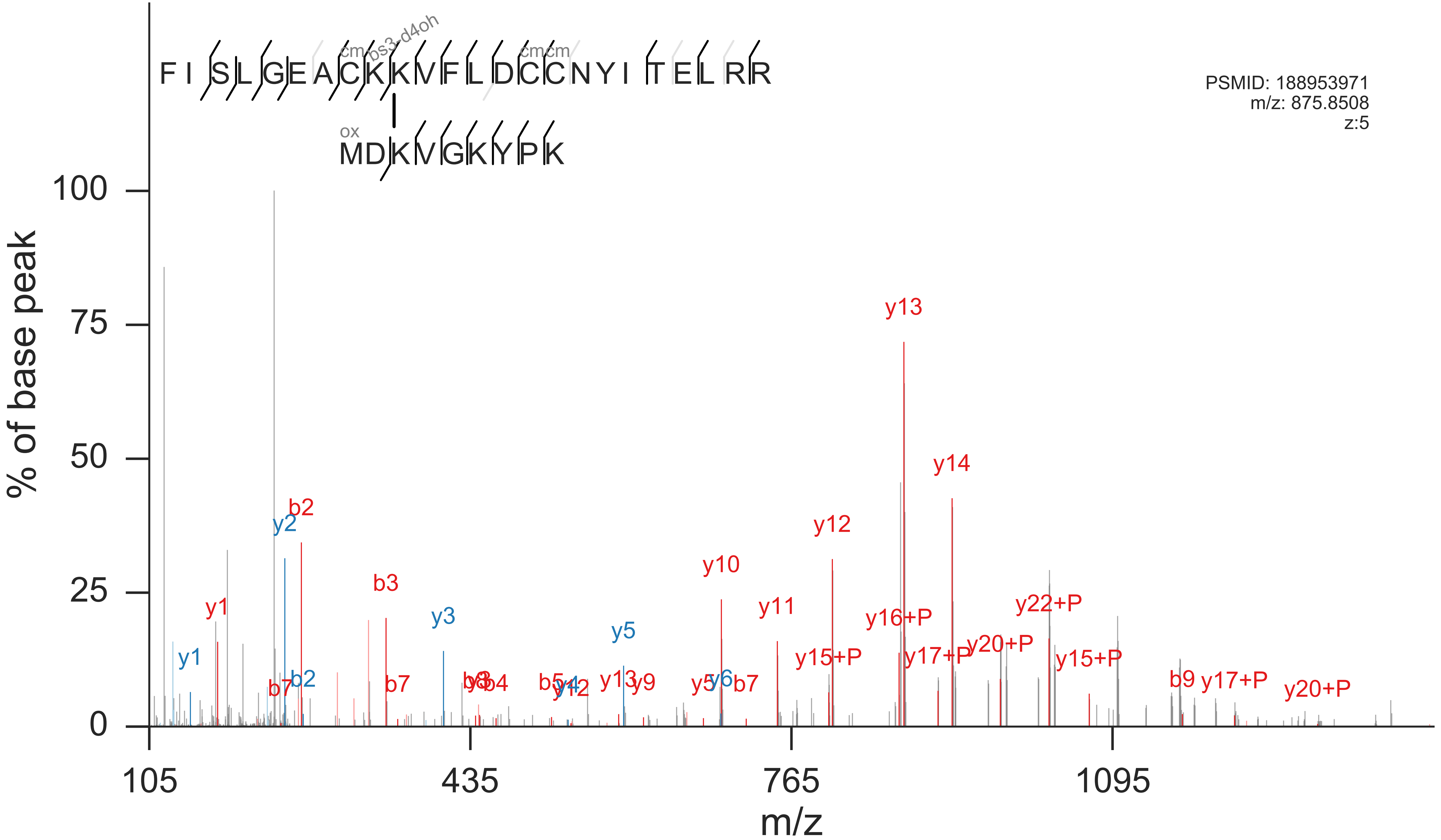

PSMID: 188953971
m/z: 875.8508
z:5
FISLGEACKKVFLDCCNYITELRR
MDKVGKYPK
% of base peak
m/z

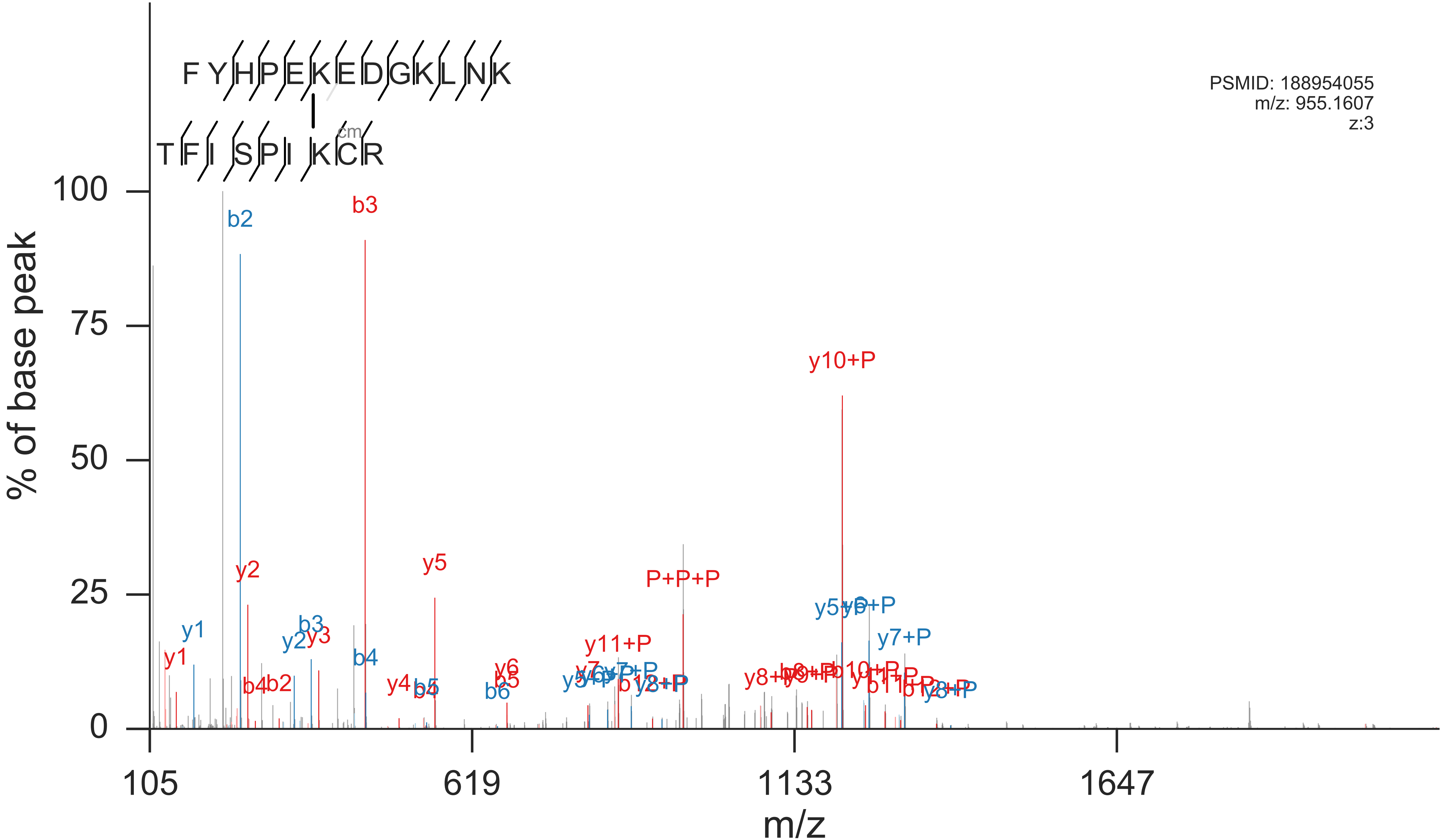
F Y H P E K E D G K L N K
T F I S P I K C R
cm
PSMID: 188954055
m/z: 955.1607
z:3
% of base peak
m/z
0
25
50
75
100
105
619
1133
1647
b2
b3
y10+P
y5
y2
P+P+P
y7+P

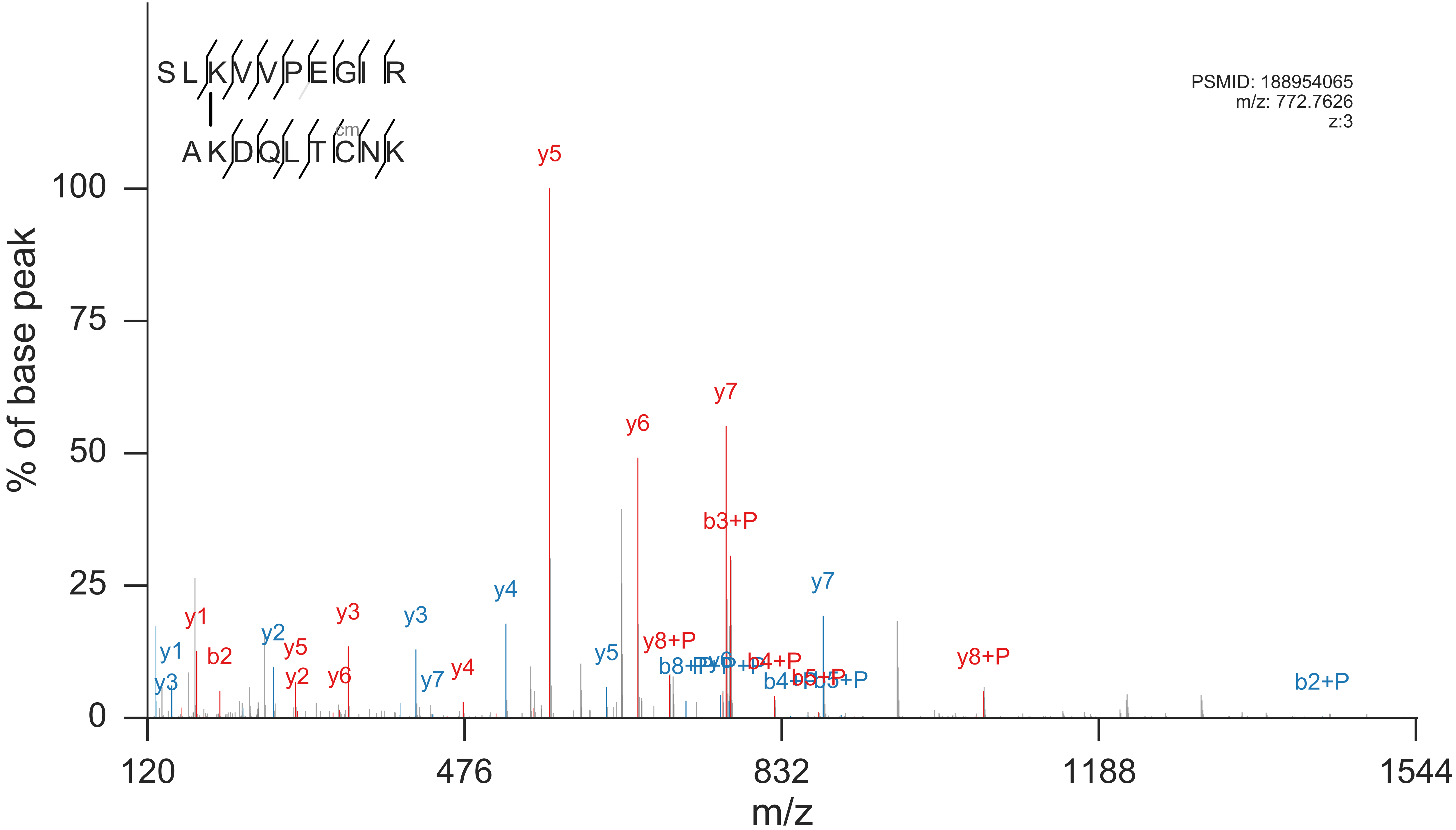
SLKVVPEGIR
AKDQLTCNK
cm
PSMID: 188954065
m/z: 772.7626
z:3
% of base peak
m/z
0
25
50
75
100
120
476
832
1188
1544
y5
y6
y7
b3+P
y7
y4
y3
y3
y2
y1
y1
b2
y5
y2
y6
y3
y7
y4
y5
y8+P
y8+P
b2+P

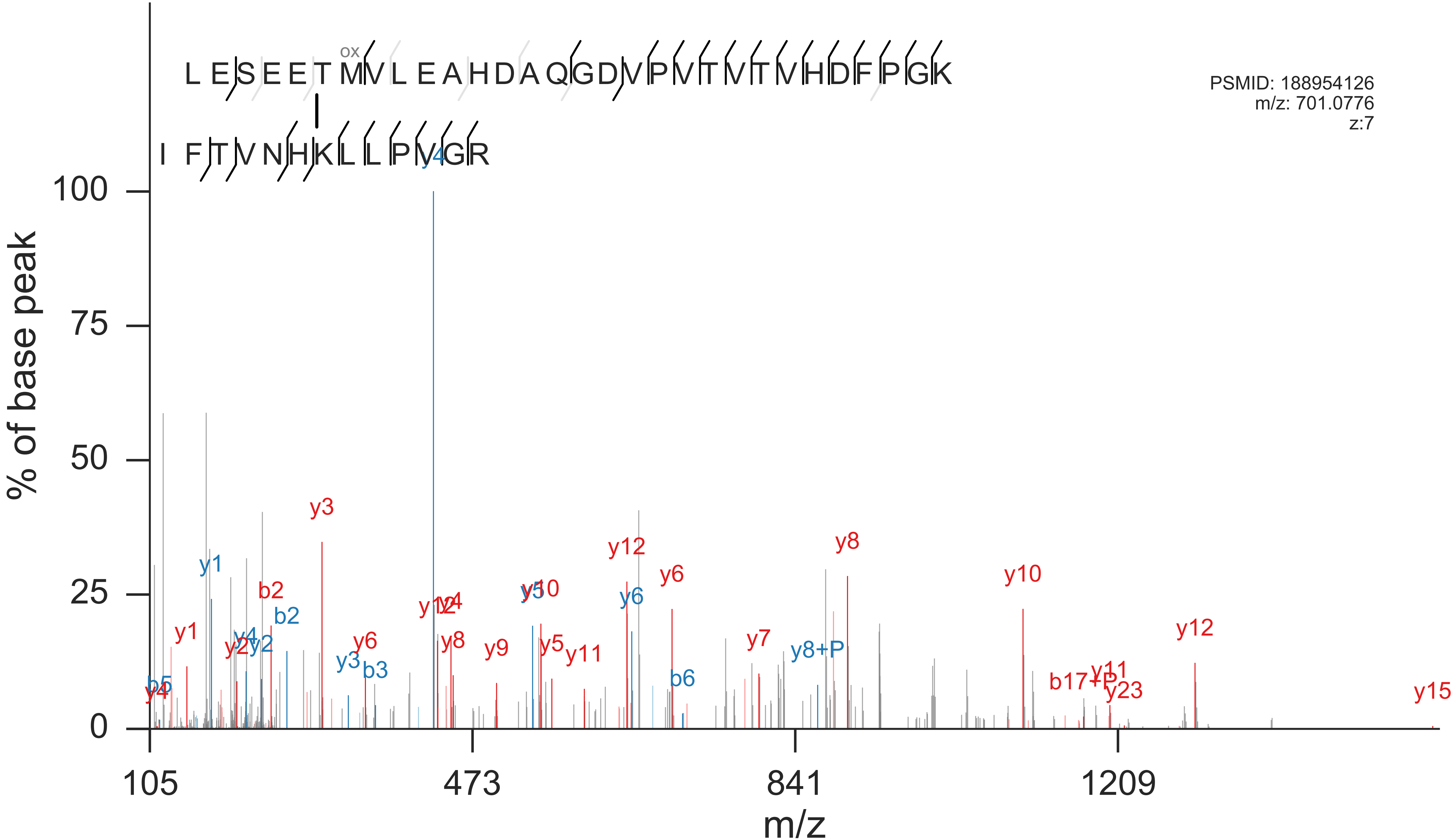

L E S E E T M V L E A H D A Q G D V P V T V T V H D F P G K
I F T V N H K L L P V G R
ox
PSMID: 188954126
m/z: 701.0776
z:7
% of base peak
m/z

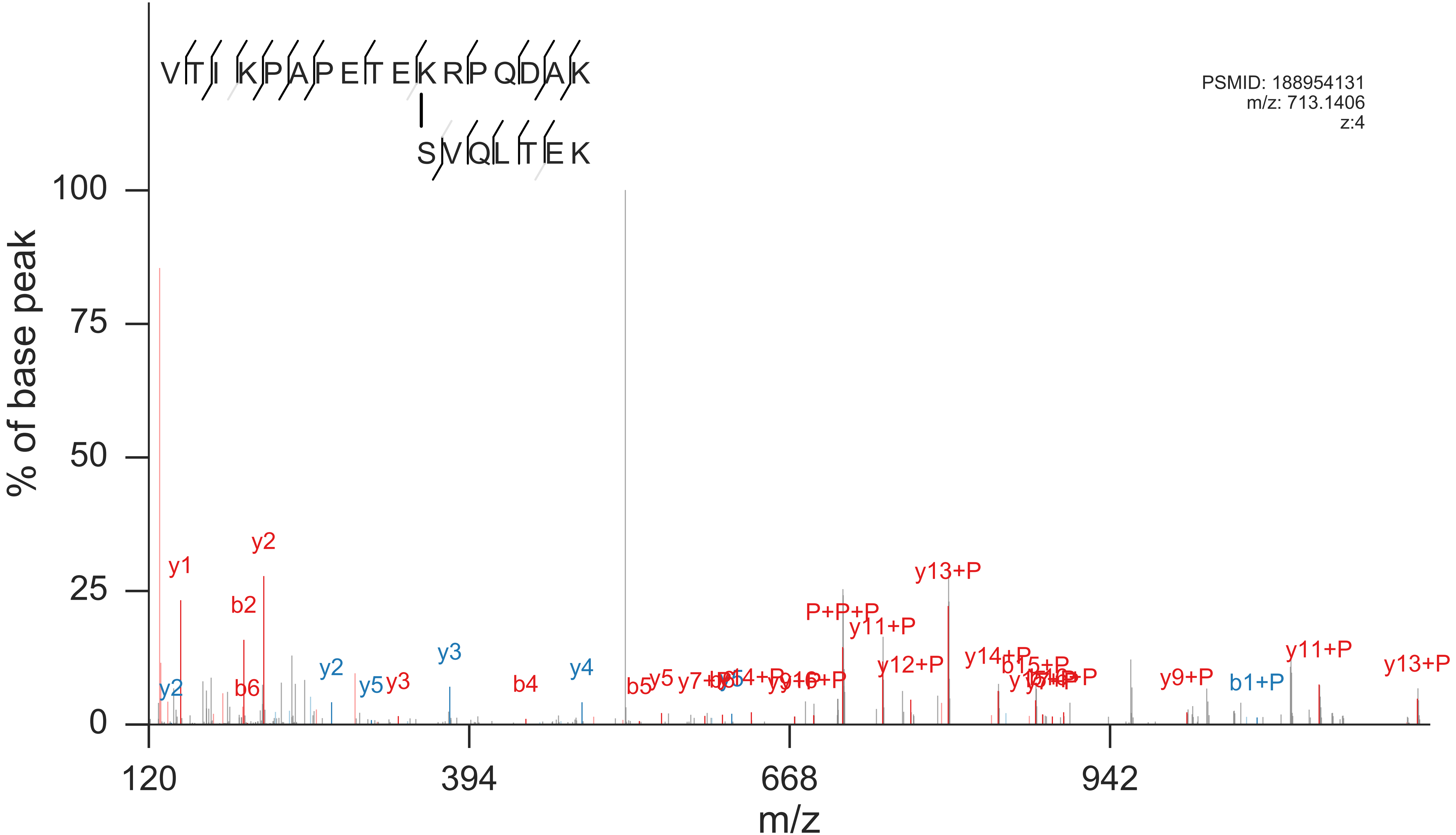

PSMID: 188954131
m/z: 713.1406
z:4
VTIKPAPETEKRPQDAK
SVQLTEK
% of base peak
m/z
0
25
50
75
100
120
394
668
942
y1
y2
b2
b6
y2
y2
y5
y3
y3
b4
y4
b5
y5
P+P+P
y11+P
y13+P
y12+P
y14+P
y9+P
b1+P
y11+P
y13+P

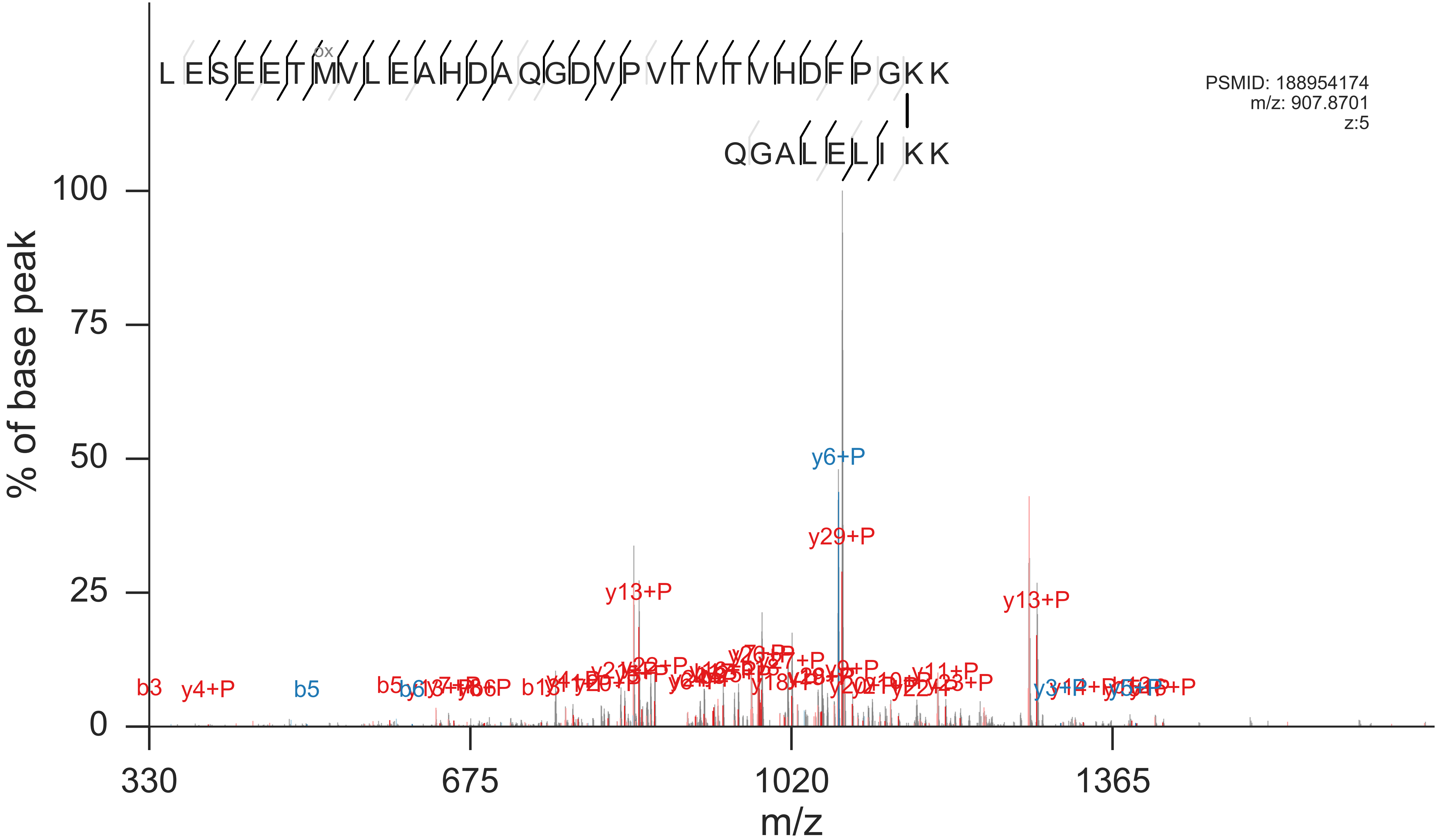

LESEETMVLEAHDAQGDVPVTVTVHDFPGKK
ox
QGALELIKK
PSMID: 188954174
m/z: 907.8701
z:5
% of base peak
100
75
50
25
0
330
675
1020
1365
m/z
y6+P
y29+P
y13+P
y13+P
b3
y4+P
b5
b5

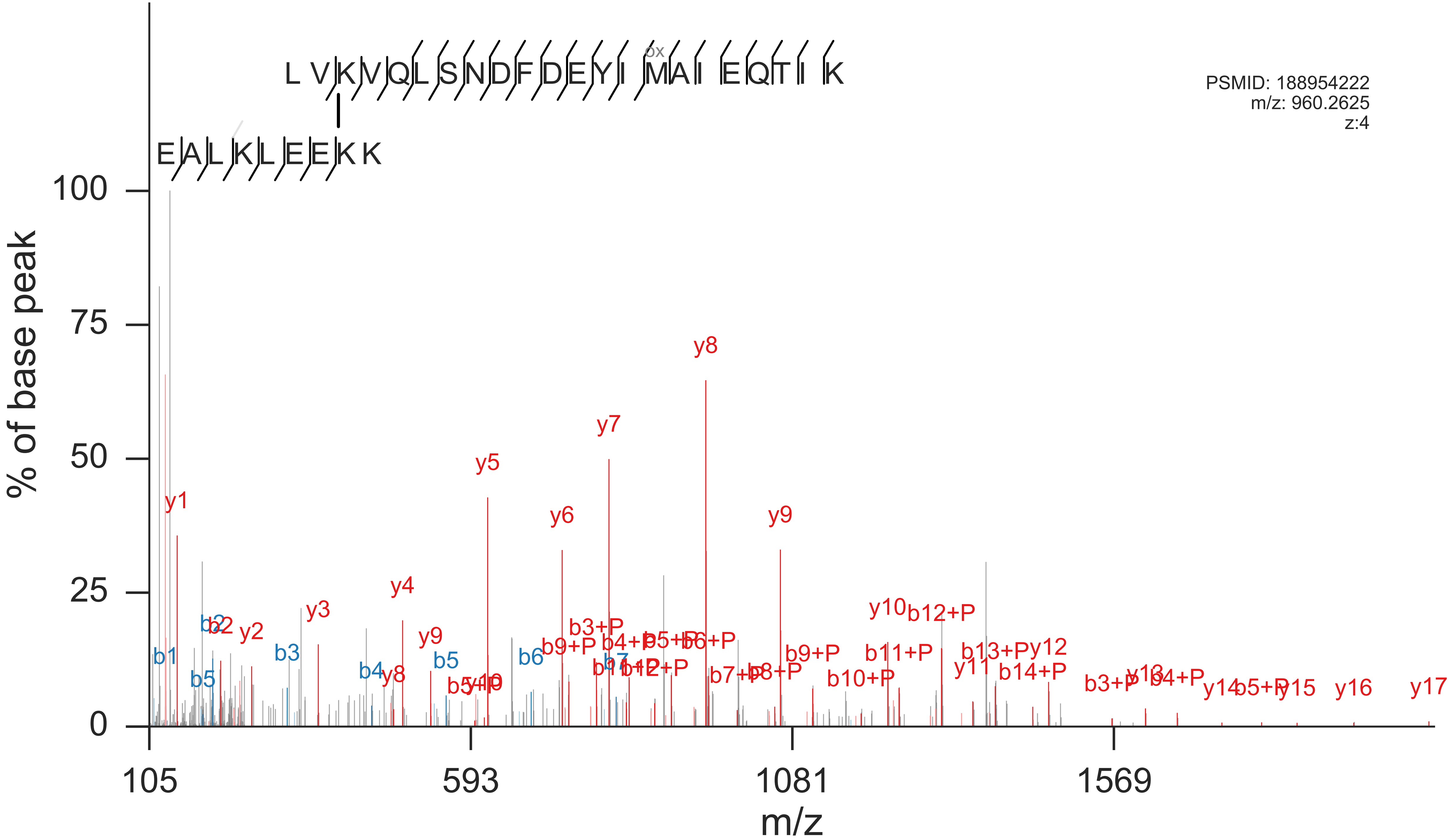

PSMID: 188954222
m/z: 960.2625
z:4
LVKVQLSNDFDEYIMAIEQTIK
EALKLEEKK
ox
% of base peak
m/z
100
75
50
25
0
105
593
1081
1569
y1
y2
y3
y4
y5
y6
y7
y8
y9
y10
y11
y12
y14
y15
y16
y17
b1
b2
b3
b4
b5
b6
b3+P
b9+P
b12+P
b11+P
b13+P
b14+P
b10+P
b7+P
b8+P

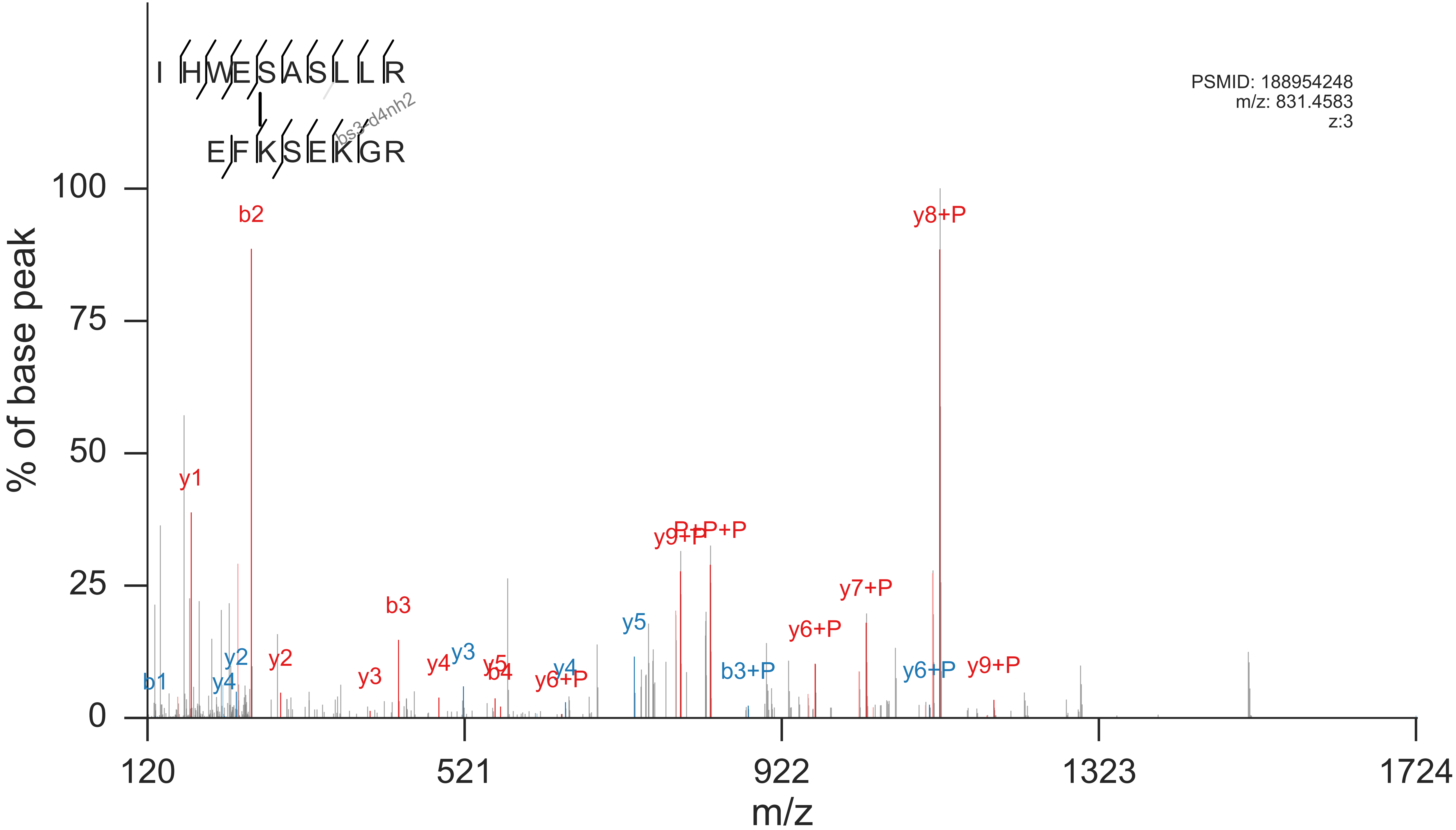

PSMID: 188954248
m/z: 831.4583
z:3
I HWESASLLR
EFKSEKGR
bs3-d4nh2
% of base peak
m/z
100
75
50
25
0
120
521
922
1323
1724
b2
y8+P
y1
b3
y5
y7+P
y6+P
b3+P
y6+P
y9+P
b1
y2
y4
y2
y3
y3
y4
b4
y6+P

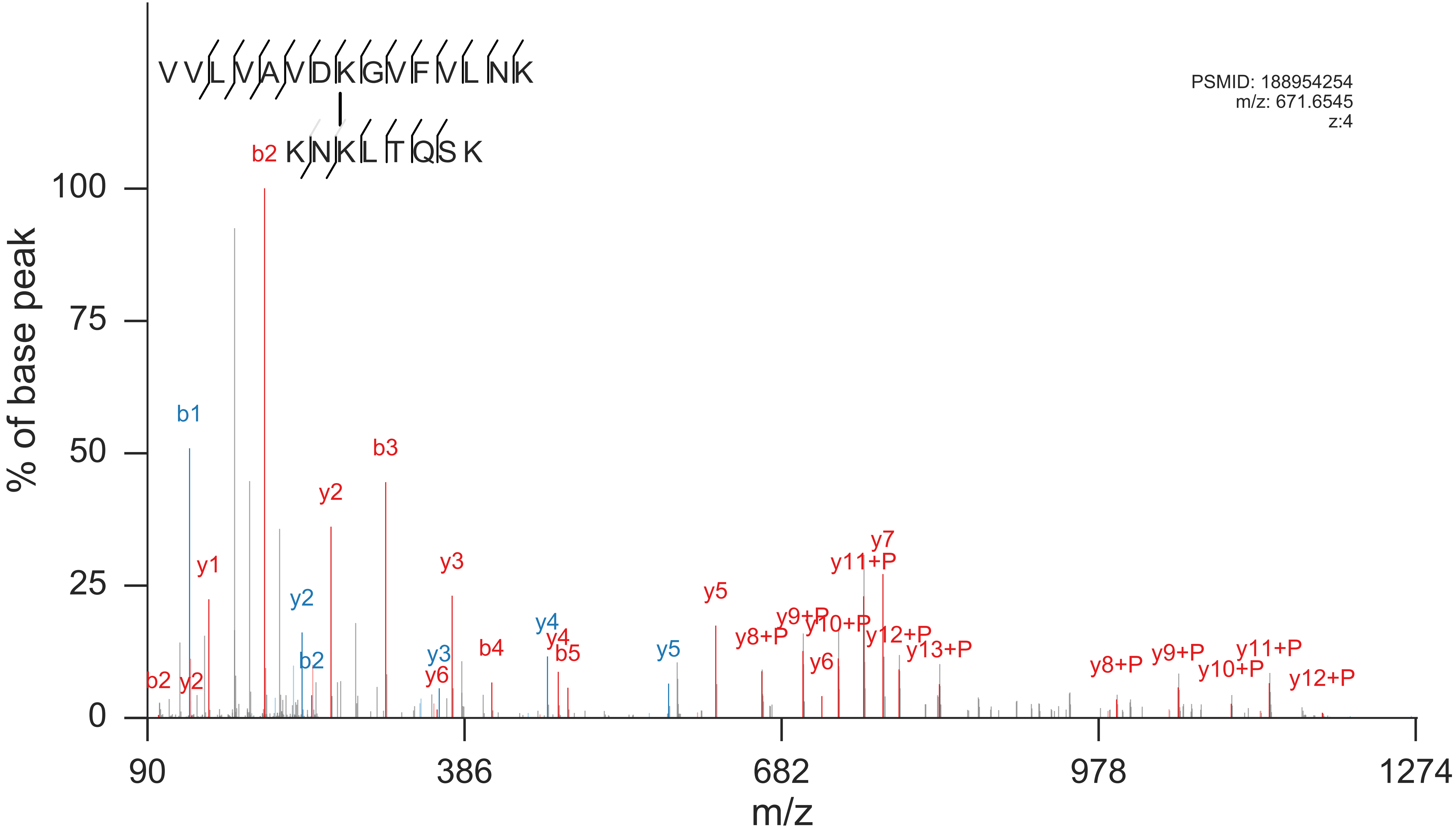

VVLVAVDKGVFVLNK
KNKLTQSK
PSMID: 188954254
m/z: 671.6545
z:4
% of base peak
m/z
0
25
50
75
100
90
386
682
978
1274
b1
b2
b3
b4
b5
y1
y2
y3
y4
y5
y6
y7
y8+P
y9+P
y10+P
y11+P
y12+P
y13+P

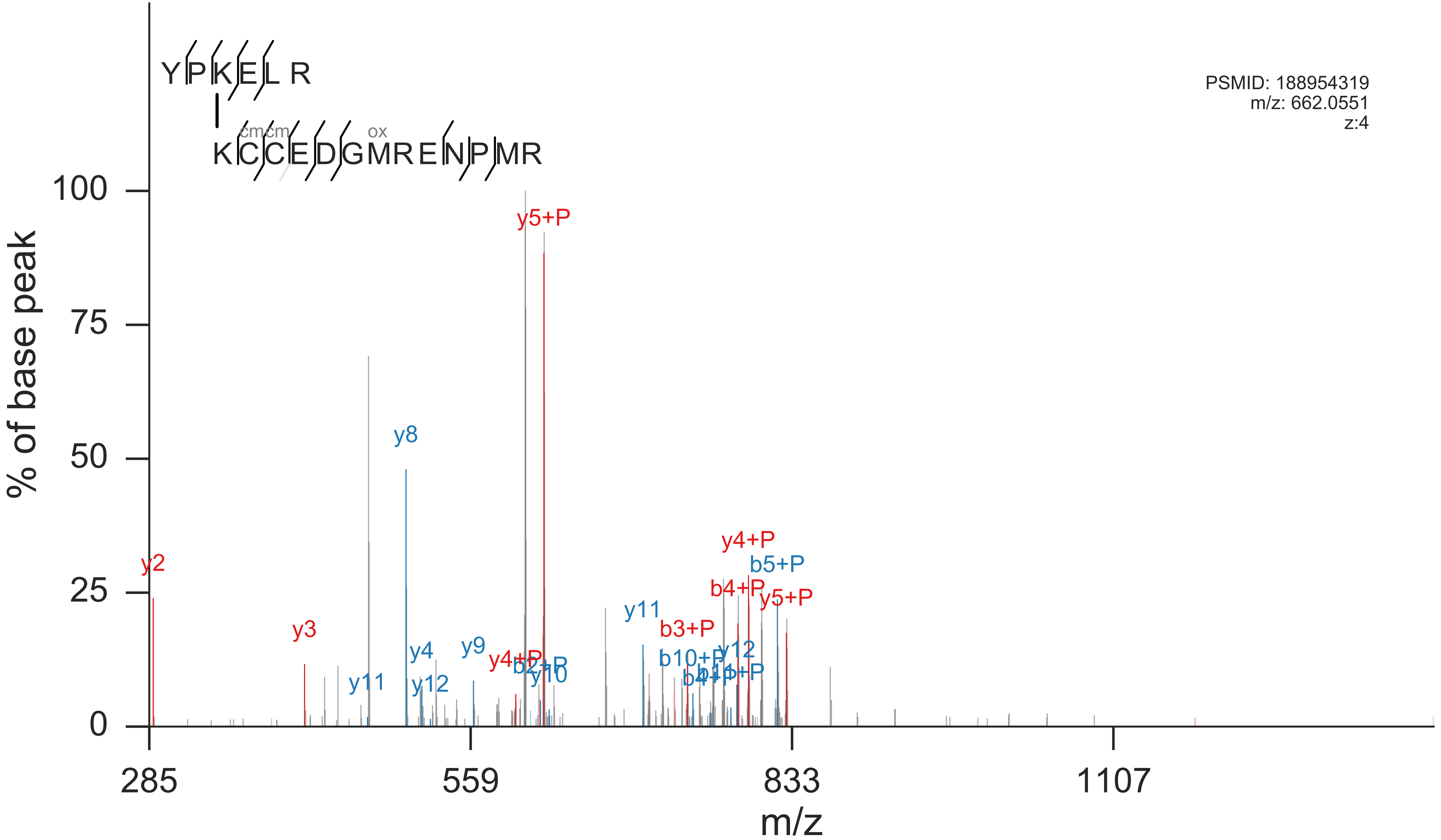
Y P K E L R
K C C E D G M R E N P M R
cm cm
ox
PSMID: 188954319
m/z: 662.0551
z:4
% of base peak
m/z
100
75
50
25
0
285
559
833
1107
y2
y3
y11
y8
y4
y12
y9
y4+P
b2+P
y10
y5+P
y11
b3+P
b10+P
b4+P
b11+P
y12
b4+P
y4+P
b5+P
y5+P

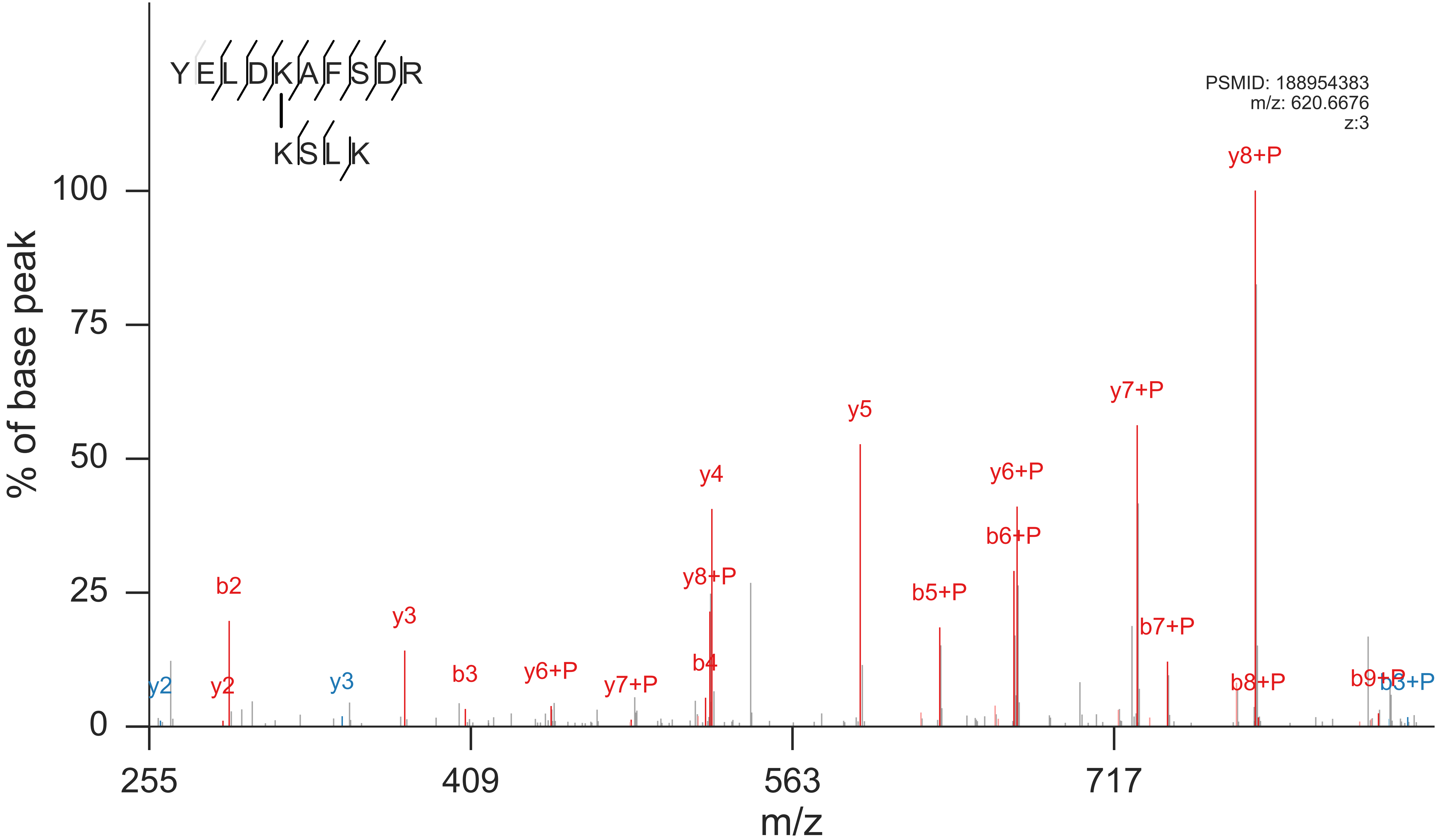
YELDKAFSDR
KSLK
PSMID: 188954383
m/z: 620.6676
z:3
% of base peak
m/z
y8+P
y7+P
y5
y6+P
y4
b6+P
y8+P
b5+P
b7+P
b2
y3
b3
y6+P
y7+P
b4
y2
y2
y3
b8+P
b9+P
b3+P
0
25
50
75
100
255
409
563
717

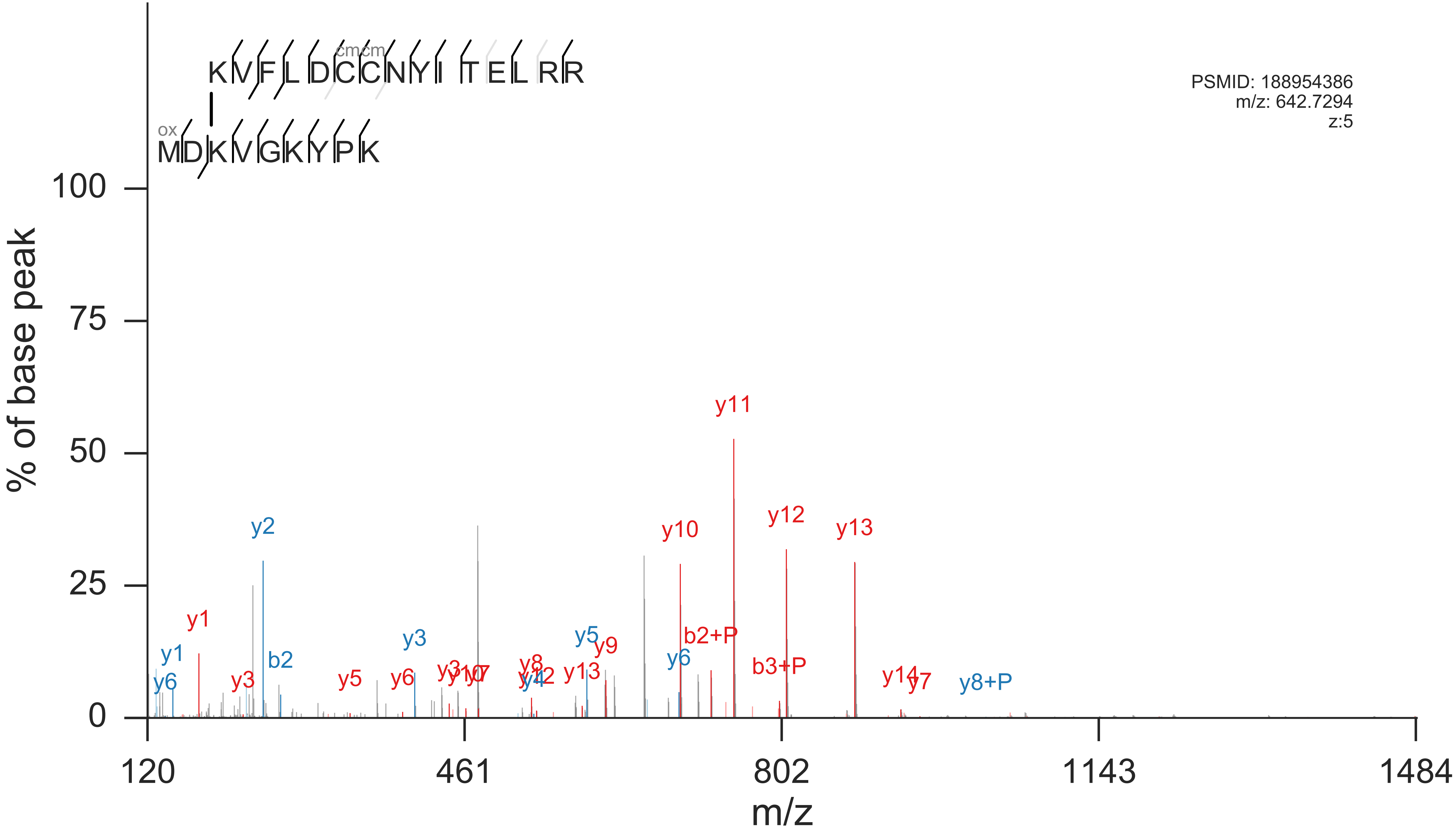

KVFLDCCNYITELRR
cm cm
ox
MDKVGKYPK
PSMID: 188954386
m/z: 642.7294
z:5
% of base peak
100
75
50
25
0
120
461
802
1143
1484
m/z
y11
y12
y13
y10
y2
y1
y1
y6
b2
y3
y5
y6
y3
y3
y10
y7
y8
y4
y12
y13
y5
y9
y6
b2+P
b3+P
y14
y7
y8+P

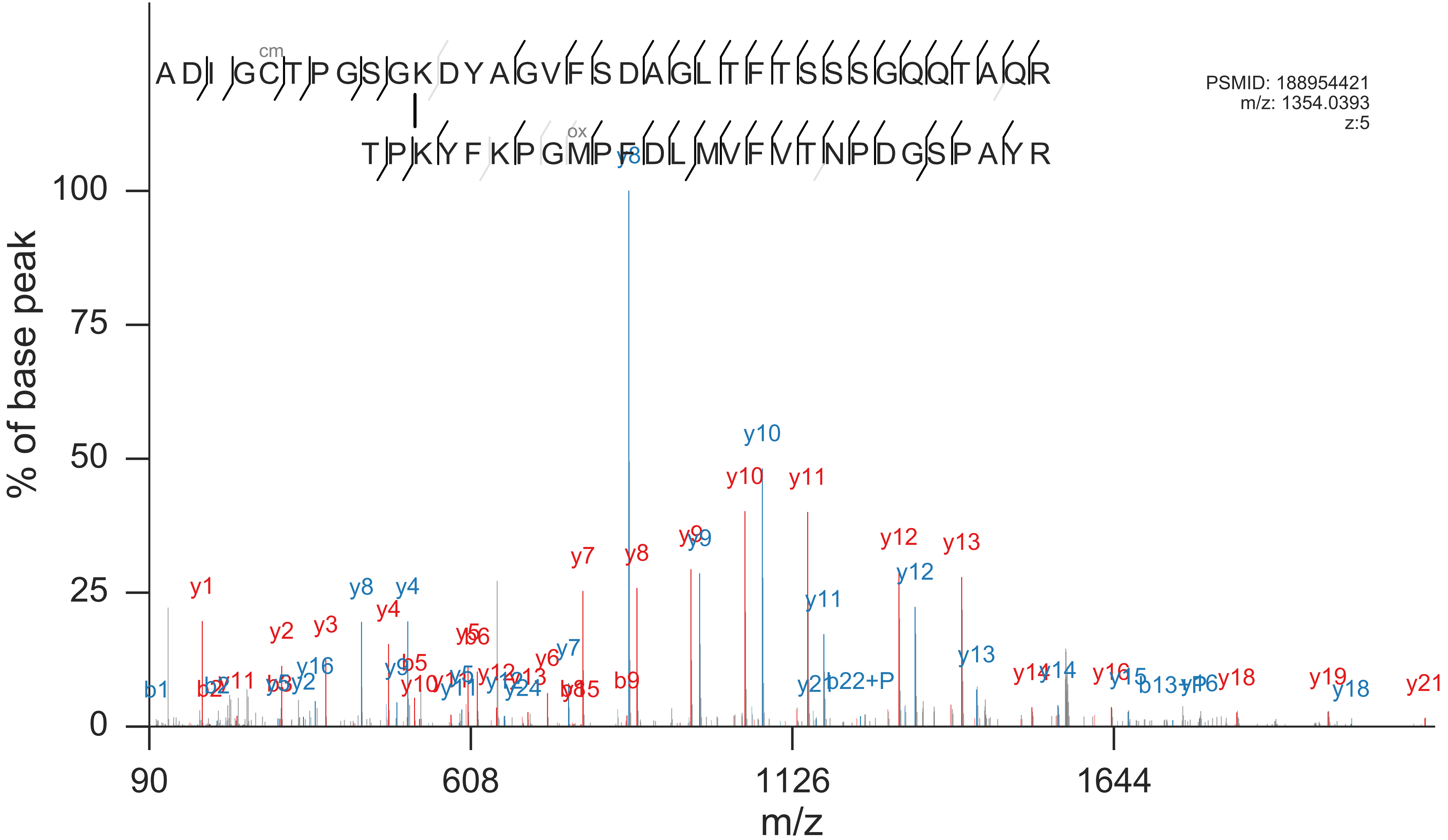

A D I G C T P G S G K D Y A G V F S D A G L T F T S S S G Q Q T A Q R
cm
T P K Y F K P G M P F D L M V F V T N P D G S P A Y R
ox
PSMID: 188954421
m/z: 1354.0393
z:5
% of base peak
100
75
50
25
0
90
608
1126
1644
m/z
y1
y2
y3
y4
y7
y8
y9
y10
y11
y12
y13
y14
y16
y18
y19
y21
b9
y6
y8
y4
y7
y9
y10
y11
y12
y13
y14
y15
y16
y18
y21
b1
b22+P

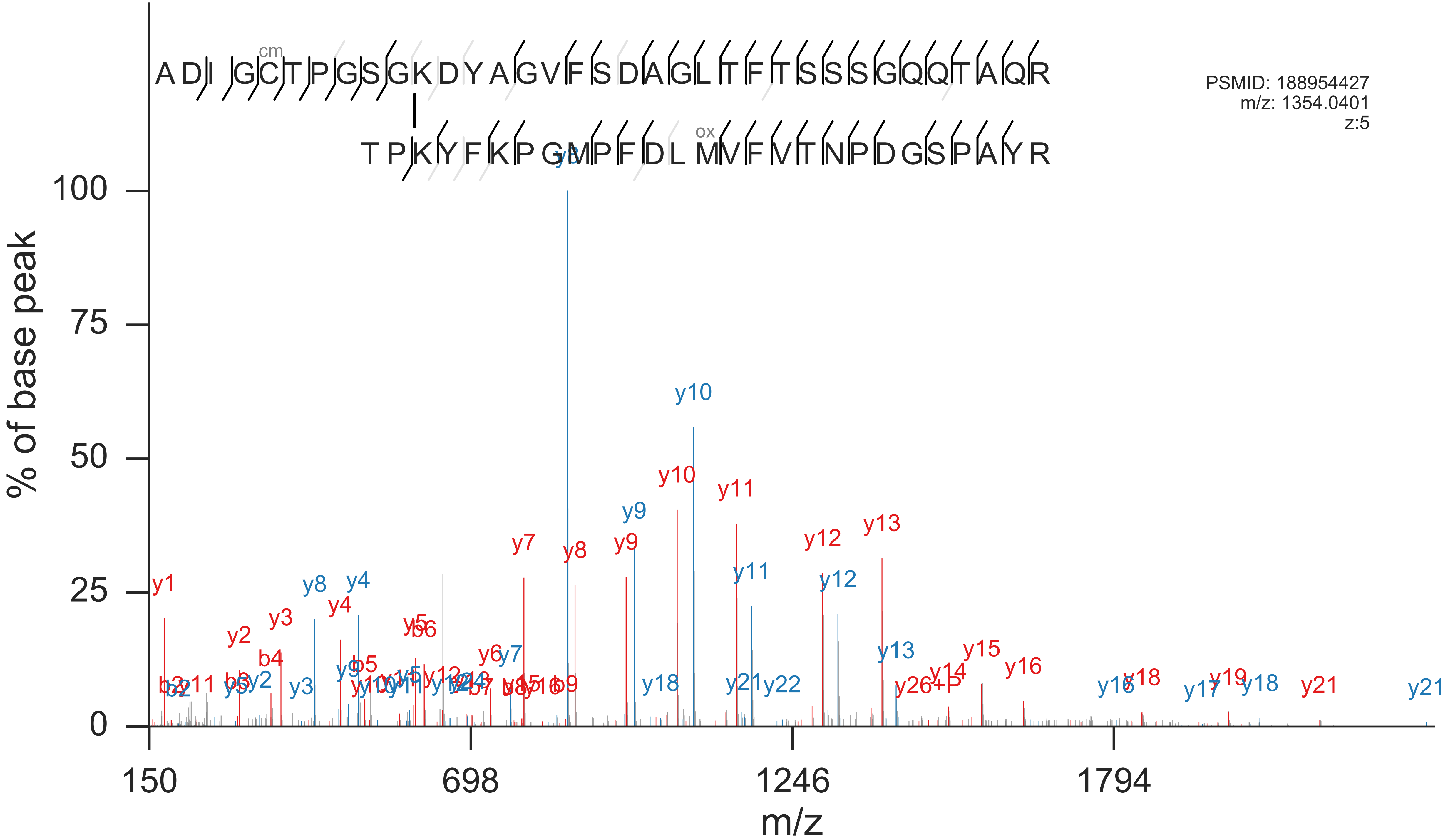
PSMID: 188954427
m/z: 1354.0401
z:5
A D I G C T P G S G K D Y A G V F S D A G L T F T S S S G Q Q T A Q R
T P K Y F K P G M P F D L M V F V T N P D G S P A Y R
cm
ox
% of base peak
m/z
100
75
50
25
0
150
698
1246
1794

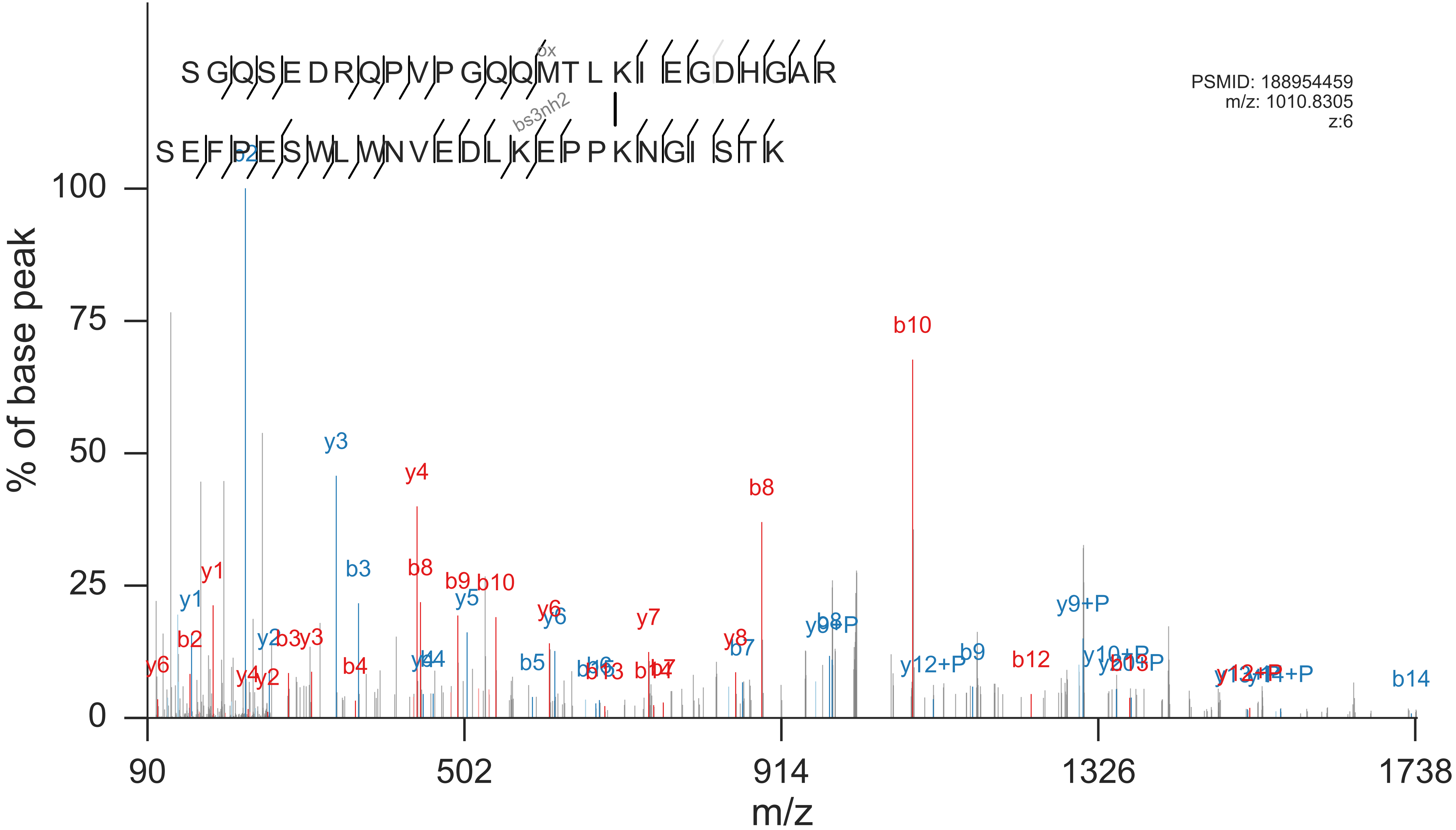
SGQSEDRQPVPGQQMTLKIEGDHGAR
SEFPESWLWNVEDLKEPPKNGISTK
ox
bs3nh2
PSMID: 188954459
m/z: 1010.8305
z:6
% of base peak
100
75
50
25
0
90
502
914
1326
1738
m/z
b10
y3
y4
b8
b3
b8
b9
b10
y1
y1
y5
y6
y6
y7
y8
b7
b2
y2
b3
y3
y9+P
y6
y4
y2
b4
y4
b5
b9
y12+P
b12
b14

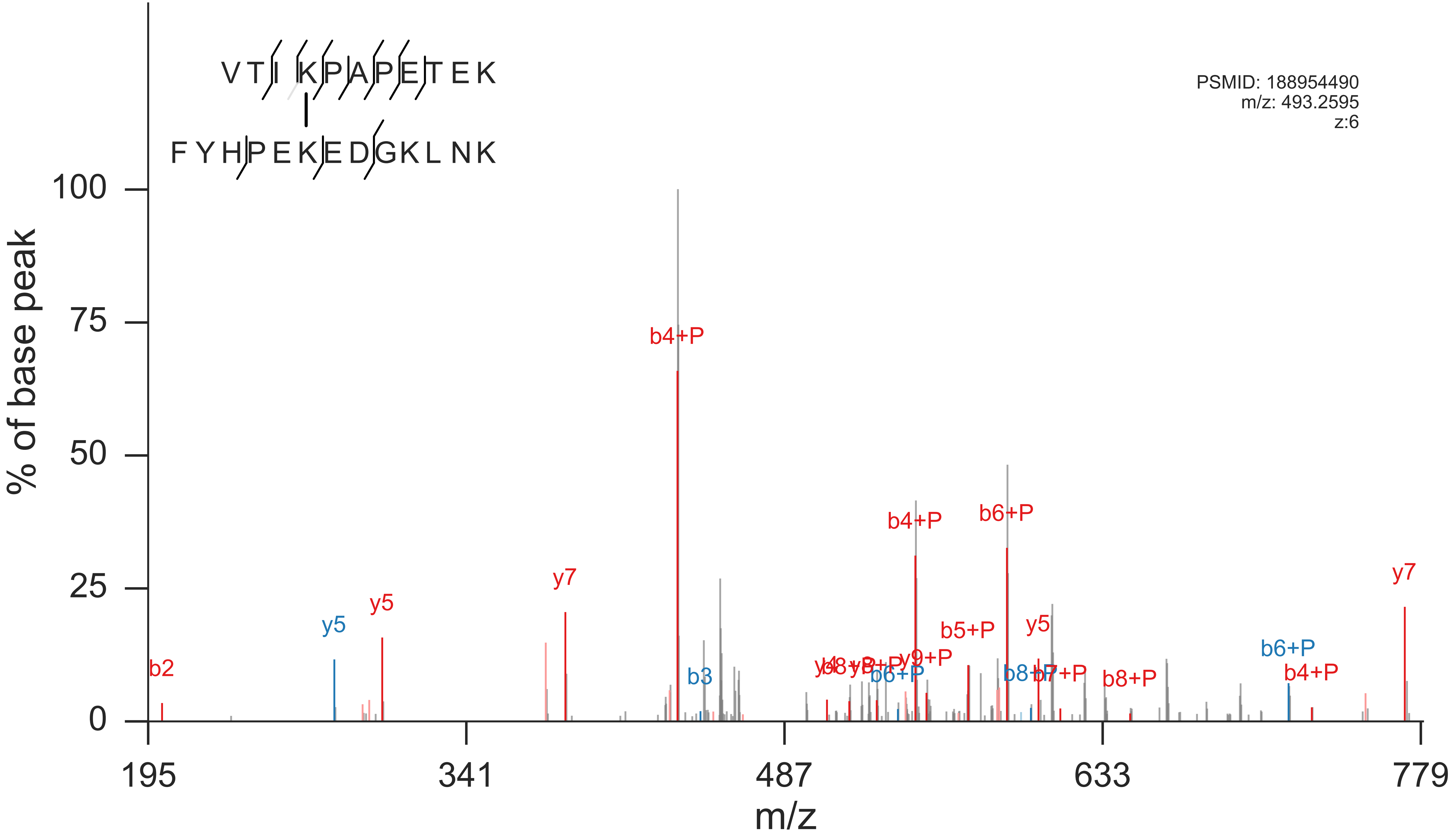

V T I K P A P E T E K
F Y H P E K E D G K L N K
PSMID: 188954490
m/z: 493.2595
z:6
% of base peak
100
75
50
25
0
195
341
487
633
779
m/z
b2
y5
y5
y7
b4+P
b3
b4+P
b6+P
b5+P
y5
b6+P
b8+P
b6+P
b4+P
y7

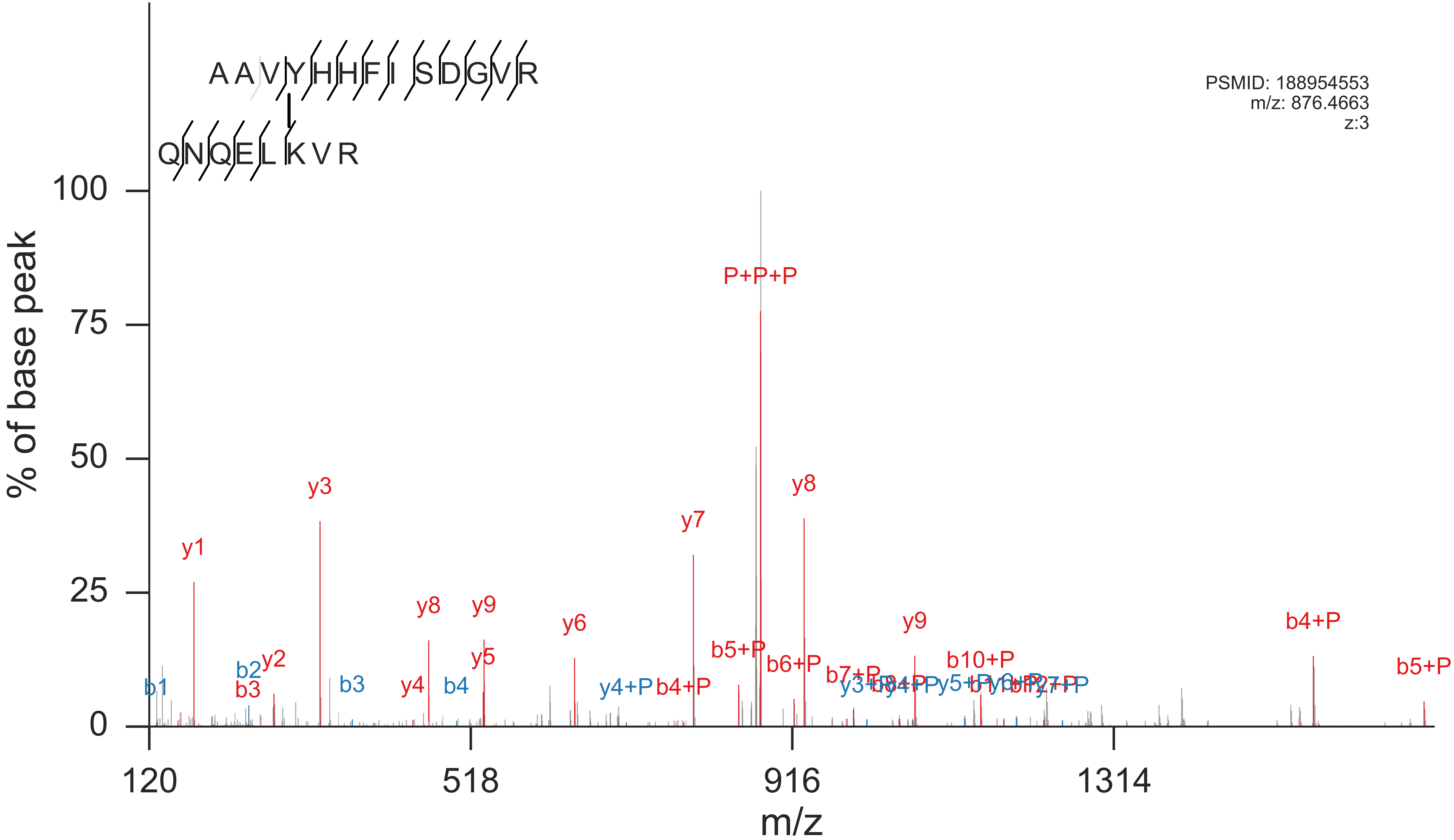
AAVYHHFISDGVR
QNQELKVR
PSMID: 188954553
m/z: 876.4663
z:3
% of base peak
100
75
50
25
0
120
518
916
1314
m/z
P+P+P
y1
y2
y3
y4
y5
y6
y7
y8
y9
y8
y9
b1
b2
b3
b3
b4
y4+P
b4+P
b5+P
b6+P
b7+P
b10+P
b4+P
b5+P

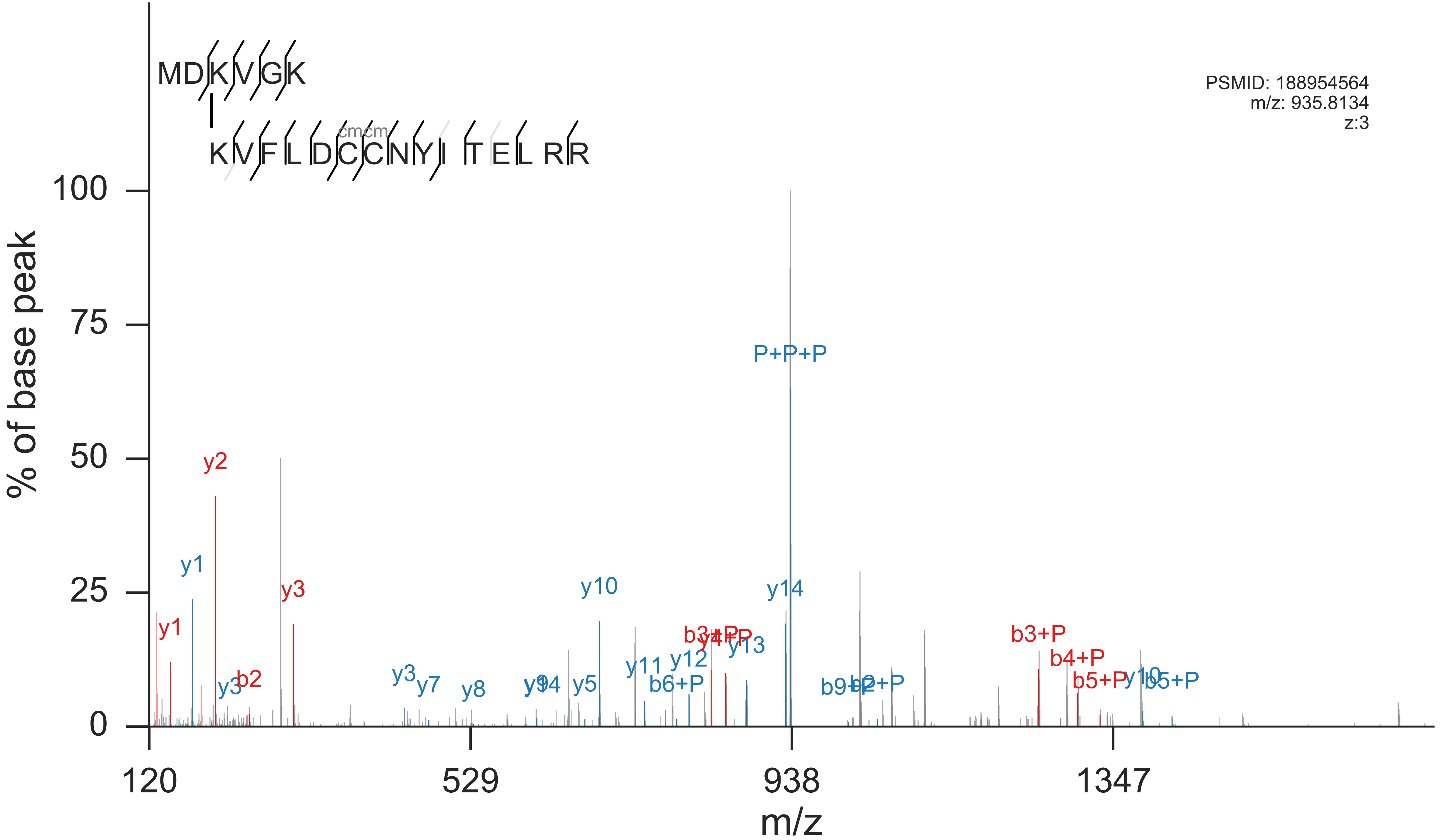

MDKVGK
KVFLDCCNYITELRR
cmcm
PSMID: 188954564
m/z: 935.8134
z:3
% of base peak
m/z
P+P+P
y1
y2
y3
b2
y10
y14
y11
y12
y13
b6+P
b3+P
b4+P
b5+P
y5
y7
y8

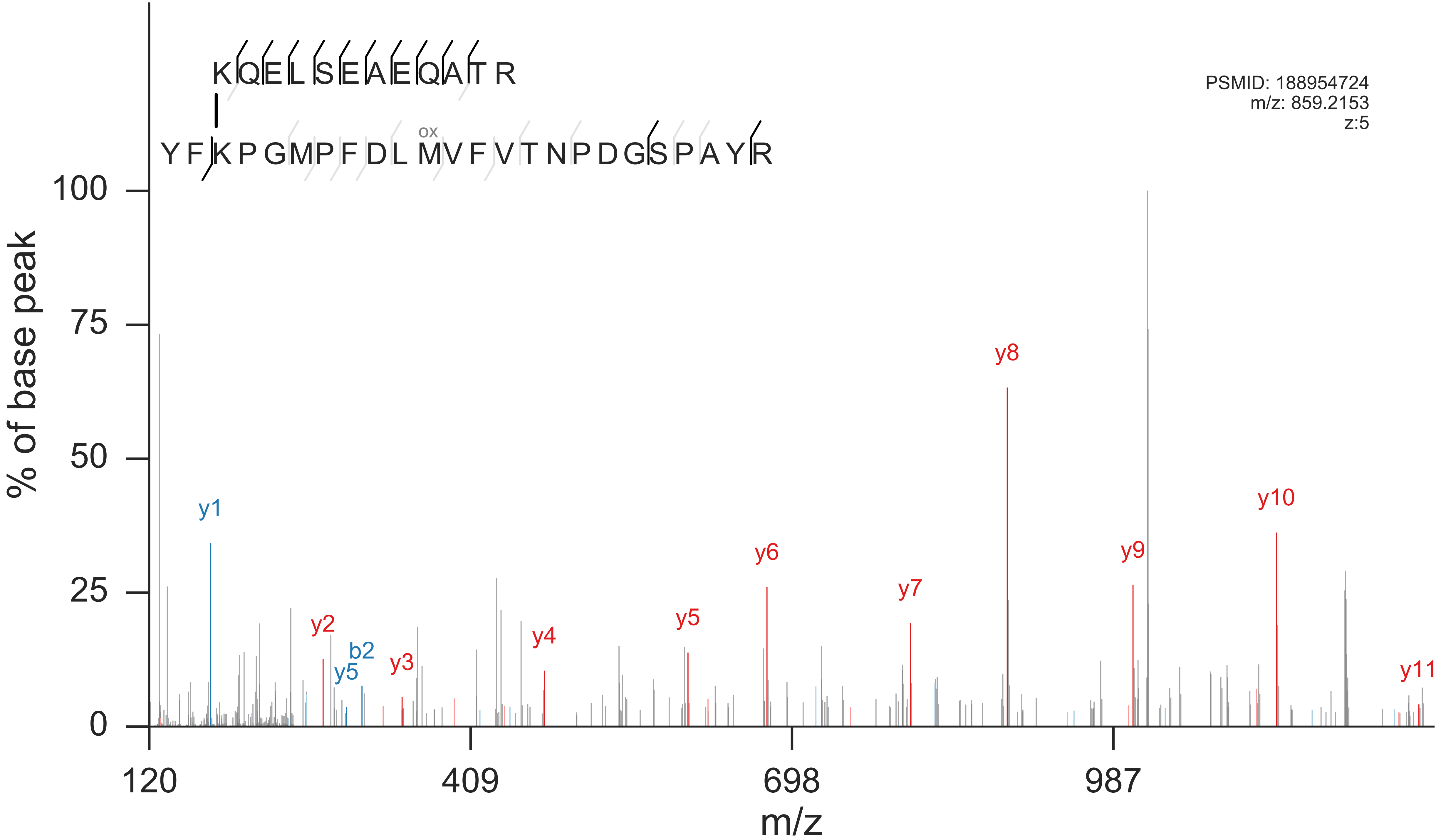

KQELSEAEQATR
YFKPGMPFDLMVFVTNPDGSPAYR
ox
PSMID: 188954724
m/z: 859.2153
z:5
% of base peak
100
75
50
25
0
y1
y2
y5
b2
y3
y4
y5
y6
y7
y8
y9
y10
y11
120
409
698
987
m/z

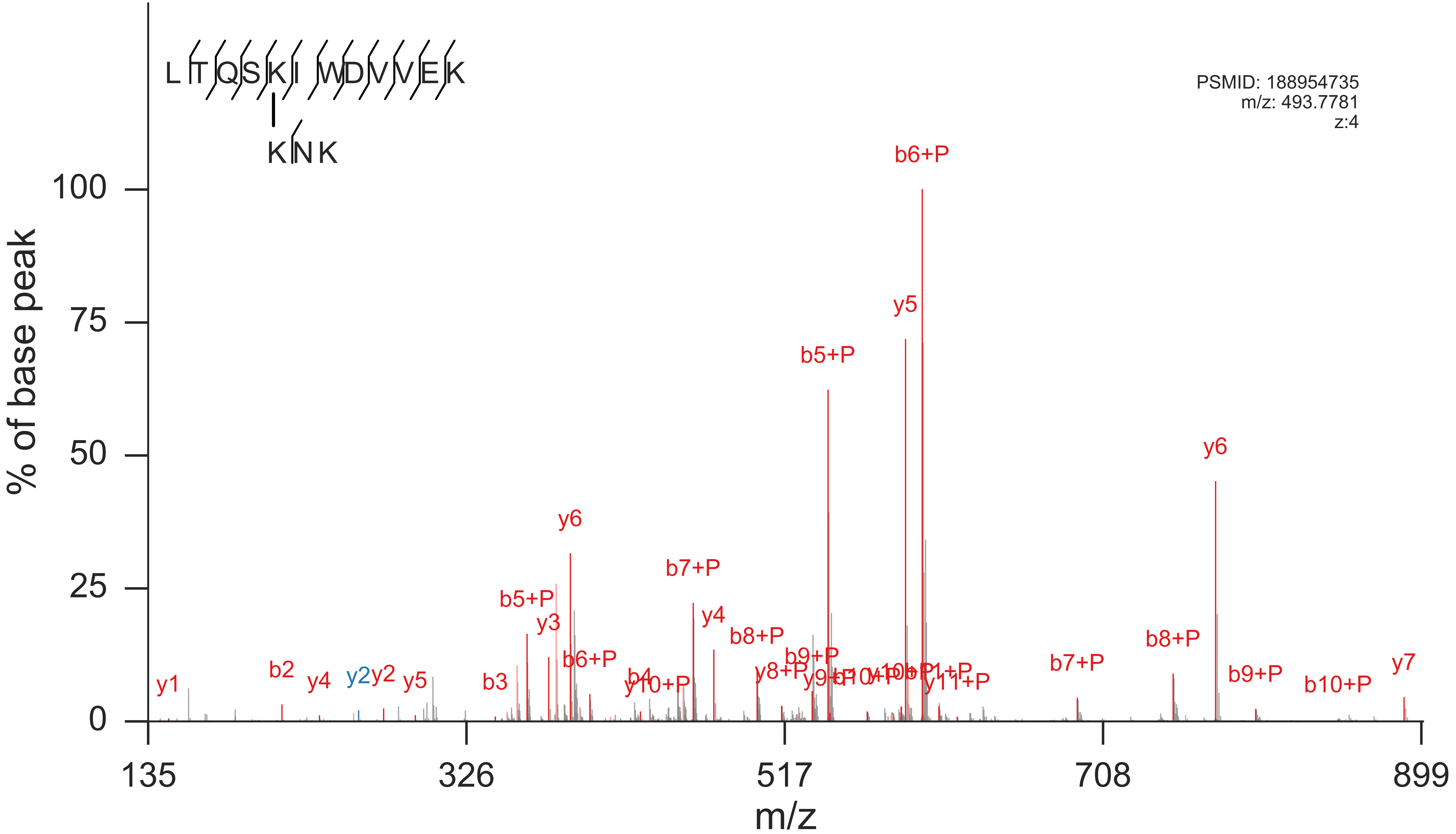

LTQSKIWDVVEK
KNK
PSMID: 188954735
m/z: 493.7781
z:4
% of base peak
m/z
b6+P
y5
b5+P
y6
b7+P
b5+P
y3
y4
b8+P
b6+P
b9+P
y8+P
y10+P
b4
b7+P
b8+P
b9+P
b10+P
y7
y11+P
y1
b2
y4
y2
y2
y5
b3

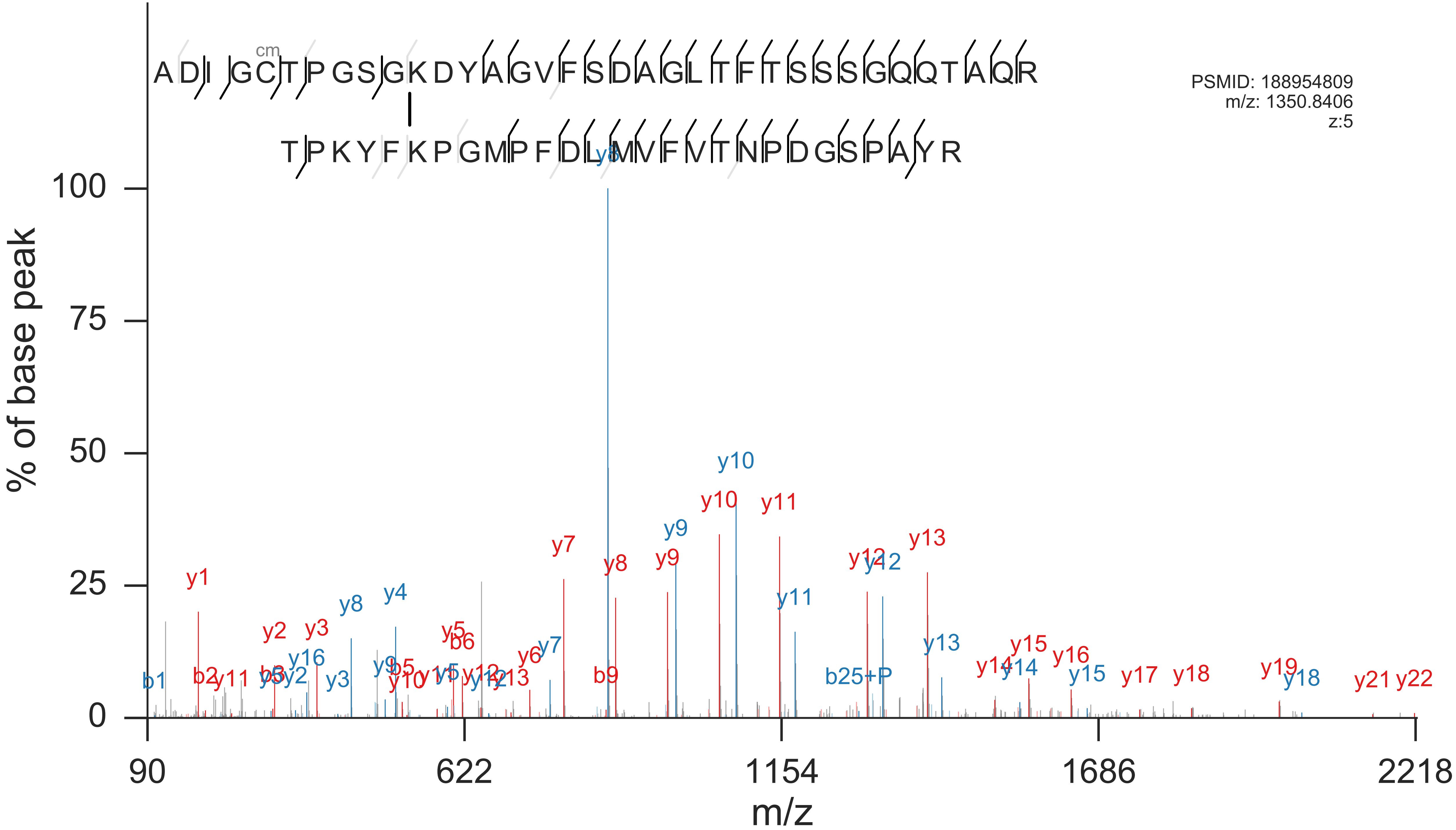

ADIGCTPGSGKDYAGVFSDAGLTFTSSSGQQTAQR
cm
TPKYFKPGMPFDLMVFVTNPDGSPAYR
PSMID: 188954809
m/z: 1350.8406
z:5
% of base peak
100
75
50
25
0
90
622
1154
1686
2218
m/z

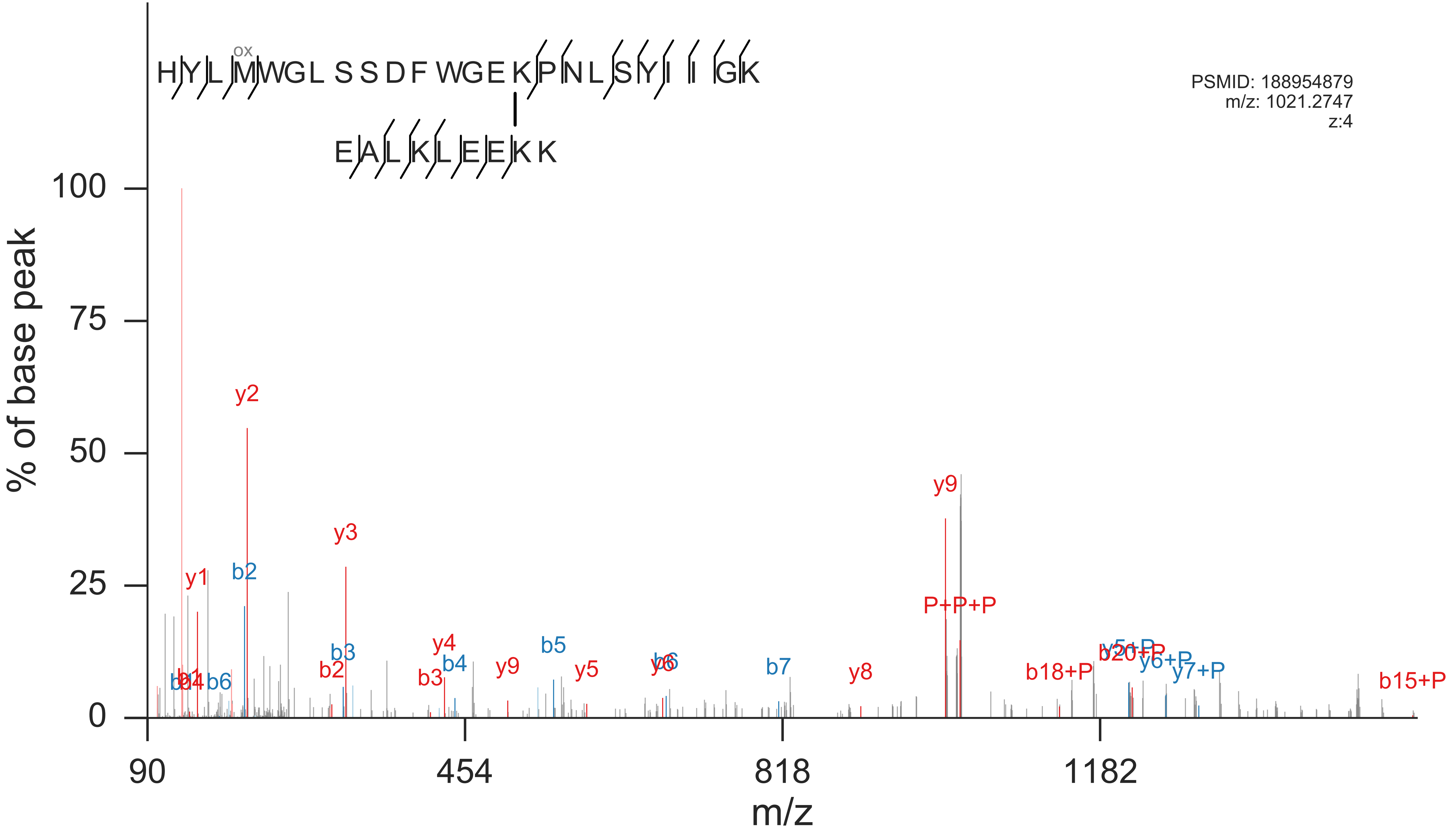

HYLMWGLSSDFWGEKPNLSYIIGK
ox
EALKLEEKK
PSMID: 188954879
m/z: 1021.2747
z:4
% of base peak
100
75
50
25
0
90
454
818
1182
m/z
y2
y1
b2
y3
b3
b2
b4
y4
b4
y9
b5
y5
y6
b6
b7
y8
y9
P+P+P
b18+P
b20+P
y5+P
y6+P
y7+P
b15+P
b1
b6
b3

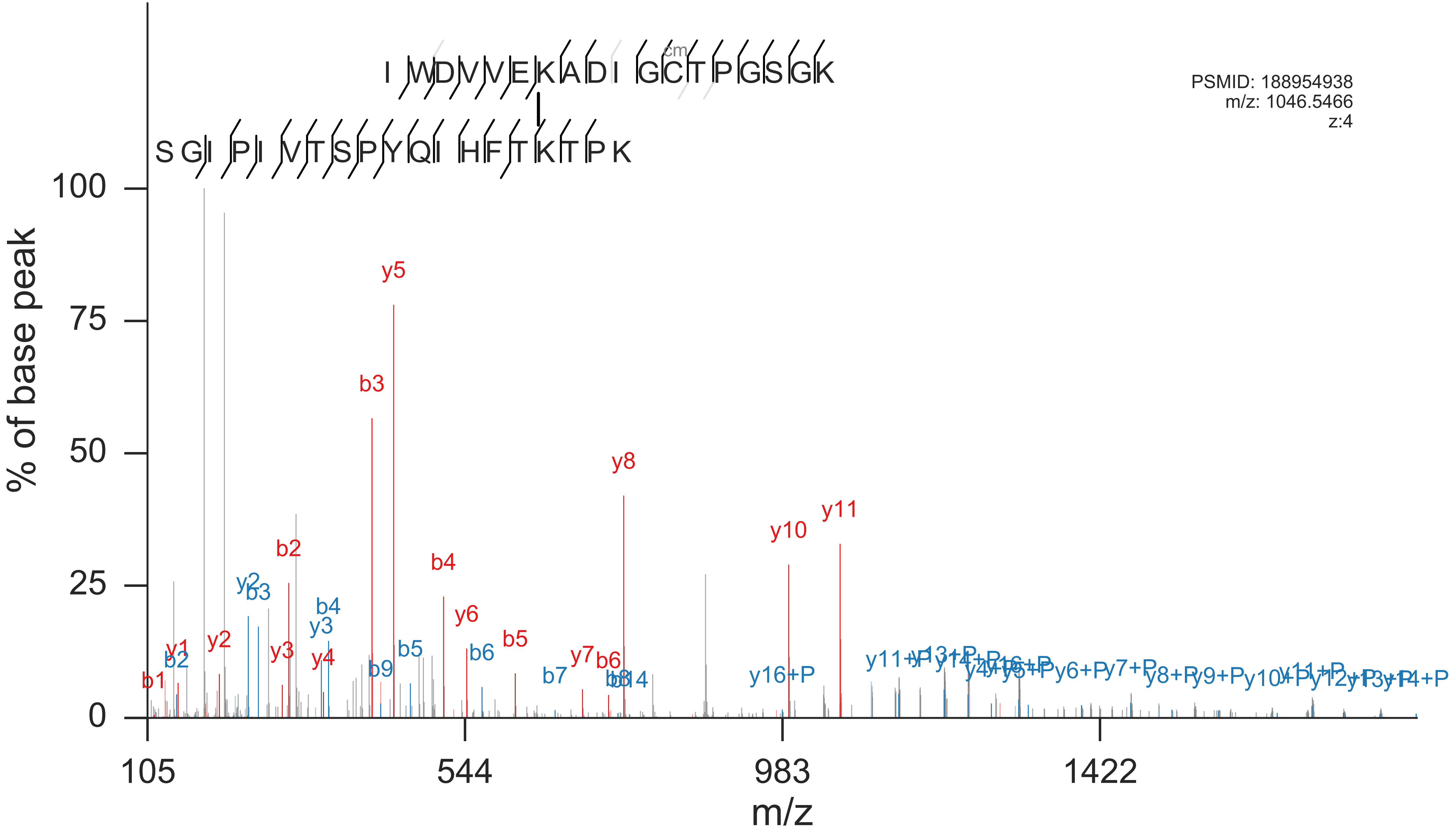

I WDVVEKADI GCTPGSGK
SGIPIVTSPYQIHFTKTPK
PSMID: 188954938
m/z: 1046.5466
z:4
% of base peak
m/z
105
544
983
1422
0
25
50
75
100
y5
b3
y8
y10
y11
b2
b4
y6
b5
y7
b6
y1
b1
y2
y3
y4
b2
y2
b3
b4
y3
b5
b6
b9
b7
y16+P

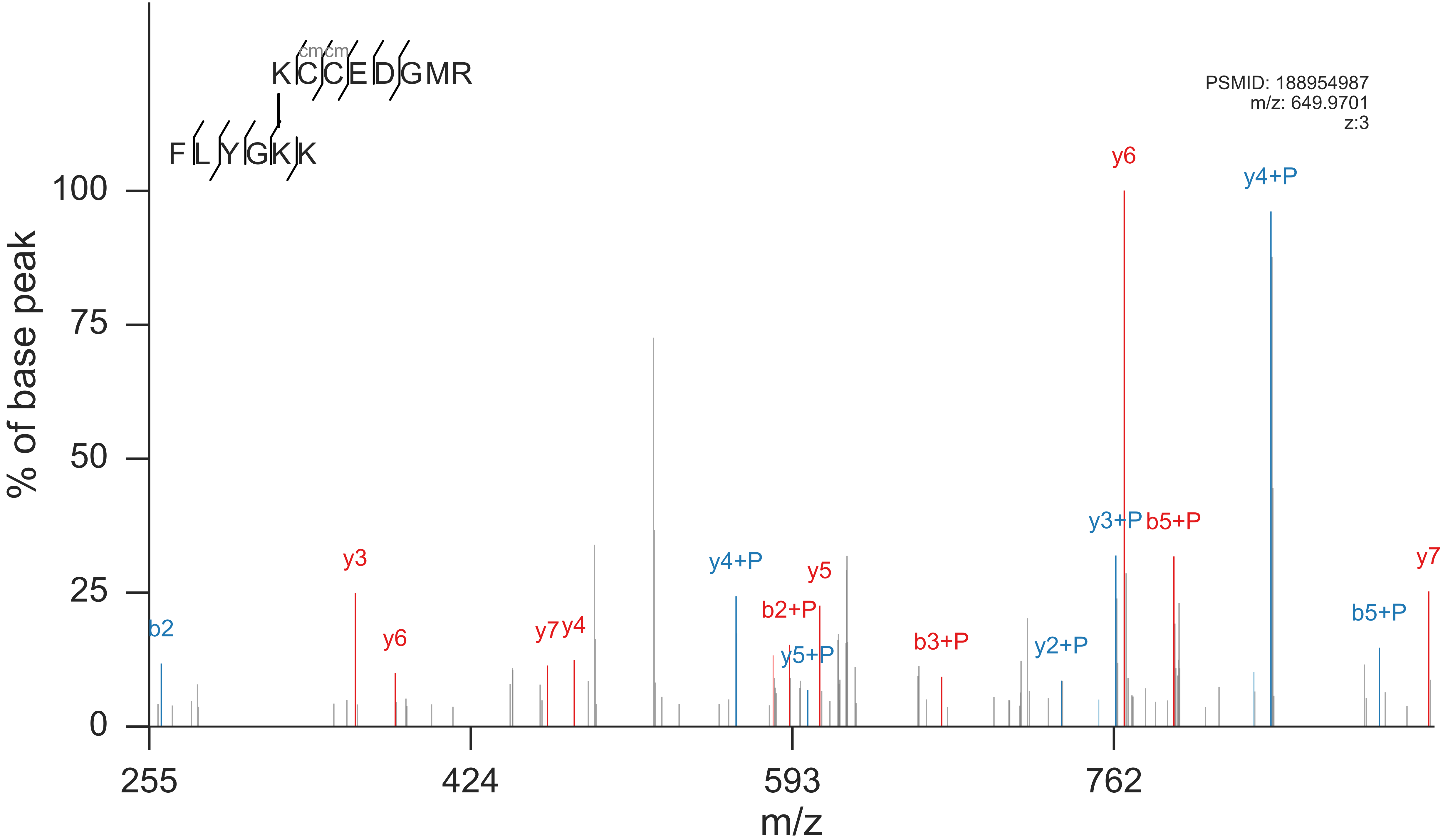

KCCEDGMR
cmcm
FLYGKK
PSMID: 188954987
m/z: 649.9701
z:3
% of base peak
100
75
50
25
0
255
424
593
762
m/z
b2
y3
y6
y7
y4
y4+P
b2+P
y5
y5+P
b3+P
y2+P
y3+P
y6
b5+P
y4+P
b5+P
y7

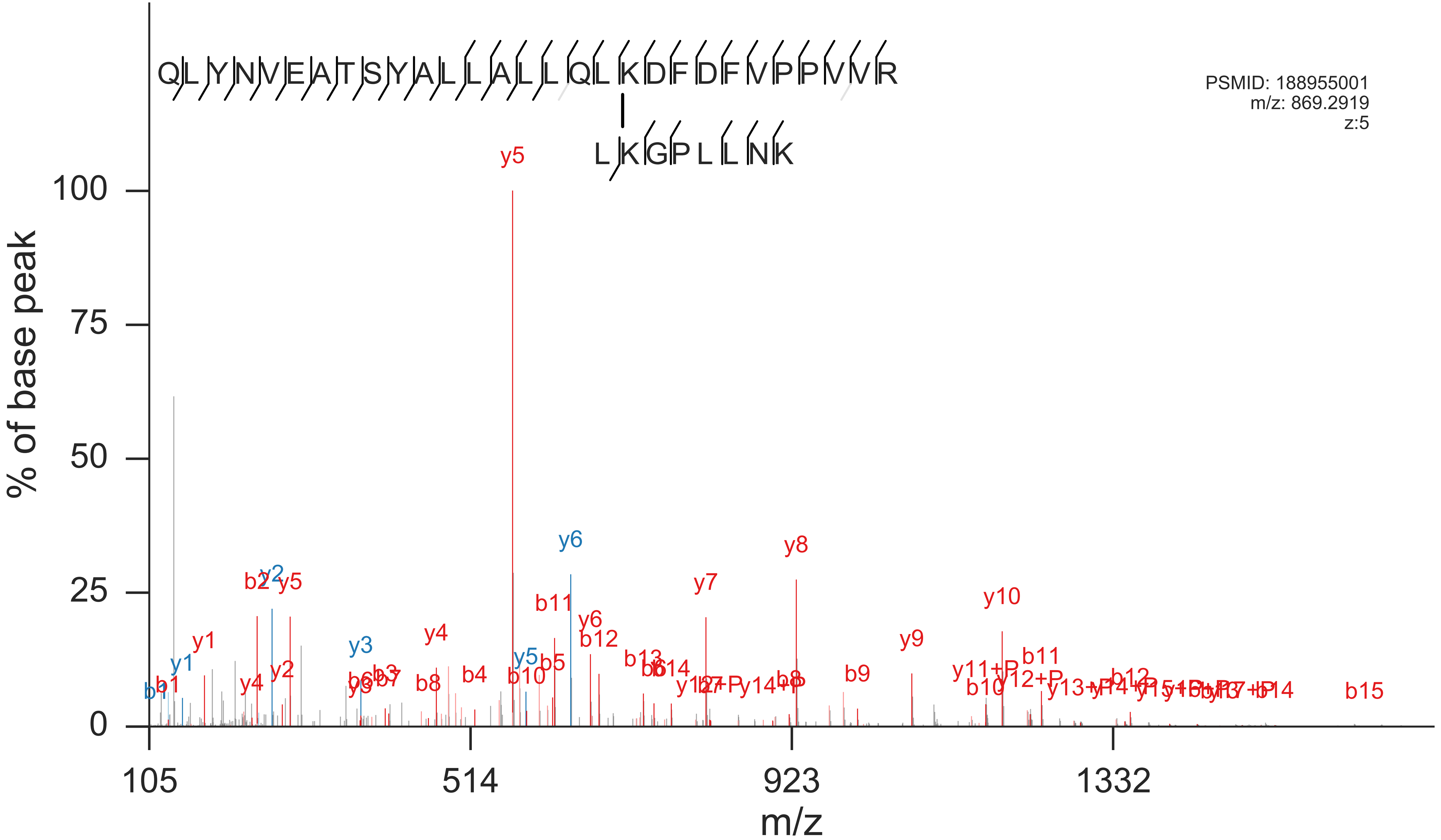

QLYNVEATSYALLALLQLKDFDFVPPVVR
LKGPLLNK
PSMID: 188955001
m/z: 869.2919
z:5
% of base peak
m/z

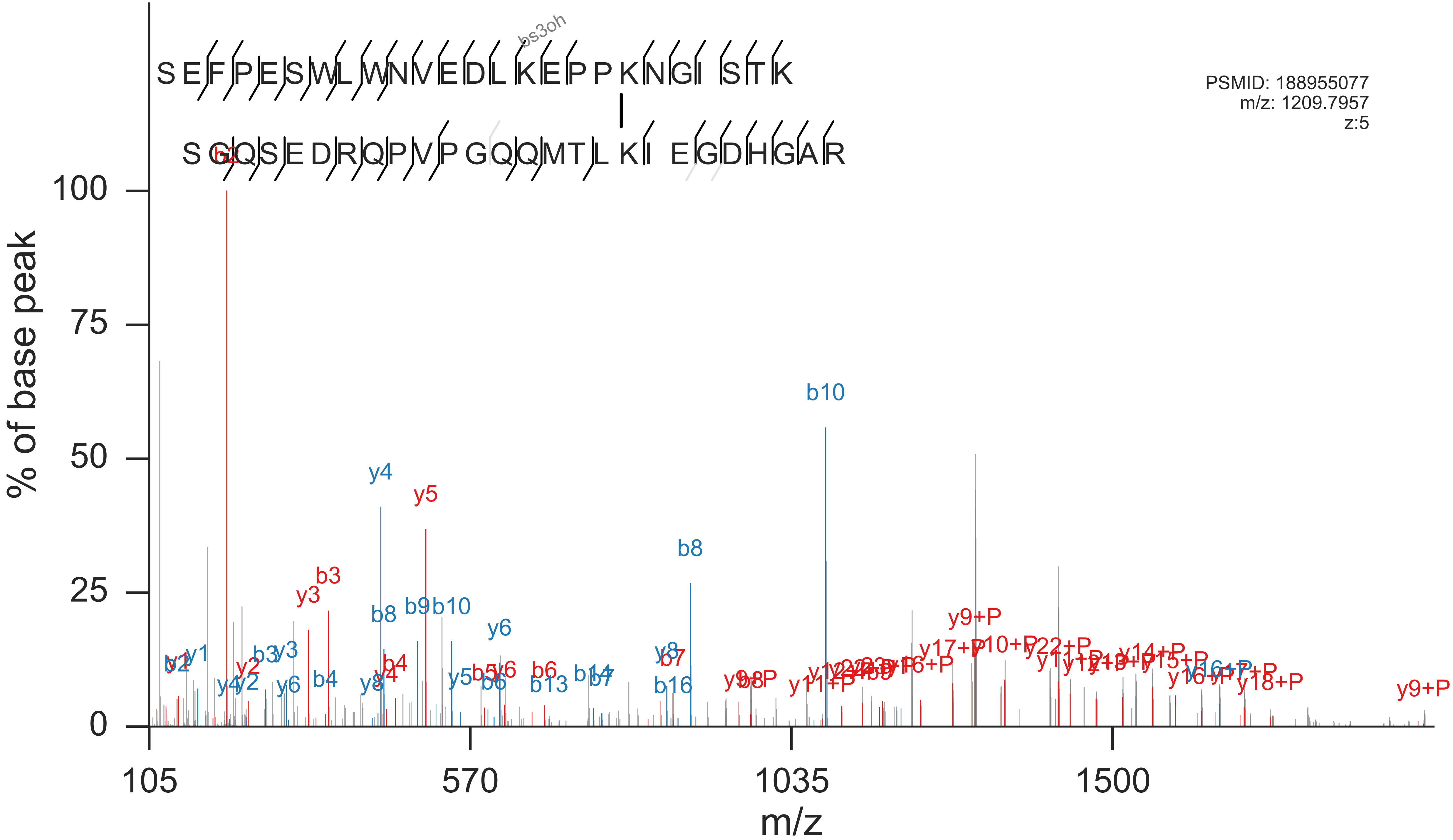

PSMID: 188955077
m/z: 1209.7957
z:5
S E F P E S W L W N V E D L K E P P K N G I S T K
S G Q S E D R Q P V P G Q Q M T L K I E G D H G A R
bs3oh
% of base peak
m/z

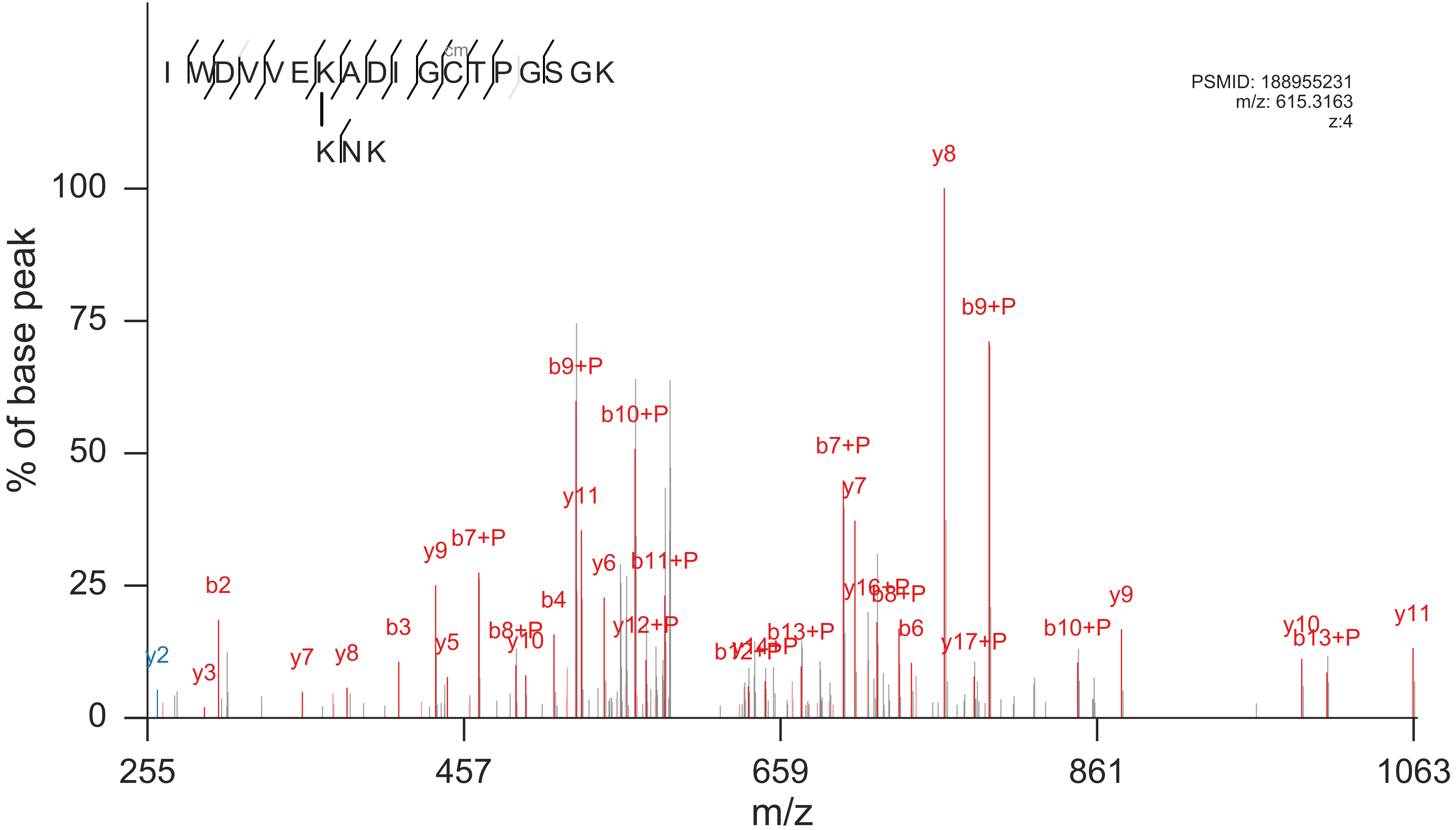

I W D V V E K A D I G C T P G S G K
cm
K N K
PSMID: 188955231
m/z: 615.3163
z:4
% of base peak
m/z

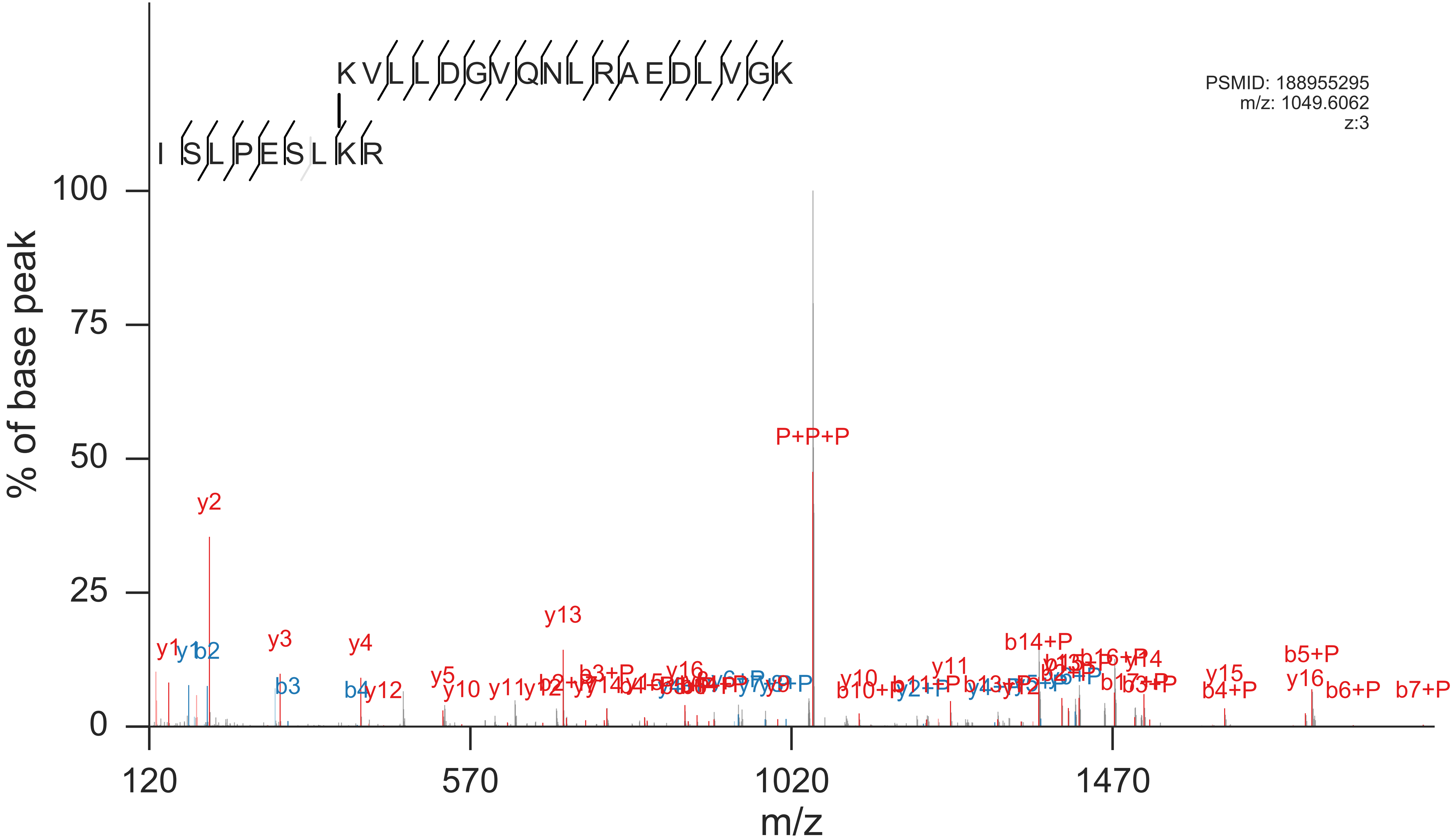
KVLLDGVQNLRAEDLVGK
ISLPESLKR
PSMID: 188955295
m/z: 1049.6062
z:3
% of base peak
m/z
P+P+P
y2
y13

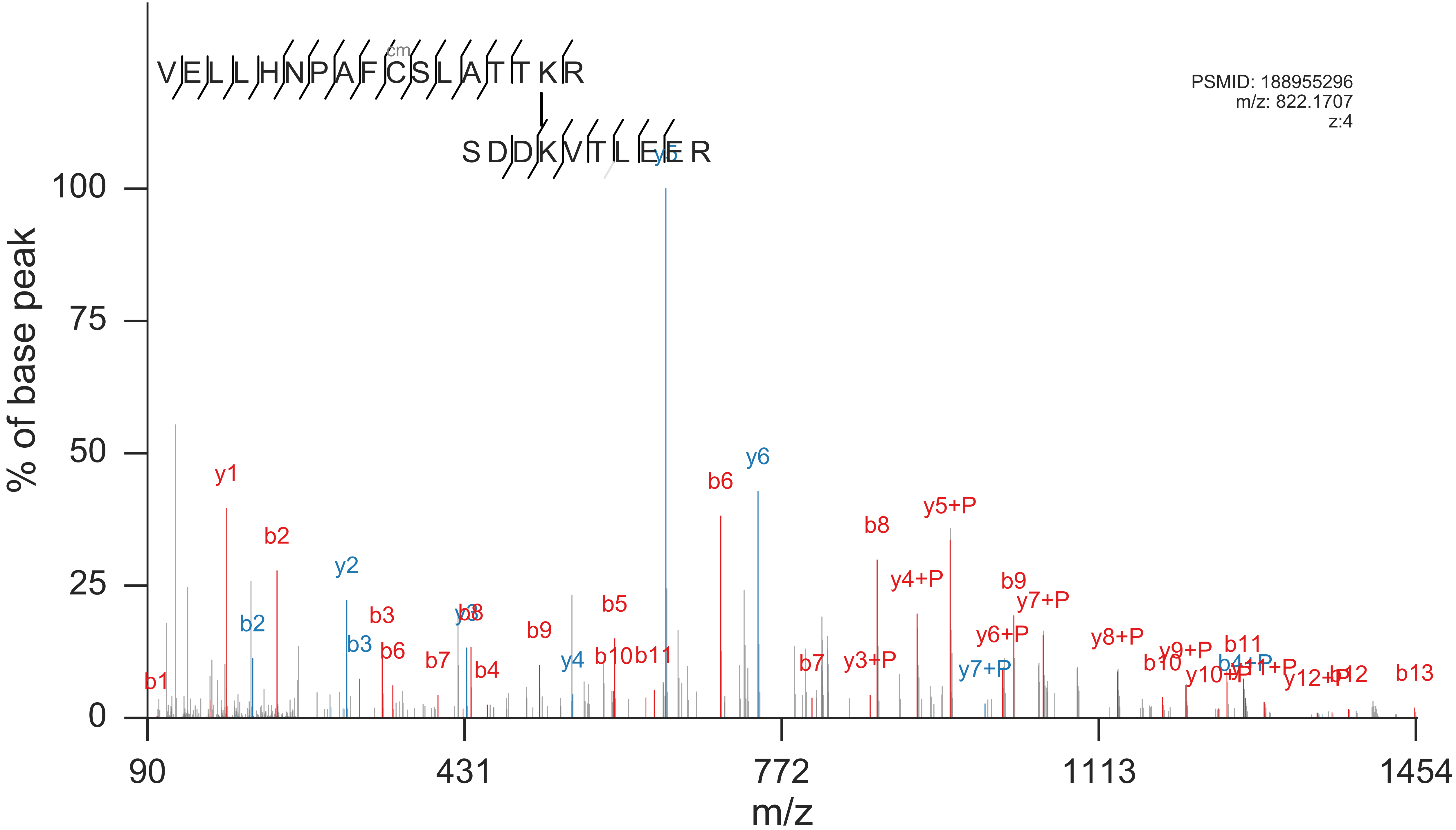

VELLHNPAFCSLATTKR
cm
SDDKVTLEER
PSMID: 188955296
m/z: 822.1707
z:4
% of base peak
m/z
100
75
50
25
0
90
431
772
1113
1454

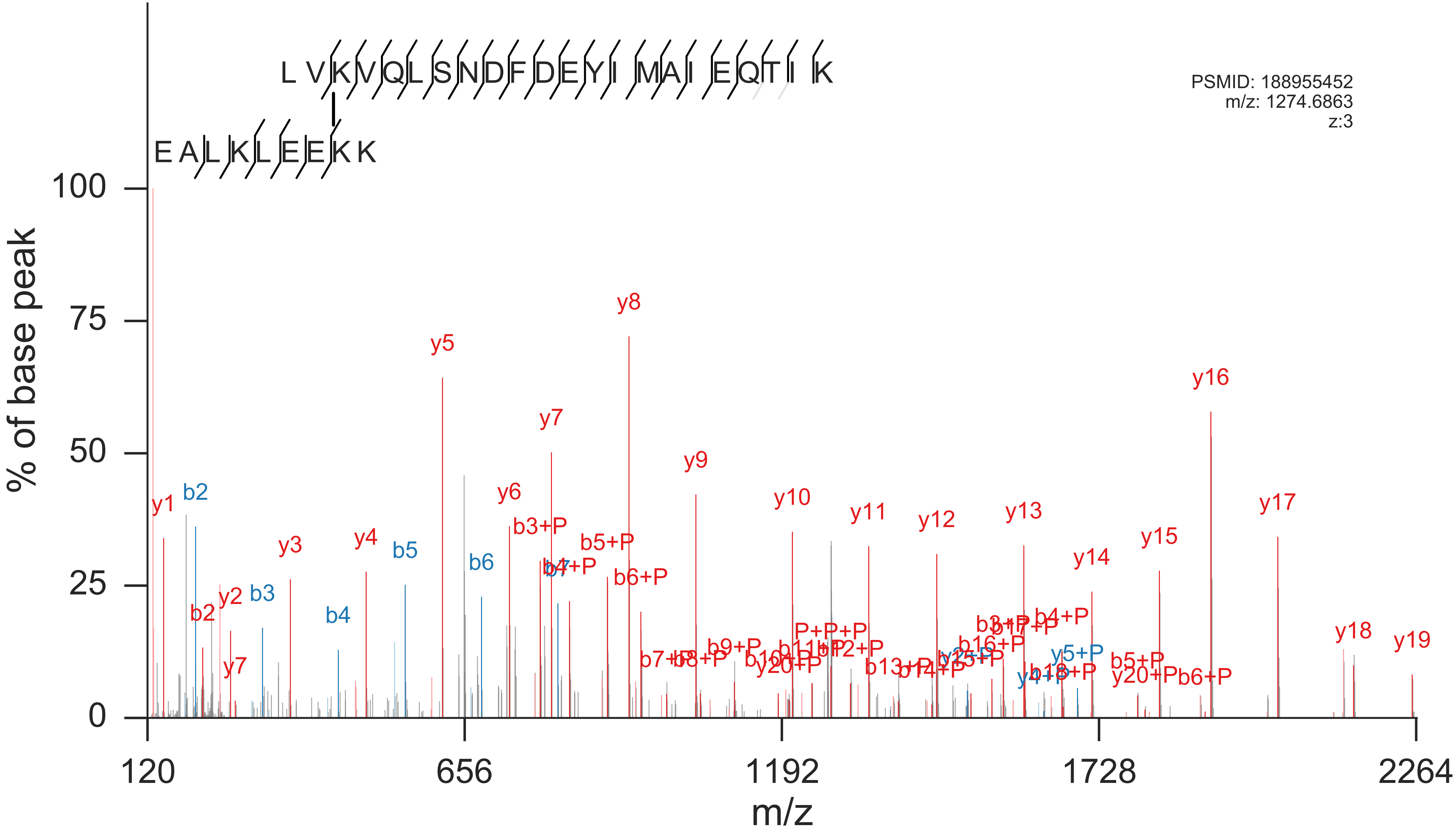

L V K V Q L S N D F D E Y I M A I E Q T I K
E A L K L E E K K
PSMID: 188955452
m/z: 1274.6863
z:3
% of base peak
m/z

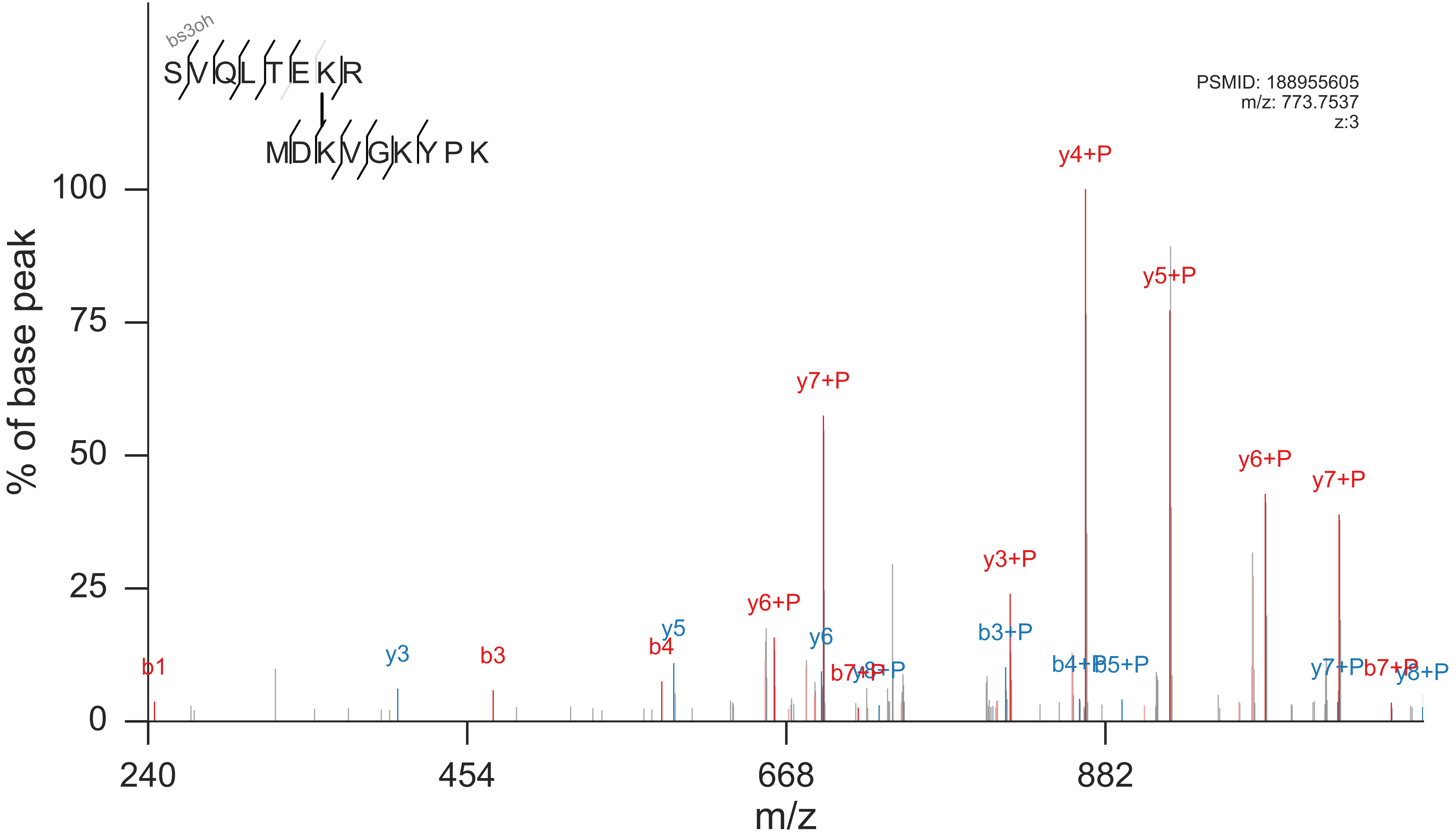

bs3oh
SVQLTEKR
MDKVGKYPK
PSMID: 188955605
m/z: 773.7537
z:3
% of base peak
m/z

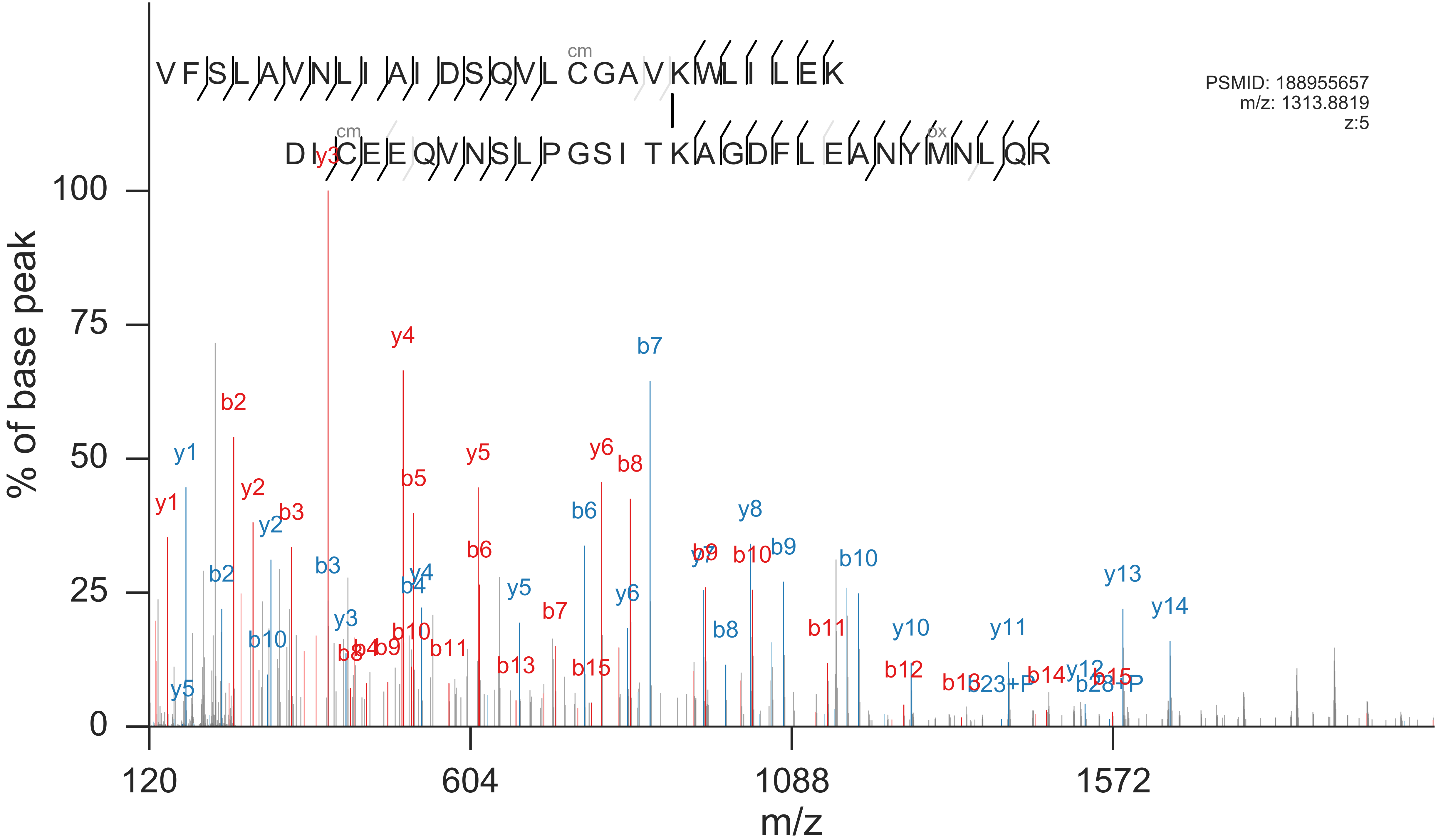
VFSLAVNLIAIDSQVLCGAVKWLILEK
DICEEQVNSLPGSITKAGDFLEANYMNLQR
cm
ox
PSMID: 188955657
m/z: 1313.8819
z:5
% of base peak
m/z
0
25
50
75
100
120
604
1088
1572

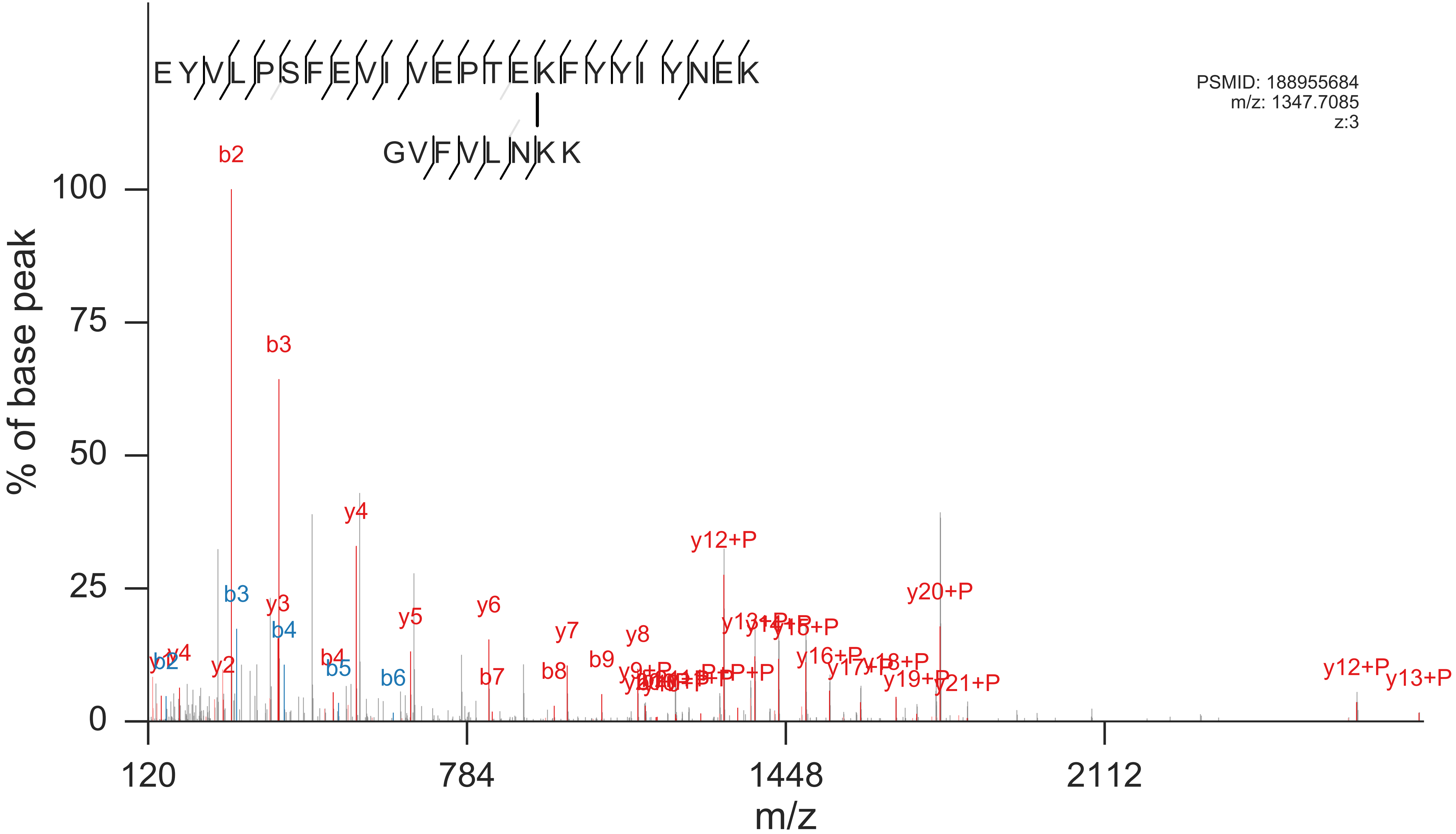

EYVLPSFEVIVEPTEKFYYIYNEK
GVFVLNKK
PSMID: 188955684
m/z: 1347.7085
z:3
% of base peak
m/z

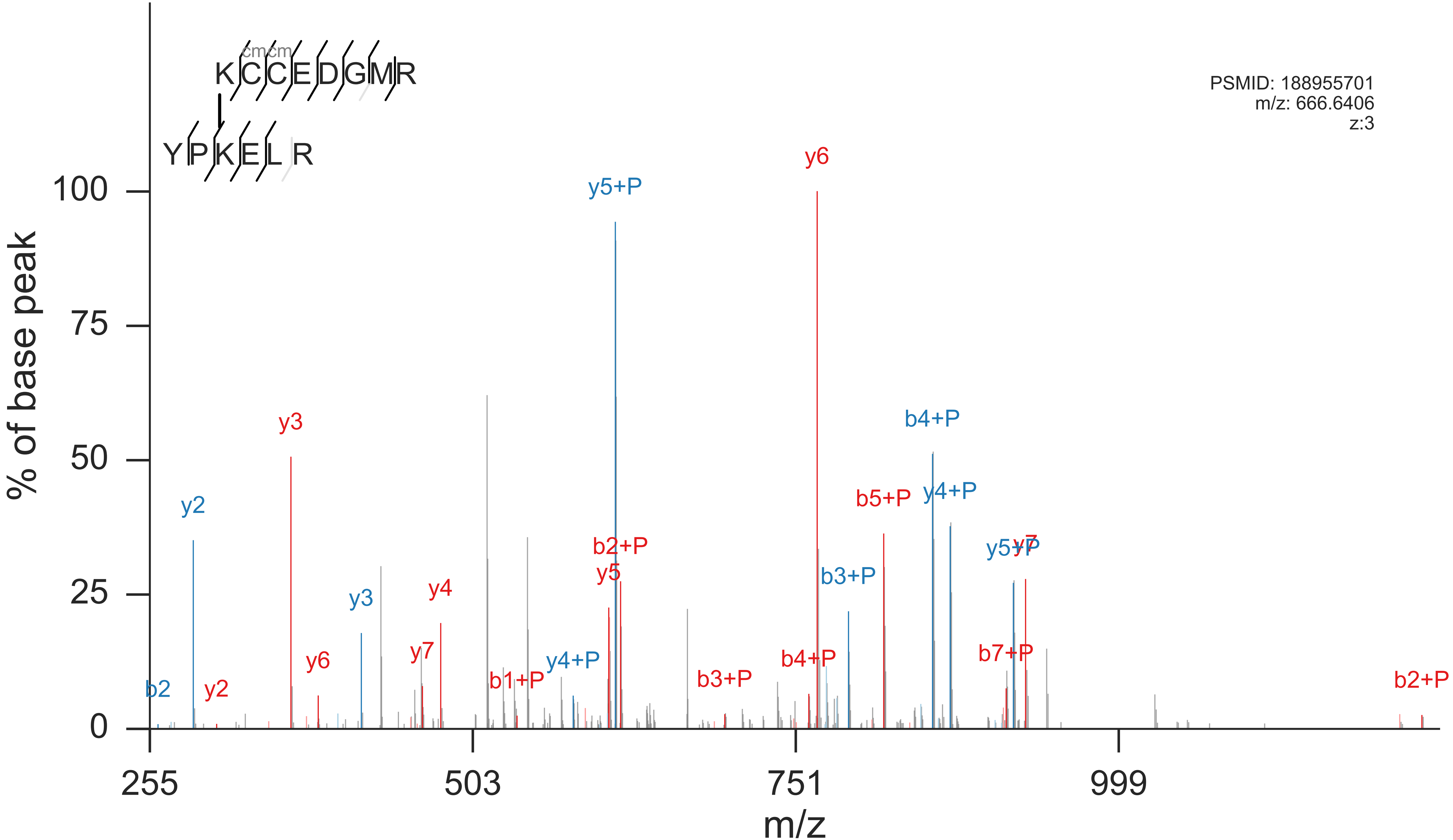
KCCEDGMR
cm cm
YPKELR
PSMID: 188955701
m/z: 666.6406
z:3
% of base peak
100
75
50
25
0
255
503
751
999
m/z
y6
y5+P
y3
b4+P
b5+P
y4+P
y2
y5+P
y7
b2+P
y5
b3+P
y3
y4
y7
y6
b4+P
b7+P
b2
y2
b1+P
y4+P
b3+P
b2+P

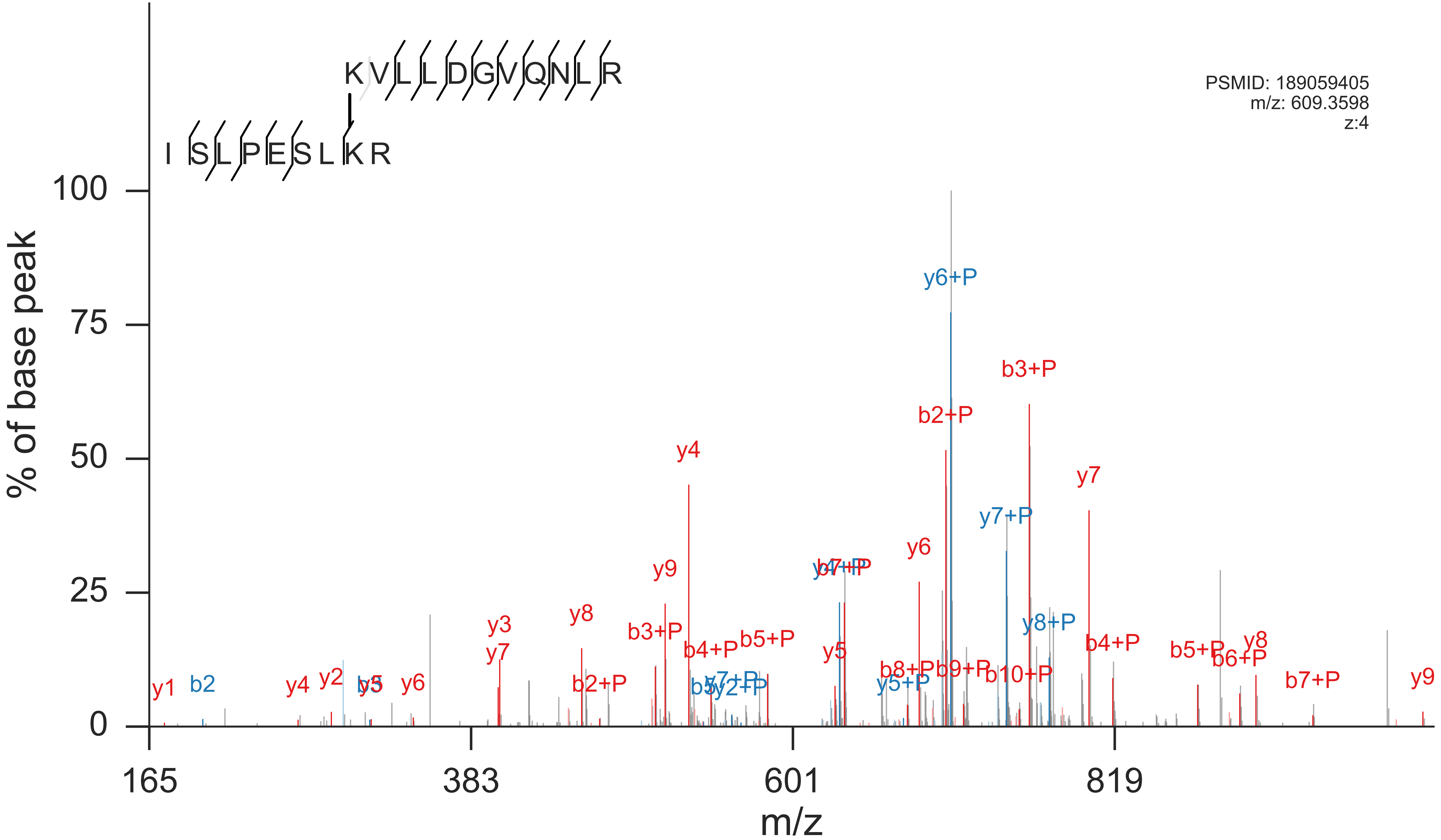

KVLLDGVQNLR
ISLPESLKR
PSMID: 189059405
m/z: 609.3598
z:4
% of base peak
100
75
50
25
0
165
383
601
819
m/z
y1
b2
y4
y2
y5
y6
y3
y7
y8
b2+P
b3+P
y9
y4
b4+P
b5+P
b5
y7+P
y2+P
y5
b7+P
y4+P
y6
b8+P
b9+P
b10+P
y5+P
b2+P
y6+P
y7+P
b3+P
y8+P
y7
b4+P
b5+P
b6+P
y8
b7+P
y9

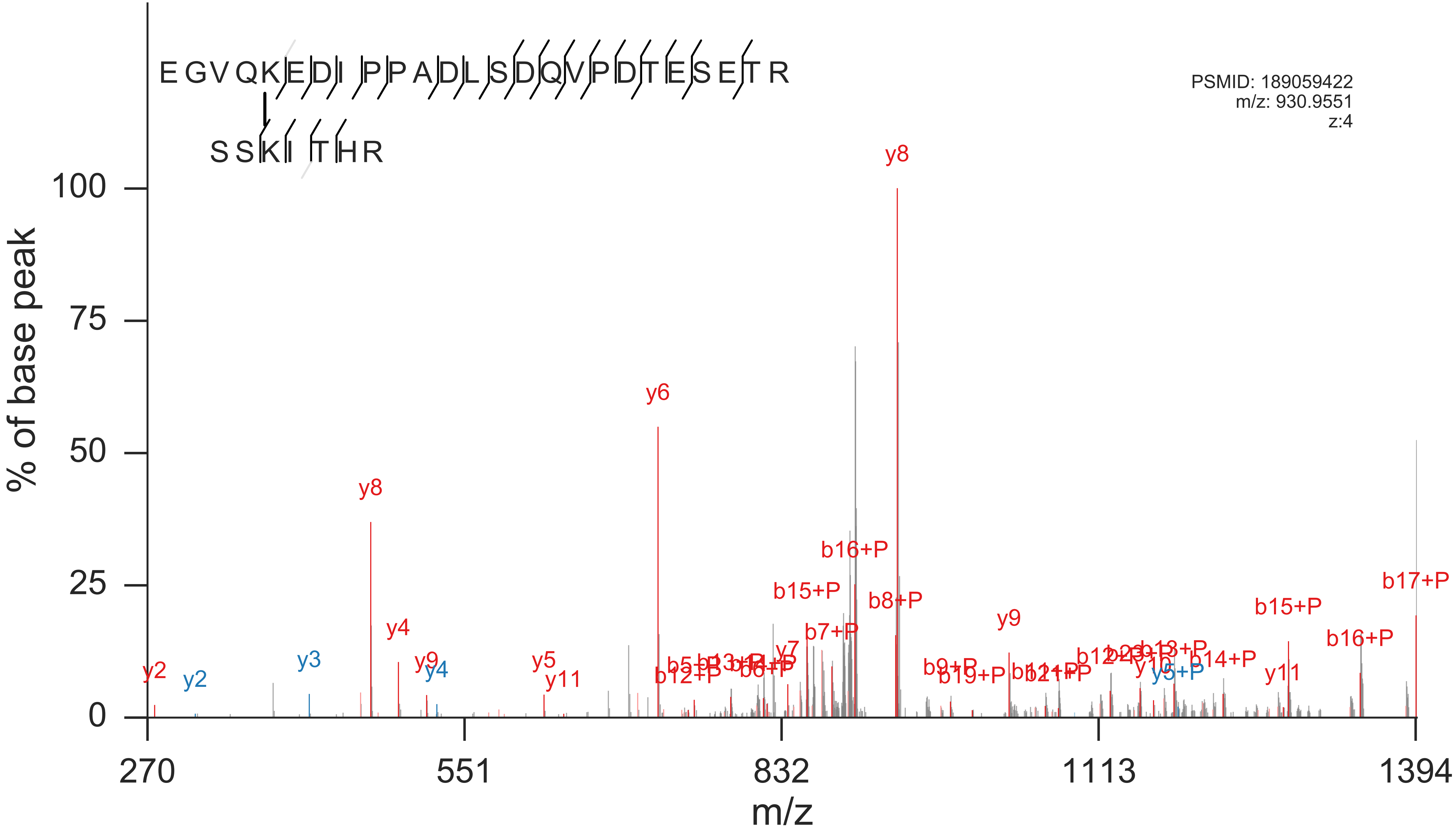
EGVQKEDIPPADLSDQVPDTESETR
SSKITHR
PSMID: 189059422
m/z: 930.9551
z:4
% of base peak
m/z
0
25
50
75
100
270
551
832
1113
1394
y8
y6
y8
y4
y2
y2
y3
y9
y4
y5
y11
y7
y9
y11
y10
y5+P
b16+P
b15+P
b8+P
b7+P
b17+P
b15+P
b16+P
b14+P
b13+P
b12+P
b9+P
b19+P
b6+P
b5+P

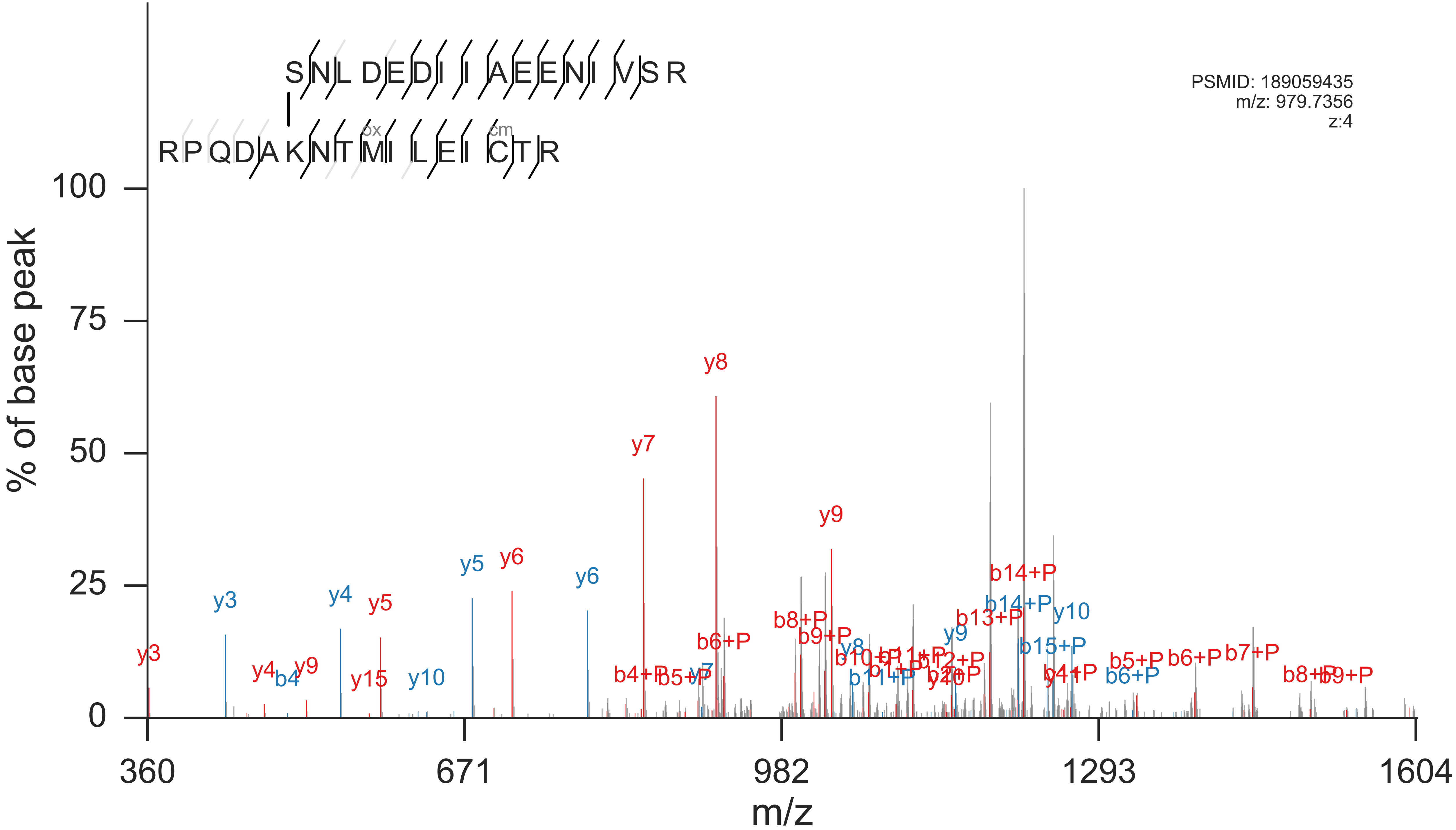
SNLDEDIIAEENIVSR
RPQDAKNTMILEICTR
ox
cm
PSMID: 189059435
m/z: 979.7356
z:4
% of base peak
m/z
0
25
50
75
100
360
671
982
1293
1604
y3
y4
y5
y6
y7
y8
y9
y10
y15
b4
b6+P
b8+P
b9+P
b13+P
b14+P
b15+P
b5+P
b6+P
b7+P
b11+P
y11

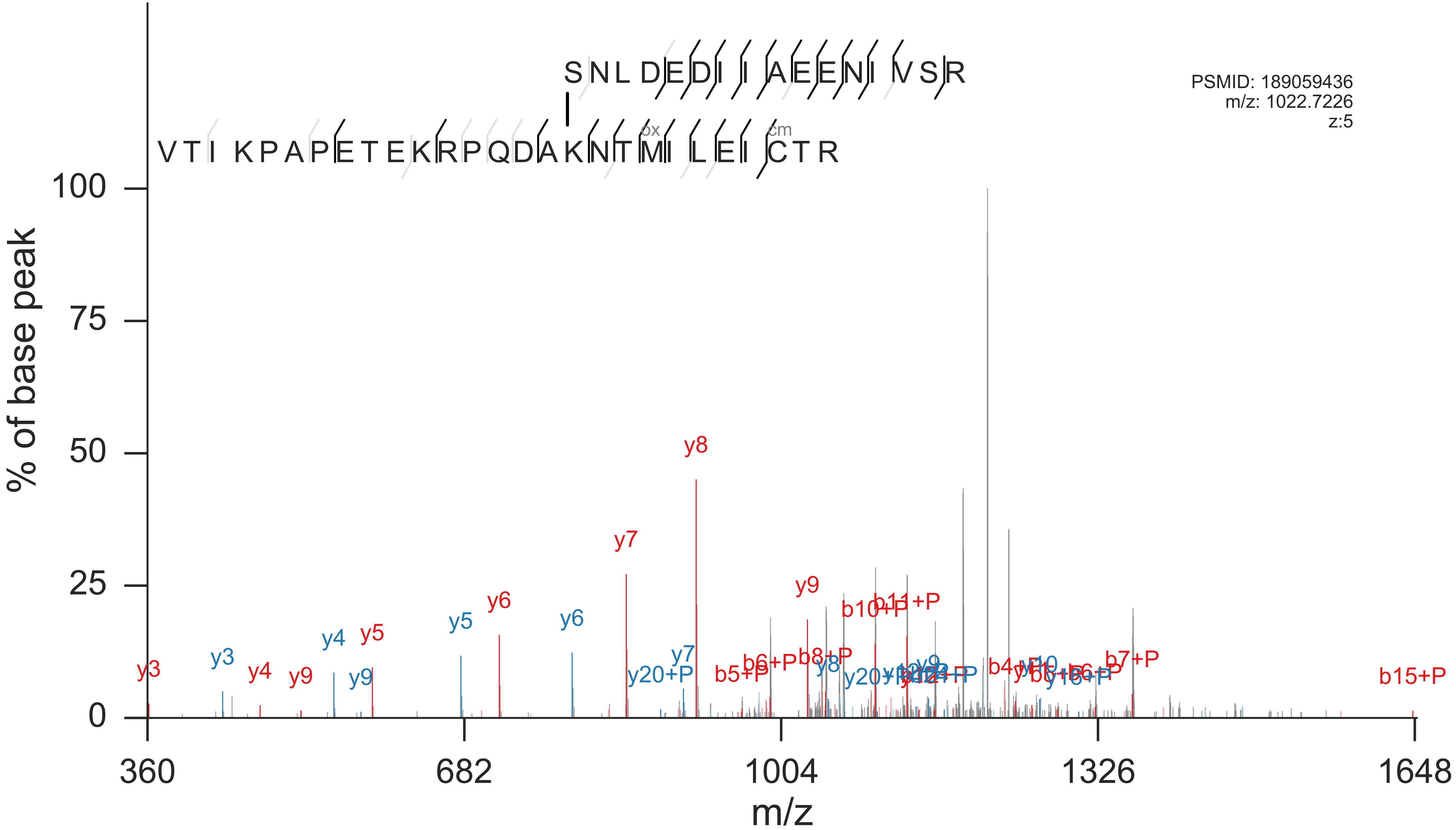

SNLDEDIIAEENIVSR
VTIKPAPETEKRPQDAKNTMILEICTR
ox
cm
PSMID: 189059436
m/z: 1022.7226
z:5
% of base peak
m/z
0
25
50
75
100
360
682
1004
1326
1648
y3
y4
y5
y6
y7
y8
y9
b5+P
b6+P
b8+P
b10+P
b11+P
b4+P
b7+P
b15+P
y20+P
y16+P

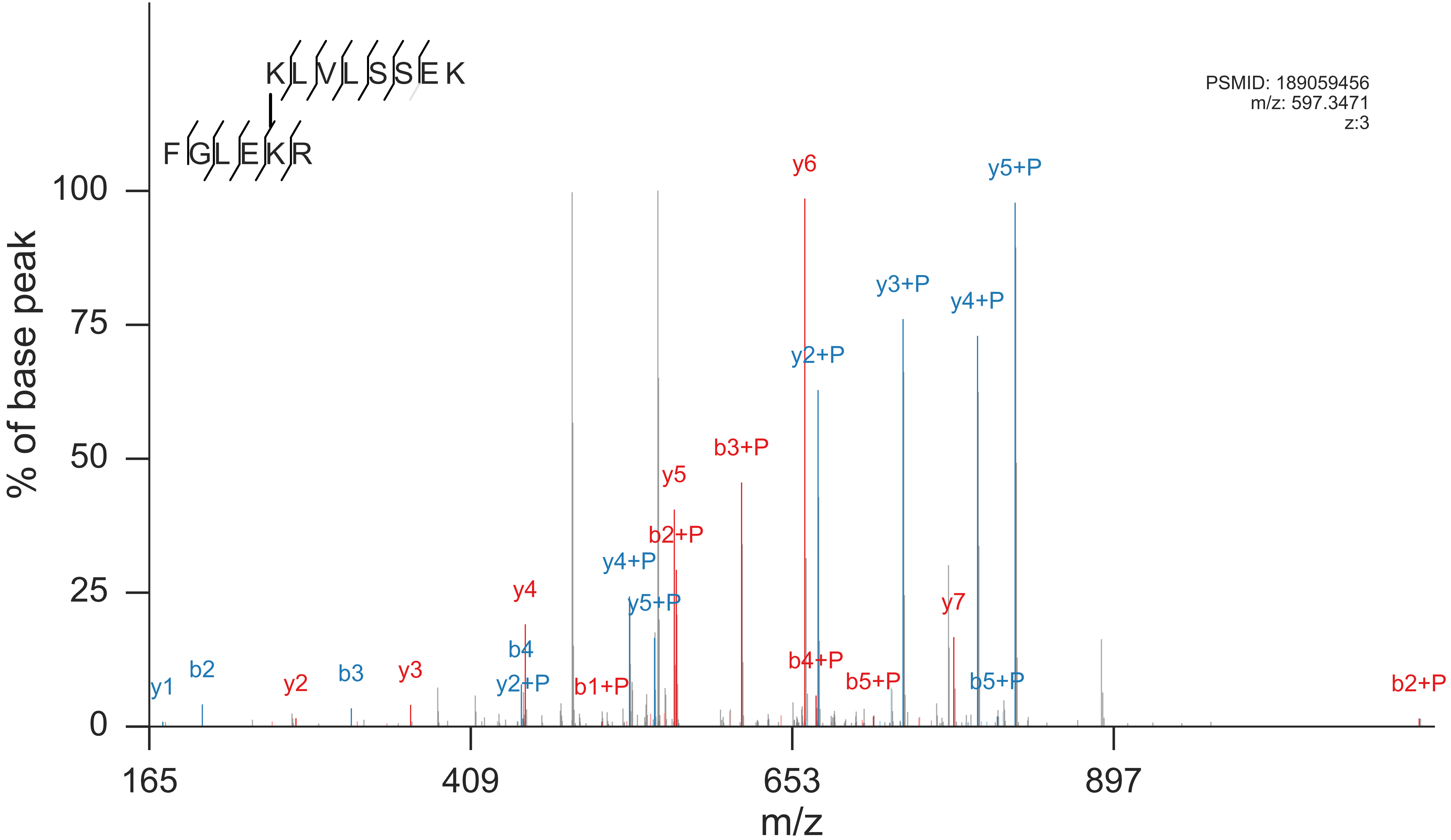

KLVLSSEK
FGLEKR
PSMID: 189059456
m/z: 597.3471
z:3
% of base peak
100
75
50
25
0
165
409
653
897
m/z
y1
b2
y2
b3
y3
b4
y2+P
y4
b1+P
y4+P
y5+P
y5
b2+P
b3+P
y6
y2+P
b4+P
b5+P
y3+P
y4+P
y7
b5+P
y5+P
b2+P

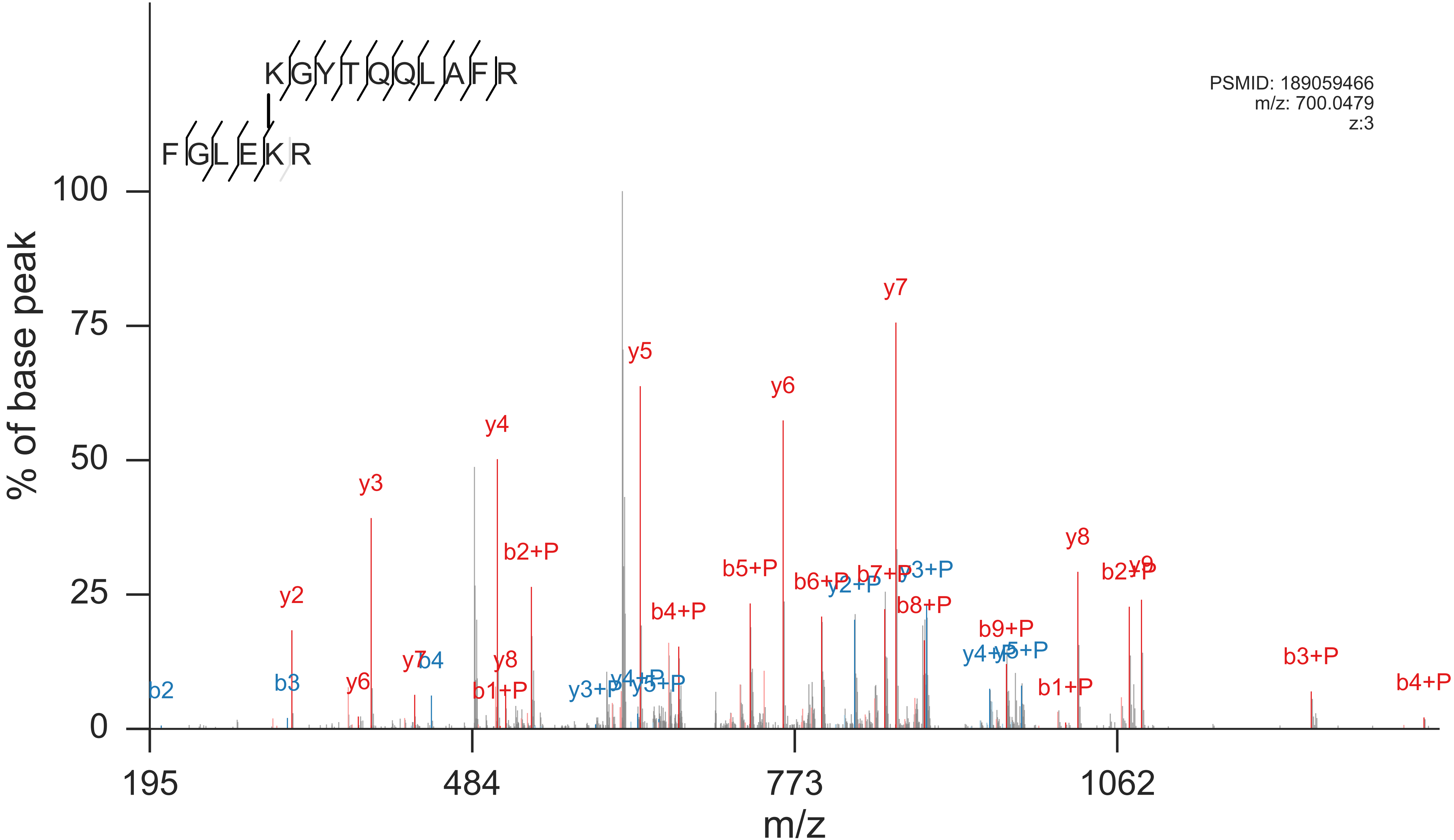
KGYTQQLAFR
FGLEKR
PSMID: 189059466
m/z: 700.0479
z:3
% of base peak
100
75
50
25
0
195
484
773
1062
m/z
y2
y3
y4
y5
y6
y7
y8
y9
b2+P
b4+P
b5+P
b6+P
b7+P
b8+P
b9+P
b1+P
b3+P
y6
y7
y8
b1+P
b2
b3
b4
y3+P
y4+P
y5+P
y2+P
y4+P
y5+P

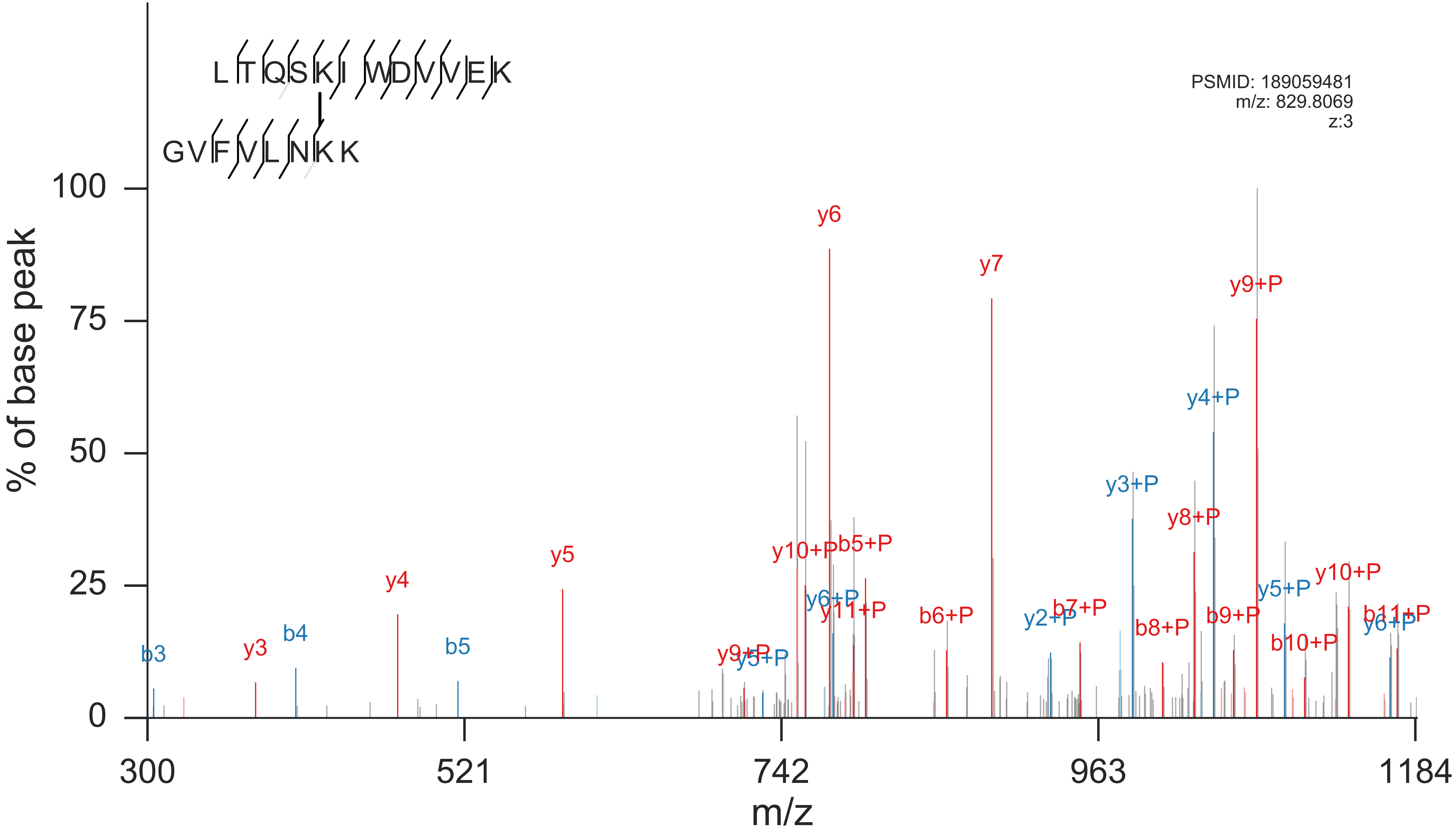

LTQSKIWDVVEK
GVFVLNKK
PSMID: 189059481
m/z: 829.8069
z:3
% of base peak
m/z
0
25
50
75
100
300
521
742
963
1184
y6
y7
y9+P
y4+P
y3+P
y8+P
y10+P
b5+P
y5
y4
y3
b3
b4
b5
y9+P
y5+P
y6+P
y11+P
b6+P
y2+P
b7+P
b8+P
b9+P
y5+P
y10+P
b10+P
b11+P
y6+P

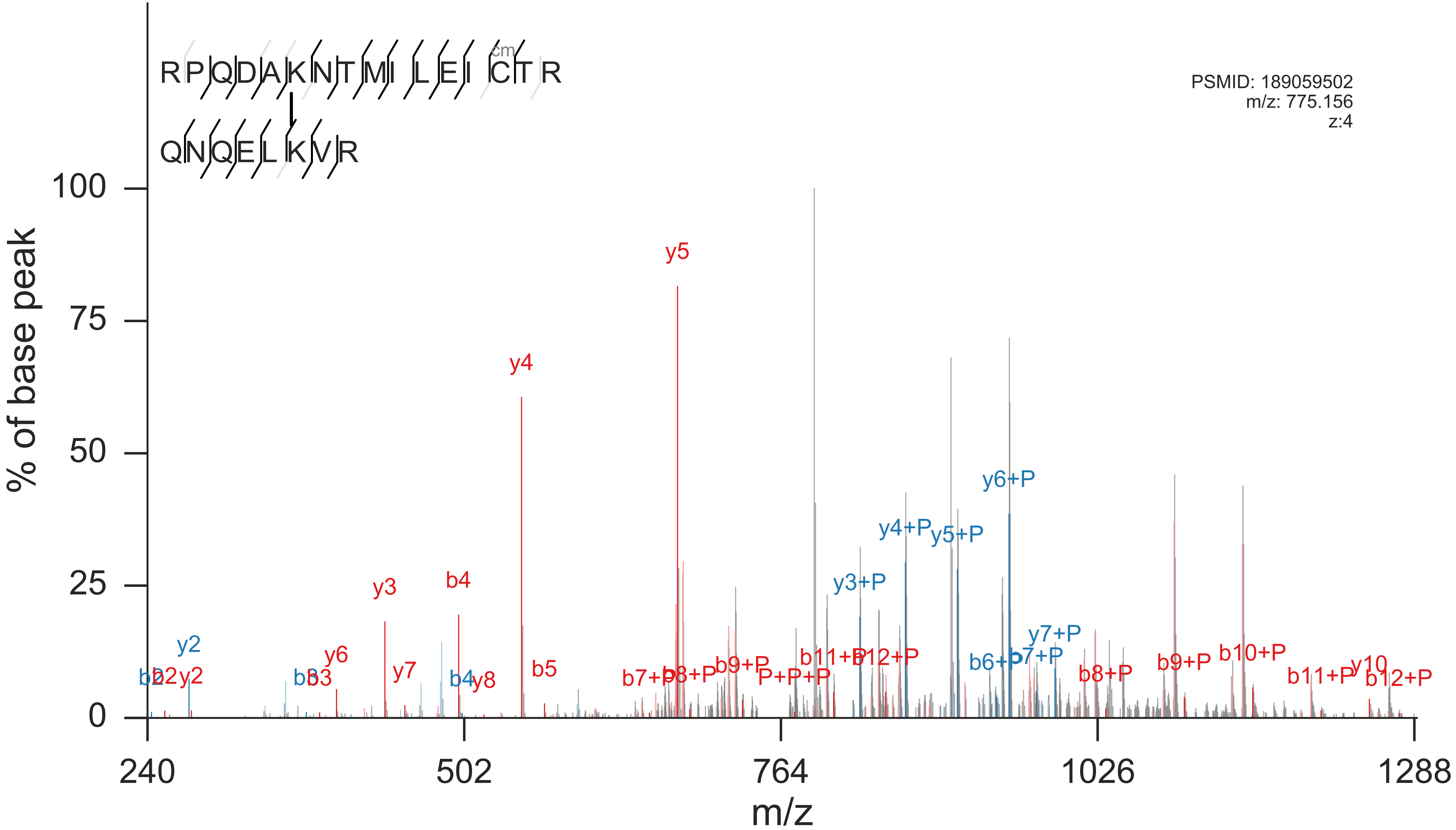
RPQDAKNTMILEICTR
cm
QNQELKVR
PSMID: 189059502
m/z: 775.156
z:4
% of base peak
m/z
y5
y4
y3
b4
y6+P
y4+P
y5+P
y3+P
y7+P
y2
y6
y7
y8
b5
b9+P
b10+P
b11+P
y10
b12+P
b8+P

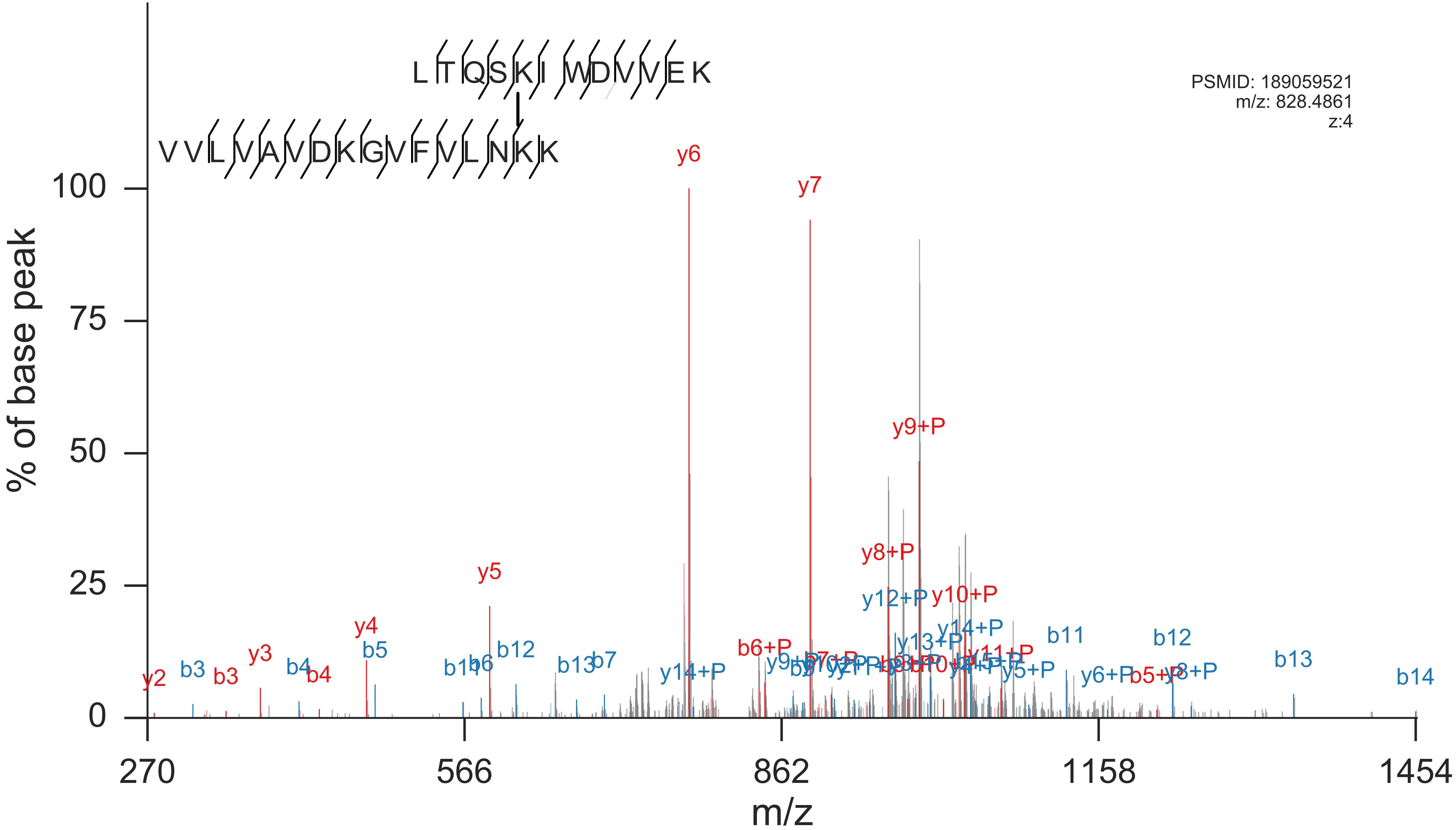

PSMID: 189059521
m/z: 828.4861
z:4
LTQSKIWDVVEK
VVLVAVDKGVFVLNKK
% of base peak
m/z

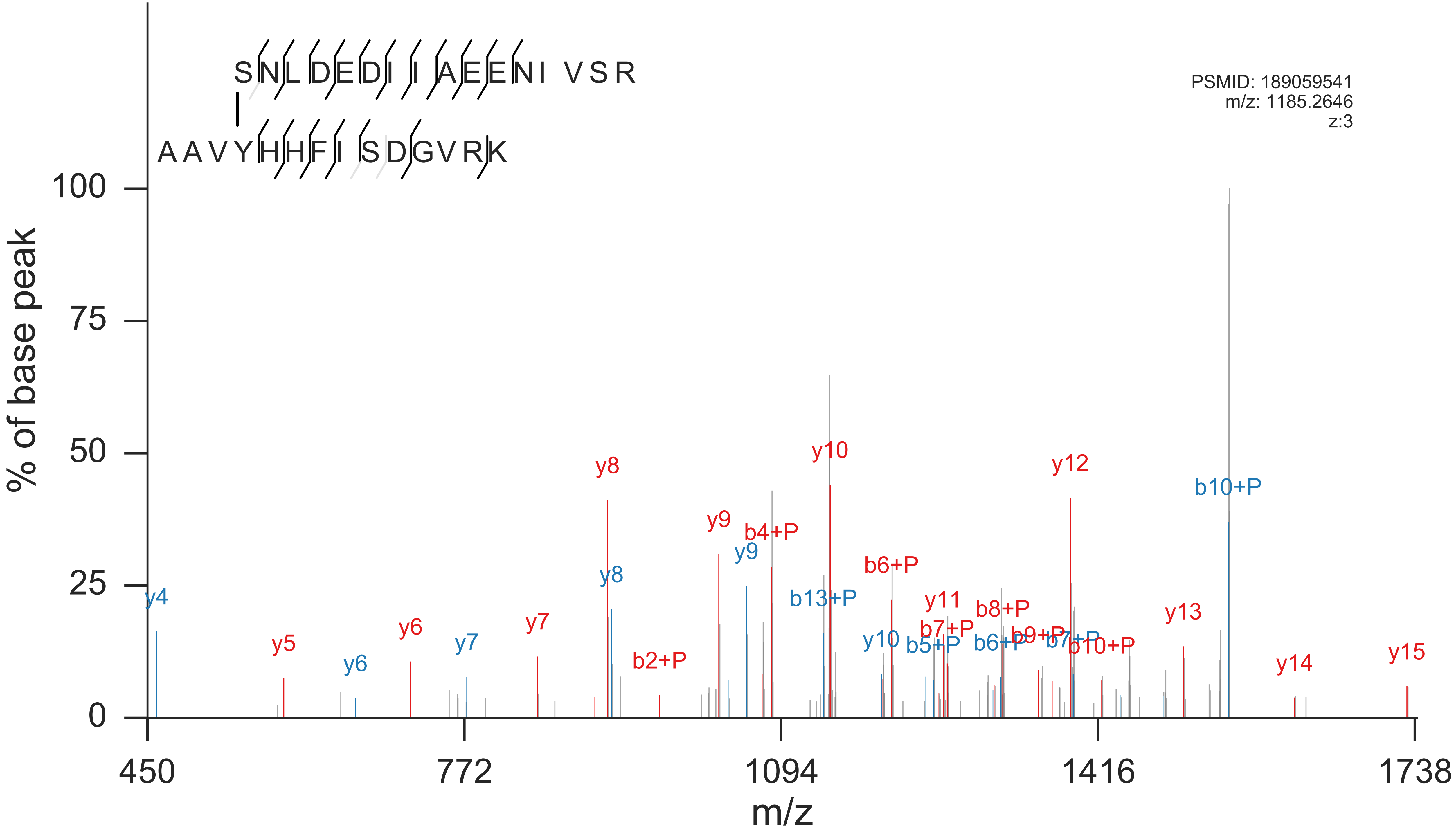
SNLDEDIIAEENIVSR
AAVYHHFISDGVRK
PSMID: 189059541
m/z: 1185.2646
z:3
% of base peak
m/z
0
25
50
75
100
450
772
1094
1416
1738
y4
y5
y6
y6
y7
y7
y8
y8
b2+P
y9
y9
b4+P
y10
b13+P
y10
b6+P
b5+P
y11
b7+P
b6+P
b8+P
b9+P
b7+P
y12
b10+P
y13
b10+P
y14
y15

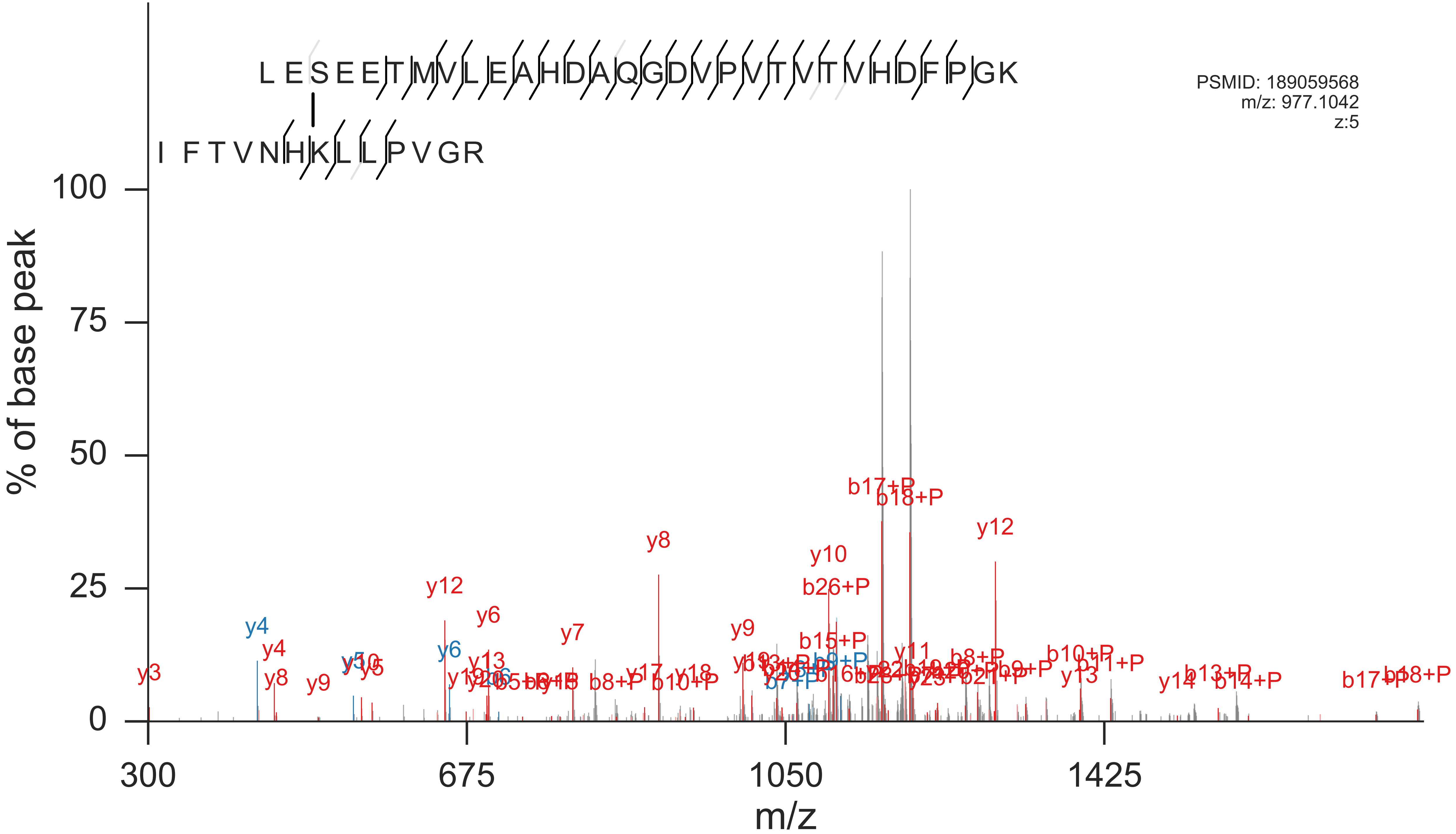
PSMID: 189059568
m/z: 977.1042
z:5
LESEETMVLEAHDAQGDVPVTVTVHDFPGK
IFTVNHKLLPVGR
% of base peak
m/z

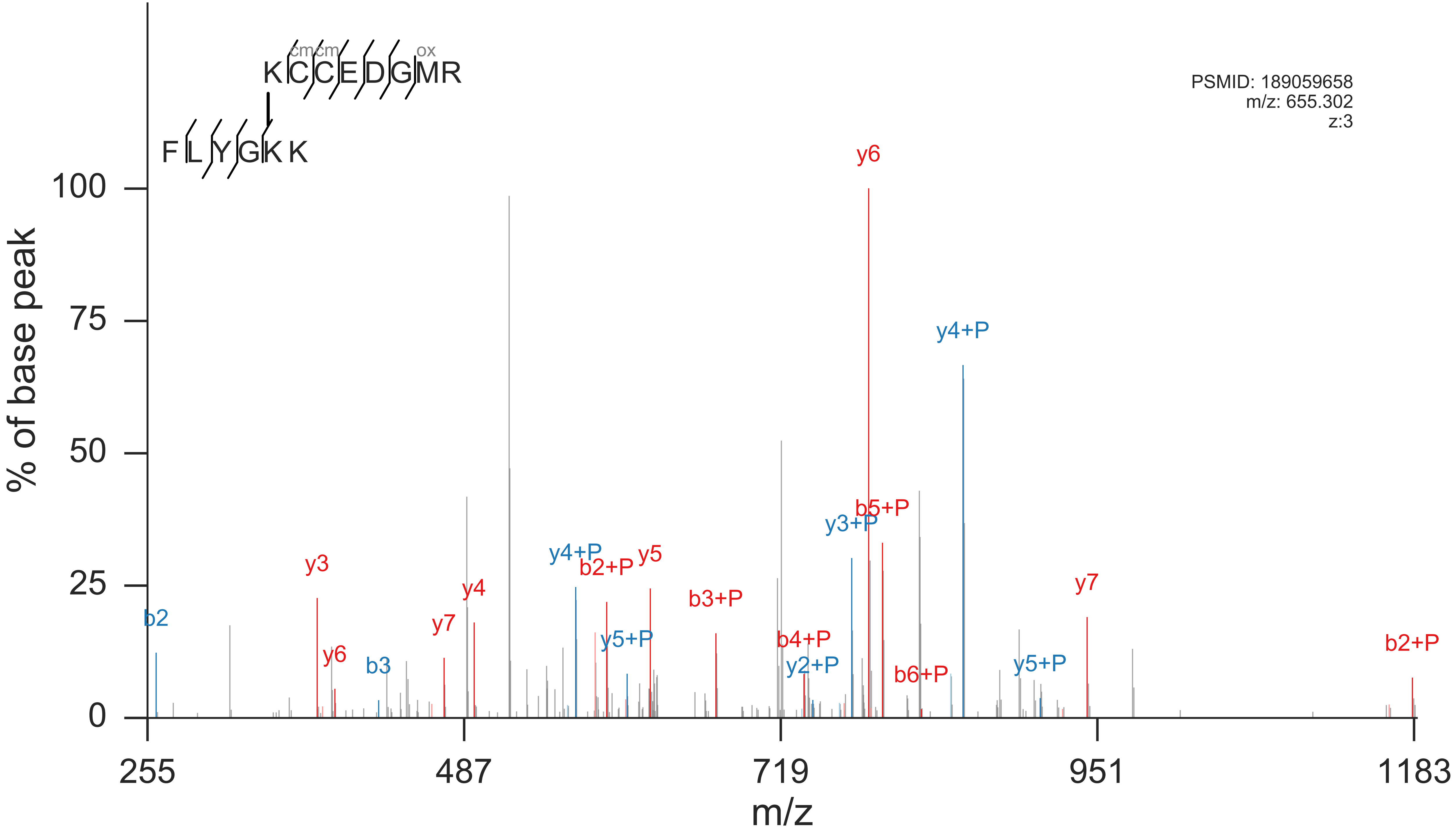

KCCEDGMR
cm cm ox
FLYGKK
PSMID: 189059658
m/z: 655.302
z:3
% of base peak
m/z
y6
y4+P
b5+P
y3+P
y4+P
b2+P
y5
y3
y4
y7
b3+P
y7
b2
y6
b3
y5+P
b4+P
y2+P
b6+P
y5+P
b2+P
100
75
50
25
0
255
487
719
951
1183

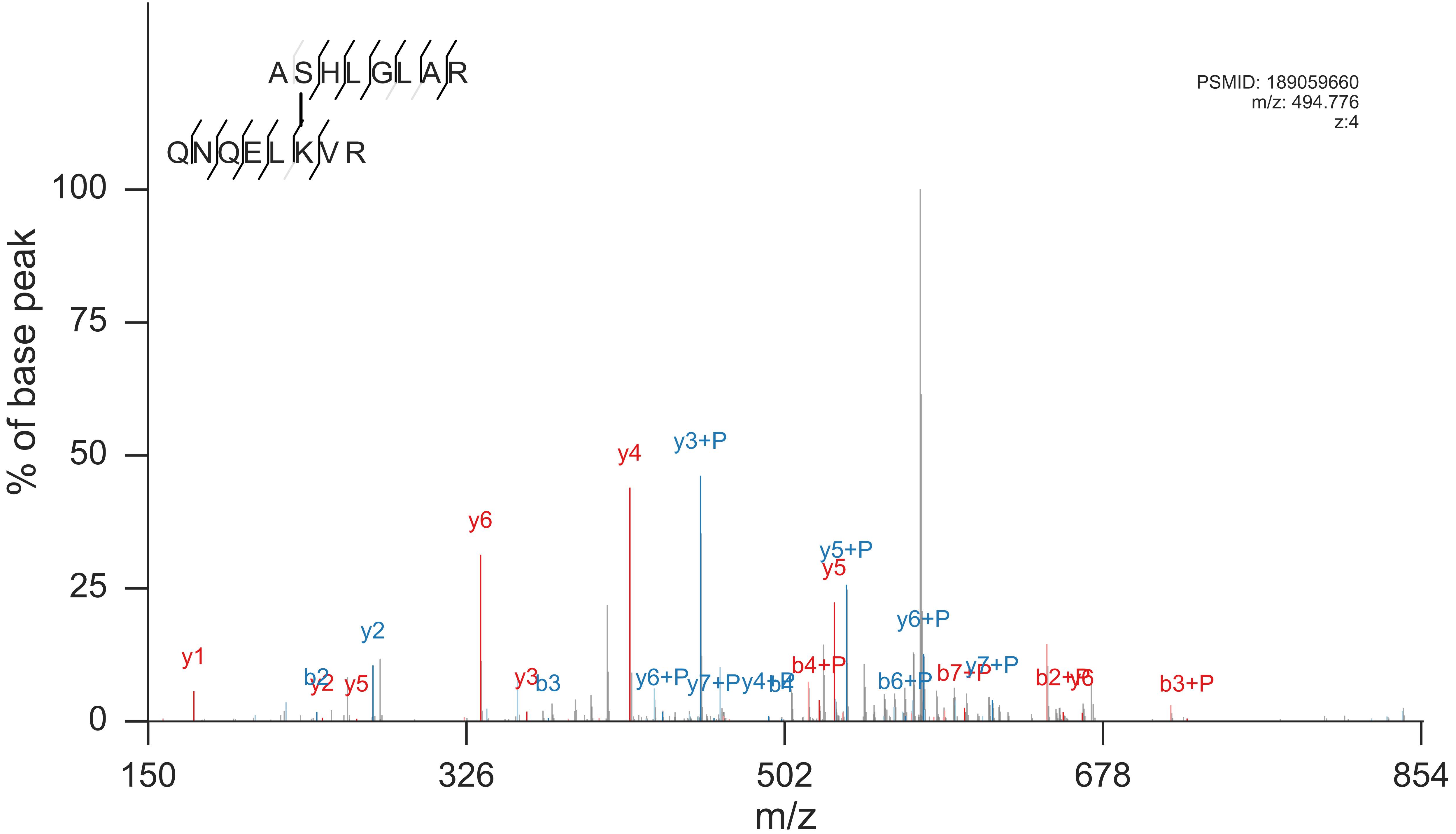

ASHLGLAR
QNQELKVR
PSMID: 189059660
m/z: 494.776
z:4
% of base peak
m/z
y1
b2
y2
y5
y2
y6
y3
b3
y4
y6+P
y3+P
y7+P
y4+P
b4
b4+P
y5+P
y5
b6+P
y6+P
b7+P
y7+P
b2+P
y6
b3+P
0
25
50
75
100
150
326
502
678
854

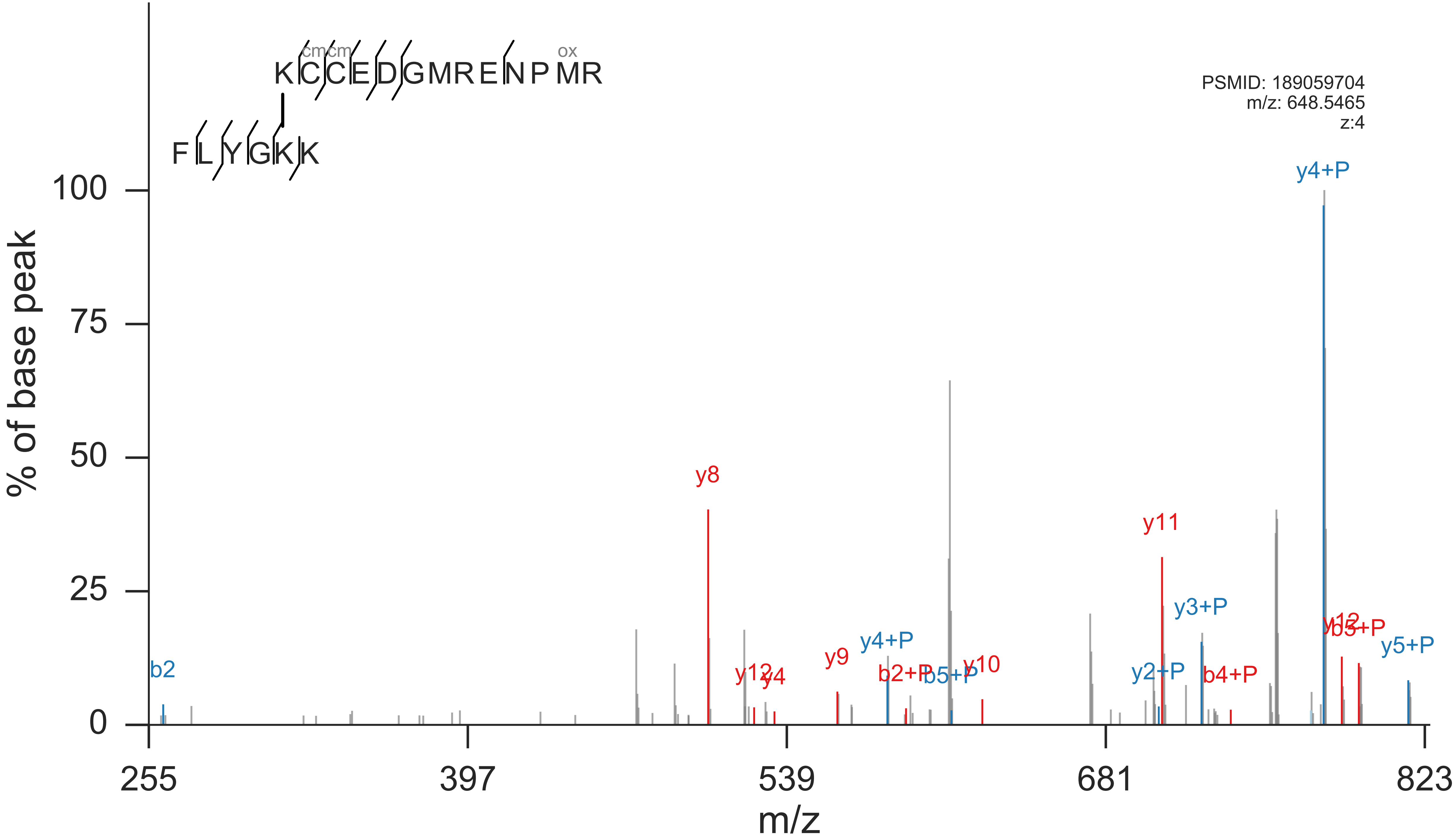

KCCEDGMRENPMR
cm cm ox
FLYGKK
PSMID: 189059704
m/z: 648.5465
z:4
% of base peak
100
75
50
25
0
255
397
539
681
823
m/z
y4+P
y8
y11
y3+P
y12
b3+P
y5+P
y4+P
b2
y12
y4
y9
b2+P
b5+P
y10
y2+P
b4+P

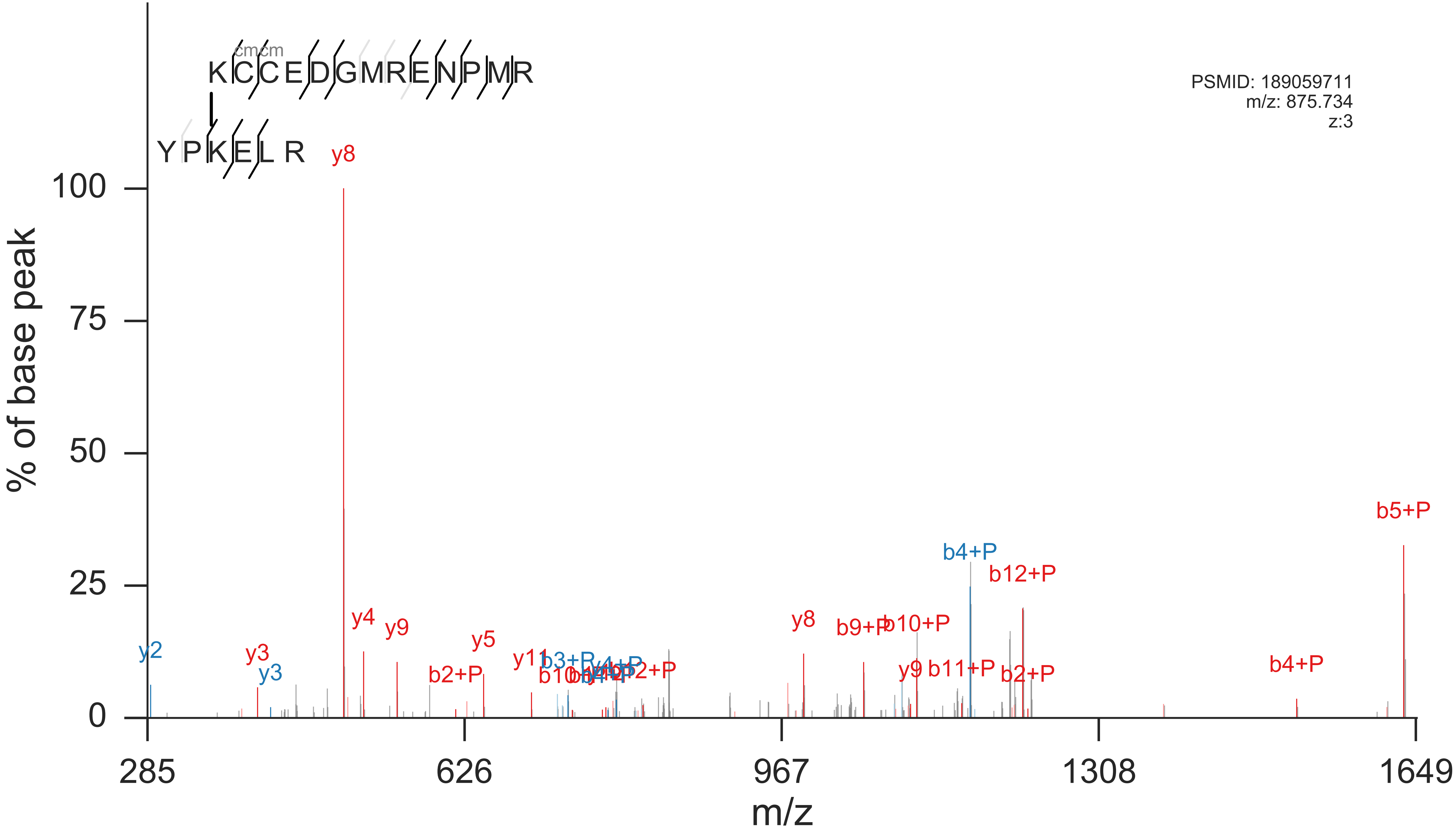

KCCEDGMRENPMR
cmcm
YPKELR
PSMID: 189059711
m/z: 875.734
z:3
% of base peak
m/z
100
75
50
25
0
285
626
967
1308
1649
y8
b5+P
b4+P
b12+P
y4
y9
y5
y8
b9+P
b10+P
y2
y3
y3
b2+P
y11
b3+P
y9
b11+P
b2+P
b4+P

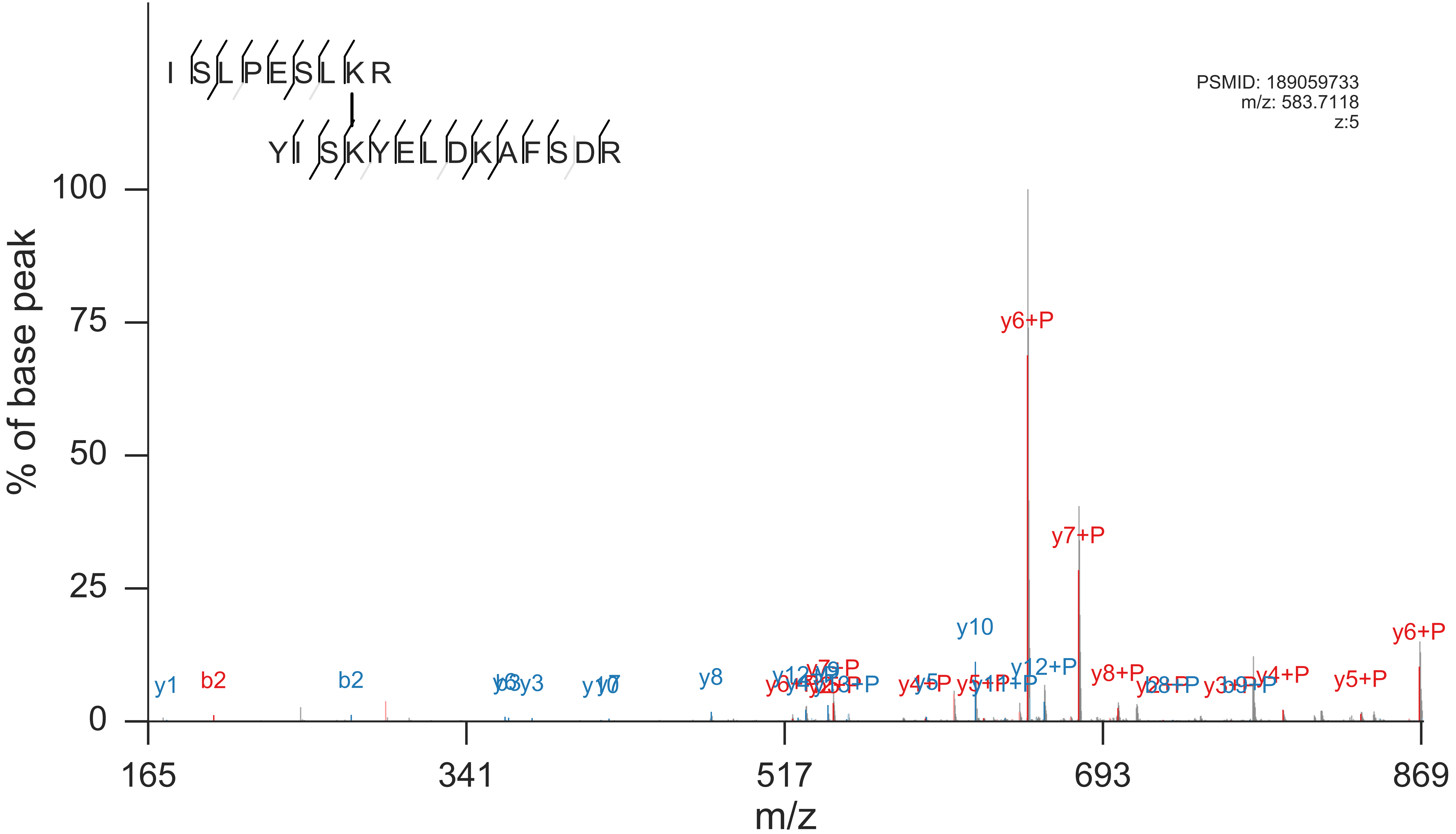

ISLPESLKR
YISKYELDKAFSDR
PSMID: 189059733
m/z: 583.7118
z:5
% of base peak
m/z
y6+P
y7+P
y10
y6+P
y12+P
y8+P
y4+P
y5+P
y1
b2
b2
y8

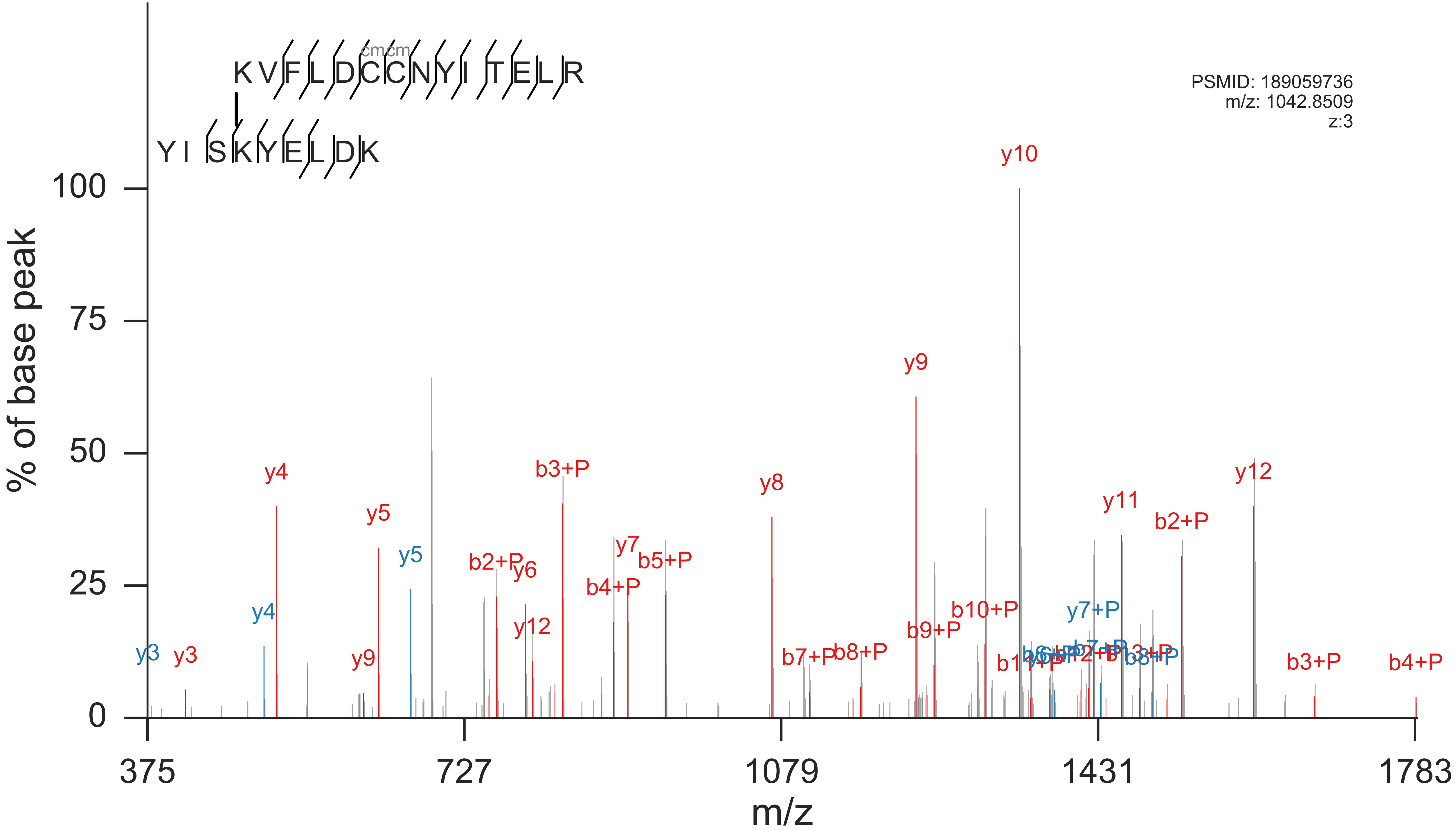
KVFLDCCNYITELR
cmcm
YISKYELDK
PSMID: 189059736
m/z: 1042.8509
z:3
% of base peak
m/z
y10
y9
y12
y11
b2+P
y8
b3+P
y4
y5
y7
b5+P
b4+P
y6
y12
b10+P
b9+P
b8+P
b7+P
y7+P
b3+P
b4+P
y3
y9
100
75
50
25
0
375
727
1079
1431
1783

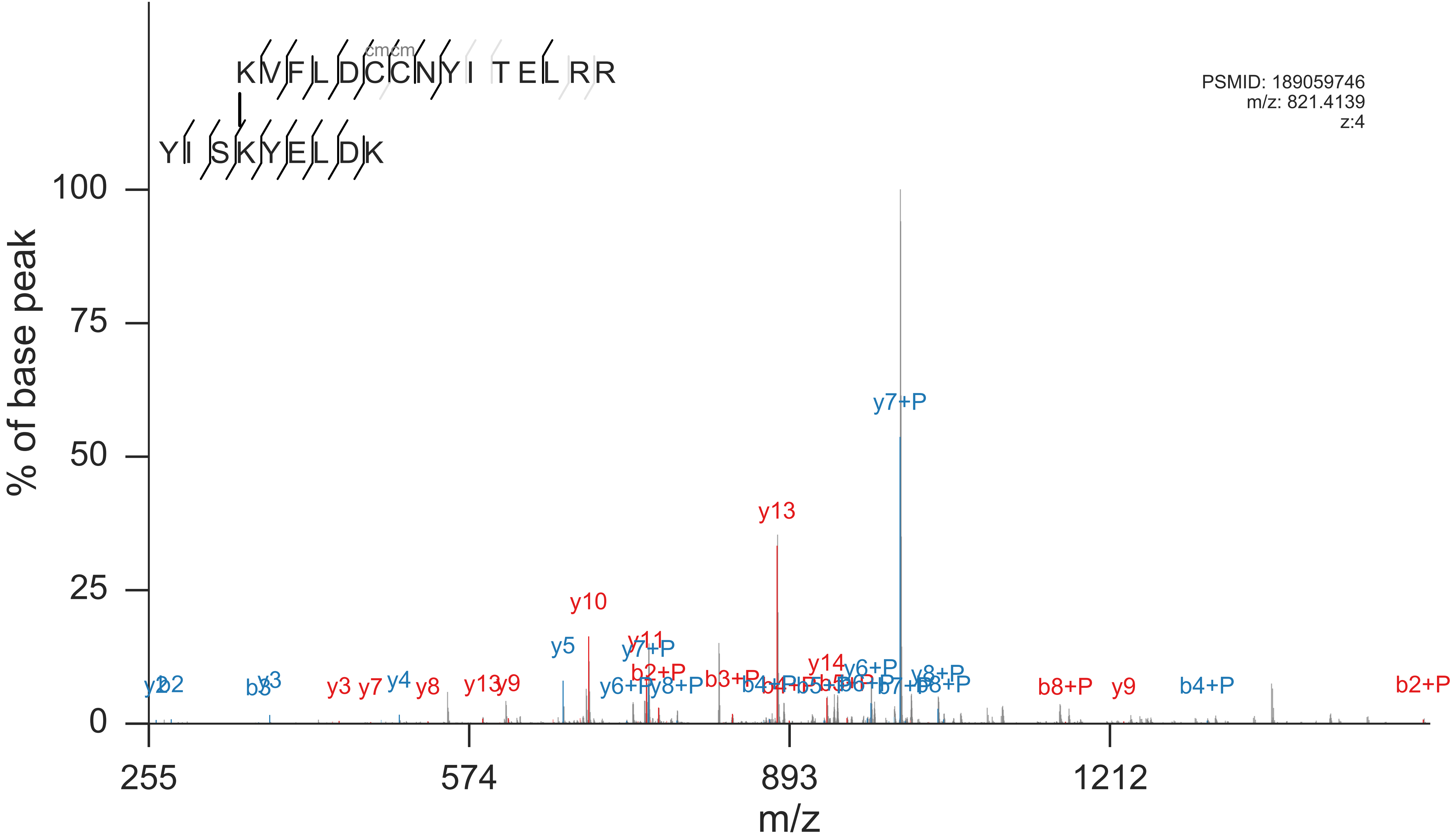

KVFLDCCNYITELRR
YISKYELDK
PSMID: 189059746
m/z: 821.4139
z:4
% of base peak
m/z
y7+P
y13
y10
y5
y11
y14

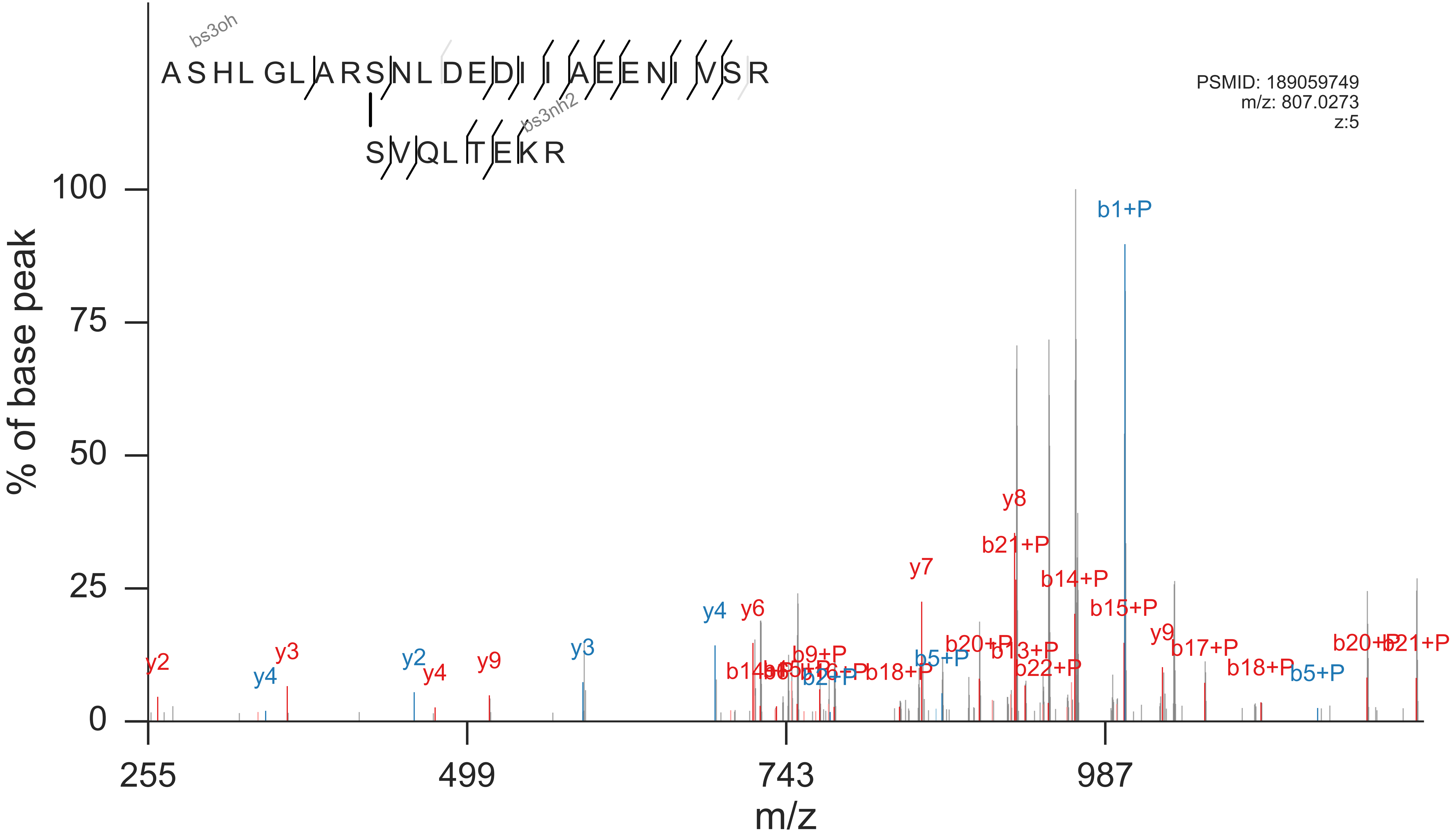
PSMID: 189059749
m/z: 807.0273
z:5
ASHLGLARSNLDEDIIAEENIVSR
SVQLTEKR
bs3oh
bs3nh2
% of base peak
m/z
255
499
743
987
0
25
50
75
100
b1+P
y8
b21+P
y7
b14+P
b15+P
y9
b17+P
b18+P
b5+P
b20+P
b21+P
b13+P
b22+P
y4
y6
y3
y2
y9
b9+P

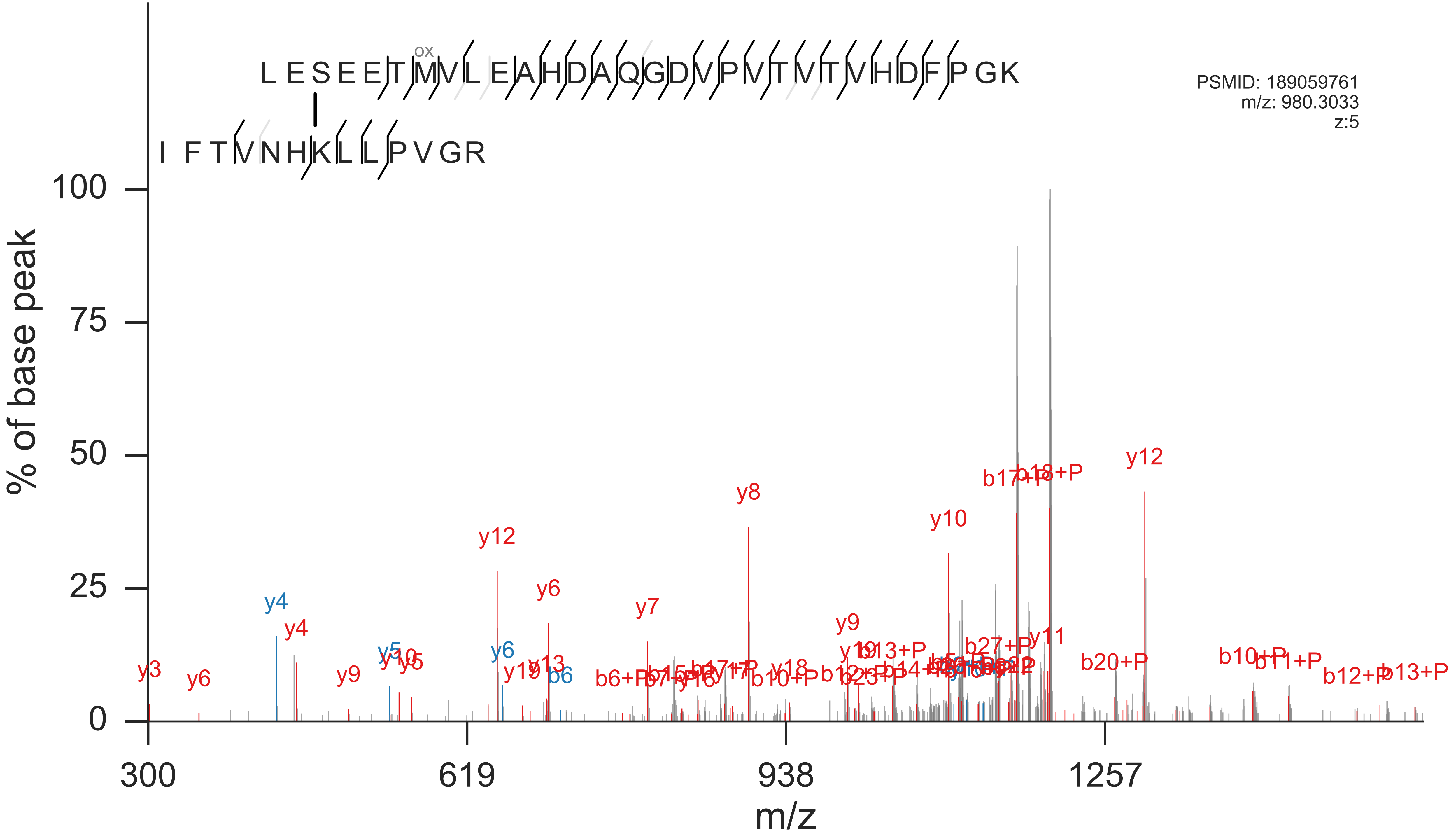
PSMID: 189059761
m/z: 980.3033
z:5
LESEETMVLEAHDAQGDVPVTVTVHDFPGK
IFTVNHKLLPVGR
ox
% of base peak
m/z
0
25
50
75
100
300
619
938
1257
y12
y8
y10
b17+P
b18+P
y6
y7
y9
y11
y4
b20+P
b10+P
b11+P
b12+P
b13+P
b6
y6
y5
y3

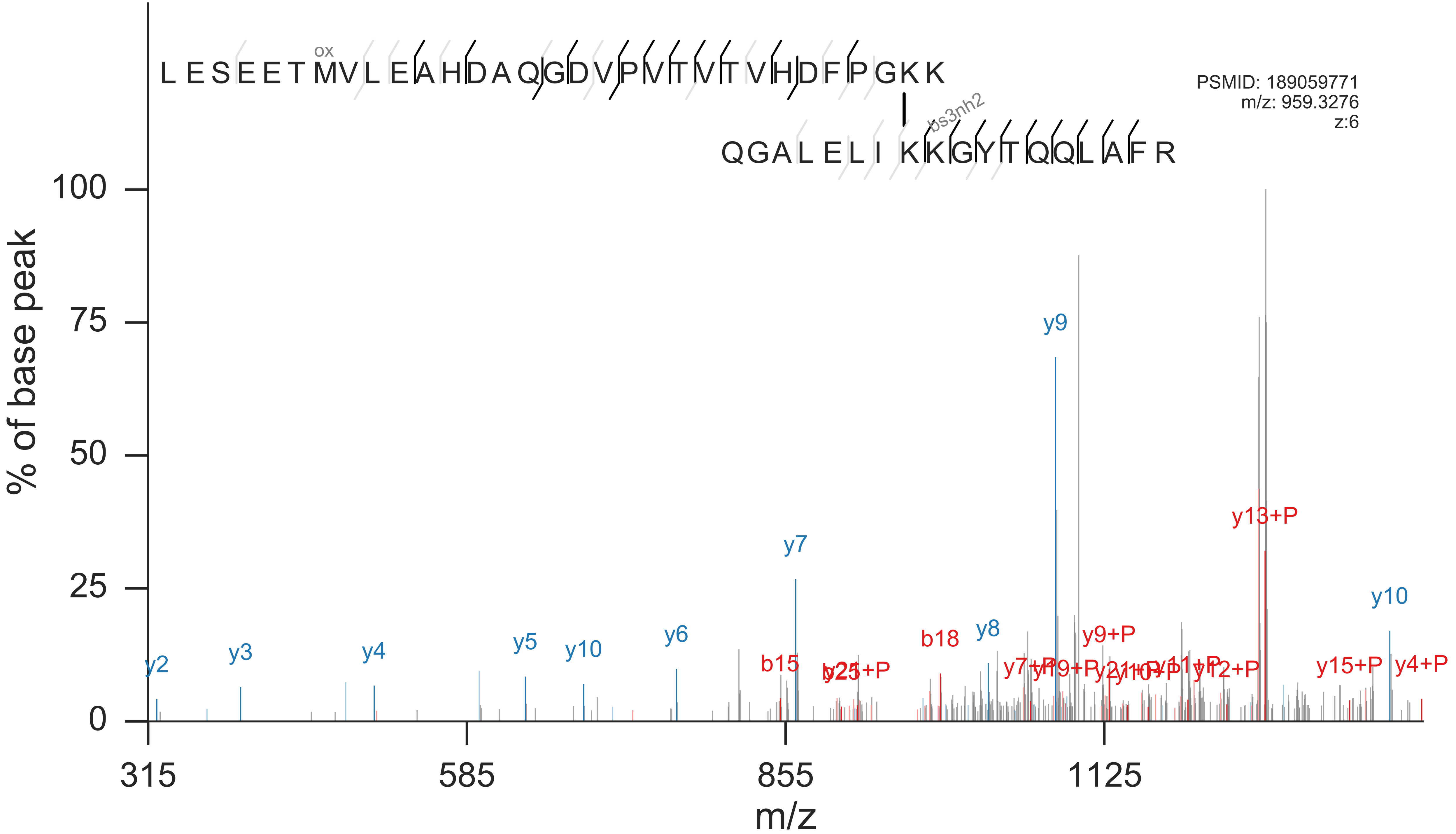

PSMID: 189059771
m/z: 959.3276
z:6
LESEETMVLEAHDAQGDVPVTVTVHDFPGKK
ox
QGALELIKKGYTQQLAFR
bs3nh2
% of base peak
m/z
y2
y3
y4
y5
y10
y6
y7
b15
b18
y8
y9
y9+P
y13+P
y10
y15+P
y4+P
y12+P

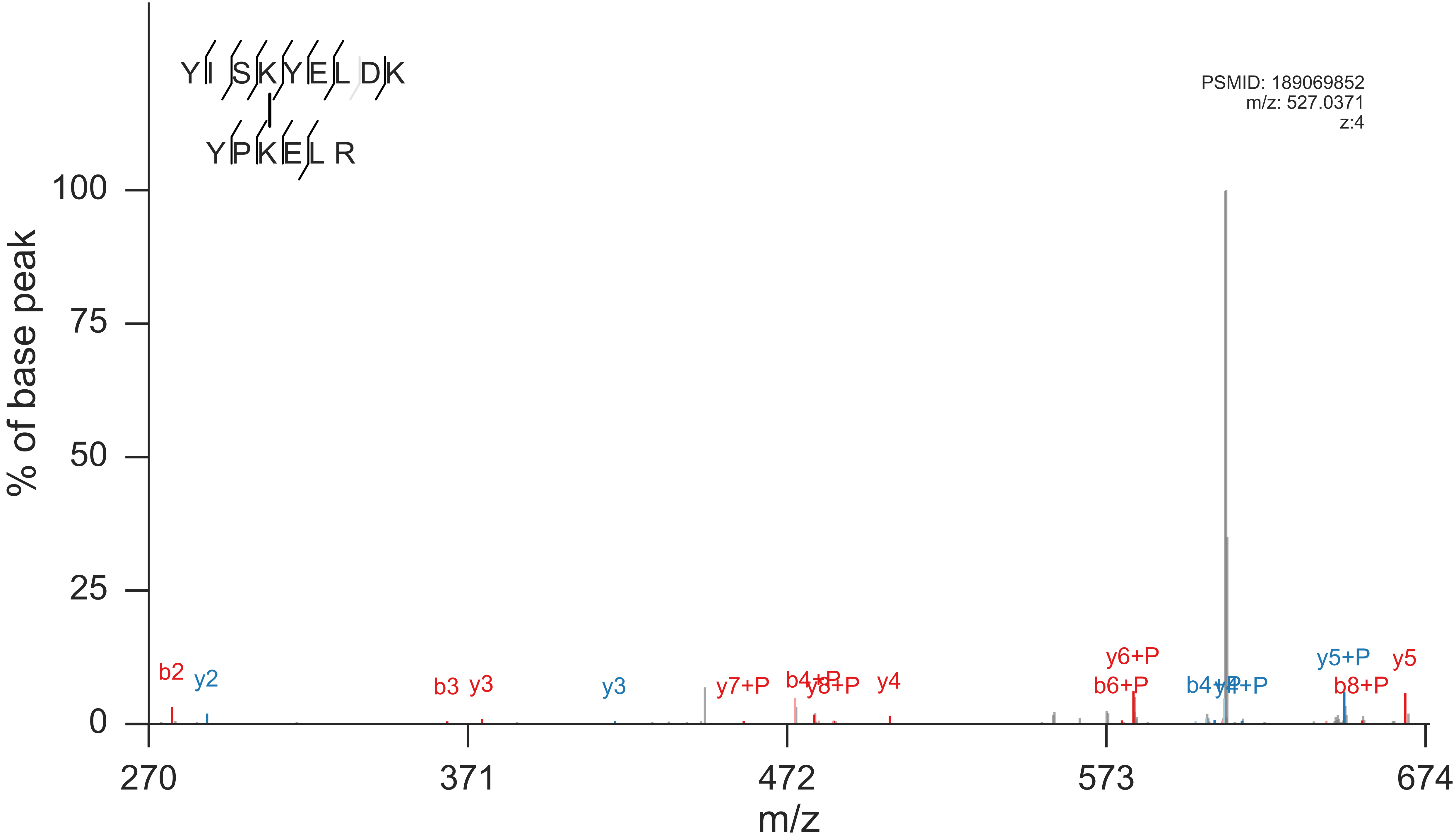

YISKYELDK
YPKELR
PSMID: 189069852
m/z: 527.0371
z:4
% of base peak
100
75
50
25
0
b2
y2
b3
y3
y3
y7+P
b4+P
y8+P
y4
y6+P
b6+P
b4+P
y4+P
y5+P
y5
b8+P
270
371
472
573
674
m/z

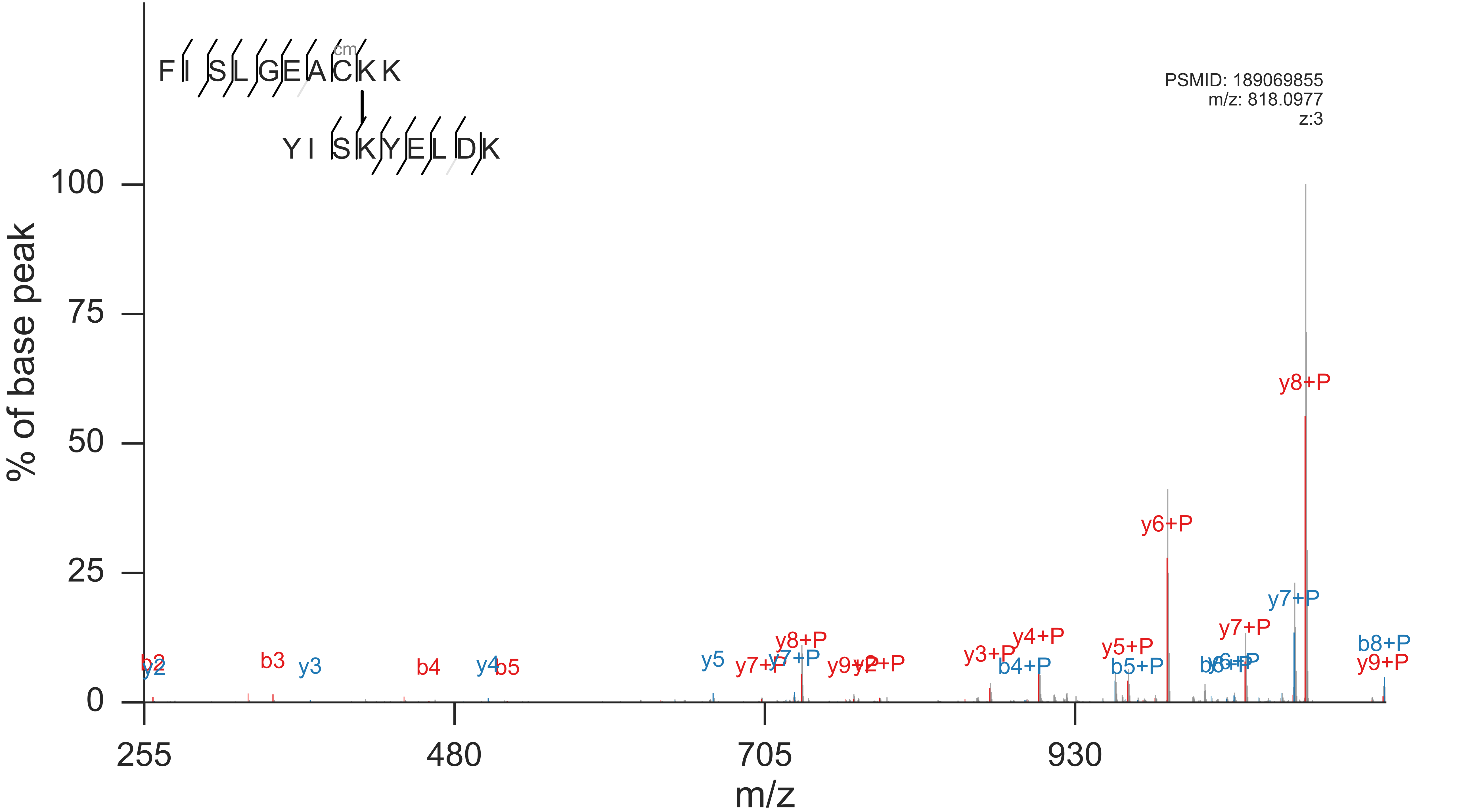

FISLGEACKK
cm
YISKYELDK
PSMID: 189069855
m/z: 818.0977
z:3
% of base peak
m/z
y8+P
y6+P
y7+P
b8+P
y9+P

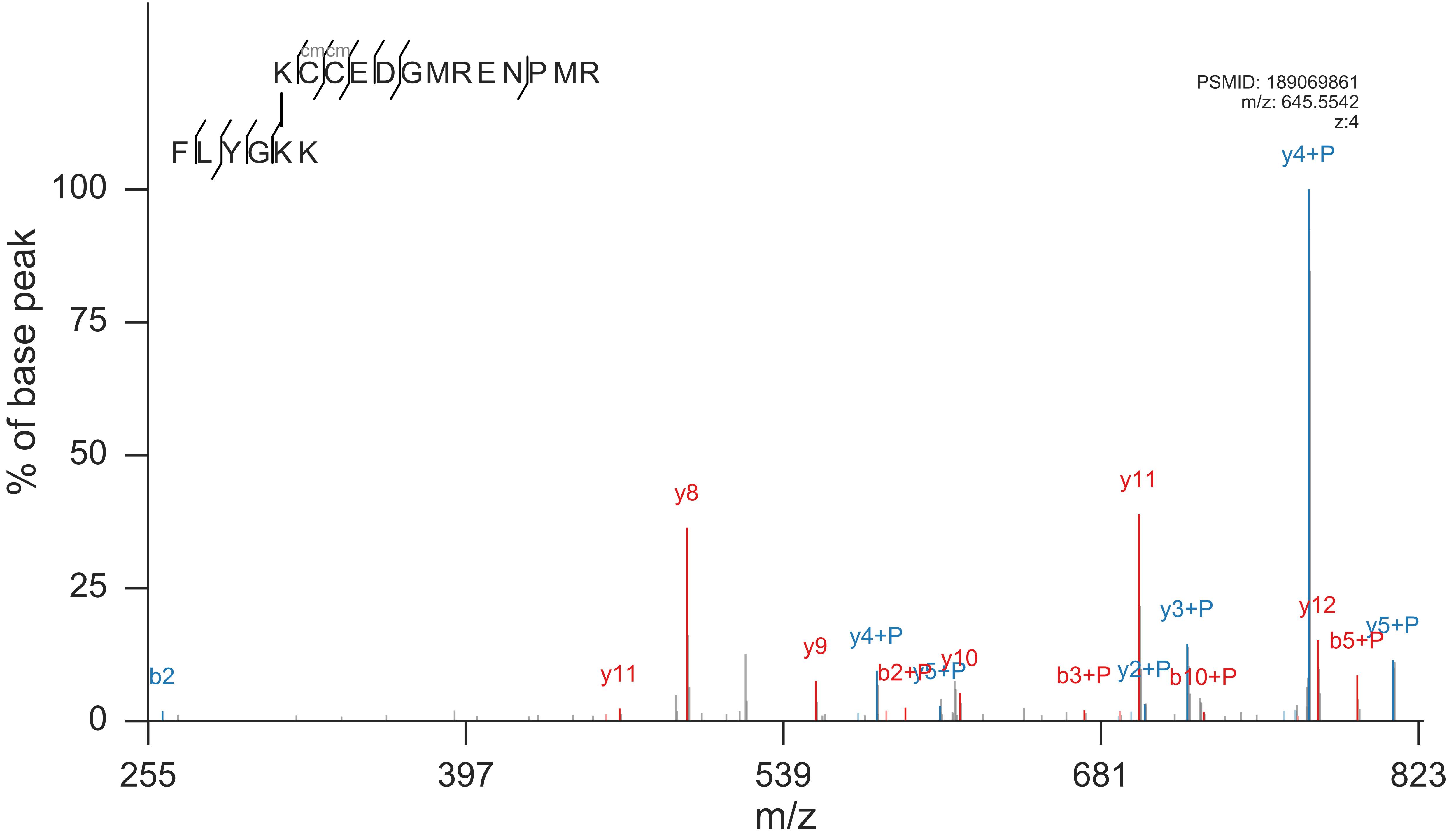

KCCEDGMRENPMR
cm cm
FLYGKK
PSMID: 189069861
m/z: 645.5542
z:4
% of base peak
m/z
y4+P
y8
y11
y3+P
y12
y5+P
b5+P
y9
y4+P
y10
b2+P
y5+P
b3+P
y2+P
b10+P
b2
y11

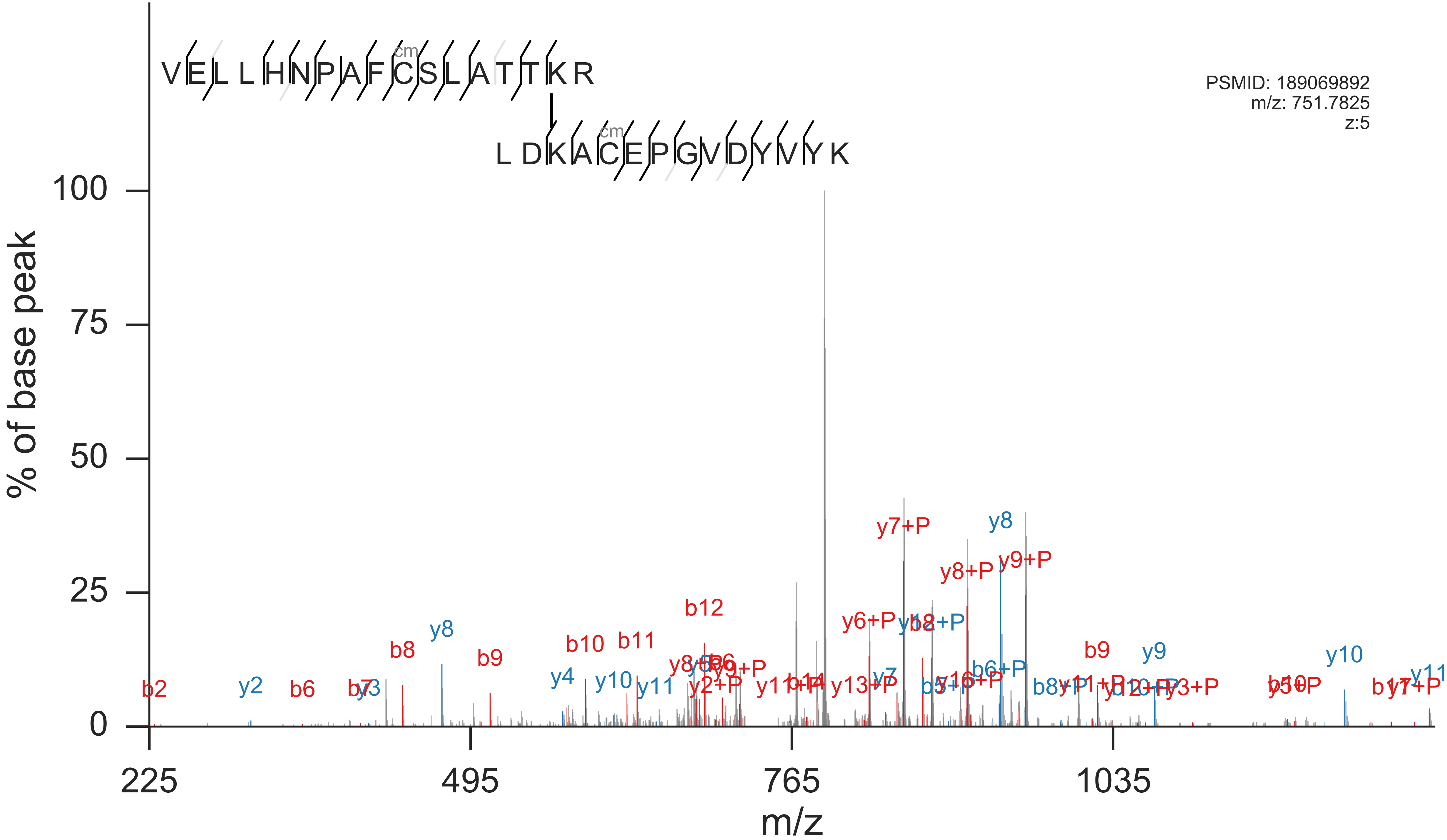

VELLHNPAFCSLATTKR
LDKACEPGVDYVYK
cm
PSMID: 189069892
m/z: 751.7825
z:5
% of base peak
100
75
50
25
0
225
495
765
1035
m/z
b2
y2
b6
b8
y8
b9
y4
b10
y10
b11
y11
b12
y7+P
y8+P
y9+P
y8
y6+P
b9
y9
y10
y11
b8+P
y13+P
b6+P

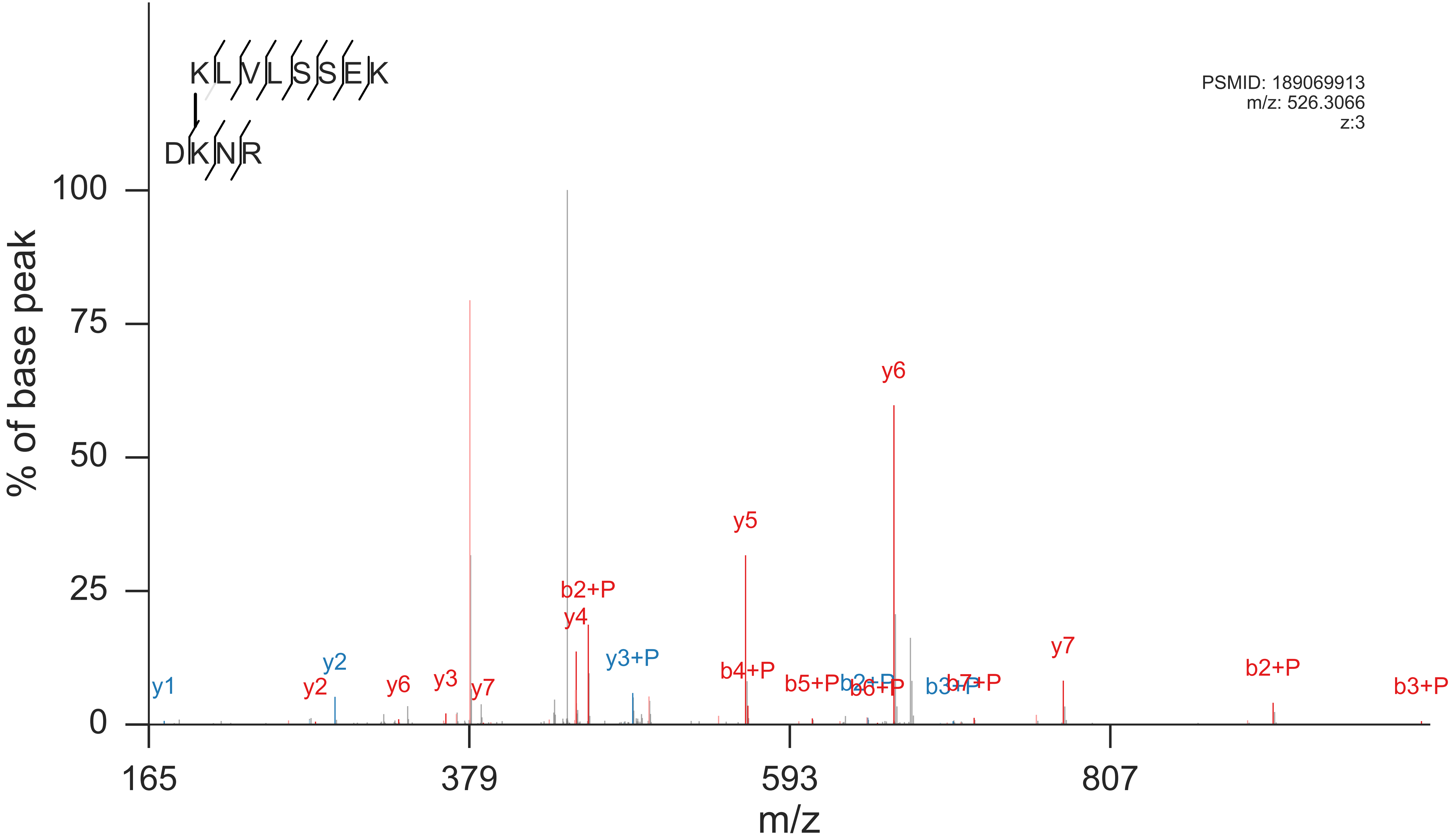
K L V L S S E K
D K N R
PSMID: 189069913
m/z: 526.3066
z:3
% of base peak
100
75
50
25
0
165
379
593
807
m/z
y1
y2
y2
y6
y3
y7
b2+P
y4
y3+P
y5
b4+P
b5+P
y6
y7
b2+P
b3+P

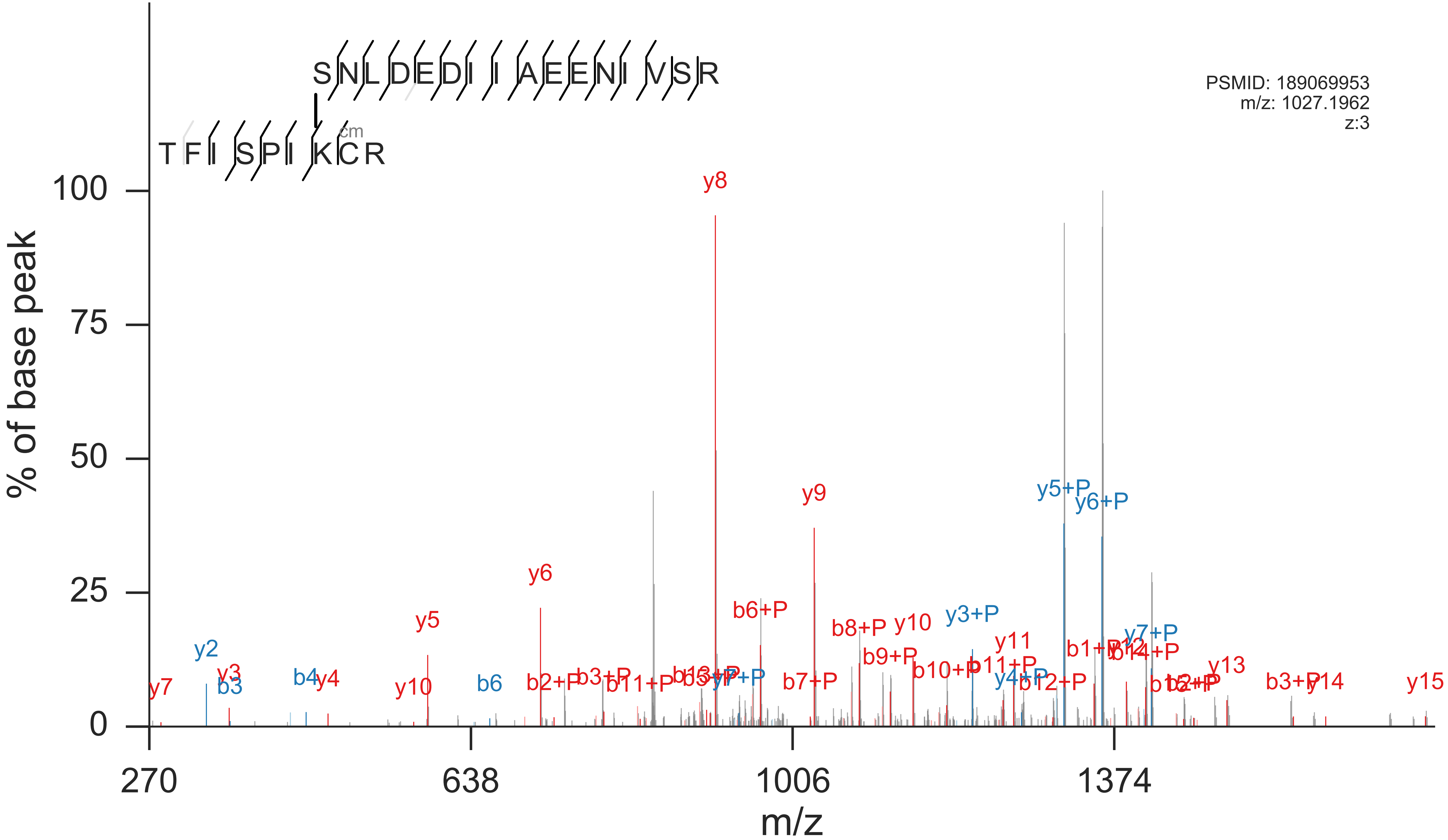
SNLDEDI I AEENI VSR
TFI SPI KCR
cm
PSMID: 189069953
m/z: 1027.1962
z:3
% of base peak
100
75
50
25
0
270
638
1006
1374
m/z
y8
y9
y6
y5
y2
y3
b3
b4
y4
y7
y10
b6
b2+P
b3+P
b11+P
b6+P
b8+P
y10
y3+P
b9+P
y11
b10+P
b11+P
y4+P
b12+P
b7+P
y5+P
y6+P
y7+P
b14+P
y13
b3+P
y14
y15

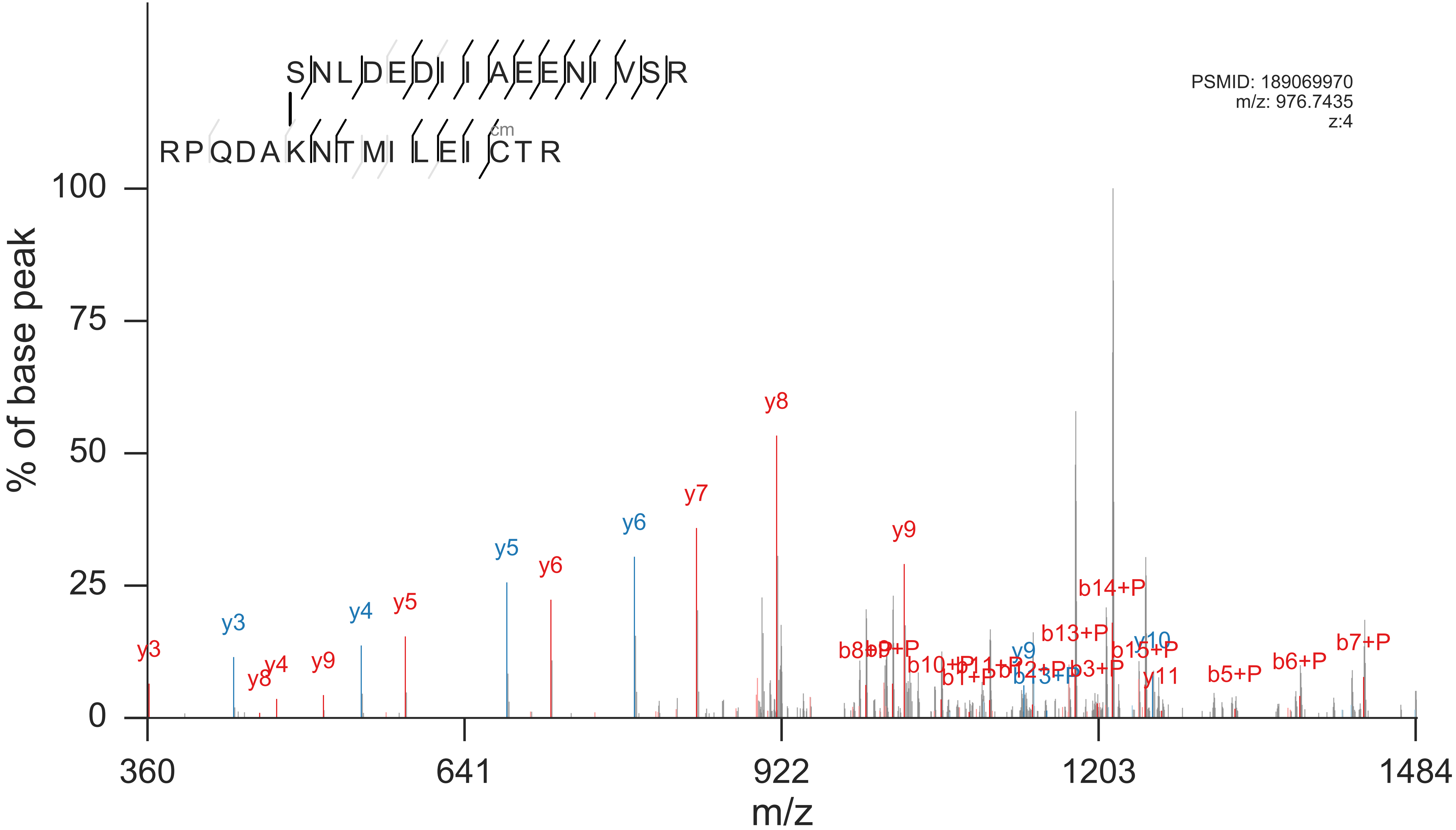

SNLDEDIIAEENIVSR
RPQDAKNTMILEICTR
cm
PSMID: 189069970
m/z: 976.7435
z:4
% of base peak
m/z

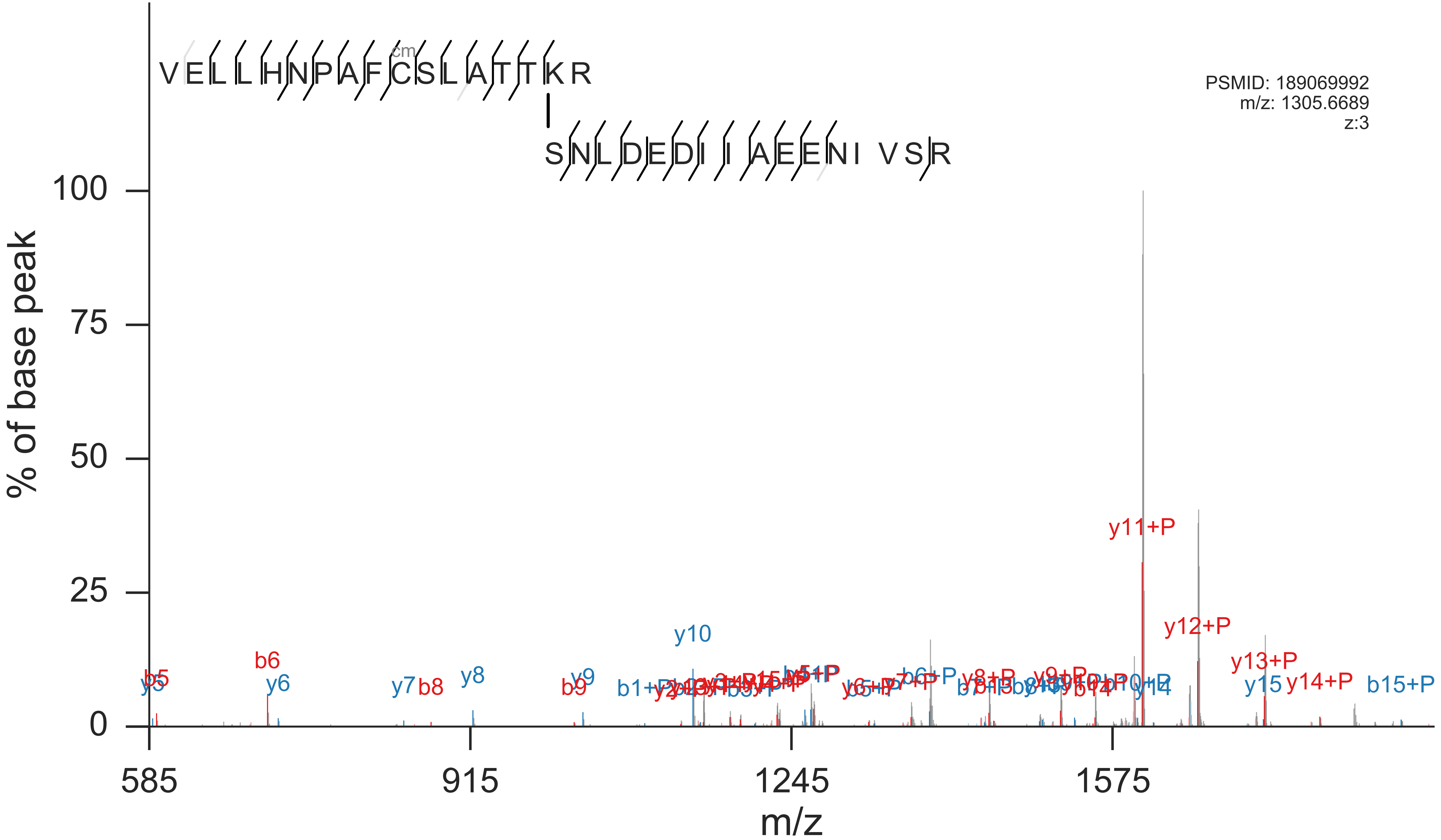

VELLHNPAFCSLATTKR
cm
SNLDEDIIAEENIVSR
PSMID: 189069992
m/z: 1305.6689
z:3
% of base peak
100
75
50
25
0
585
915
1245
1575
m/z
y11+P
y12+P
y13+P
y14+P
y15
b15+P
y10
b6
b5
y5
y6
y7
b8
y8
b9
y9
y14

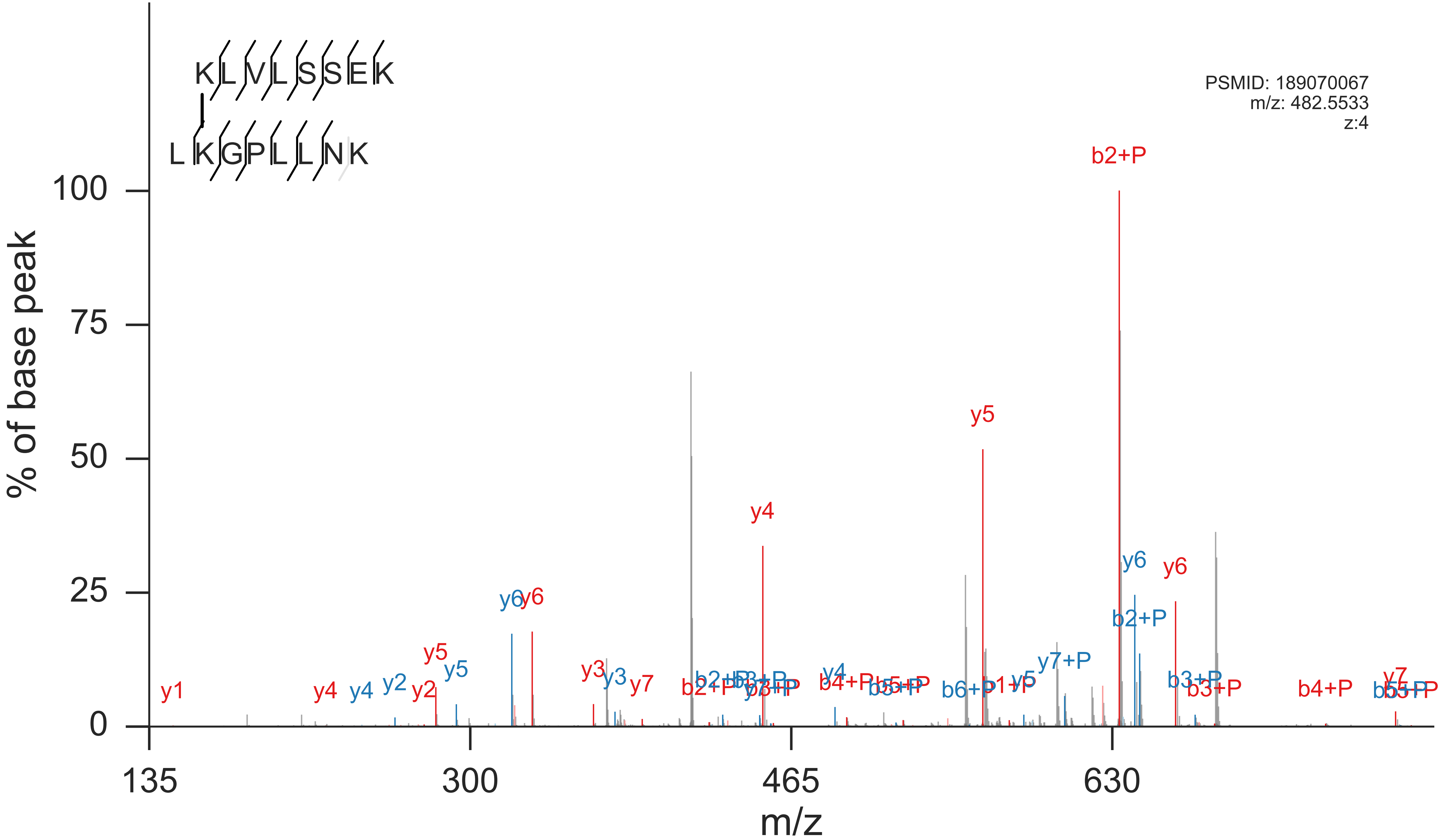
KLVLSSEK
LKGPLLNK
PSMID: 189070067
m/z: 482.5533
z:4
% of base peak
m/z
b2+P
y5
y4
y6
y7+P
b3+P
b4+P
y7

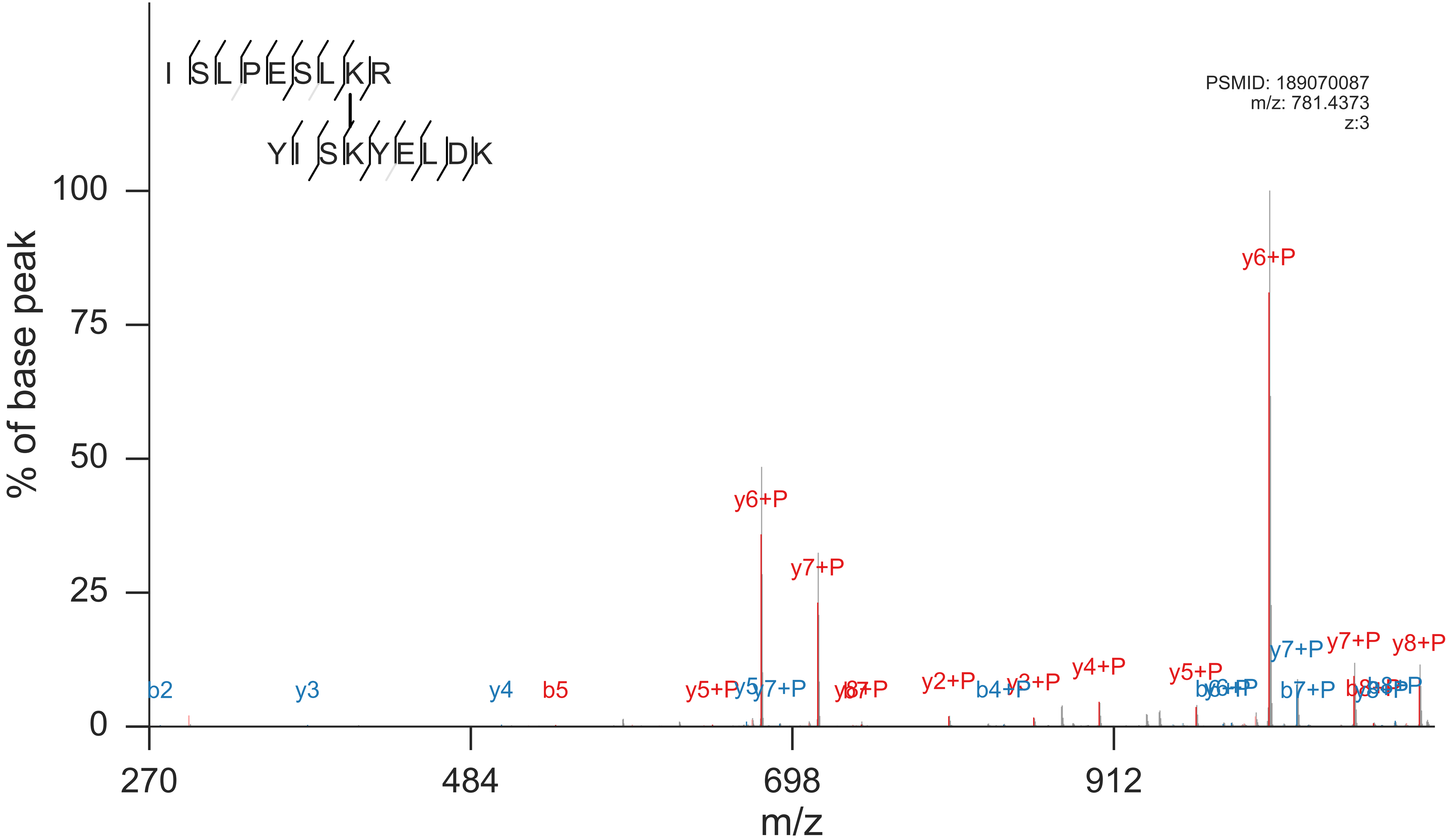
I S L P E S L K R
Y I S K Y E L D K
PSMID: 189070087
m/z: 781.4373
z:3
% of base peak
100
75
50
25
0
270
484
698
912
m/z
y6+P
y6+P
y7+P
y7+P
y7+P
y8+P
y4+P
y5+P
y2+P
y3+P
b4+P
b7+P
y5+P
y5
y7+P
b2
y3
y4
b5

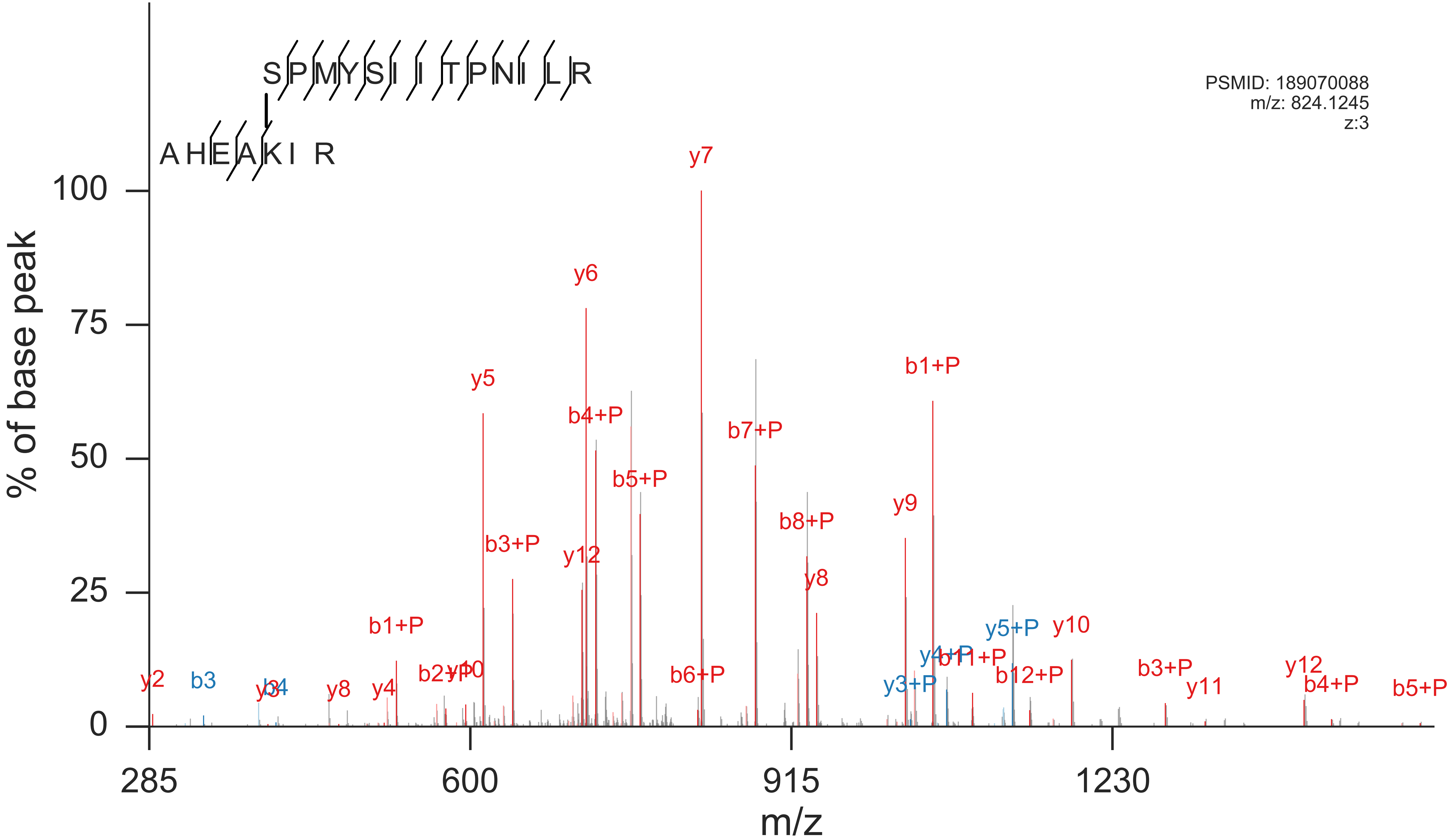

SPMYSIITPNILR
AHEAKIR
PSMID: 189070088
m/z: 824.1245
z:3
% of base peak
m/z
0
25
50
75
100
285
600
915
1230
y7
y6
y5
b4+P
b7+P
b1+P
b5+P
y9
b8+P
b3+P
y12
y8
y5+P
y10
b1+P
y4+P
b11+P
b2+P
y10
b6+P
b12+P
b3+P
y3+P
y12
y2
b3
y3
b4
y8
y4
y11
b4+P
b5+P

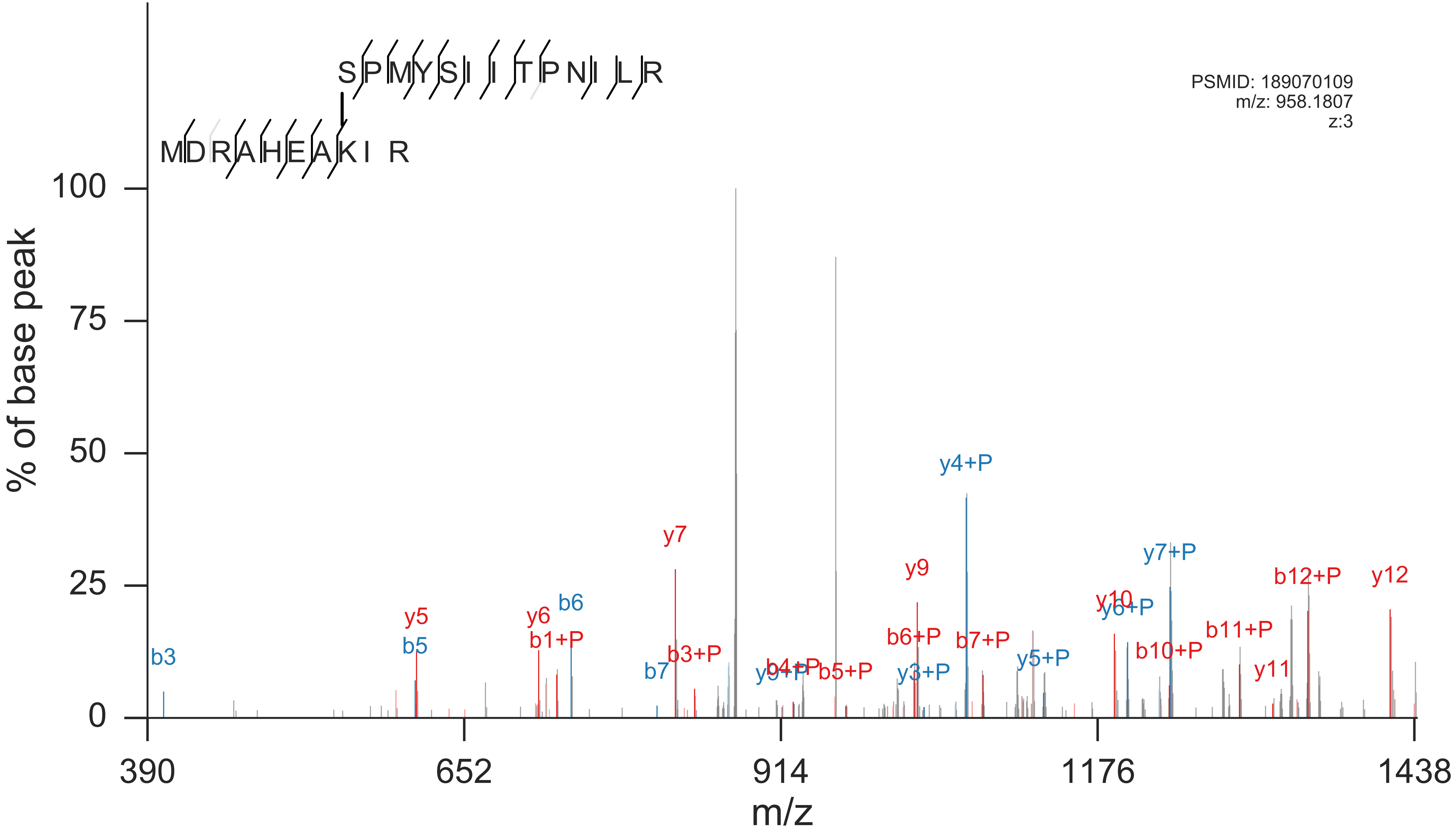

S P M Y S I I T P N I L R
M D R A H E A K I R
PSMID: 189070109
m/z: 958.1807
z:3
% of base peak
100
75
50
25
0
m/z
390
652
914
1176
1438
b3
y5
b5
y6
b1+P
b6
b7
y7
b3+P
b4+P
b5+P
y9
b6+P
y3+P
y4+P
b7+P
y5+P
y10
y6+P
b10+P
y7+P
b11+P
y11
b12+P
y12

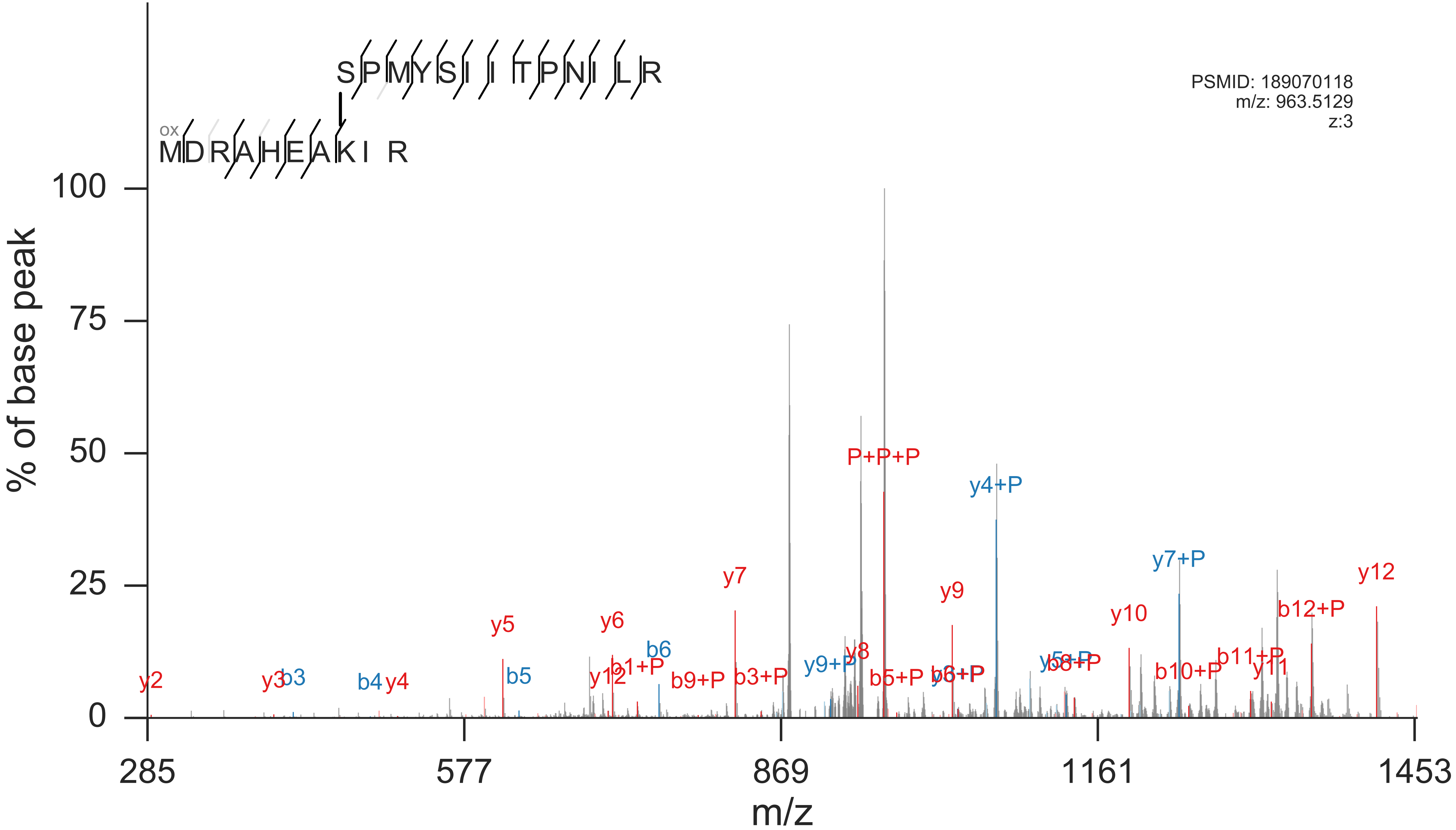
SPMYSIITPNILR
ox
MDRAHEAKIR
PSMID: 189070118
m/z: 963.5129
z:3
% of base peak
m/z
100
75
50
25
0
285
577
869
1161
1453
y2
y3
b3
b4
y4
y5
b5
y6
y12
b1+P
b6
b9+P
y7
b3+P
y9+P
y8
P+P+P
b5+P
y9
b6+P
y4+P
b8+P
y10
b10+P
y7+P
b11+P
y11
b12+P
y12

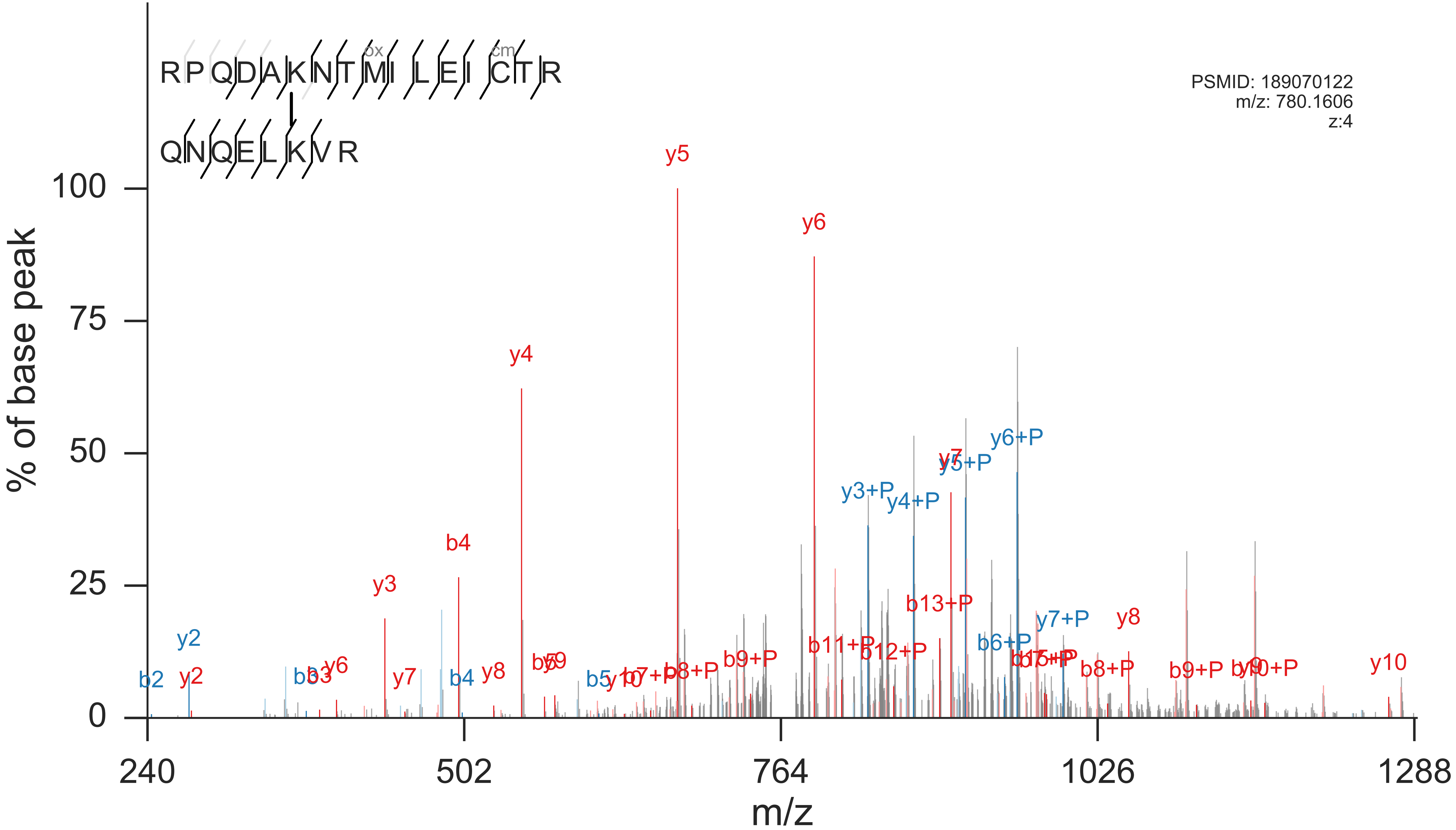
RPQDAKNTMILEICTR
QNQELKVR
ox
cm
PSMID: 189070122
m/z: 780.1606
z:4
% of base peak
m/z
0
25
50
75
100
240
502
764
1026
1288
y5
y6
y4
y7
y6+P
y5+P
y3+P
y4+P
b4
y3
b13+P
y7+P
y8
b6+P
y2
b11+P
b12+P
b9+P
b8+P
b8+P
b9+P
y10
b2
y2
b3
y6
y7
b4
y8
b5
b5
y10

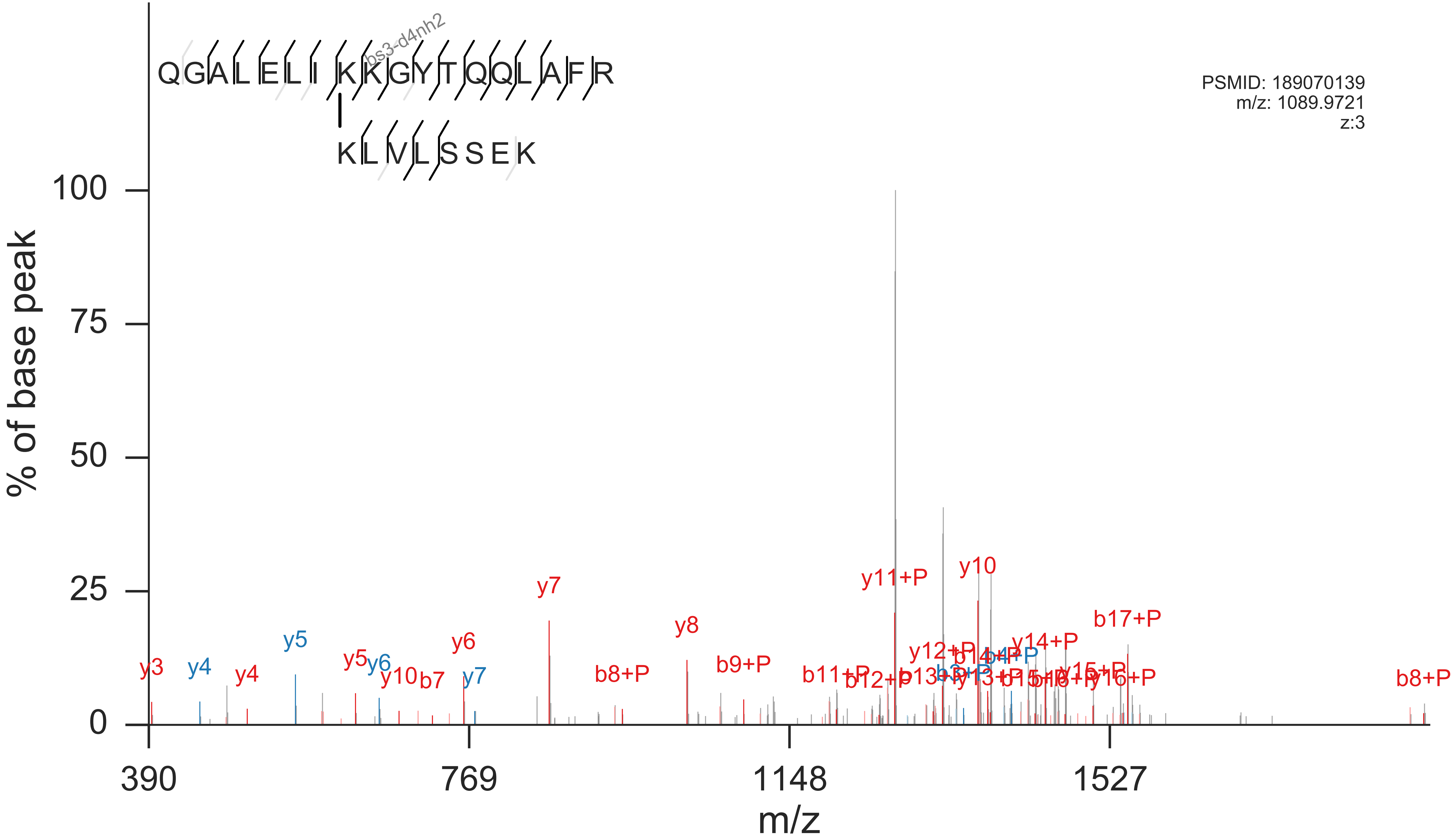
QGALELIKKGYTQQLAFR
KLVLSSEK
bs3-d4nh2
PSMID: 189070139
m/z: 1089.9721
z:3
% of base peak
m/z
y3
y4
y4
y5
y5
y6
y10
b7
y6
y7
y7
b8+P
y8
b9+P
b11+P
b12+P
y11+P
y12+P
y10
y14+P
b17+P
b8+P

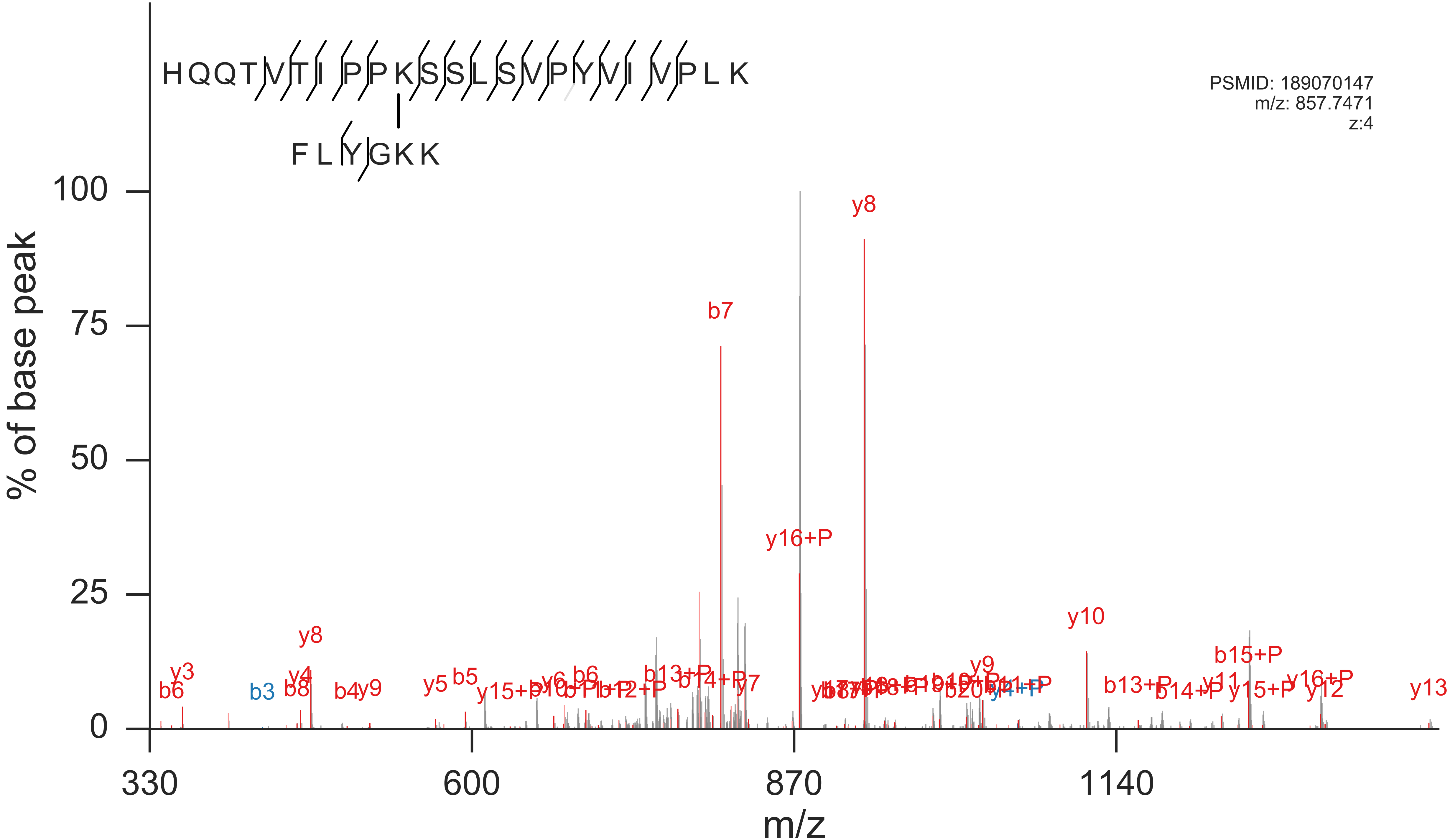

HQQTVTIPPKSSLSVPYVIVPLK
FLYGKK
PSMID: 189070147
m/z: 857.7471
z:4
% of base peak
m/z
100
75
50
25
0
330
600
870
1140
b7
y8
y16+P
y10
b15+P
y13

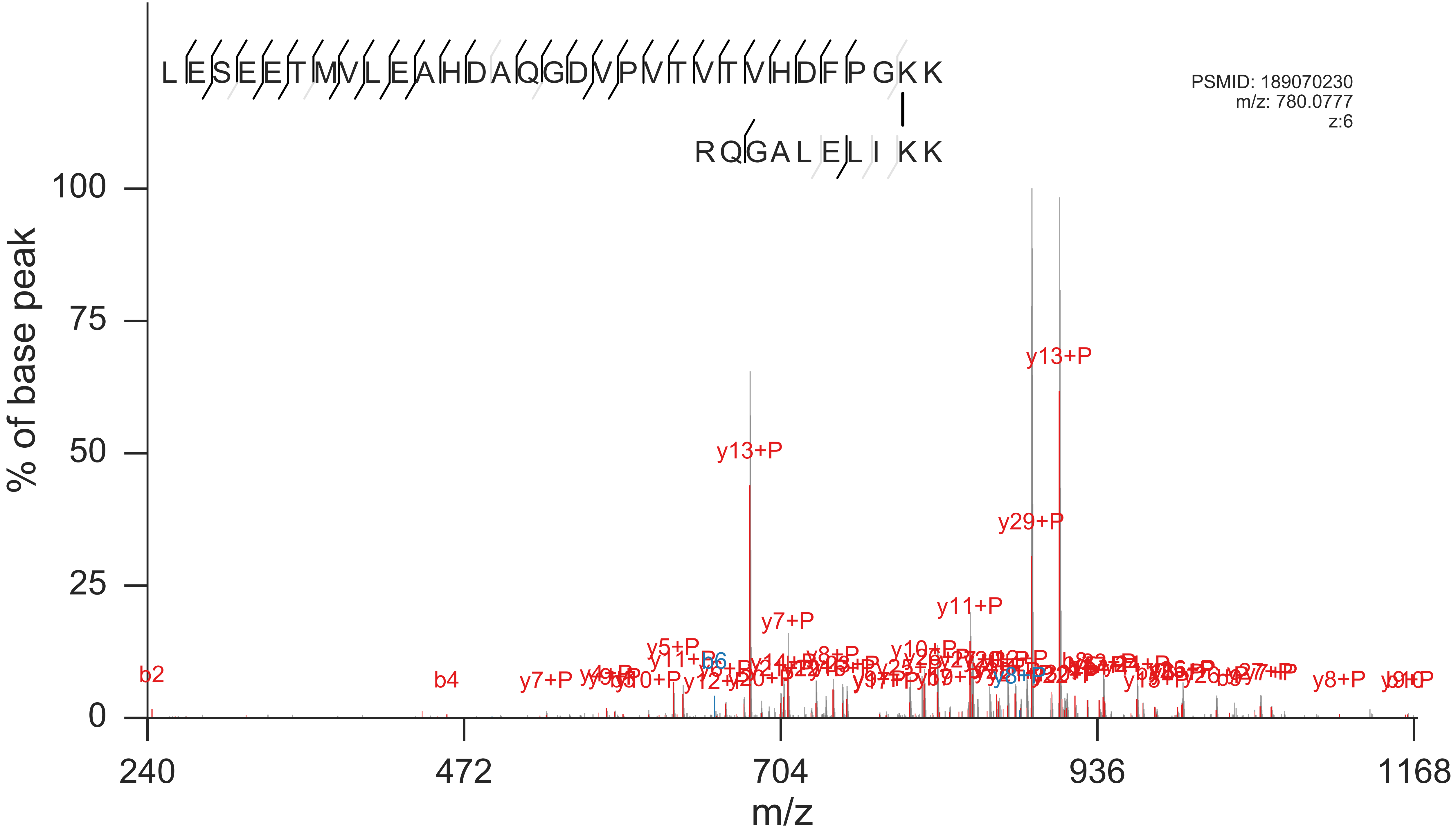
LESEETMVLEAHDAQGDVPVTVTVHDFPGKK
RQGALELIKK
PSMID: 189070230
m/z: 780.0777
z:6
% of base peak
100
75
50
25
0
240
472
704
936
1168
m/z
y13+P
y13+P
y29+P
y11+P
y7+P
y5+P
b2
b4
y7+P
y8+P

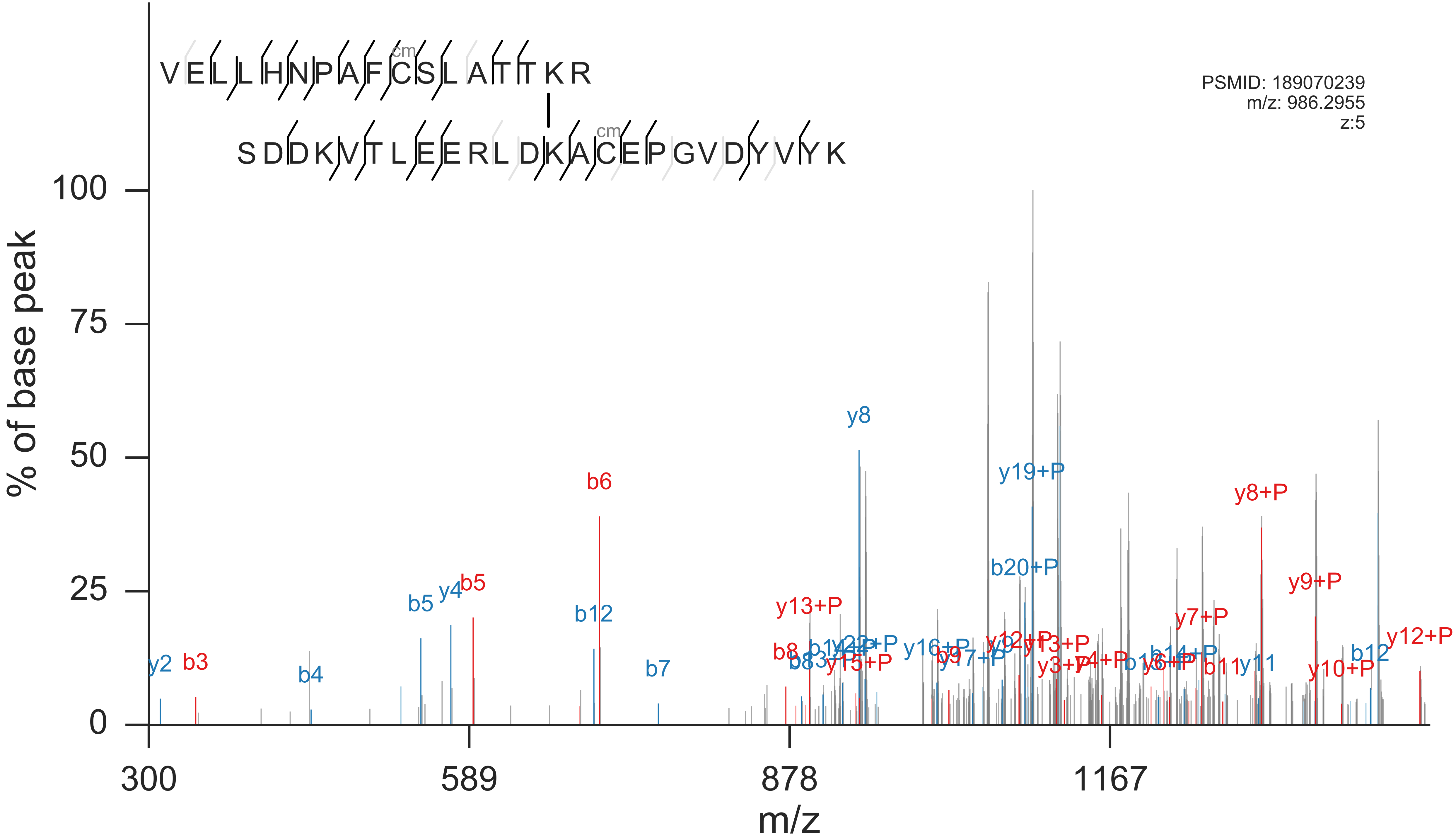
VELLHNPAFCSLATTKR
SDDKVTLEERLDKACEPGVDYVYK
cm
cm
PSMID: 189070239
m/z: 986.2955
z:5
% of base peak
0
25
50
75
100
m/z
300
589
878
1167
y2
b3
b4
b5
y4
b5
b6
b12
b7
y13+P
y8
y19+P
b20+P
y8+P
y9+P
y7+P
y12+P
b12
y10+P
y11
b11
y15+P
b8
y16+P

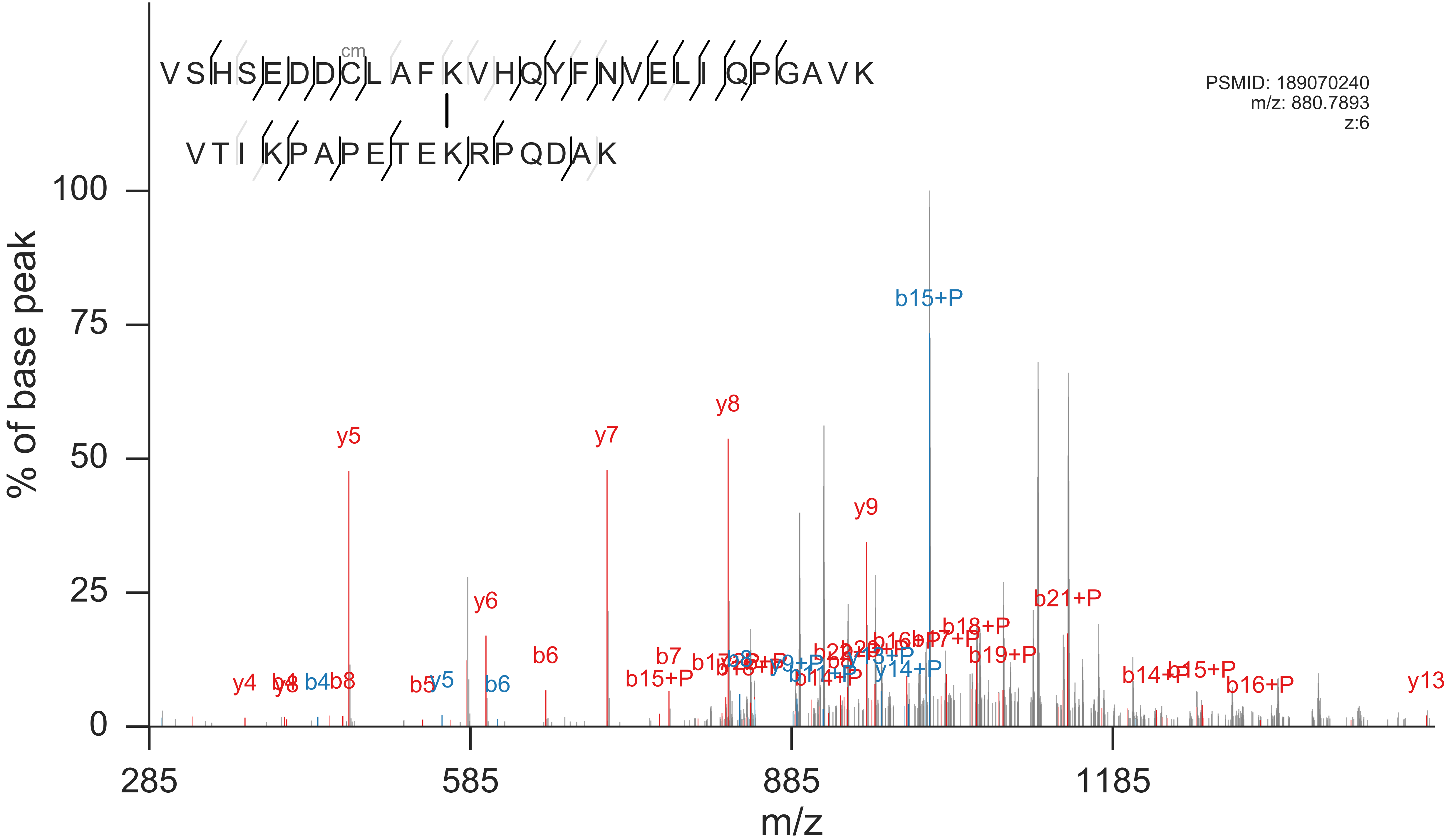
VSHSEDDCLAFKVHQYFNVELIQPGAVK
cm
VTIKPAPETEKRPQDAK
PSMID: 189070240
m/z: 880.7893
z:6
% of base peak
100
75
50
25
0
285
585
885
1185
m/z
b15+P
y8
y5
y7
y9
y6
b21+P
b18+P
b19+P
b6
b7
b15+P
y4
b4
b8
b5
y5
b6
y14+P
b14+P
b15+P
b16+P
y13

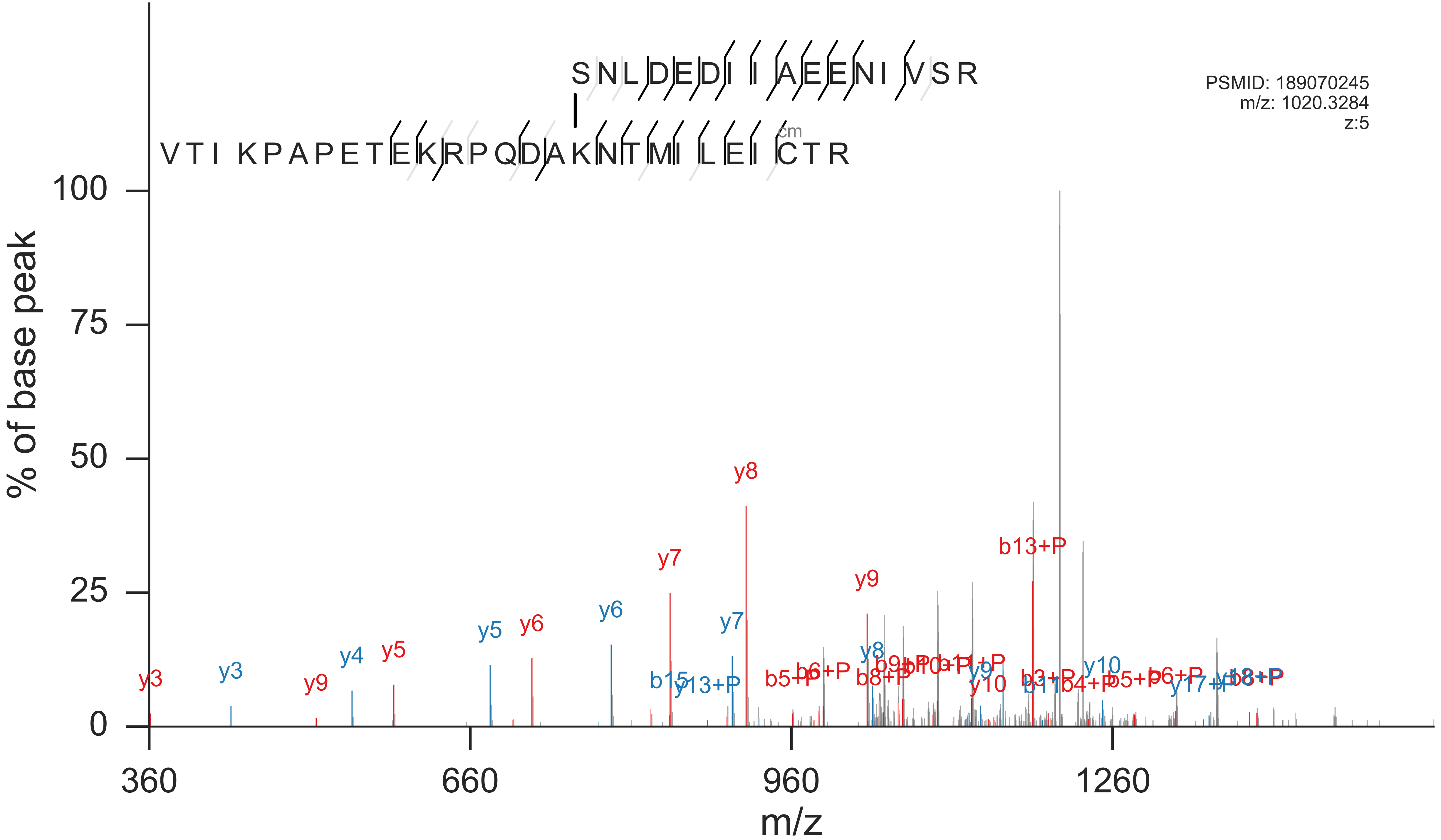

SNLDEDIIAEENIVSR
VTIKPAPETEKRPQDAKNTMILEICTR
cm
PSMID: 189070245
m/z: 1020.3284
z:5
% of base peak
100
75
50
25
0
360
660
960
1260
m/z
y8
y7
y9
b13+P
y3
y3
y9
y4
y5
y5
y6
y6
y7
b15
y13+P
b5+P
b6+P
y8
b8+P
y9
y10
y10
b11
y17+P

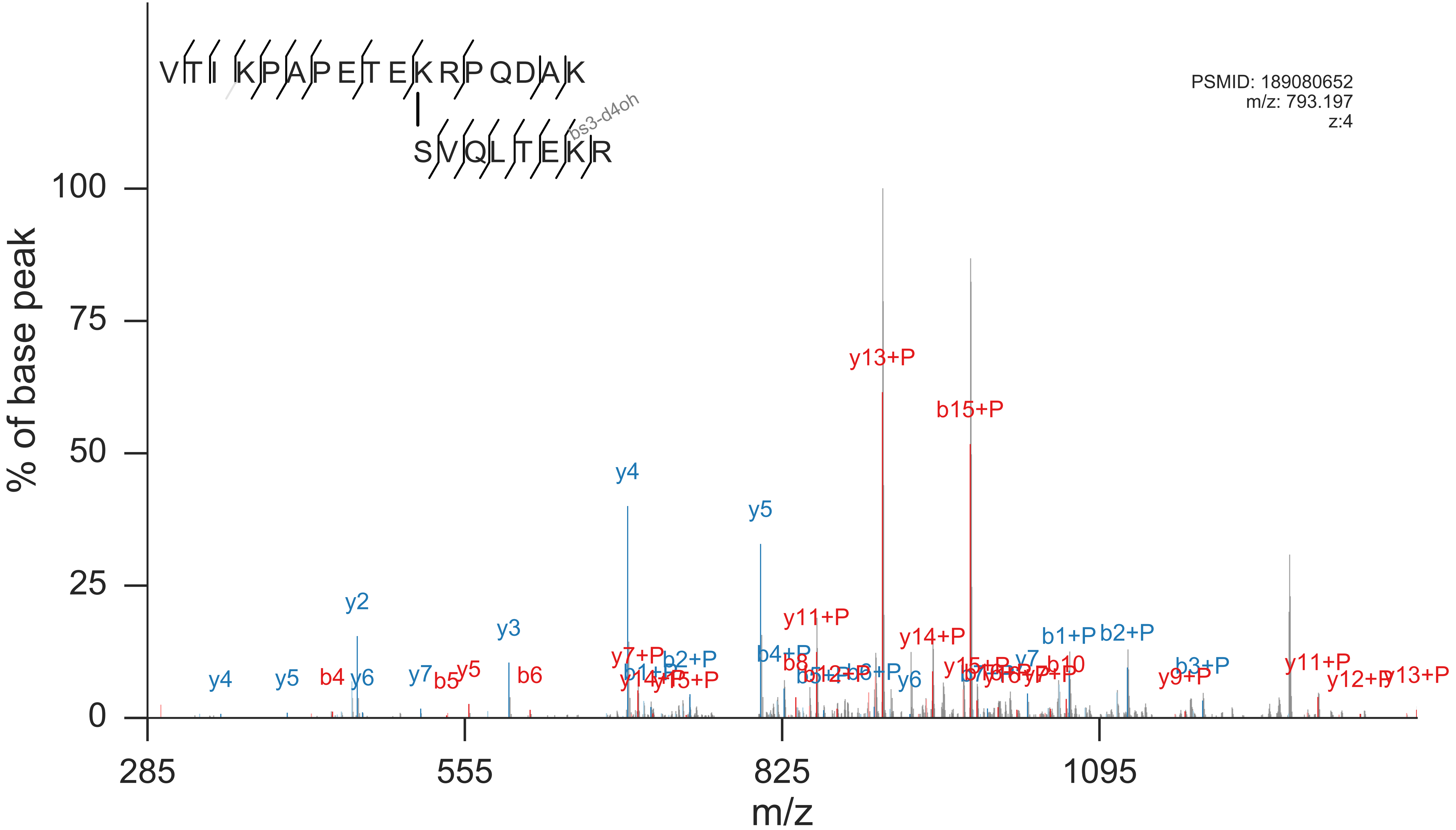

VTIKPAPETEKRPQDAK
SVQLTEKR
bs3-d4oh
PSMID: 189080652
m/z: 793.197
z:4
% of base peak
m/z
y13+P
b15+P
y4
y5
y2
y3
y11+P
y14+P
b1+P
b2+P
b3+P
y9+P
y11+P
y12+P
y13+P
y4
y5
b4
y6
y7
b5
y5
b6
y7+P
b2+P
y14+P
y15+P
b4+P
b8
y6
y7
b10

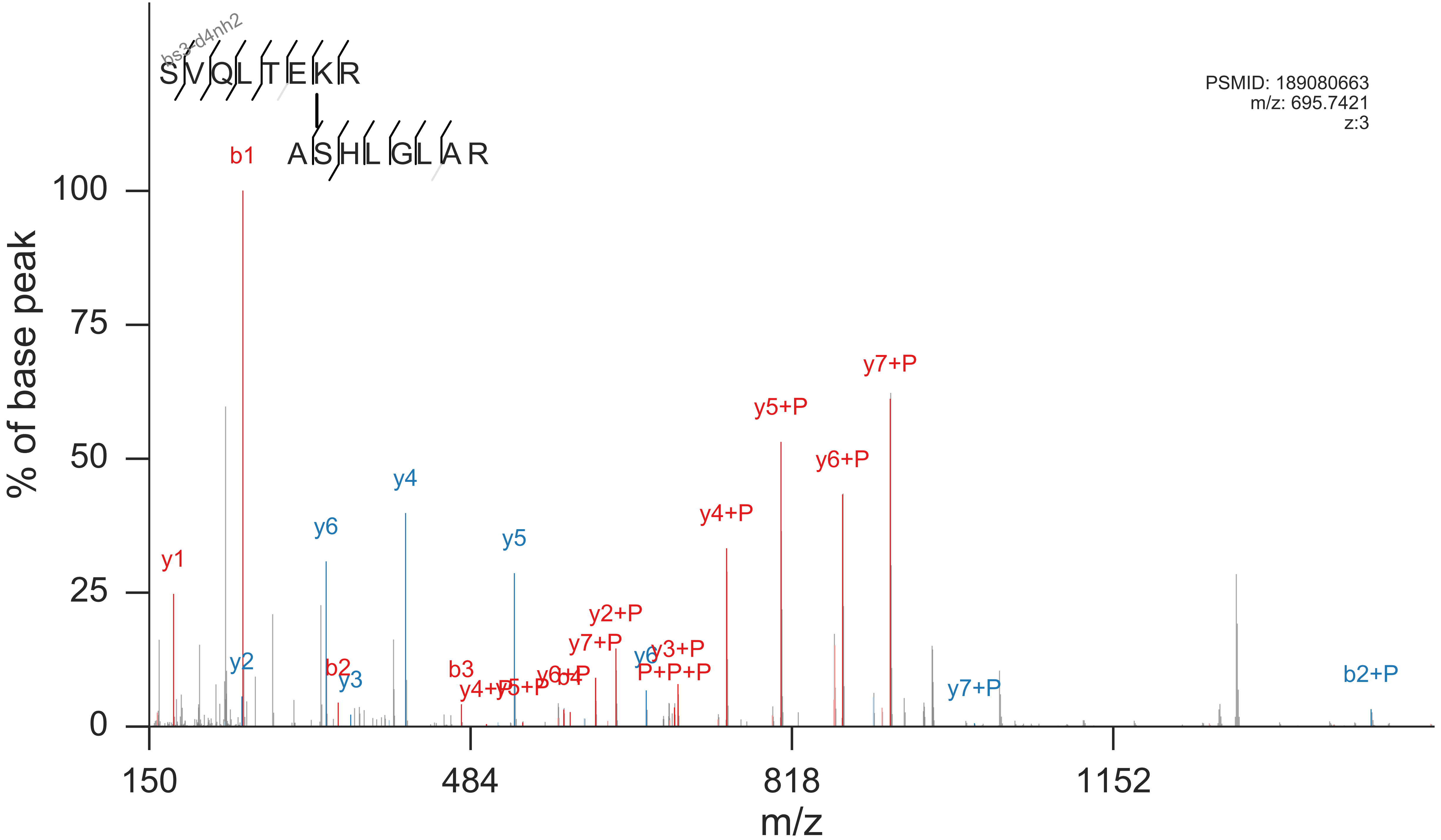

bs3-d4nh2
SVQLTEKR
ASHLGLAR
PSMID: 189080663
m/z: 695.7421
z:3
% of base peak
100
75
50
25
0
150
484
818
1152
m/z
b1
y1
y2
y6
y4
y5
b2
y3
b3
y2+P
y7+P
y4+P
y5+P
y6+P
y7+P
y3+P
P+P+P
b2+P

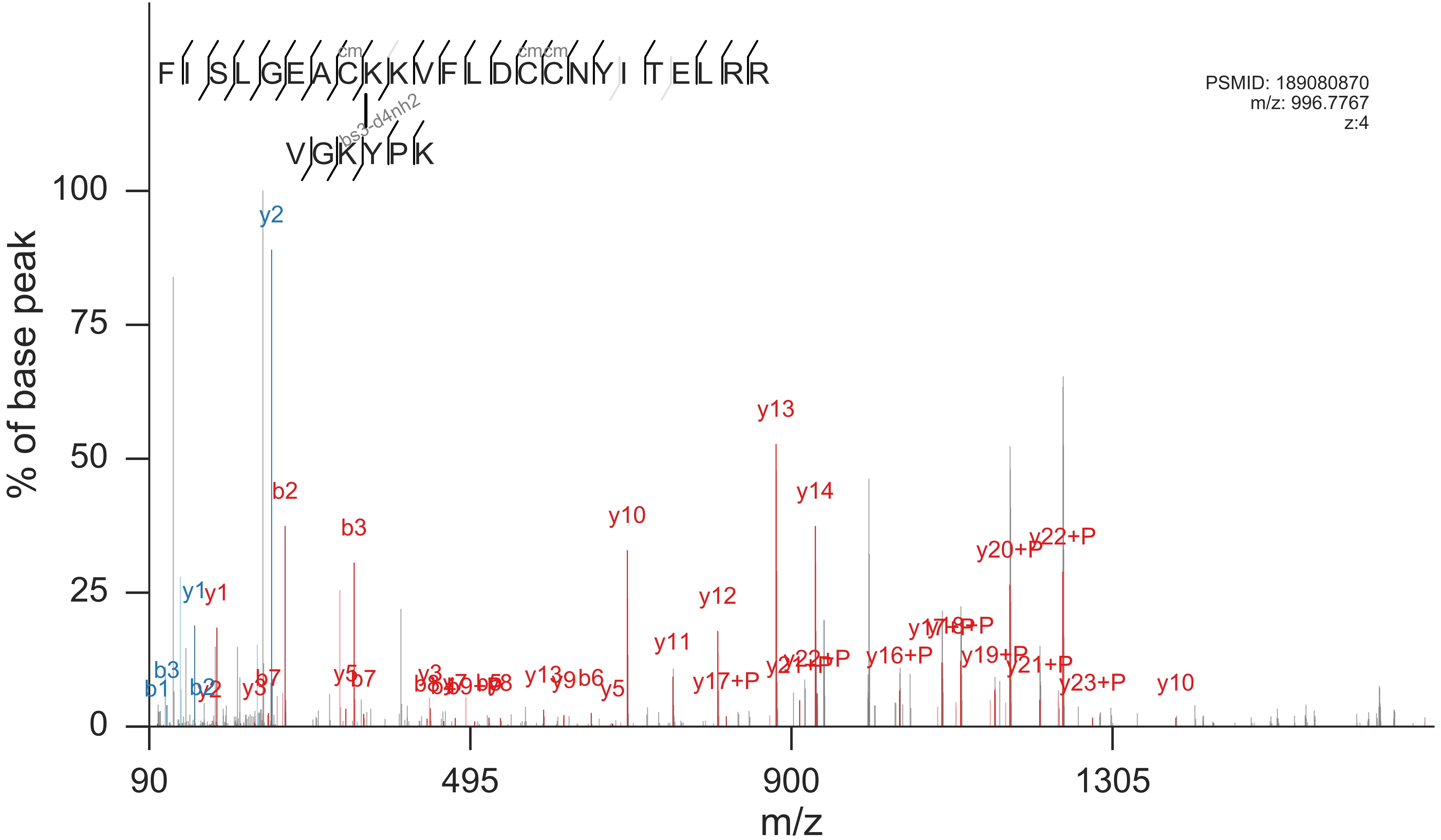

FISLGEACKKVFLDCCNYITELRR
cm
cmcm
bs3-d4nh2
VGKYPK
PSMID: 189080870
m/z: 996.7767
z:4
% of base peak
m/z
0
25
50
75
100
90
495
900
1305

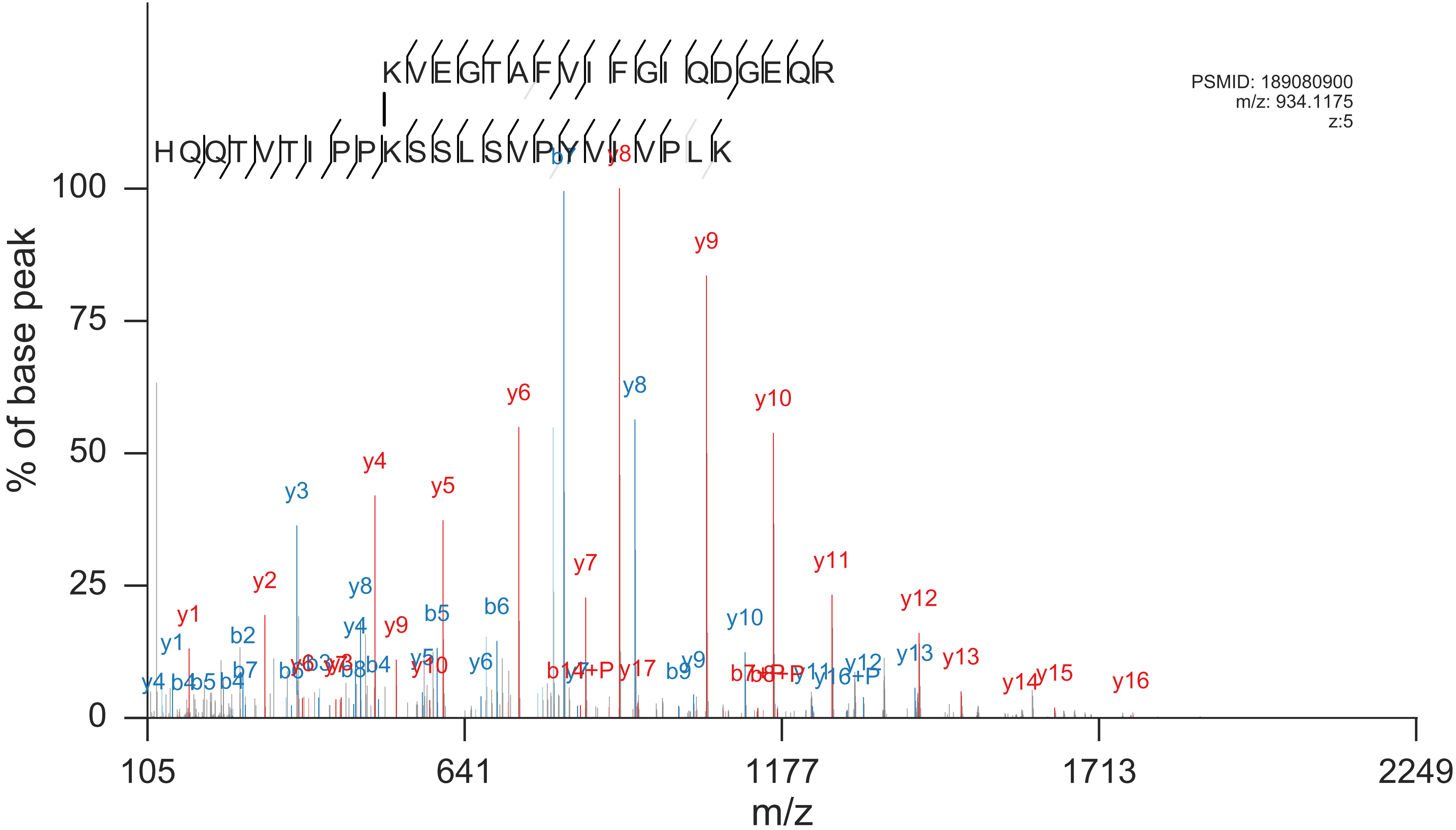

PSMID: 189080900
m/z: 934.1175
z:5
KVEGTAFVIFGIQDGEQR
HQQTVTIPPKSSLSVPYVIVPLK
% of base peak
m/z
0
25
50
75
100
105
641
1177
1713
2249

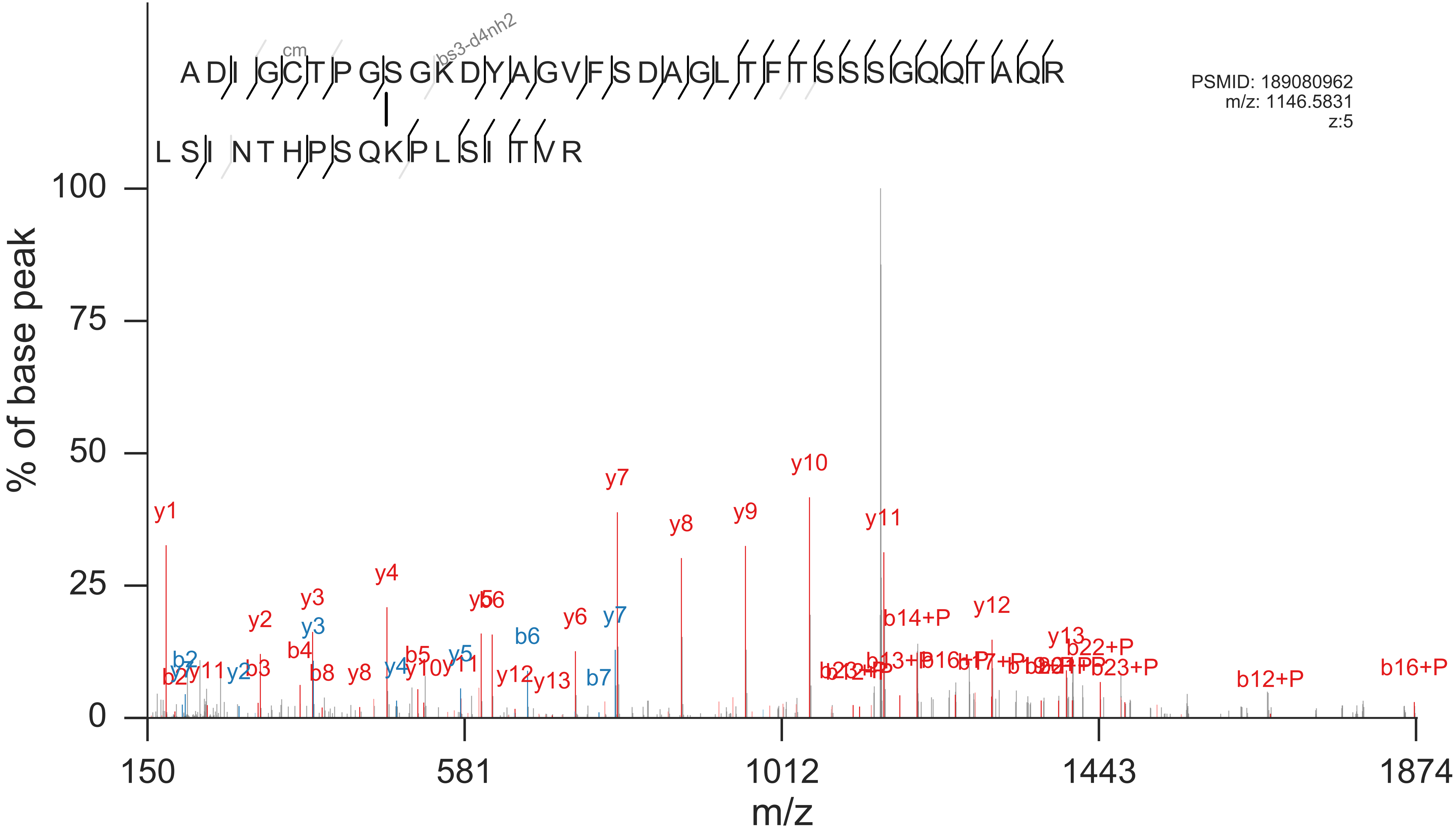
PSMID: 189080962
m/z: 1146.5831
z:5
A D I G C T P G S G K D Y A G V F S D A G L T F T S S S G Q Q T A Q R
cm
bs3-d4nh2
L S I N T H P S Q K P L S I T V R
% of base peak
m/z
100
75
50
25
0
150
581
1012
1443
1874

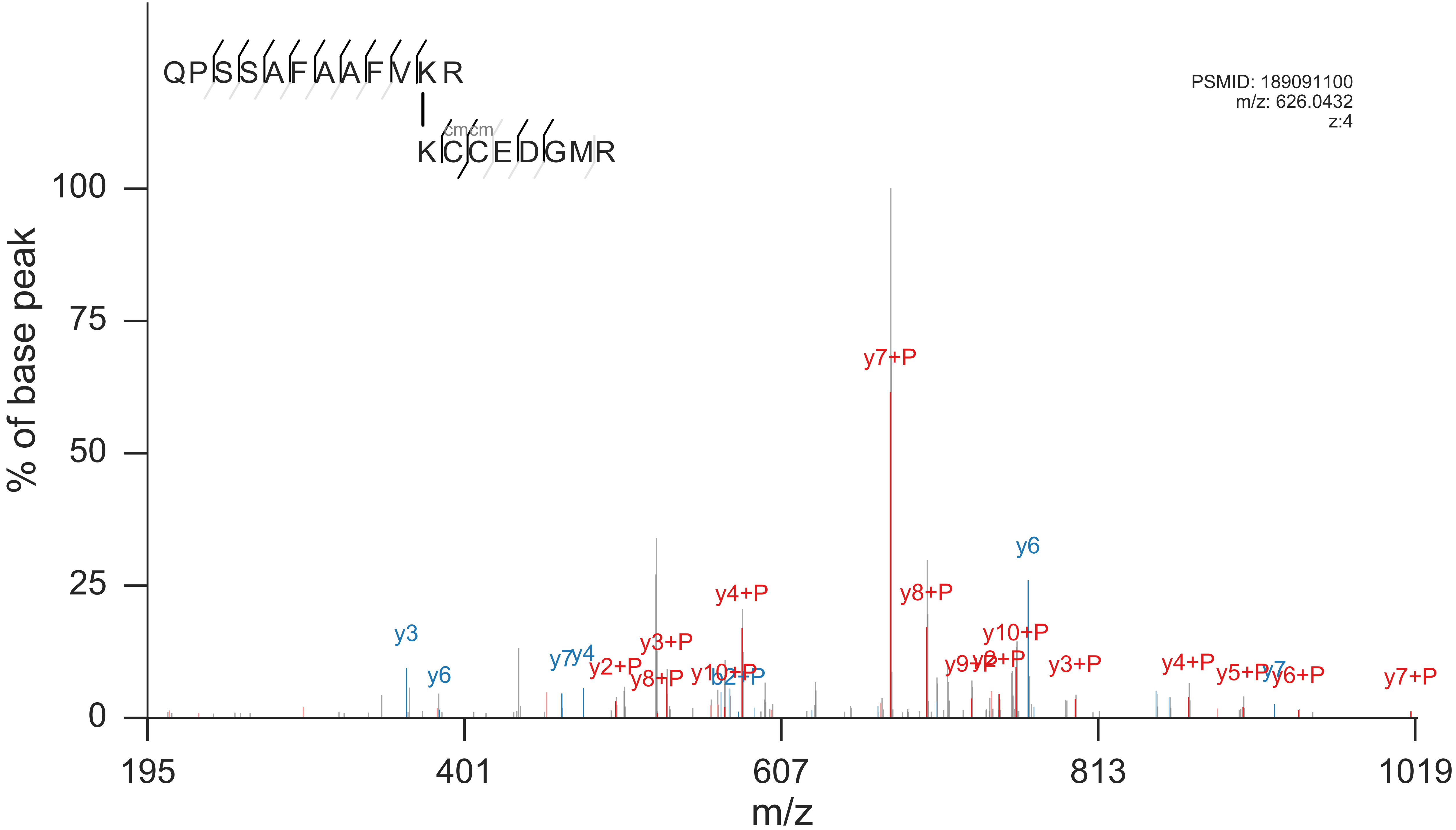

QPSSAFAAFVKR
KCCEDGMR
cm cm
PSMID: 189091100
m/z: 626.0432
z:4
% of base peak
100
75
50
25
0
195
401
607
813
1019
m/z
y7+P
y6
y8+P
y4+P
y10+P
y3+P
y3
y6
y7
y4
y2+P
y8+P
y10+P
b2+P
y9+P
y2+P
y3+P
y4+P
y5+P
y7
y6+P
y7+P

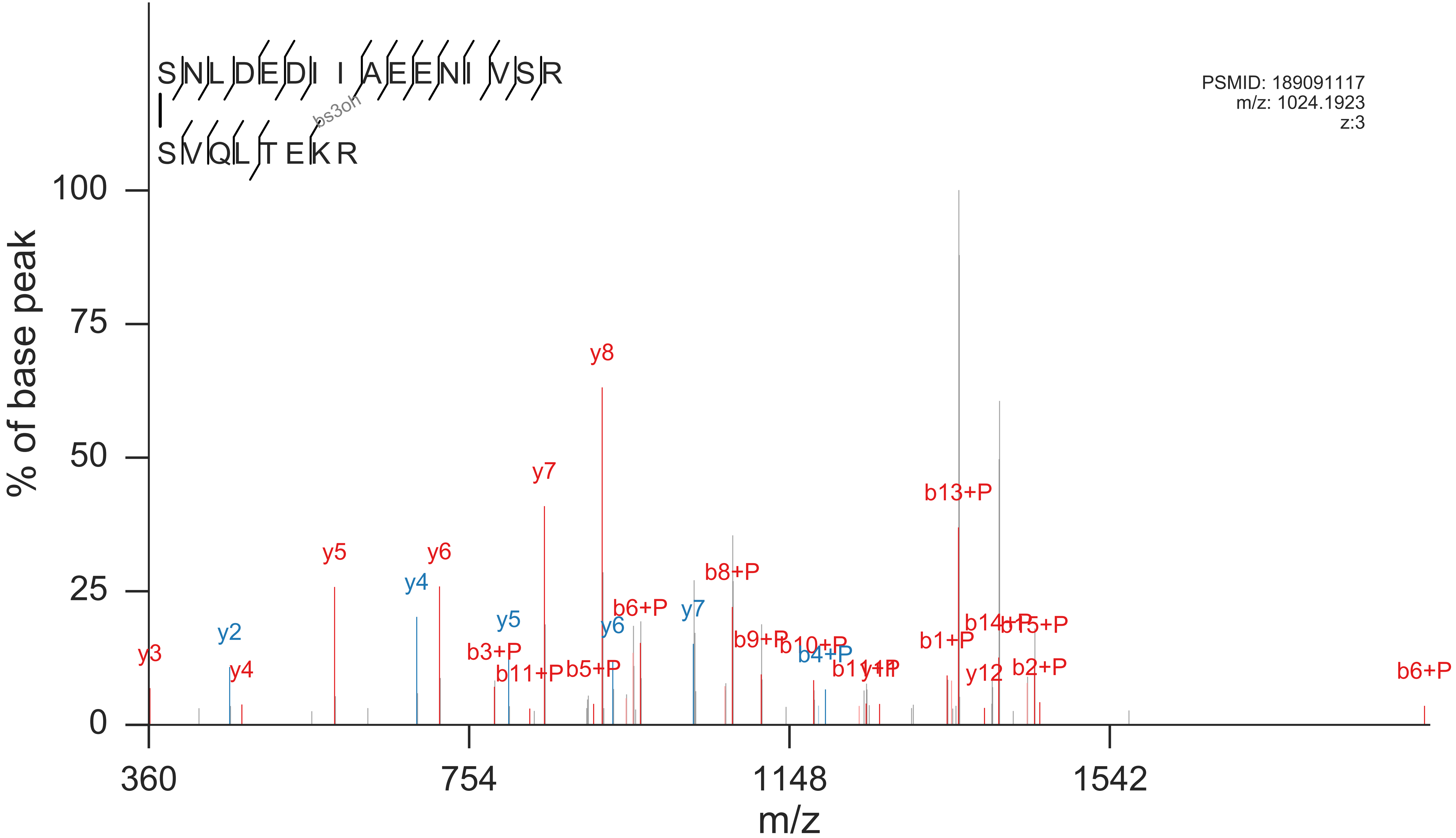

SNLDEDIIAEENIVSR
bs3oh
SVQLTEKR
PSMID: 189091117
m/z: 1024.1923
z:3
% of base peak
m/z

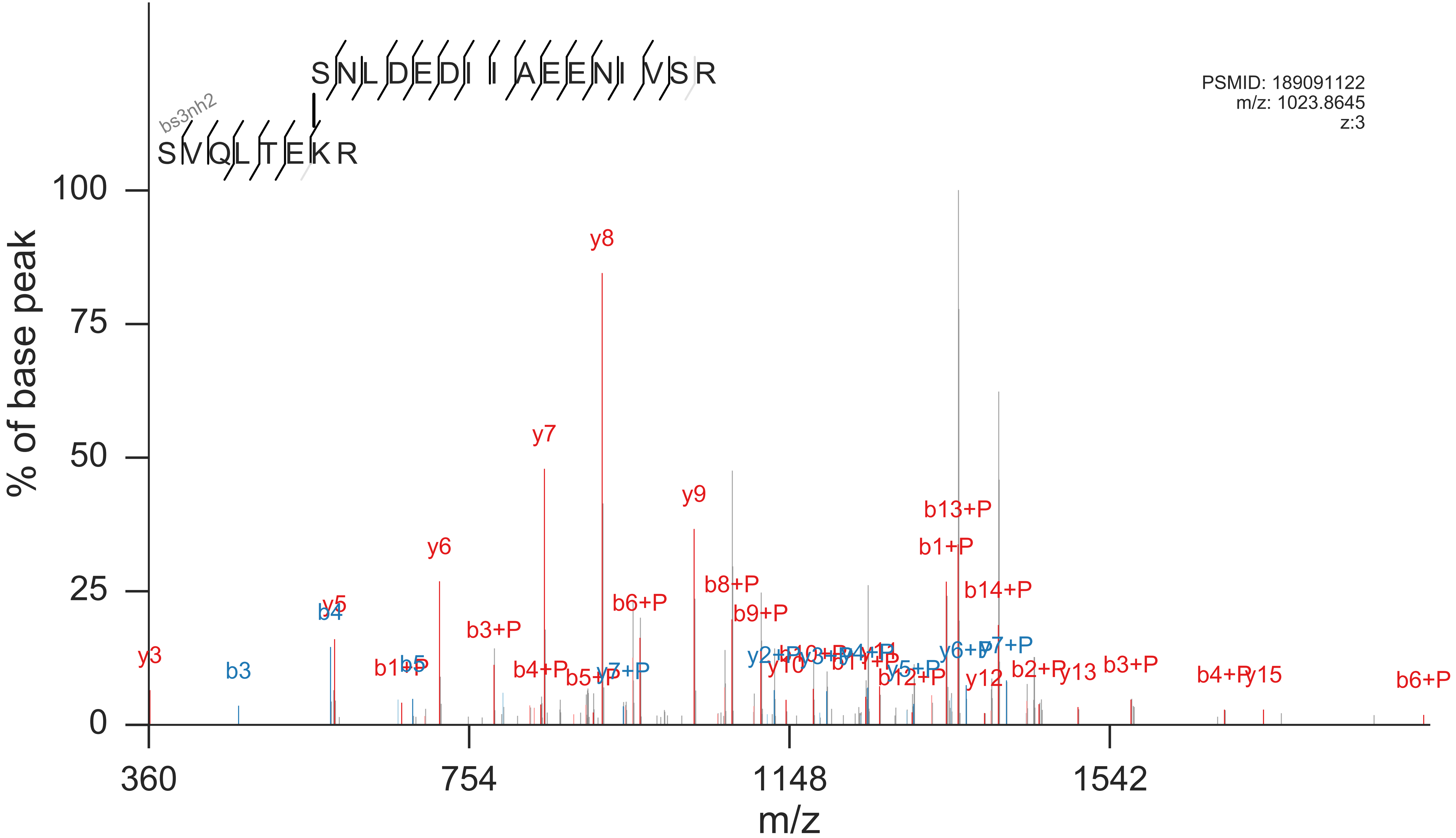
PSMID: 189091122
m/z: 1023.8645
z:3
SNLDEDIIAEENIVSR
SVQLTEKR
bs3nh2
% of base peak
m/z
y8
y7
y9
y6
b13+P
b1+P
b14+P
b8+P
b9+P
b6+P
b3+P
y5
b4
y3
b3
b1+P
b5
b4+P
b5+P
y7+P
y2+P
y10
y3+P
b10+P
b4+P
b11+P
b12+P
y5+P
y6+P
y7+P
y12
b2+P
y13
b3+P
b4+P
y15
b6+P
100
75
50
25
0
360
754
1148
1542

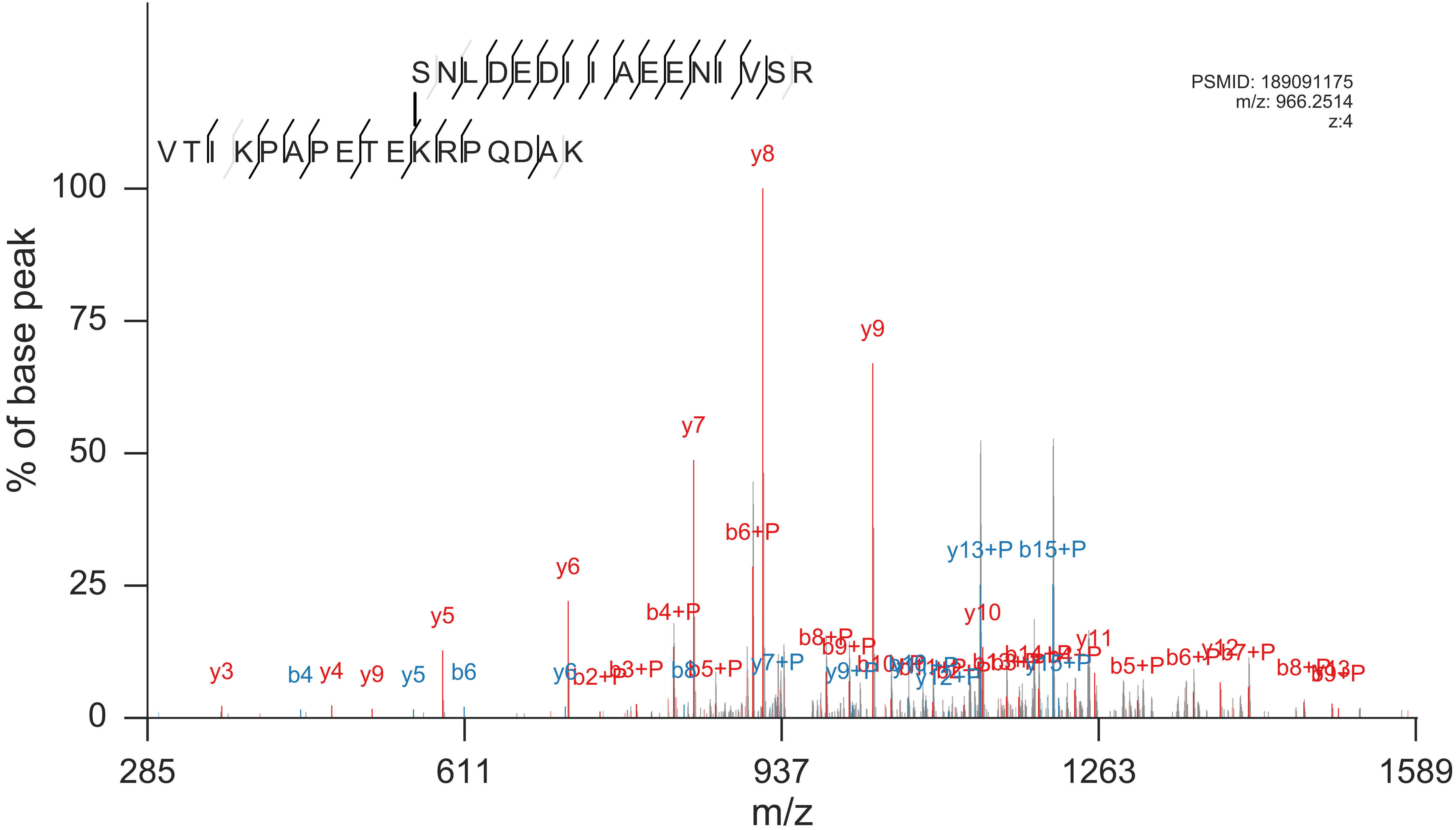

SNLDEDIIAEENIVSR
VTIKPAPETEKRPQDAK
PSMID: 189091175
m/z: 966.2514
z:4
% of base peak
m/z

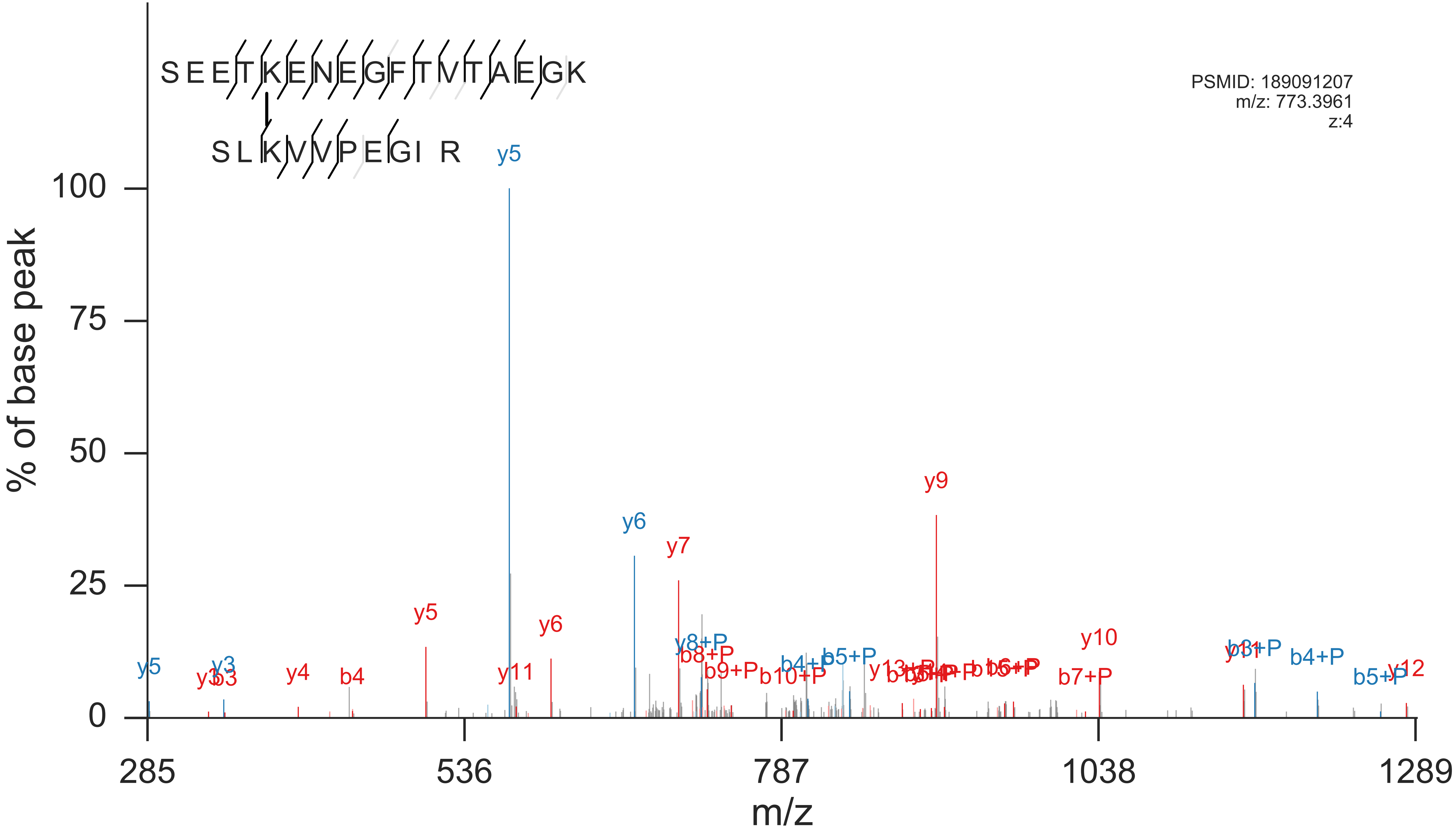

SEETKENEGFTVTAEGK
SLKVVPEGIR
PSMID: 189091207
m/z: 773.3961
z:4
% of base peak
m/z
0
25
50
75
100
285
536
787
1038
1289
y5
y6
y7
y9
y10
y11
y12
b4
b7+P
b9+P
b10+P
b8+P
y8+P
b4+P
b5+P

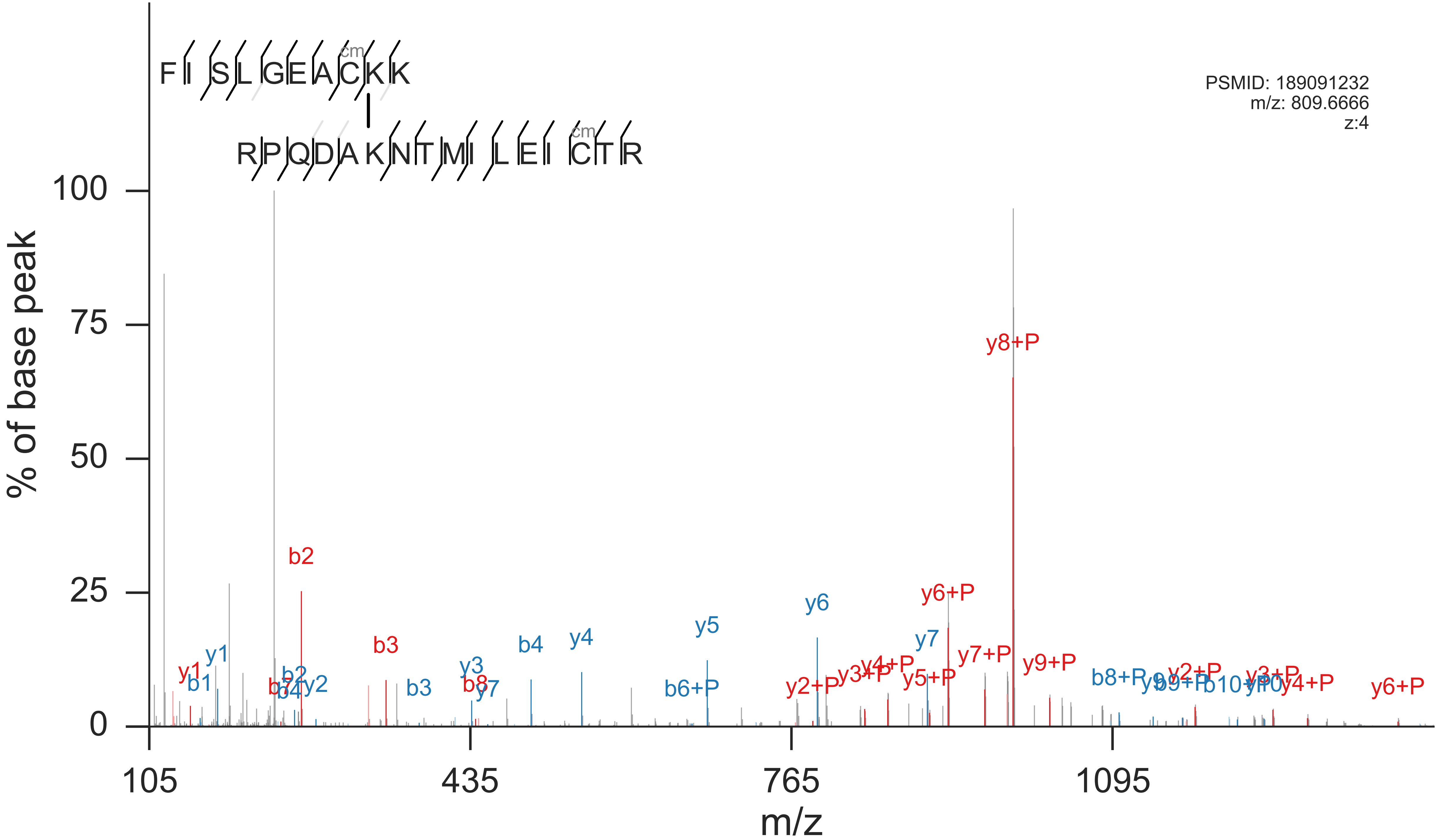

FISLGEACKK
RPQDAKNTMILEICTR
cm
PSMID: 189091232
m/z: 809.6666
z:4
% of base peak
m/z
100
75
50
25
0
105
435
765
1095
y8+P
b2
y6+P
y6
y5
y7
y7+P
y9+P
y4+P
y3+P
y2+P
y5+P
b3
b4
y4
y3
b8
y7
b3
b6+P
b8+P
b10+P
y1
y1
b1
b2
y2
y6+P

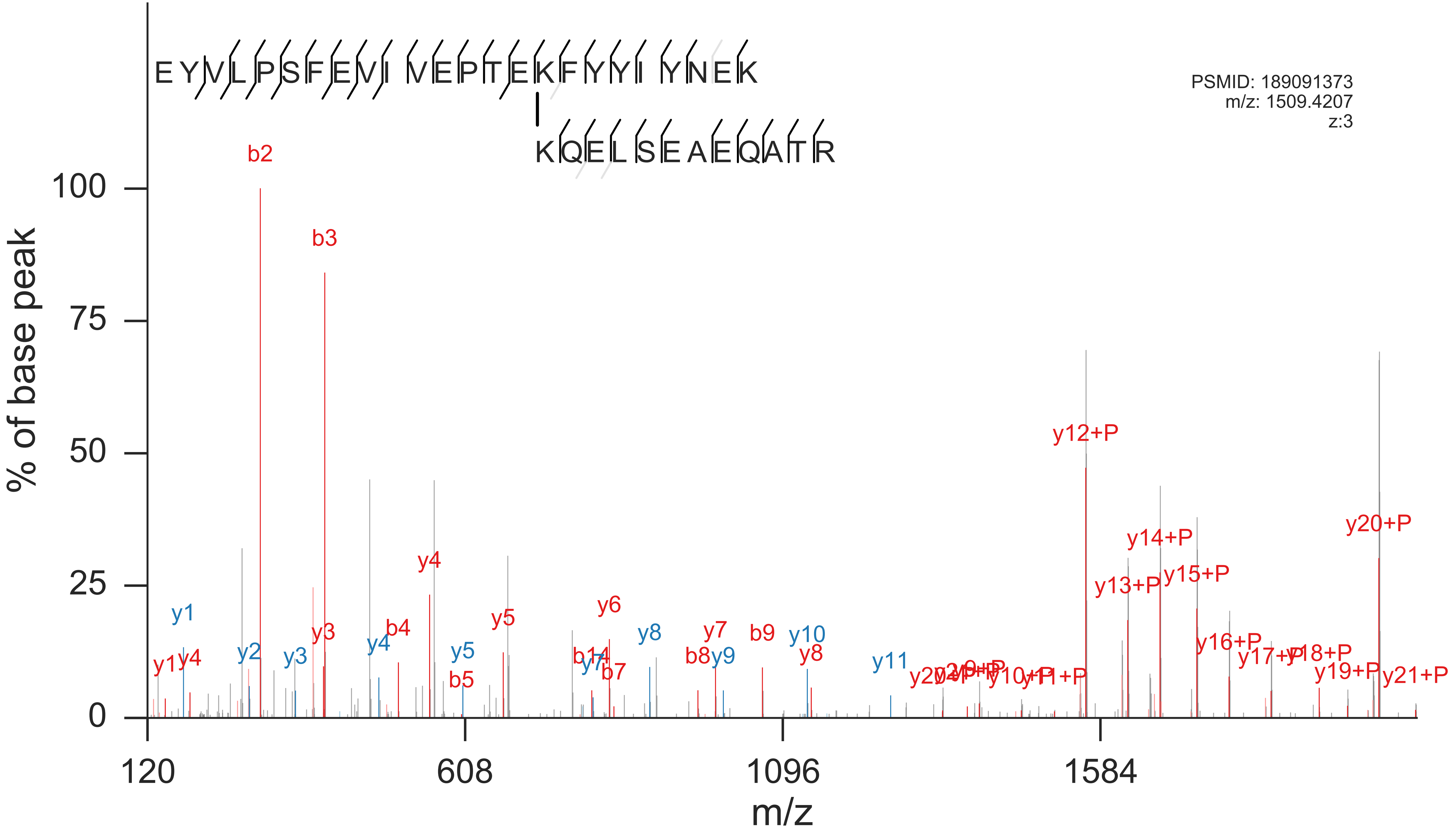
EYVLPSFEVIVEPTEKFYYIYNEK
KQELSEAEQATR
PSMID: 189091373
m/z: 1509.4207
z:3
% of base peak
m/z
0
25
50
75
100
120
608
1096
1584
b2
b3
y1
y4
y2
y3
y4
b4
y5
b5
y6
b14
y7
b7
y8
b8
y9
y7
b9
y10
y8
y11
y12+P
y13+P
y14+P
y15+P
y16+P
y18+P
y19+P
y20+P
y21+P

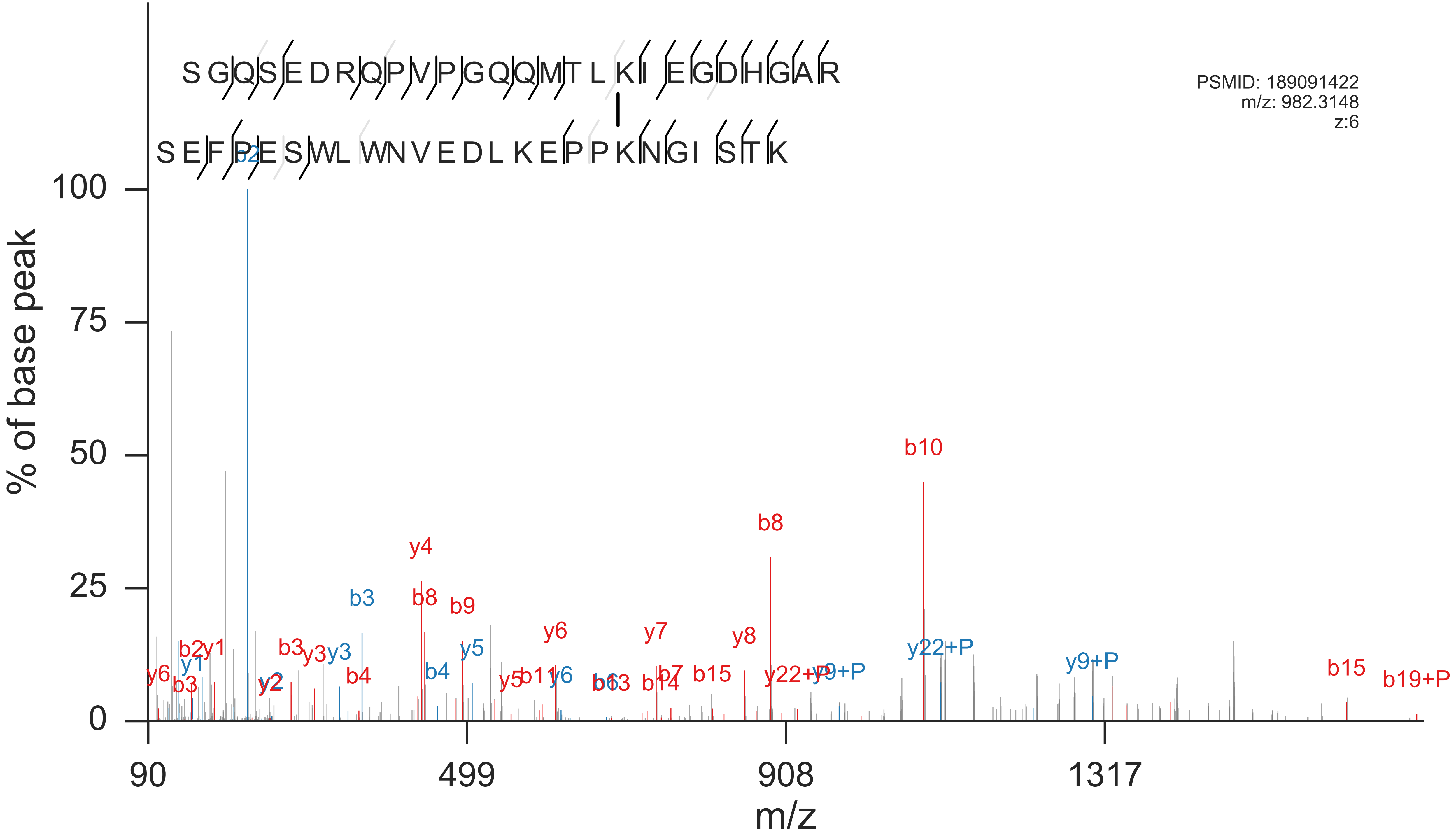

S G Q S E D R Q P V P G Q Q M T L K I E G D H G A R
S E F P E S W L W N V E D L K E P P K N G I S T K
PSMID: 189091422
m/z: 982.3148
z:6
% of base peak
m/z

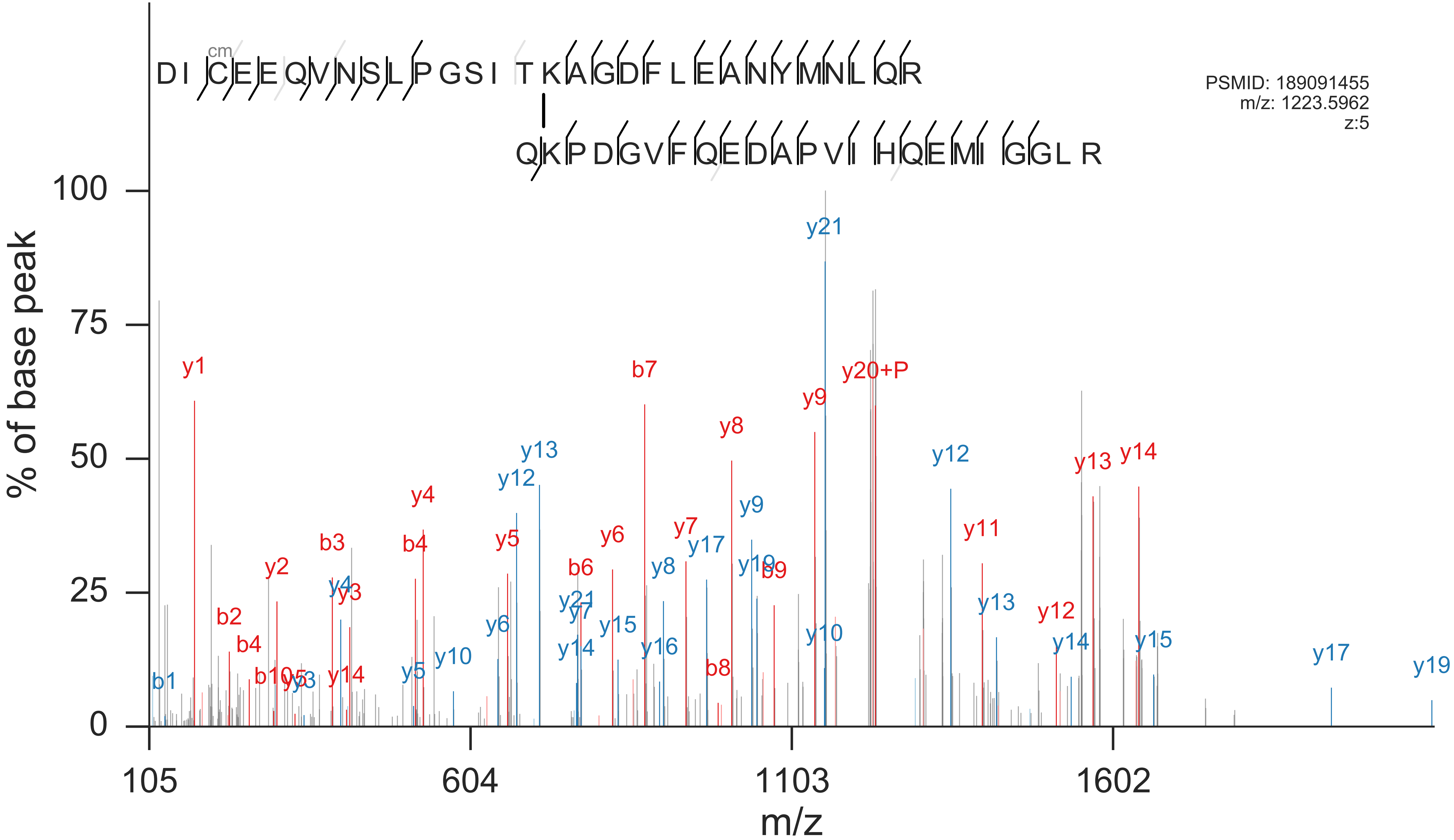

DI CEEQVNSLPGSI TKAGDFLEANYMNLQR
QKPDGVFQEDAPVI HQEMI GGLR
cm
PSMID: 189091455
m/z: 1223.5962
z:5
% of base peak
m/z
0
25
50
75
100
105
604
1103
1602

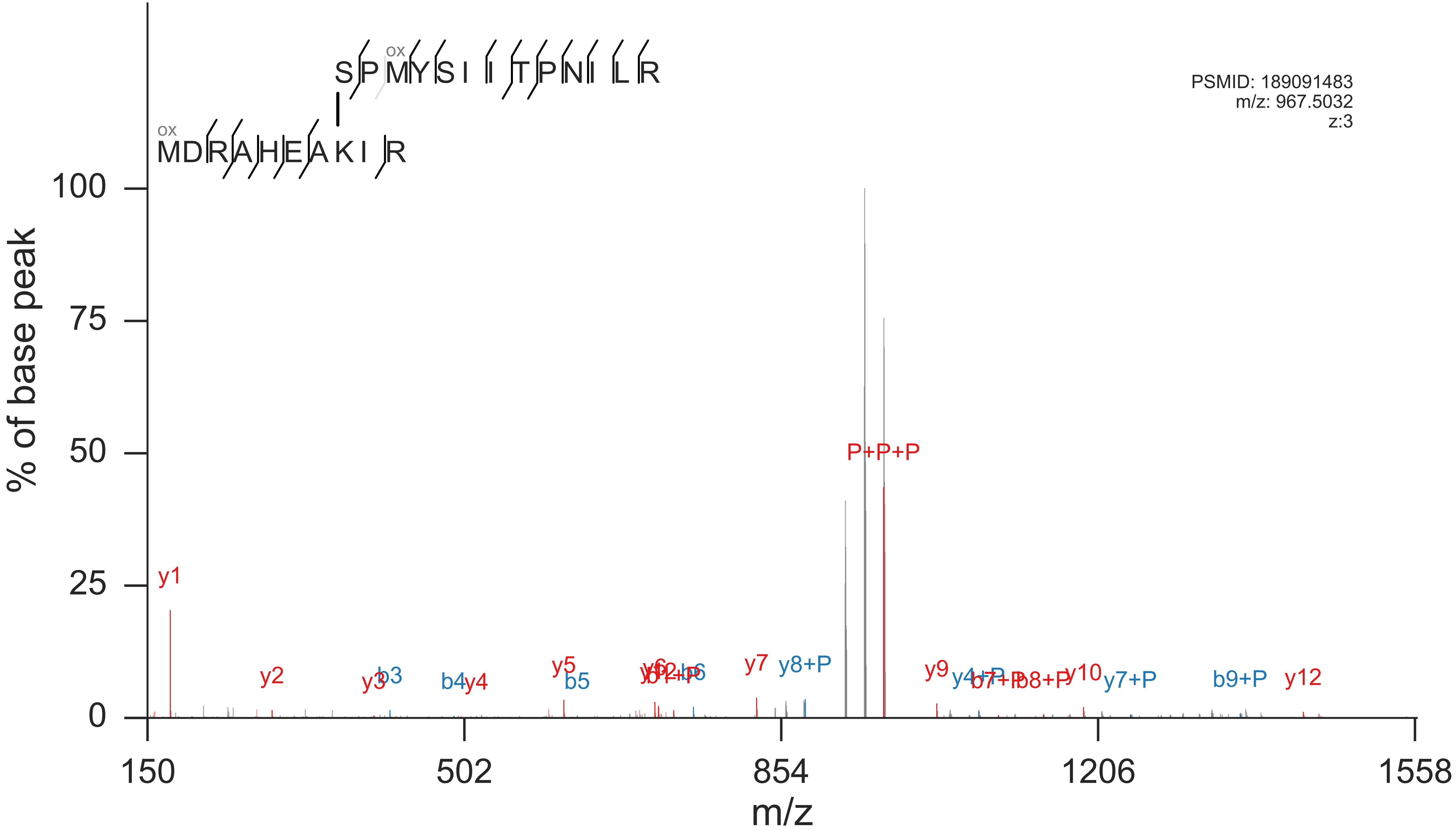
SPMYSIITPNILR
MDRAHEAKIR
PSMID: 189091483
m/z: 967.5032
z:3
% of base peak
m/z
P+P+P
y1
y2
y3
b3
b4
y4
y5
b5
y7
y8+P
y9
y10
y7+P
b9+P
y12

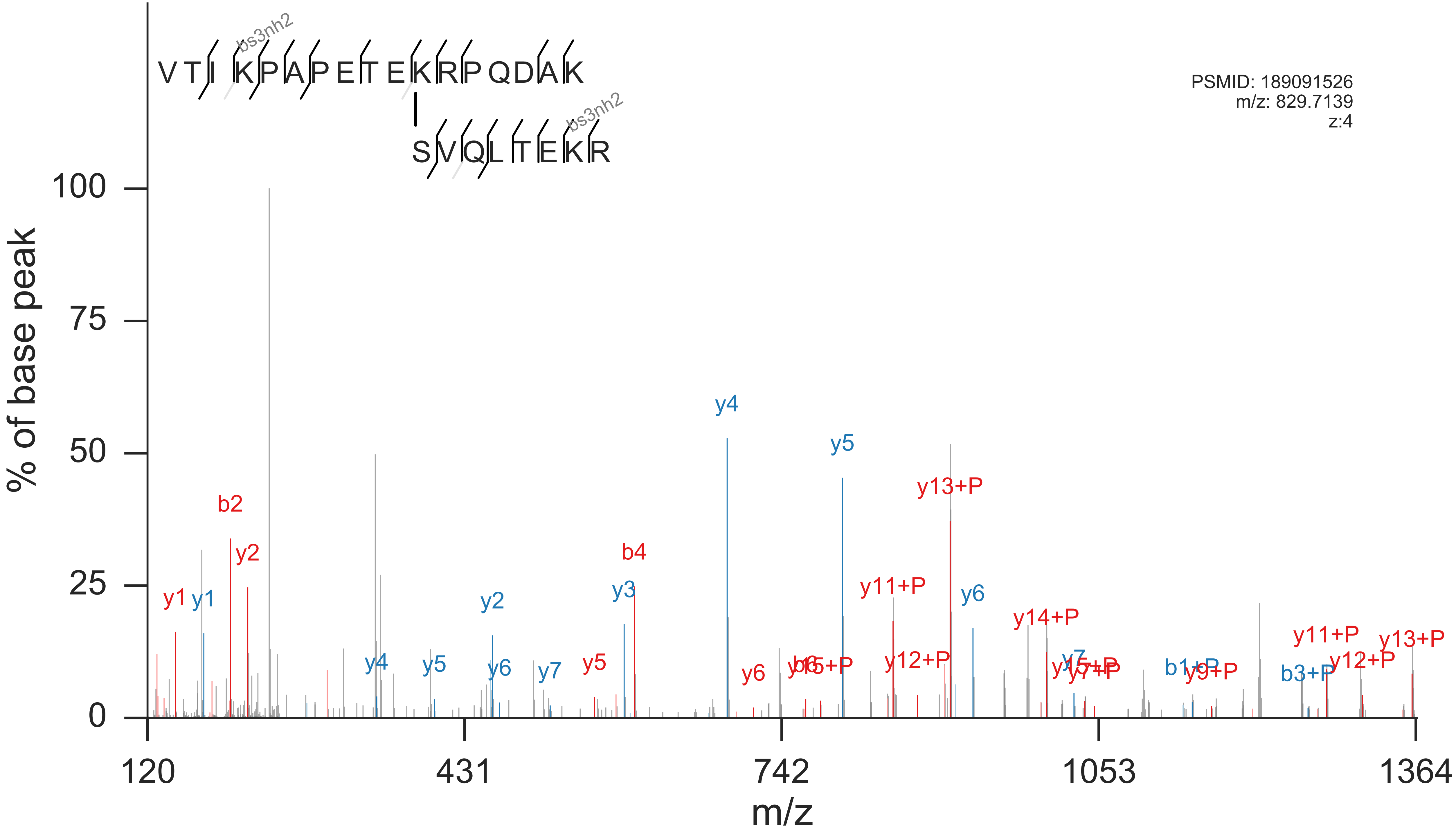
VTIKPAPETEKRPQDAK
SVQLTEKR
bs3nh2
PSMID: 189091526
m/z: 829.7139
z:4
% of base peak
m/z
0
25
50
75
100
120
431
742
1053
1364
y1
y1
b2
y2
y4
y5
y2
y6
y7
y5
y3
b4
y4
y6
y5
y11+P
y12+P
y13+P
y6
y14+P
y11+P
y12+P
y13+P
b1+P
y9+P
b3+P

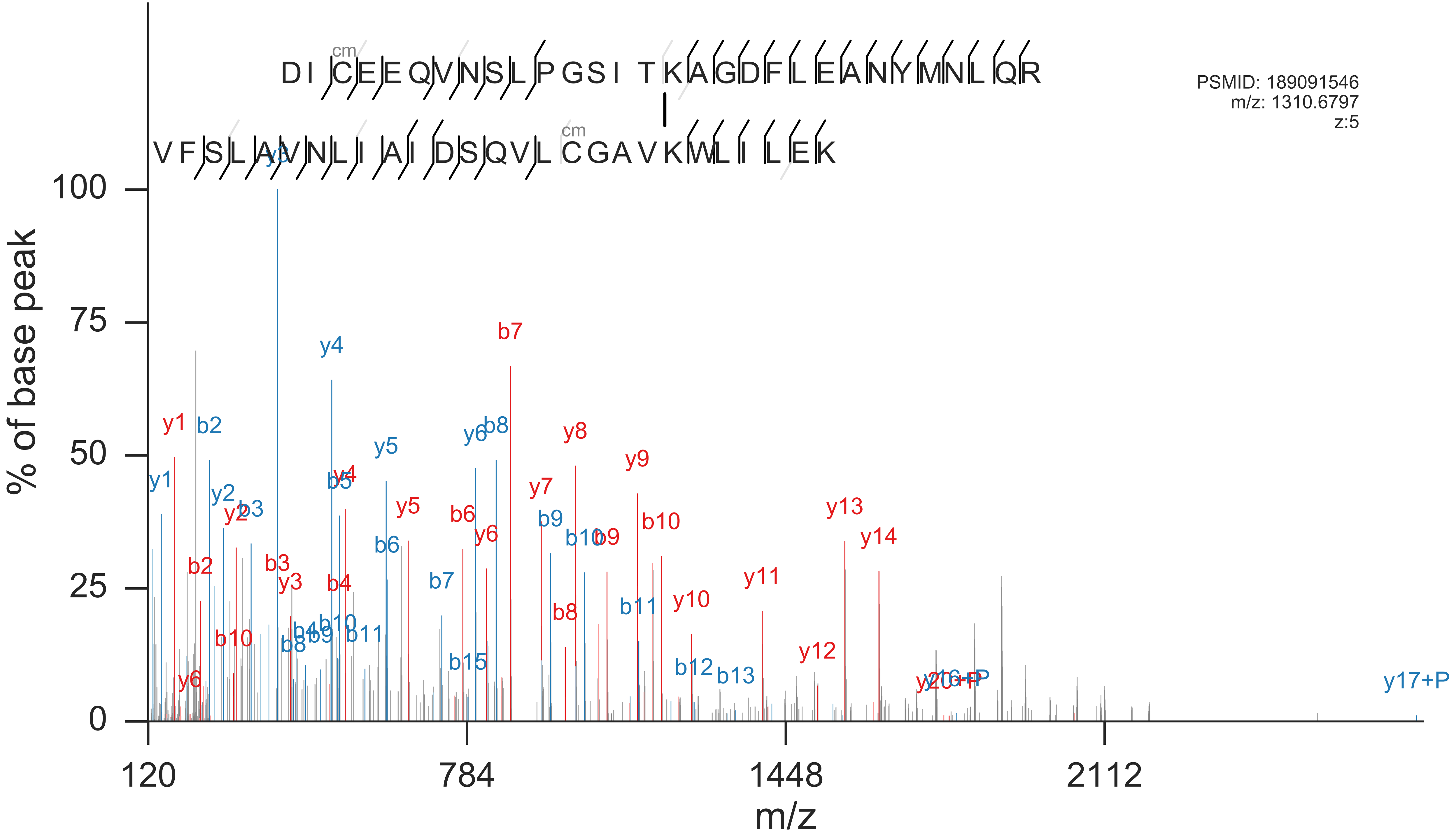

DI CEEQVNSLPGSI TKAGDFLEANYMNLQR
VFSLAVNLI AI DSQVLCGAVKWLI LEK
cm
cm
PSMID: 189091546
m/z: 1310.6797
z:5
% of base peak
100
75
50
25
0
120
784
1448
2112
m/z
y1
b2
y2
b3
y3
y4
b5
y5
y6
b8
b6
b7
b9
b10
b11
b12
b13
b15
b7
y8
y9
y7
y13
y14
y11
y10
y12
b10
b9
b8
y6
y17+P

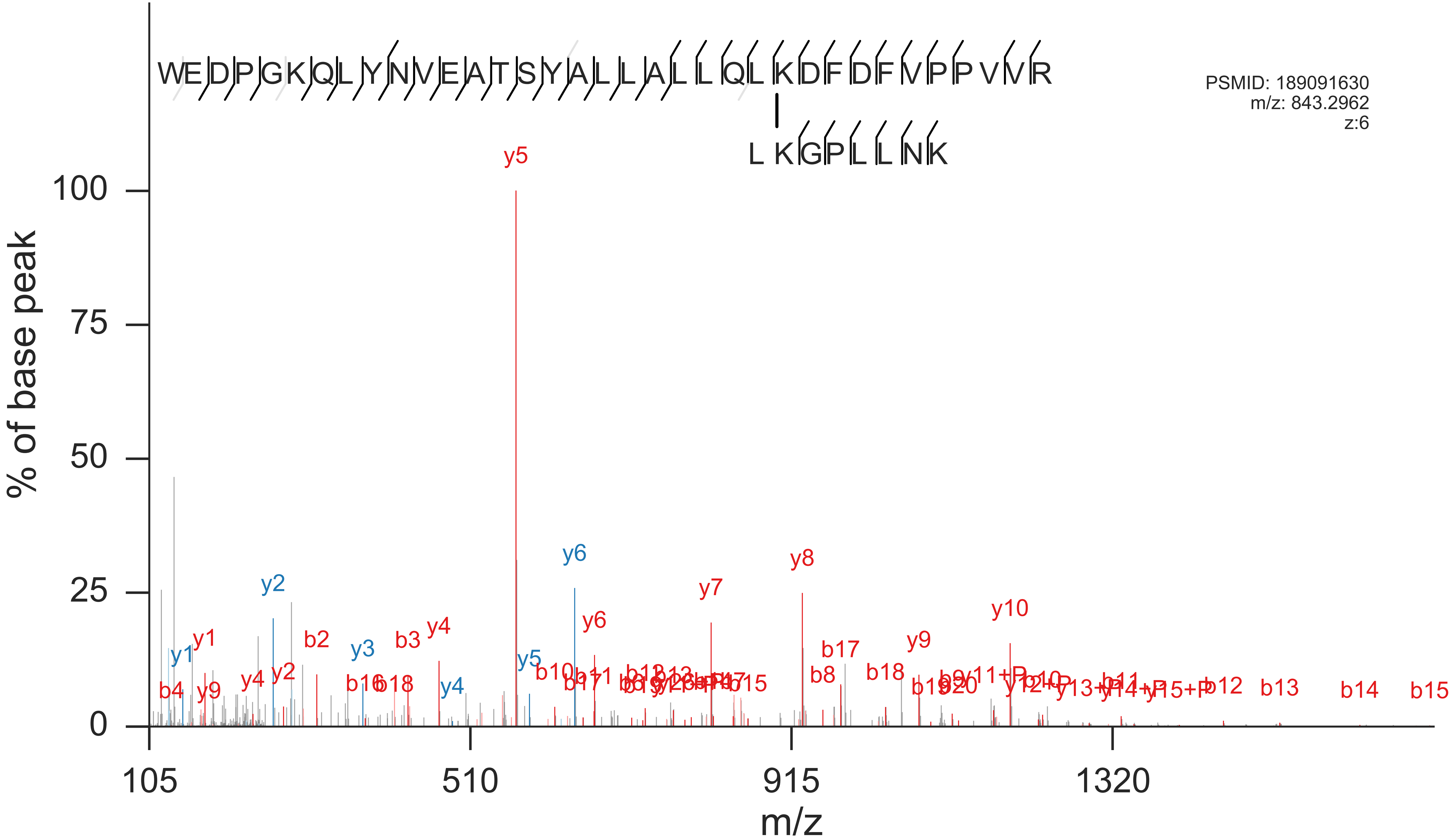

WEDPGKQLYNVEATSYALLALLQLKDFDFVPPVVR
LKGPLLNK
PSMID: 189091630
m/z: 843.2962
z:6
% of base peak
m/z

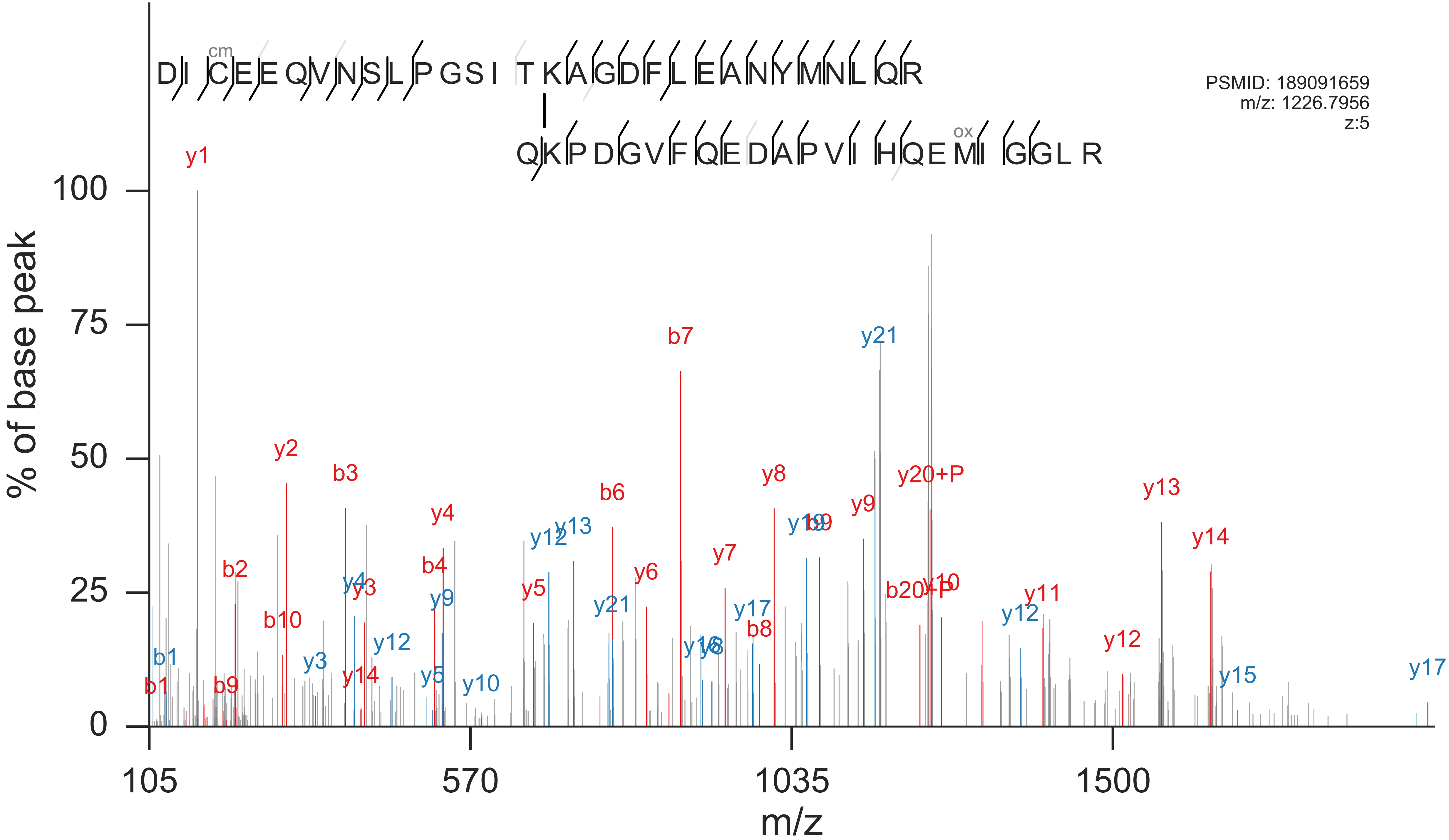
DICEEQVNSLPGSITKAGDFLEANYMNLQR
cm
QKPDGVFQEDAPVIHQEMIGGLR
ox
PSMID: 189091659
m/z: 1226.7956
z:5
% of base peak
m/z
0
25
50
75
100
105
570
1035
1500

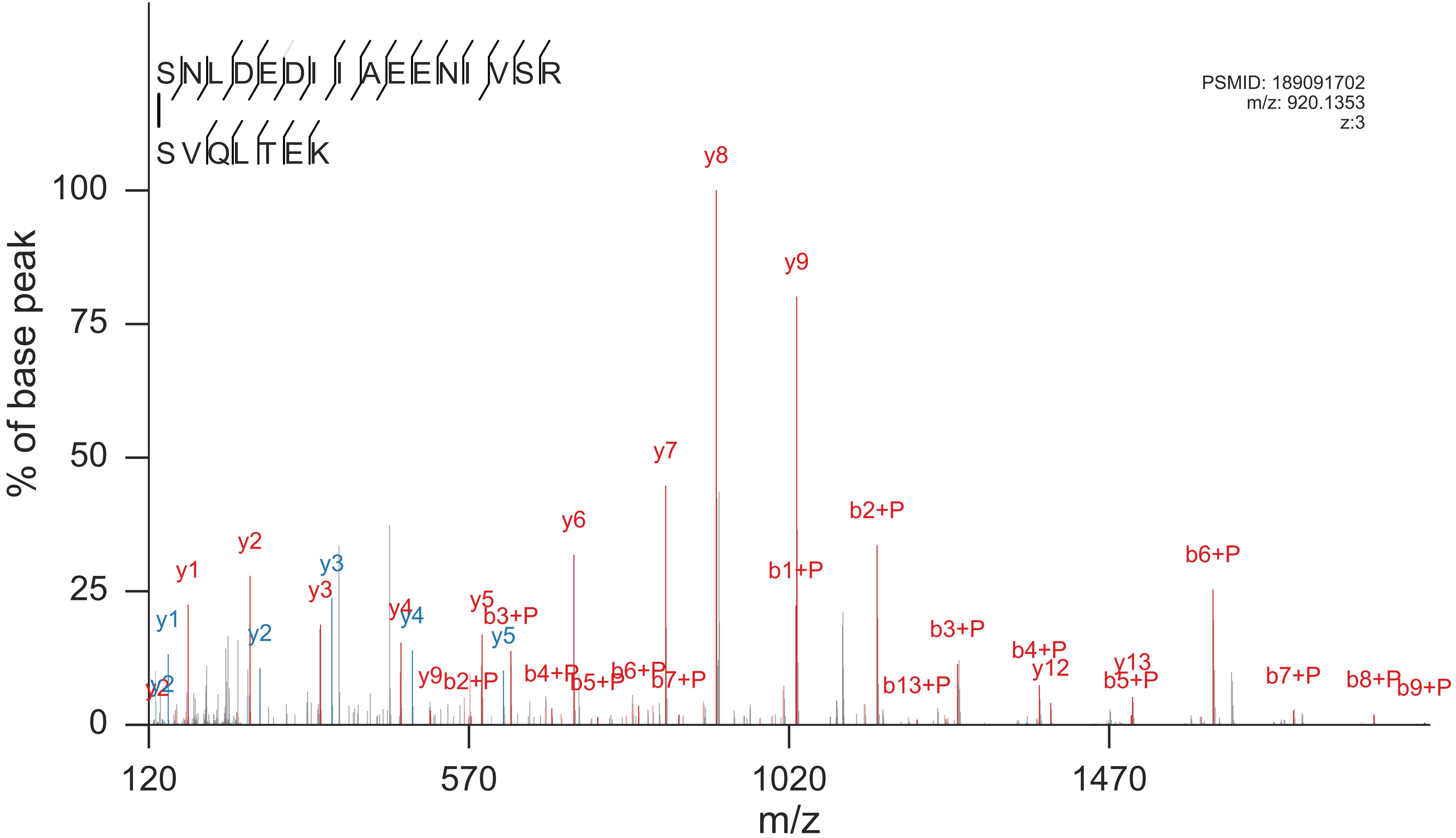

SNLDEDIIAEENIVSR
SVQLTEK
PSMID: 189091702
m/z: 920.1353
z:3
% of base peak
m/z
100
75
50
25
0
120
570
1020
1470
y8
y9
y7
y6
y2
y1
y3
y4
y5
b2+P
b1+P
b6+P
b3+P
b4+P
b5+P
b6+P
b7+P
b8+P
b9+P
b13+P
y12
y13
y9

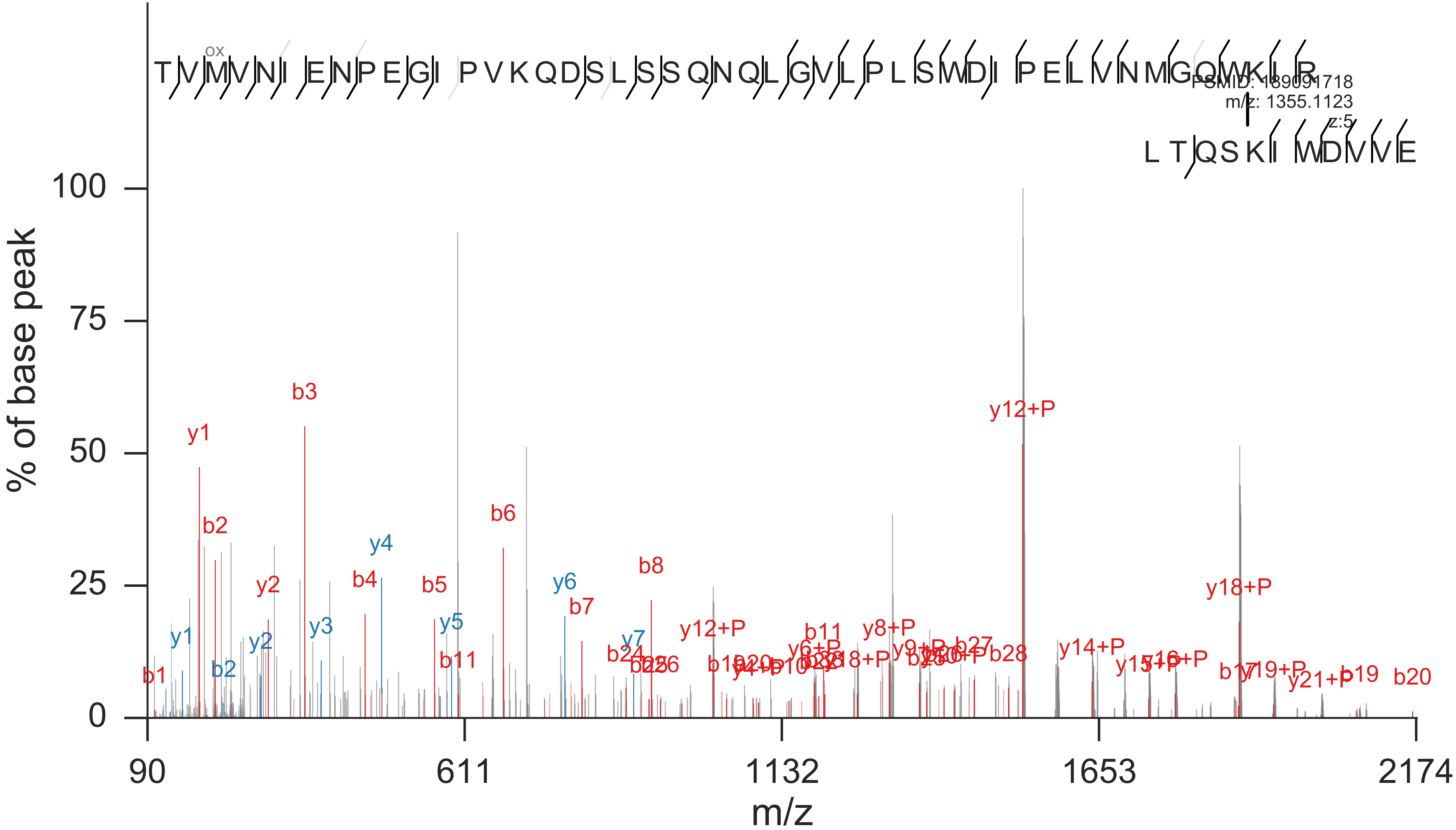

PSMID: 189091718
m/z: 1355.1123
z:5
TVMVNIENPEGIPVKQDSLSSQNQLGVLPLSWDIPELVNMGQWKLR
LTQSKIWDVVE
% of base peak
m/z

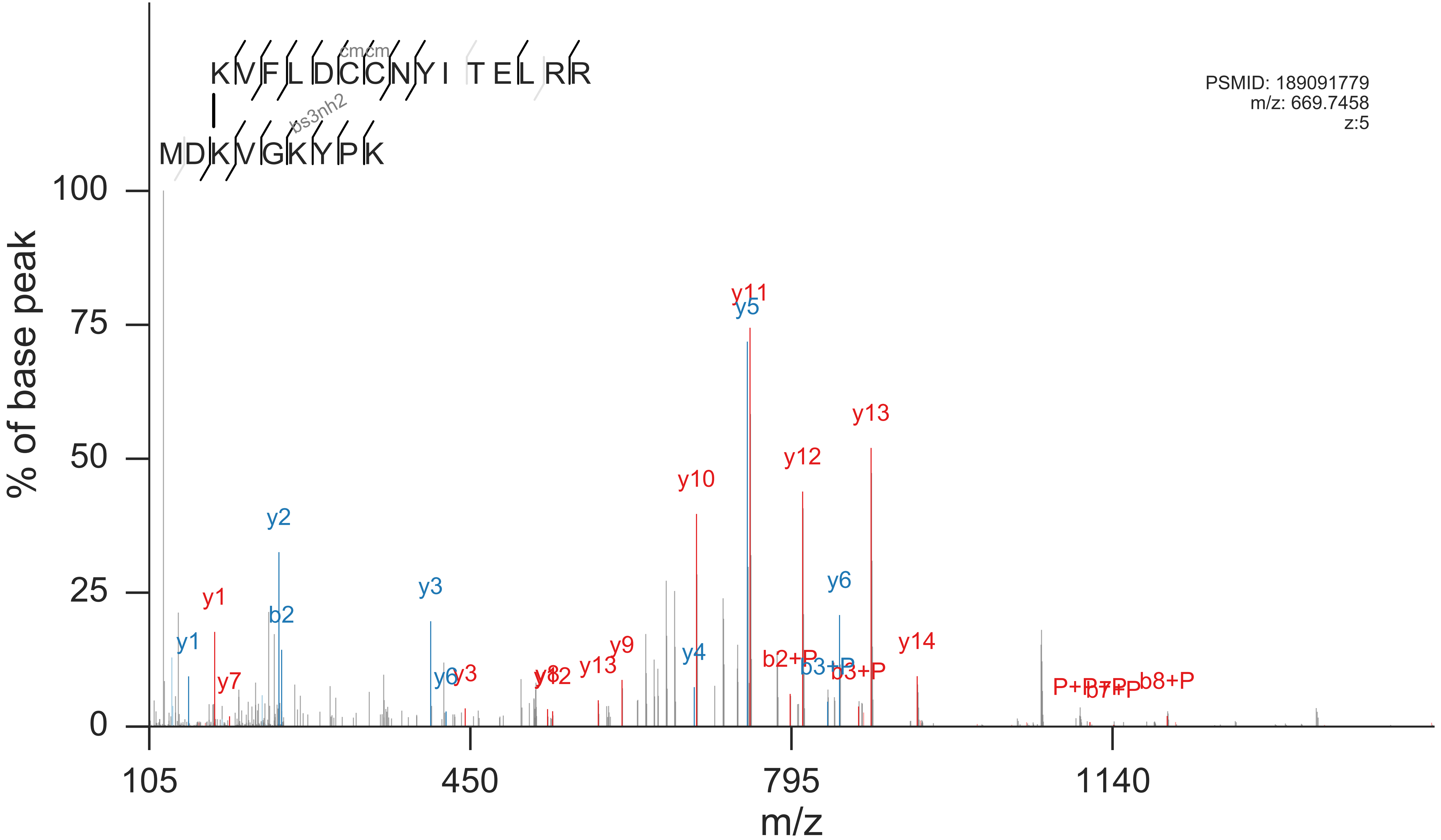

KVFLDCCNYITELRR
MDKVGKYPK
cmcm
bs3nh2
PSMID: 189091779
m/z: 669.7458
z:5
% of base peak
m/z
100
75
50
25
0
105
450
795
1140
y1
y2
b2
y3
y6
y4
y5
y6
y1
y7
y3
y8
y12
y13
y9
y10
y11
y12
y13
y14
b2+P
b3+P
b3+P
P+P
b7+P
b8+P

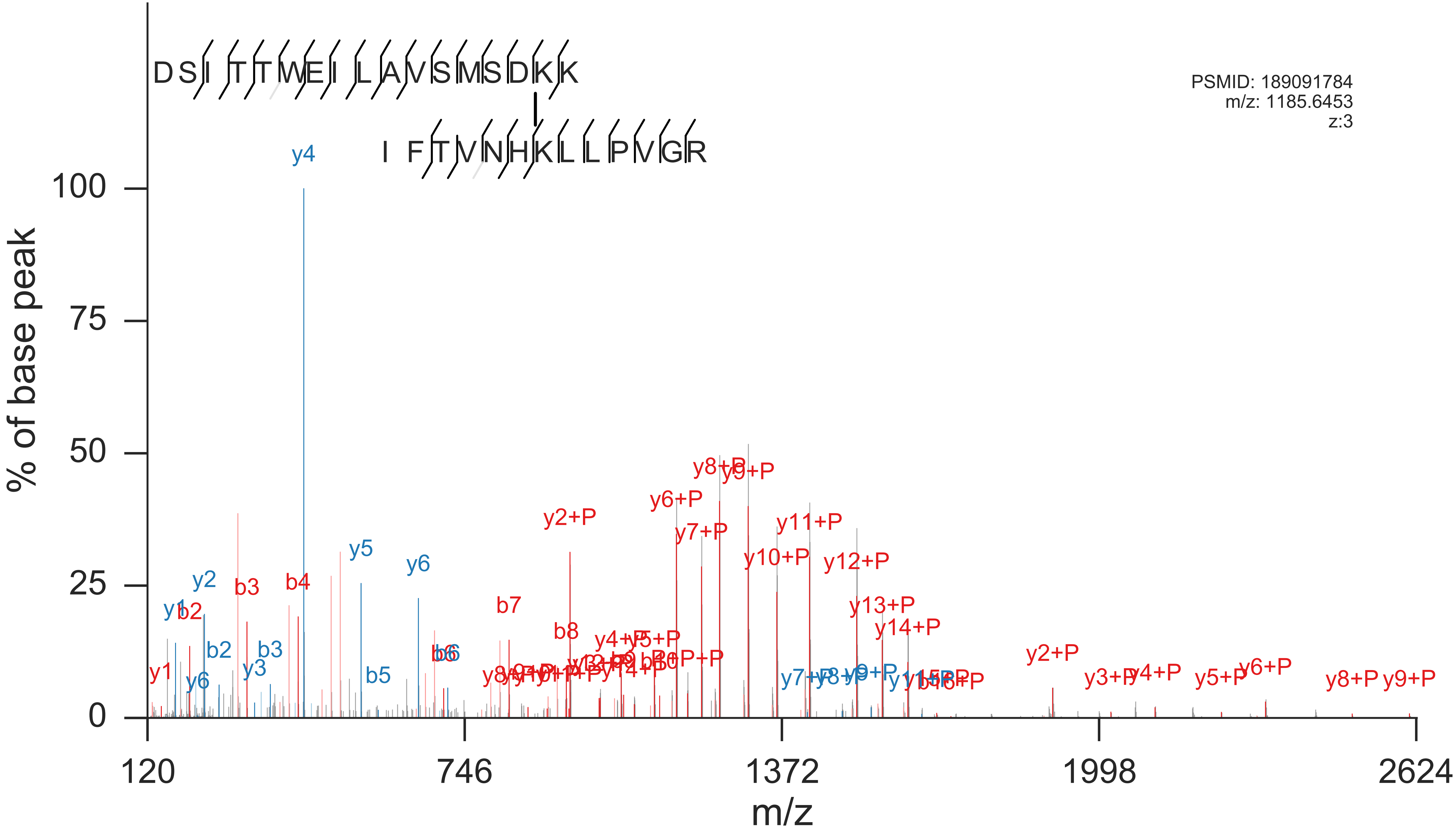

DSITTWEILAVSMSDKK
IFTVNHKLLPVGR
PSMID: 189091784
m/z: 1185.6453
z:3
% of base peak
m/z
100
75
50
25
0
120
746
1372
1998
2624

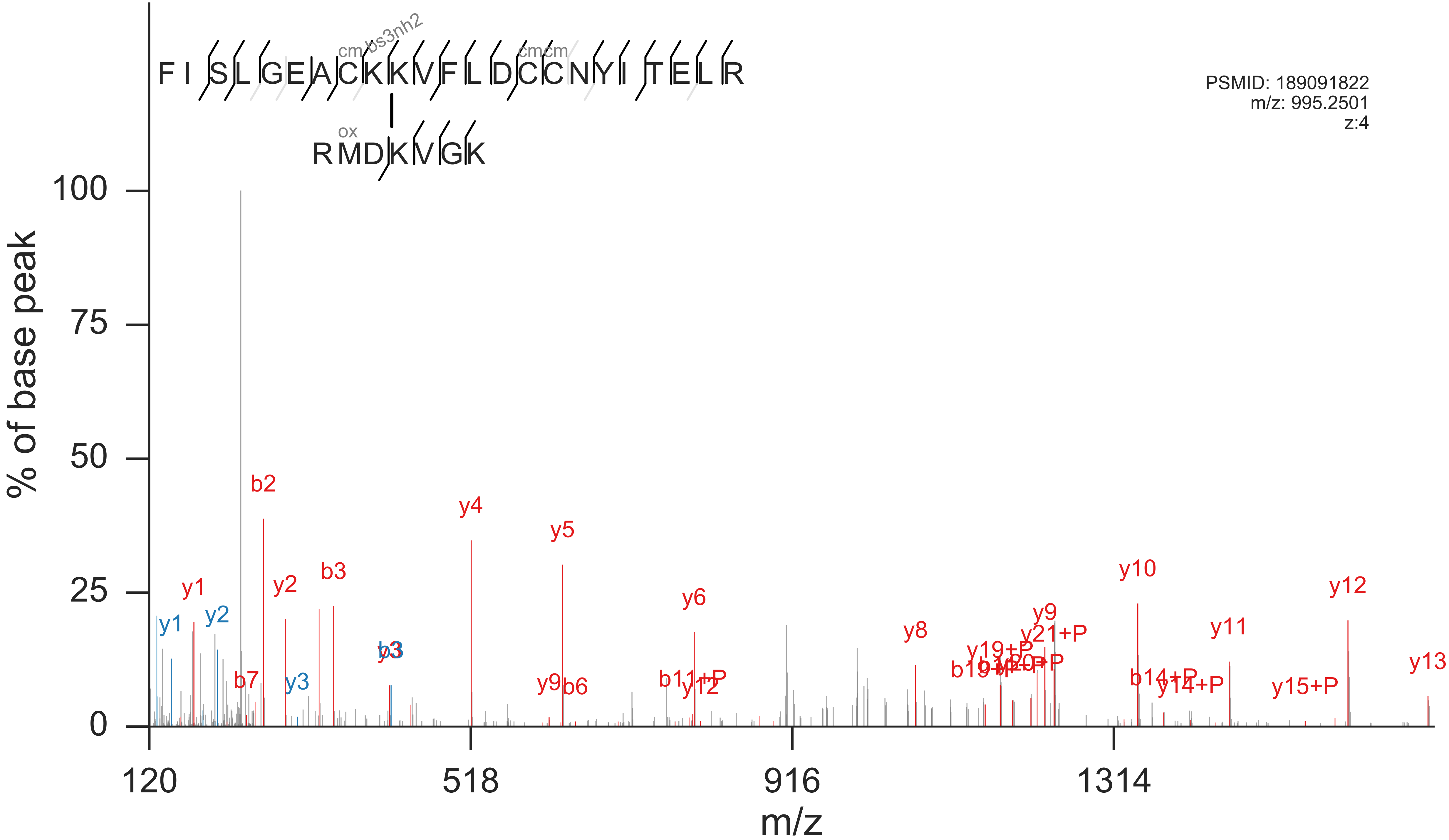
FISLGEACKKVFLDCCNYITELR
RMDKVGK
cm
bs3nh2
cmcm
ox
PSMID: 189091822
m/z: 995.2501
z:4
% of base peak
m/z
100
75
50
25
0
120
518
916
1314
b2
y4
y5
y1
y2
b3
y6
y10
y12
y9
y21+P
y8
y11
y13
y3
b7
b6
y14+P
y15+P

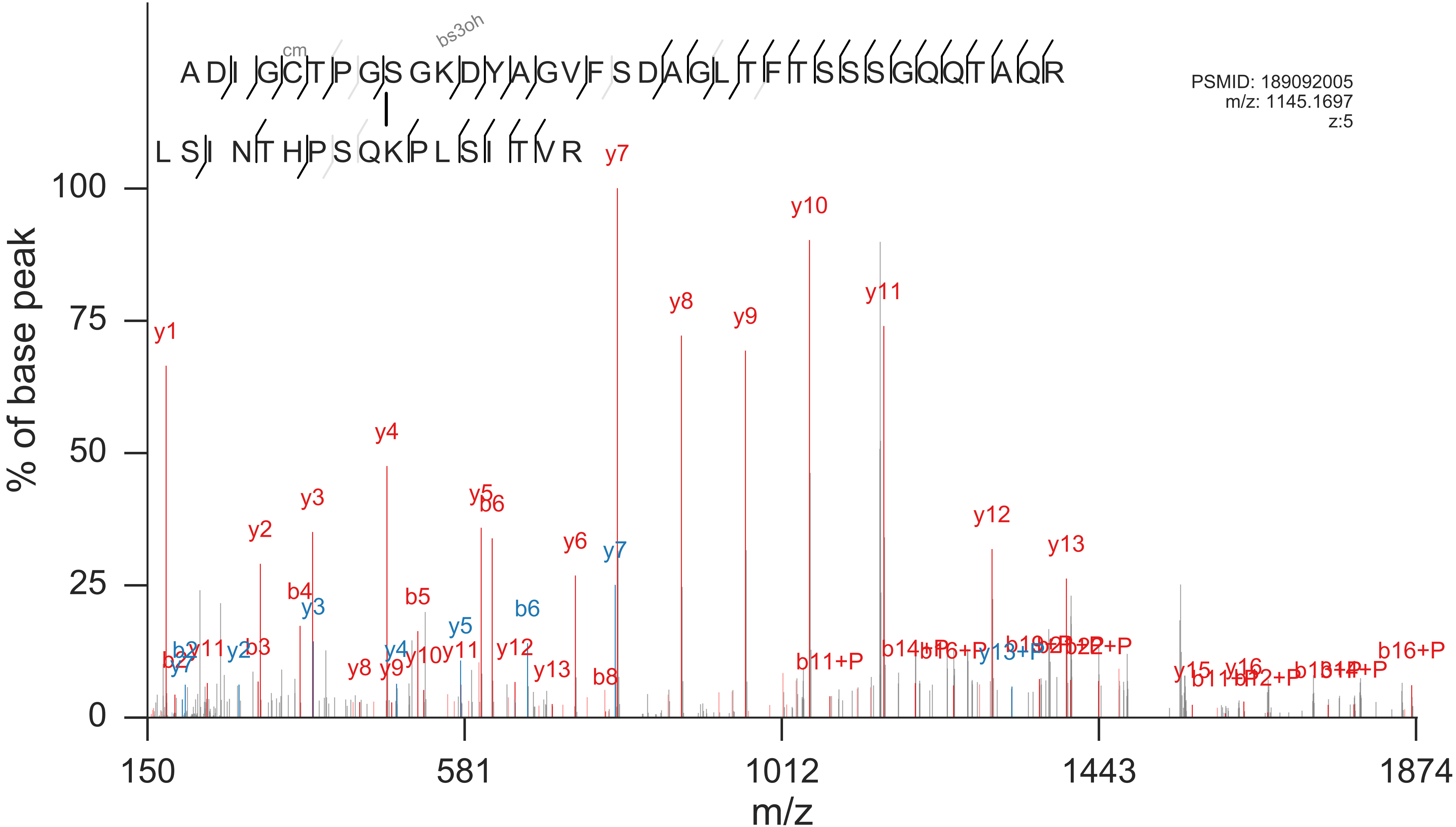

A D I G C T P G S G K D Y A G V F S D A G L T F T S S S G Q Q T A Q R
cm
bs3oh
L S I N T H P S Q K P L S I T V R
PSMID: 189092005
m/z: 1145.1697
z:5
% of base peak
m/z
0
25
50
75
100
150
581
1012
1443
1874

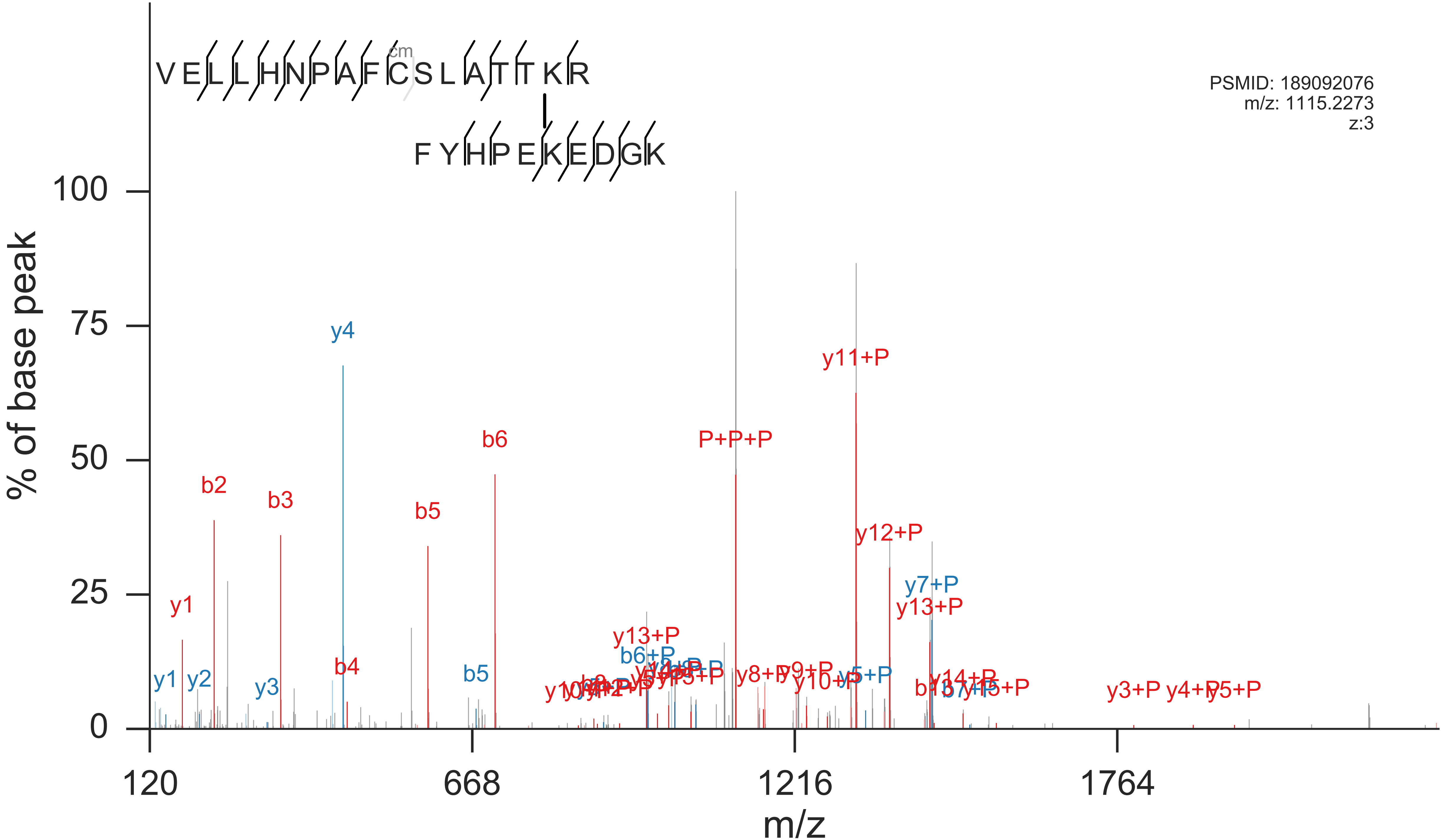
VELLHNPAFCSLATTKR
cm
FYHPEKEDGK
PSMID: 189092076
m/z: 1115.2273
z:3
% of base peak
100
75
50
25
0
m/z
120
668
1216
1764
y4
y11+P
P+P+P
b6
b2
b3
b5
y12+P
y7+P
y13+P
y1
y13+P
b6+P
b4
b5
y1
y2
y3
y8+P
y10+P
y5+P
y14+P
y3+P
y4+P
y5+P

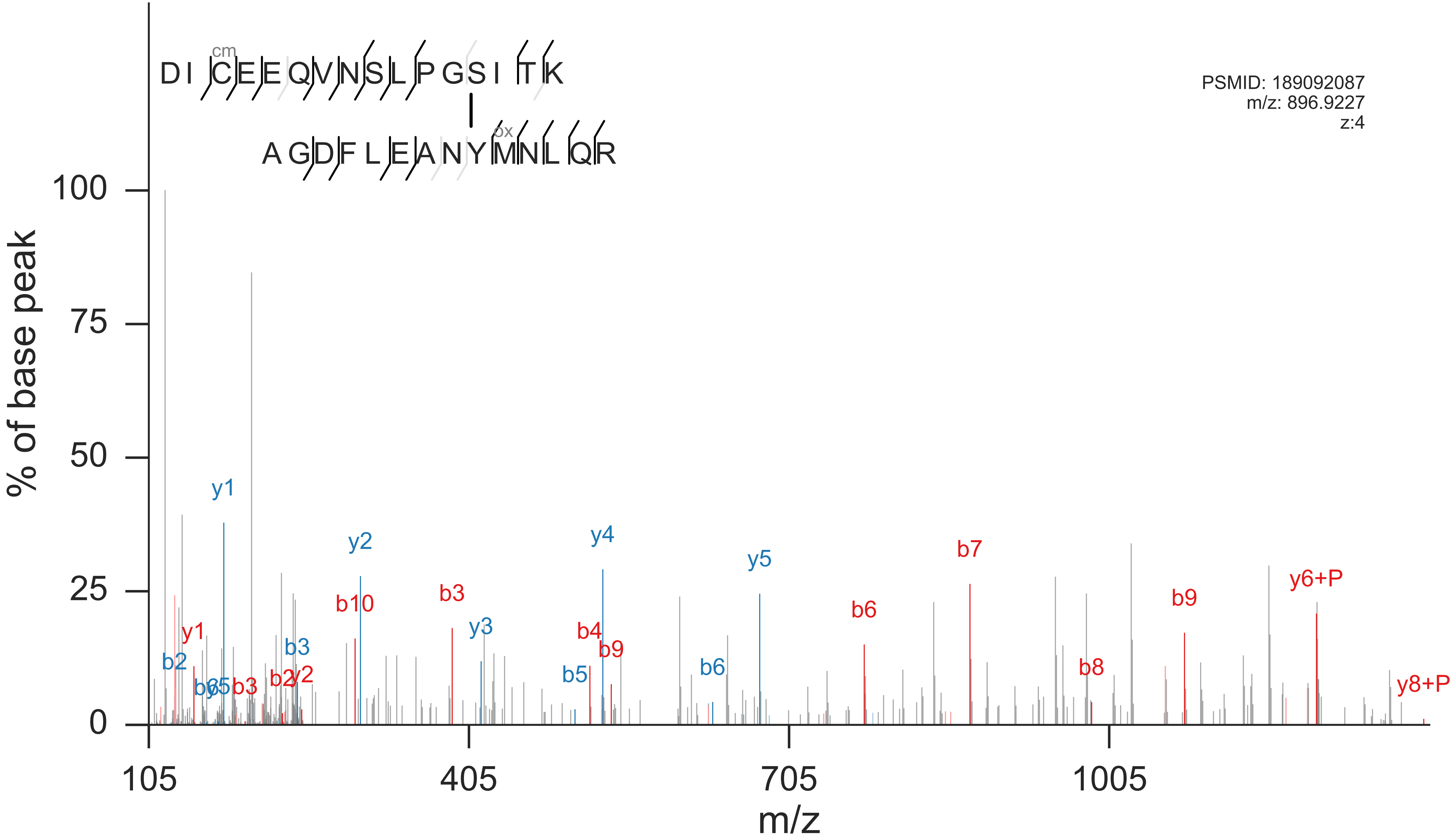

DI CEEQVNSLPGSITK
cm
AGDFLEANYMNLQR
ox
PSMID: 189092087
m/z: 896.9227
z:4
% of base peak
m/z
100
75
50
25
0
105
405
705
1005
y1
y2
y4
y5
b7
y6+P
b9
b6
b10
b3
y3
b4
b9
b2
y1
b3
b2
y2
b5
b6
b8
y8+P

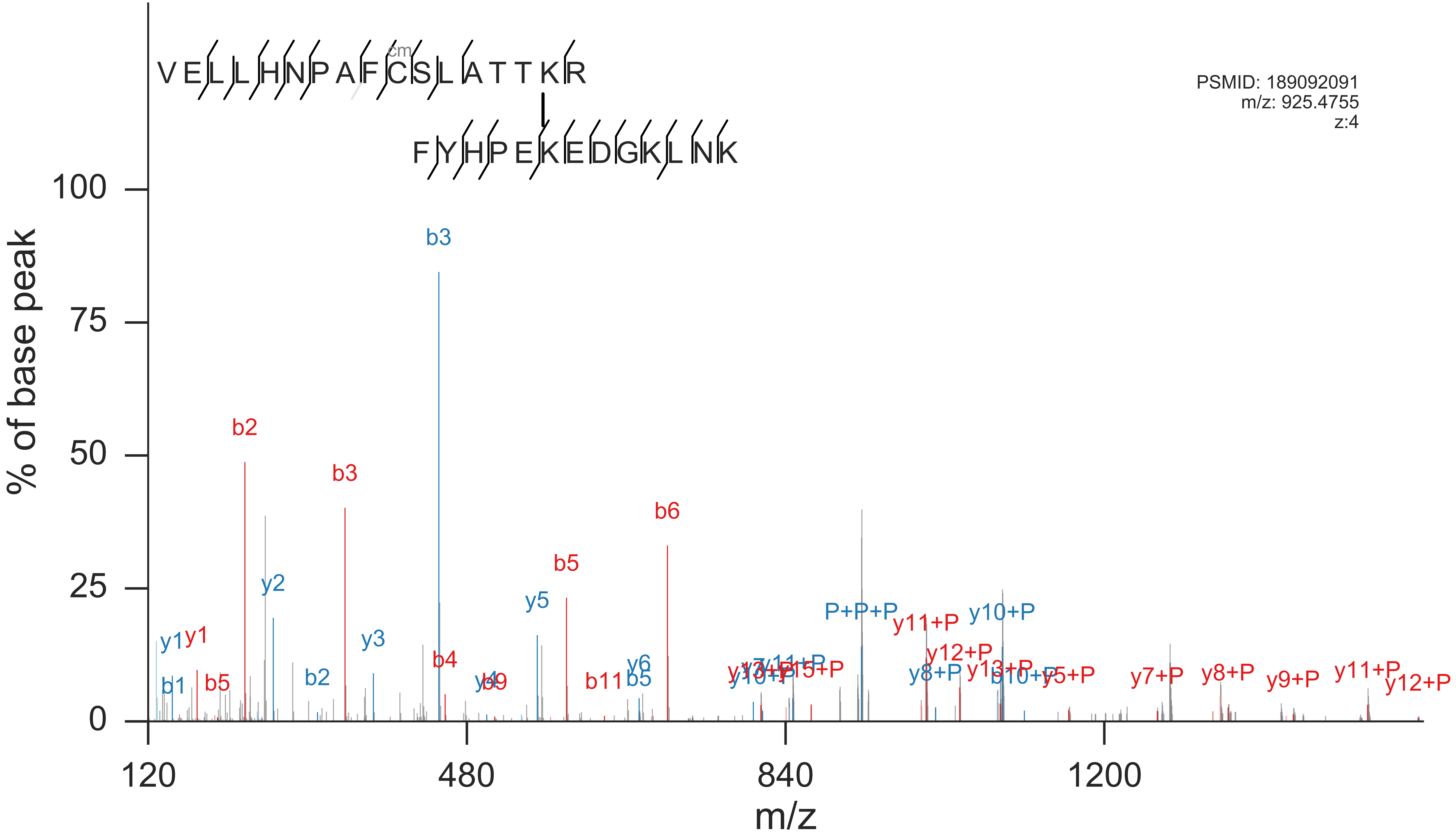
VELLHNPAFCSLATTKR
cm
FYHPEKEDGKLNK
PSMID: 189092091
m/z: 925.4755
z:4
% of base peak
100
75
50
25
0
120
480
840
1200
m/z
b3
b2
b3
b6
b5
y2
y5
y1
y1
b1
b5
b2
y3
b4
b9
y6
b5
b11
P+P+P
y11+P
y10+P
y12+P
y8+P
y13+P
b10+P
y5+P
y7+P
y8+P
y9+P
y11+P
y12+P
b15+P

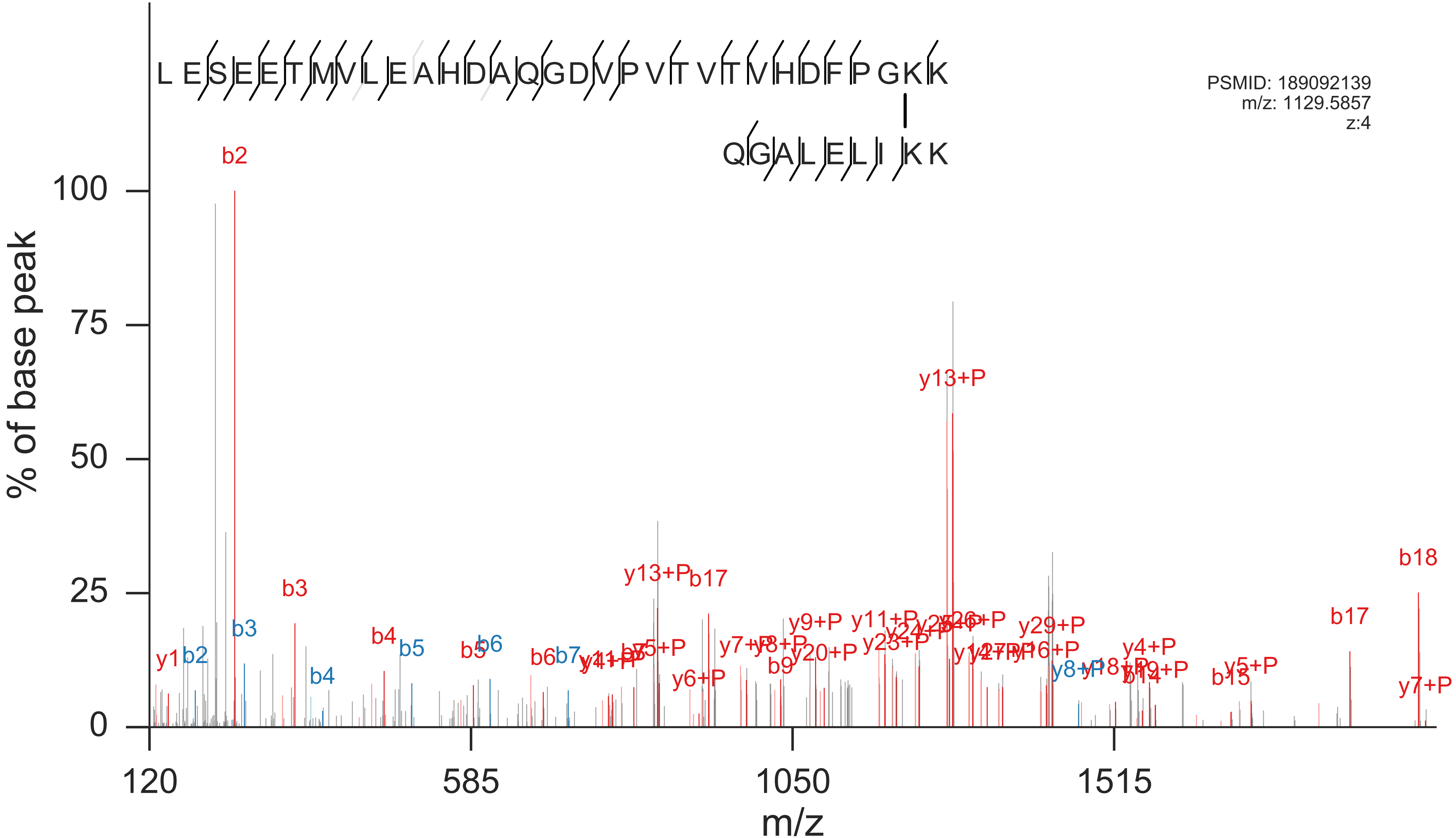

LESEETMVLEAHDAQGDVPVTVTVHDFPGKK
QGALELIKK
PSMID: 189092139
m/z: 1129.5857
z:4
% of base peak
m/z

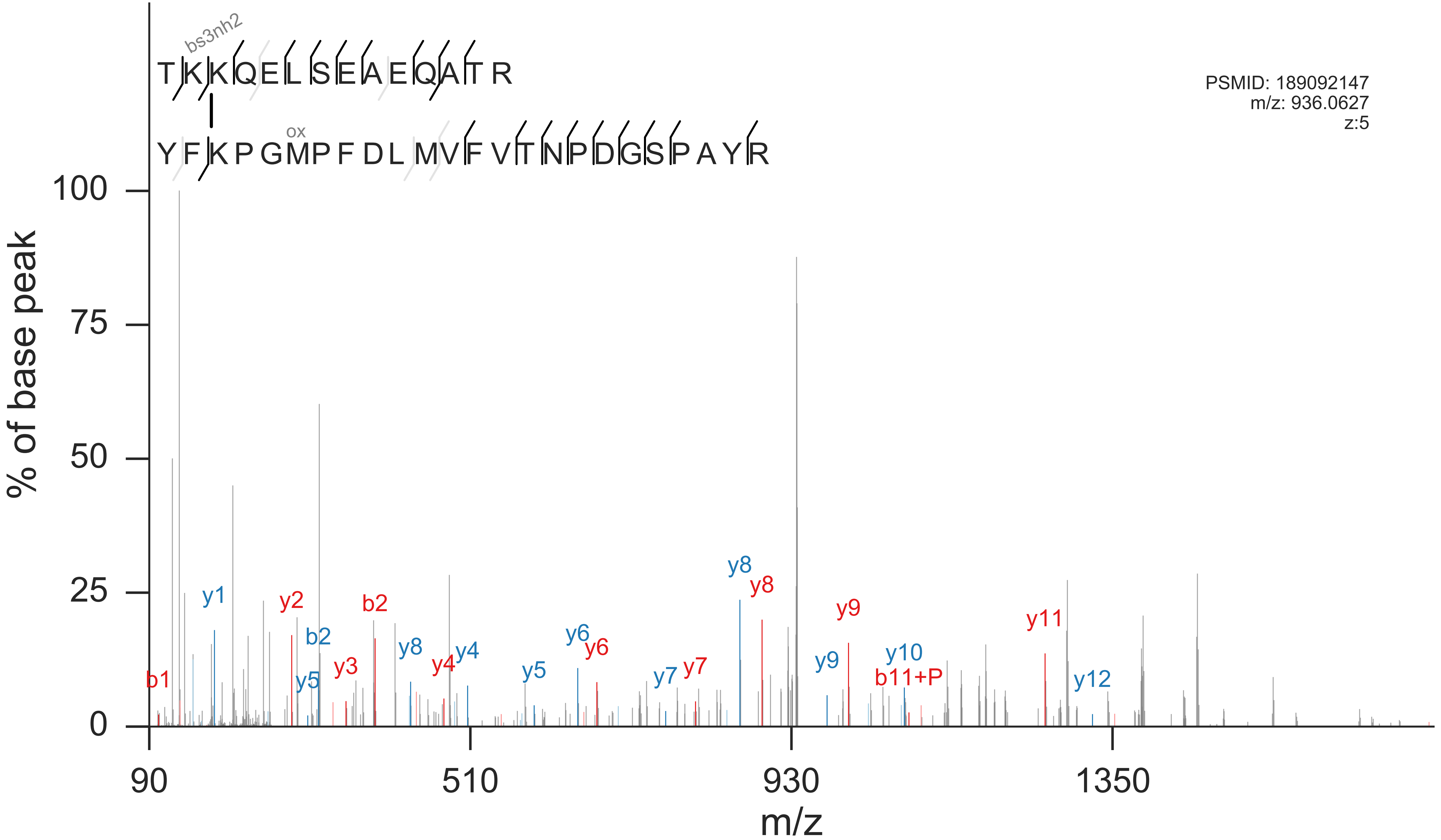

bs3nh2
TKKQELSEAEQATR
ox
YFKPGMPFDLMVFVTNPDGSPAYR
PSMID: 189092147
m/z: 936.0627
z:5
% of base peak
100
75
50
25
0
90
510
930
1350
m/z
b1
y1
y2
b2
y5
y3
b2
y8
y4
y4
y5
y6
y6
y7
y7
y8
y8
y9
y9
y10
b11+P
y11
y12

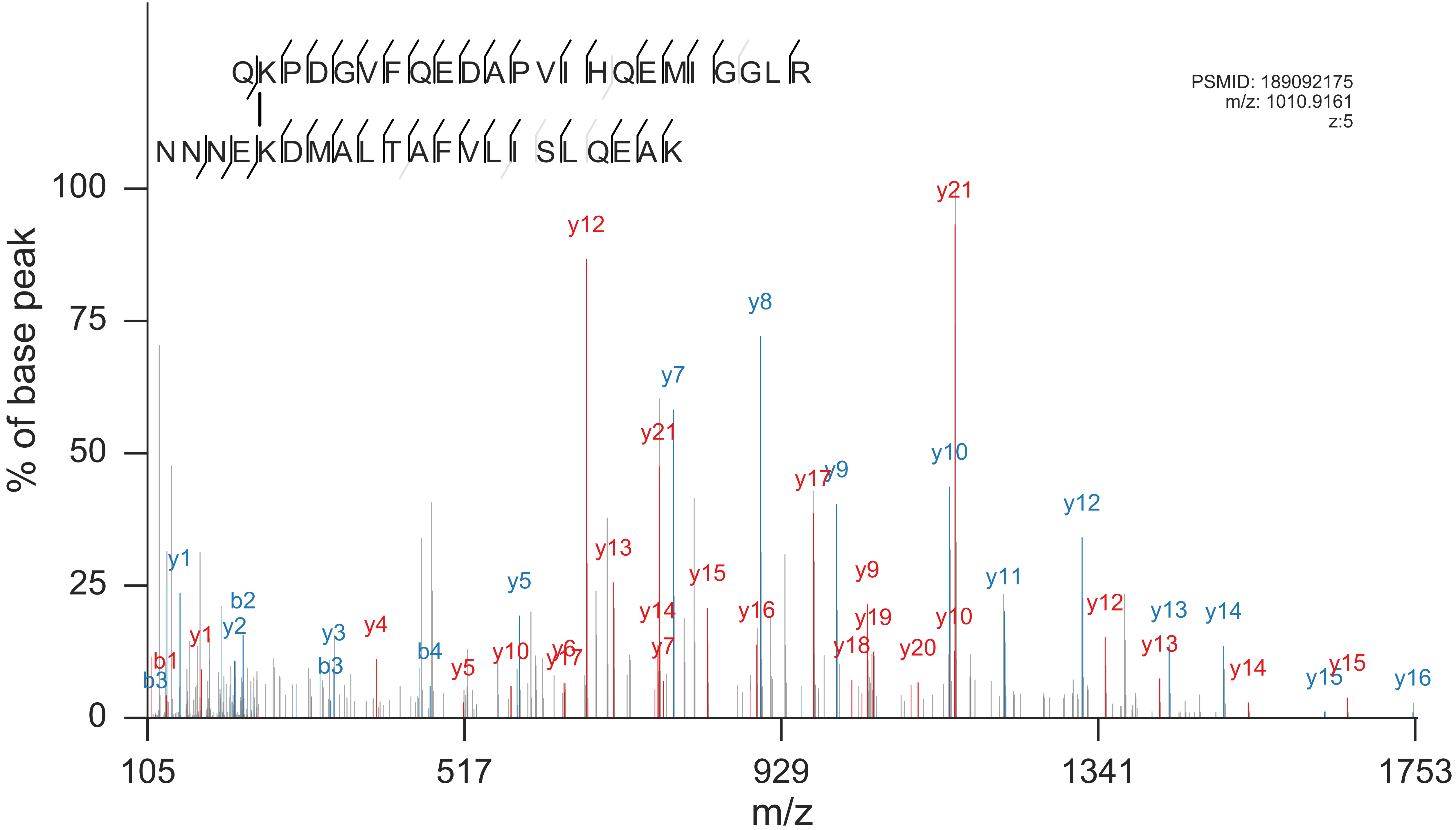
QKPDGVFQEDAPVIHQEMIGGLR
NNNEKDMALTAFVLISLQEAK
PSMID: 189092175
m/z: 1010.9161
z:5
% of base peak
m/z
0
25
50
75
100
105
517
929
1341
1753

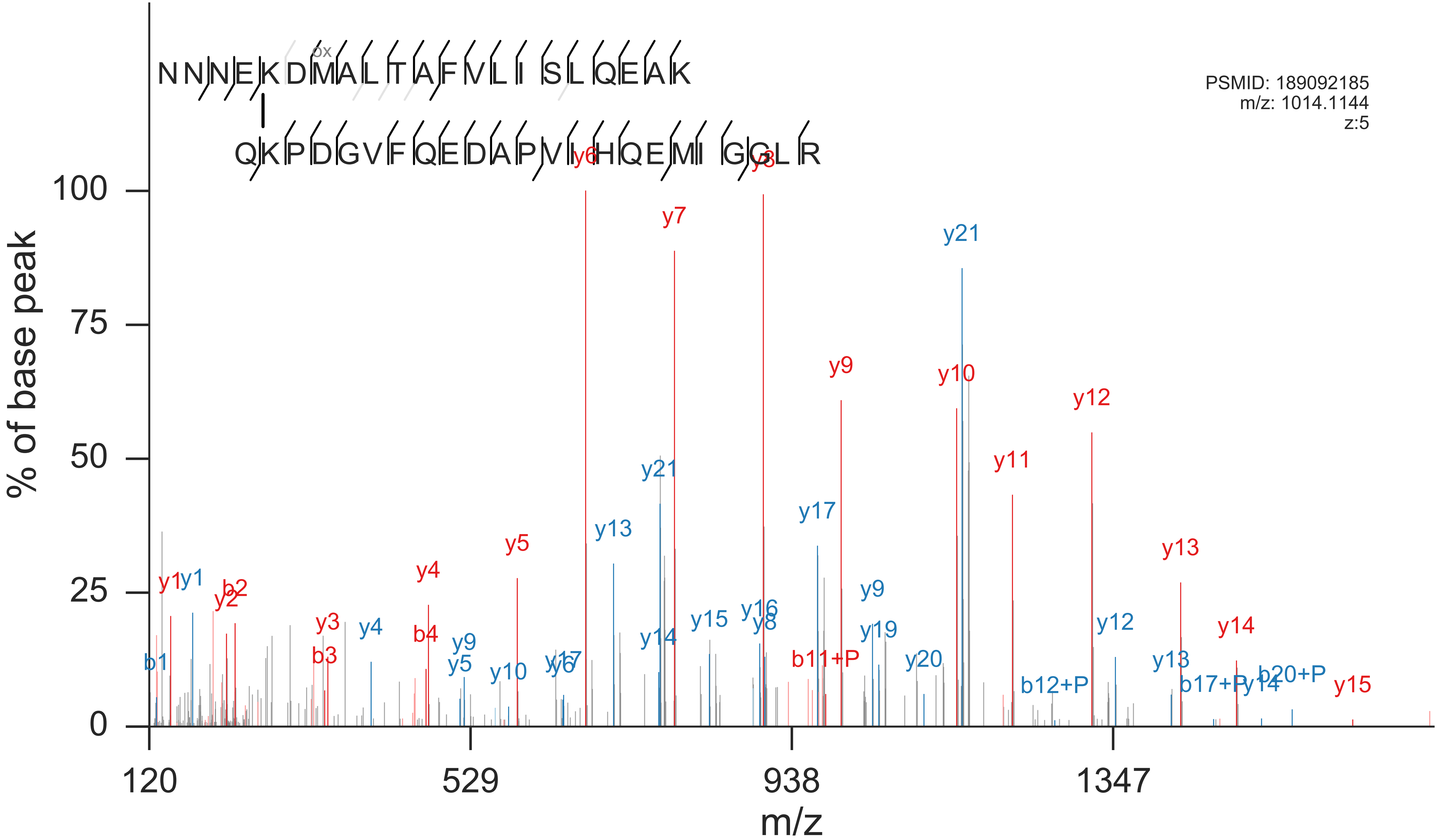

NNNEKDMALTAFVLISLQEAK
ox
QKPDGVFQEDAPVIHQEMIGGLR
PSMID: 189092185
m/z: 1014.1144
z:5
% of base peak
100
75
50
25
0
120
529
938
1347
m/z
